# Supplementary material for: The canonical ER stress IRE1α/XBP1 pathway mediates skeletal muscle wasting during pancreatic cancer cachexia
Source: EMBO Mol Med. 2025 Nov 17;17(12):3607–35. doi: 10.1038/s44321-025-00337-w (PMC12686462; doi:10.1038/s44321-025-00337-w)
Supplement: Supplementary file 1 — Table EV1 [file 44321_2025_337_MOESM1_ESM.docx]

**Table EV1.** List of primer sequences used for qPCR and ChIP assays.

| **Gene Name** | **Forward primer (5'-3')** | **Reverse primer (5'-3')** |
| --- | --- | --- |
| Xbp1 (exon2) | CCTGAGCCCGGAGGAGAA | CTCGAGCAGTCTGCGCTG |
| β-actin | CAGGCATTGCTGACAGGATG | TGCTGATCCACATCTGCTGG |
| Hspa5 (ChIP) | TGGTGGCATGGACCAATCAG | CGCCGACTCGCCTTATATAC |
| Dnajb9 (ChIP) | GAGCCGACCTACACGAAAC | AGGACCAAACGGCAACAA |
| Ern1 | CCTTTGCTGATAGTCTCTGCCCAT | TTACCACCAGTCCATCGCCATT |
| Xbp1 | TGTCCATTCCCAAGCGTGTTCT | TGGAGCAGCAAGTGGATTT |
| sXbp1 | AAGAACACGCTTGGGAATGG | CTGCACCTGCTGCGGAC |
| Becn1 | TGAAATCAATGCTGCCTGGG | CCAGAACAGTATAACGGCAACTCC |
| Map1lc3b | CTGGTGAATGGGCACAGCATG | CGTCCGCTGGTAACATCCCTT |
| Atg12 | ACAAAGAAATGGGCTGTGGAGC | GCAGTAATGCAGGACCAGTTTACC |
| Fbxo32 | GTCGCAGCCAAGAAGAGAAAGA | TGCTATCAGCTCCAACAGCCTT |
| Trim63 | TACTGCATCTCCATGCTGGTG | TGGCGTAGAGGGTGTCAAACTT |
| Fbxo30 | TCGTGGAATGGTAATCTTGC | CCTCCCGTTTCTCTATCACG |
| Ddit3 | TGAAAGCAGAACCTGGTCCA | CACTGTTCATGCTTGGTGCA |
| Dnajb9 | TTAGCCATGAAGTACCACCCTGAC | TTCCGACTATTGGCATCCGA |
| Edem1 | CGGCTATGACAACTACATGG | GTTCAGATTGGACTCTC |
| Eif2ak3 | ACTCCTGTCTTGGTTGGGTCTGAT | CGTGCTCCGCTTATTCCTTTCT |
| Bloc1s1 | GCCTACATGAACCAGAGAAAG | GTTCTCCACCATTCCAATCC |
| Pdgfr | CCAAGTCAGGTCCCATTTAC | GGTCTTTCTTCGGCTTCTC |
| Scara3 | GGGTTTCTATGGCTGGTTAG | TGCAGAGACCAGAGTAGTT |
| Sparc | CAACTGCAATTGGGCTTTC | ACCAGTCTCACTTCCTCTAC |
| Ppard | TCCATCGTCAACAAAGACGGG | ACTTGGGCTCAATGATGTCAC |
| Ppargc1a | TGGAGTGACATAGAGTGTGCTGC | CTCAAATATGTTCGCAGGCTCA |
| Cd36 | GGCCAAGCTATTGCGACAT | CAGATCCGAACACAGCGTAGA |
| Acox1 | GGATGGTAGTCCGGAGAACA | AGTCTGGATCGTTCAGAATCAAG |
| Acox2 | CCTTCCTAGACCTGCTTCCC | TGTCCGTCATAACAGCCAAG |
| Acox3 | CTTCTGAGAAACGGGGACAA | GCTCGGTAGGCACTAAGAGG |
| Sirt1 | GACGATGACAGAACGTCACAC | CGAGGATCGGTGCCAATCA |
| Hif1a | TGAGCTTGCTCATCAGTTGC | CCATCTGTGCCTTCATCTCA |
| Hadhb | GCACTTTCGGGTTTGTTG | GTGTGAGCTGGAGTCTTATC |
| Irisin (Fndc5) | CACAGAATATATCGTCCATG | GTCACCTCATCTTTGTTCTT |
| Atg5 | ATCAGACCACGACGGAGCGG | GGCGACTGCGGAAGGACAGA |
| Il6 | CCTTCTTGGGACTGATGCTGG | GCCTCCGACTTGTGAAGTGGT |
| Pdk4 | AAAGGACAGGATGGAAGGAATCA | TTTTCCTCTGGGTTTGCACAT |
| MAFbx (ChIP) | CCTCGGAAAACAAGGCGAG | GTCTCTTTGTTGCCGGAAGA |
| LC3b (ChIP) | GTTAACAGATGCTCGCCCAG | TGTGTGTCTCAGTCCGCAG |
| Atg5 (ChIP) | TTCCGAGTTCAGGCGCTC | GAACCAGAGTGAACCGCAG |
| Xbp1 (ChIP) | CCCGGGACTACAGGACCA | CCACCACCACCATAGCCA |
| Il6 (ChIP) | CTCATGCTTCTTAGGGCTAGC | GAGTGGGTGGGGCTGATT |
| Pdk4 (ChIP) | AAACAAGGACAAGTCTGGGC | TCACTAGAAAGGCCTGGCAC |
