# Supplementary material for: The canonical ER stress IRE1α/XBP1 pathway mediates skeletal muscle wasting during pancreatic cancer cachexia
Source: EMBO Mol Med. 2025 Nov 17;17(12):3607–35. doi: 10.1038/s44321-025-00337-w (PMC12686462; doi:10.1038/s44321-025-00337-w)
Supplement: Supplementary file 2 — Table EV2 [file 44321_2025_337_MOESM2_ESM.docx]

**Table EV2.** Average TPM values for control mice

| **Gene** | **Avg. TPM** | **Gene** | **Avg. TPM** | **Gene** | **Avg. TPM** | **Gene** | **Avg. TPM** | **Gene** | **Avg. TPM** |
| --- | --- | --- | --- | --- | --- | --- | --- | --- | --- |
| Ubqln4 | 40.560 | Cdk16 | 60.291 | Snx9 | 13.452 | Ctsl | 53.734 | Acox1 | 30.195 |
| Sec23a | 21.400 | Siah1a | 5.633 | Wwp2 | 4.556 | Map1lc3b | 79.470 | Plin5 | 8.484 |
| Ube2j1 | 13.683 | Ripk2 | 1.086 | Ppp2ca | 21.888 | Bnip3 | 150.388 | Cyp4f13 | 6.202 |
| Uggt1 | 4.801 | Sox17 | 6.869 | Abhd5 | 14.324 | Edem2 | 3.000 | Por | 10.225 |
| Hsp90ab1 | 511.050 | Mylip | 6.343 | Adam9 | 18.235 | Dnajb9 | 18.080 | Abcd1 | 8.015 |
| Eif2ak1 | 13.608 | Zer1 | 9.286 | Pink1 | 316.814 | Rnf121 | 10.078 | Acadsb | 46.350 |
| Sel1l | 9.256 | Prickle1 | 1.311 | Igf1r | 1.503 | Get4 | 23.249 | Ppard | 9.168 |
| Sar1a | 63.835 | Abca2 | 3.934 | Atg4d | 33.443 | Dnajc10 | 7.647 | Echdc1 | 8.069 |
| Ube4b | 14.744 | Pan2 | 2.947 | Atg101 | 27.022 | Rnf5 | 17.772 | Acox3 | 6.484 |
| Nploc4 | 19.388 | Mgrn1 | 21.404 | Atg4b | 9.843 | Syvn1 | 7.863 | Adipor2 | 27.850 |
| Dnajb12 | 25.350 | Cnot2 | 11.504 | Pik3c3 | 7.219 | Sgta | 53.460 | Acacb | 42.548 |
| Xbp1 | 28.959 | Khsrp | 6.982 | Hif1a | 4.634 | Usp19 | 49.324 | Crat | 132.295 |
| Dnaja2 | 86.389 | Fto | 31.122 | Ctsb | 92.448 | Man1b1 | 5.761 | Pex7 | 14.007 |
| Sec31b | 5.033 | Pip4k2b | 14.309 | Ambra1 | 5.278 | Ube2g2 | 46.501 | Hacl1 | 6.454 |
| Rad23a | 80.304 | Zc3h18 | 9.096 | Smcr8 | 2.811 | Stat5a | 3.486 | Acad10 | 1.680 |
| Stt3b | 26.017 | Tpcn1 | 15.327 | Usp8 | 5.099 | Stat1 | 5.025 | Gcdh | 16.737 |
| Eif2ak4 | 1.249 | Huwe1 | 14.918 | Atg9a | 45.830 | Mcl1 | 62.713 | Ivd | 65.615 |
| Map2k7 | 27.178 | Mid1 | 3.076 | Tax1bp1 | 30.003 | Csf2rb2 | 0.532 | Slc25a17 | 6.535 |
| Ubxn4 | 21.071 | Cblb | 2.069 | Akt2 | 91.849 | Csf2rb | 0.465 | Klhl25 | 1.580 |
| Rad23b | 40.924 | Ubc | 1111.328 | Optn | 29.451 | Jak3 | 1.893 | Dgat1 | 9.182 |
| Ubqln1 | 19.482 | Trim63 | 127.276 | Mapk1 | 33.787 | Il4ra | 2.008 | Pex5 | 10.411 |
| Rab12 | 152.817 | Fbxo32 | 27.393 | Mtmr4 | 1.942 | Tyk2 | 2.101 | Tysnd1 | 6.776 |
| Ddb1 | 59.740 | Zswim8 | 12.448 | Uvrag | 2.447 | Stam | 4.011 | Etfb | 254.387 |
| Zfp36l2 | 5.323 | Supt5 | 22.064 | Ddit4 | 27.123 | Stat2 | 2.084 | Scp2 | 125.223 |
| Ube2o | 6.575 | Ube2b | 372.150 | Dapk3 | 14.855 | Mtor | 5.761 | Adh5 | 46.870 |
| Vps11 | 8.044 | Cdc23 | 6.832 | Bcl2l1 | 8.863 | Osmr | 1.292 | Eci2 | 61.269 |
| Fbxw8 | 6.031 | Exosc8 | 7.630 | Tbc1d15 | 10.628 | Pik3r1 | 3.342 | Echdc2 | 8.136 |
| Sptlc2 | 3.754 | Sting1 | 2.009 | Foxo3 | 3.185 | Il6ra | 0.754 | Akt1 | 23.419 |
| Nub1 | 15.236 | Ralb | 4.303 | Rab7 | 135.455 | Stat6 | 7.432 | Eci1 | 103.961 |
| Clu | 37.495 | Slc25a5 | 36.903 | Pik3r1 | 3.342 | Aox1 | 4.489 | Mlycd | 48.059 |
| Pip4k2c | 4.647 | Zc3hav1 | 1.664 | Rhot2 | 32.676 | Il6st | 23.087 | Decr1 | 38.108 |
| Anapc5 | 259.659 | Ubox5 | 1.903 | Ulk1 | 18.231 | Stat3 | 11.730 | Auh | 38.259 |
| Ube3b | 48.180 | Nsf | 4.456 | Atg13 | 12.485 | C1qtnf9 | 14.000 | Acad12 | 6.596 |
| Gga1 | 20.818 | Il1b | 0.209 | Atg2a | 4.942 | Pdk4 | 138.439 |  |  |
| Upf1 | 9.204 | Fxr1 | 203.387 | Sqstm1 | 305.650 | Ppargc1a | 4.292 |  |  |
| Wdr24 | 4.826 | Mlh1 | 4.466 | Bnip3l | 55.155 | Cpt1b | 115.817 |  |  |
| Trim68 | 3.692 | Tardbp | 66.167 | Lamp1 | 201.108 | Acadm | 189.071 |  |  |
