# Supplementary figures and images for: The canonical ER stress IRE1α/XBP1 pathway mediates skeletal muscle wasting during pancreatic cancer cachexia (part 1 of 2)
Source: EMBO Mol Med. 2025 Nov 17;17(12):3607–35. doi: 10.1038/s44321-025-00337-w (PMC12686462; doi:10.1038/s44321-025-00337-w)

## Slide 1
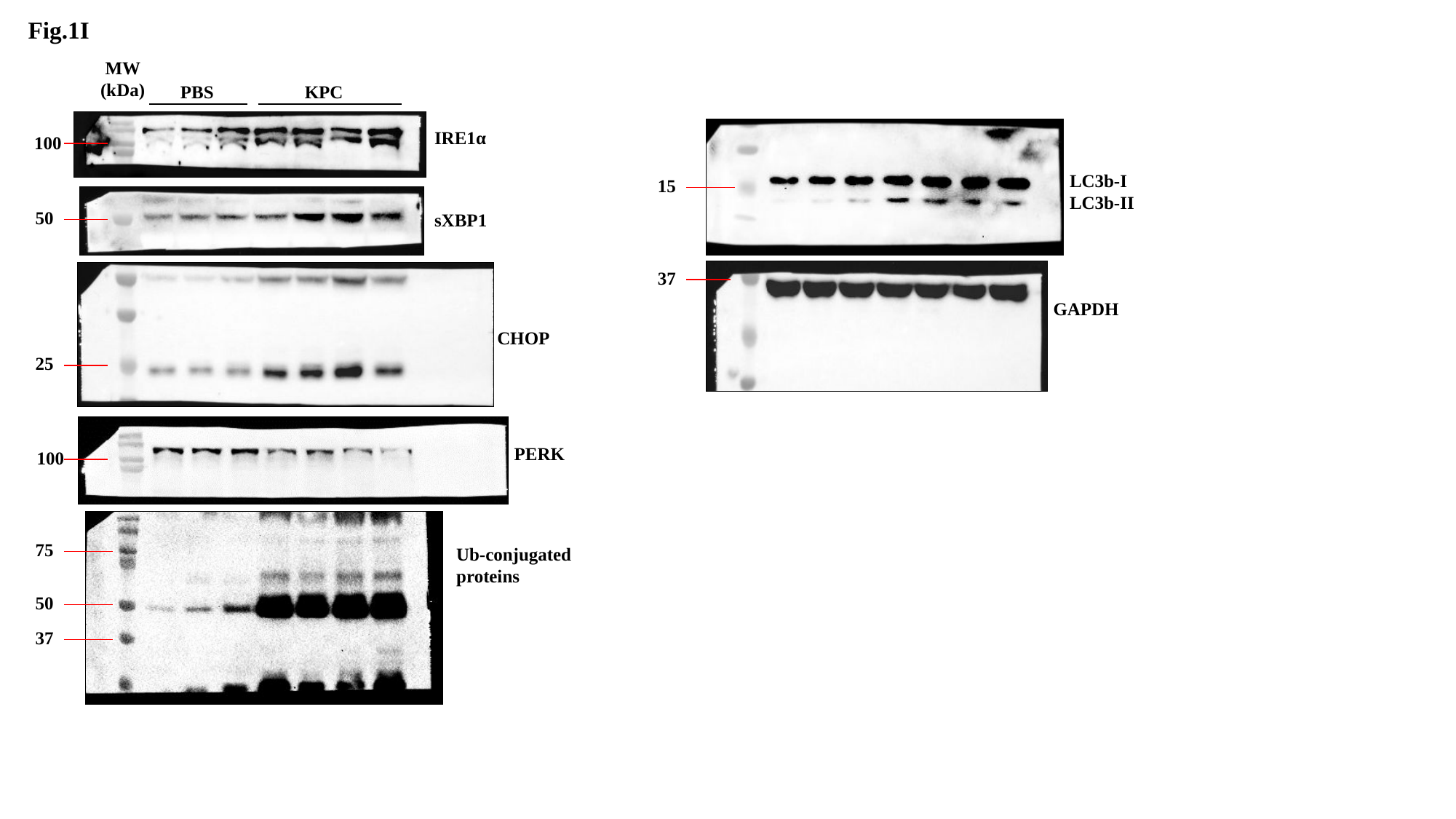

Fig.1I
MW (kDa)
PBS
KPC
IRE1α
100
LC3b-I
LC3b-II
15
50
sXBP1
37
GAPDH
CHOP
25
PERK
100
75
Ub-conjugated proteins
50
37

Supplement: Supplementary file 4 — Source data Fig. 1 [file 44321_2025_337_MOESM4_ESM.zip › Figure 1/Western blot Figure 1I/Western blot Fig1I.pptx]

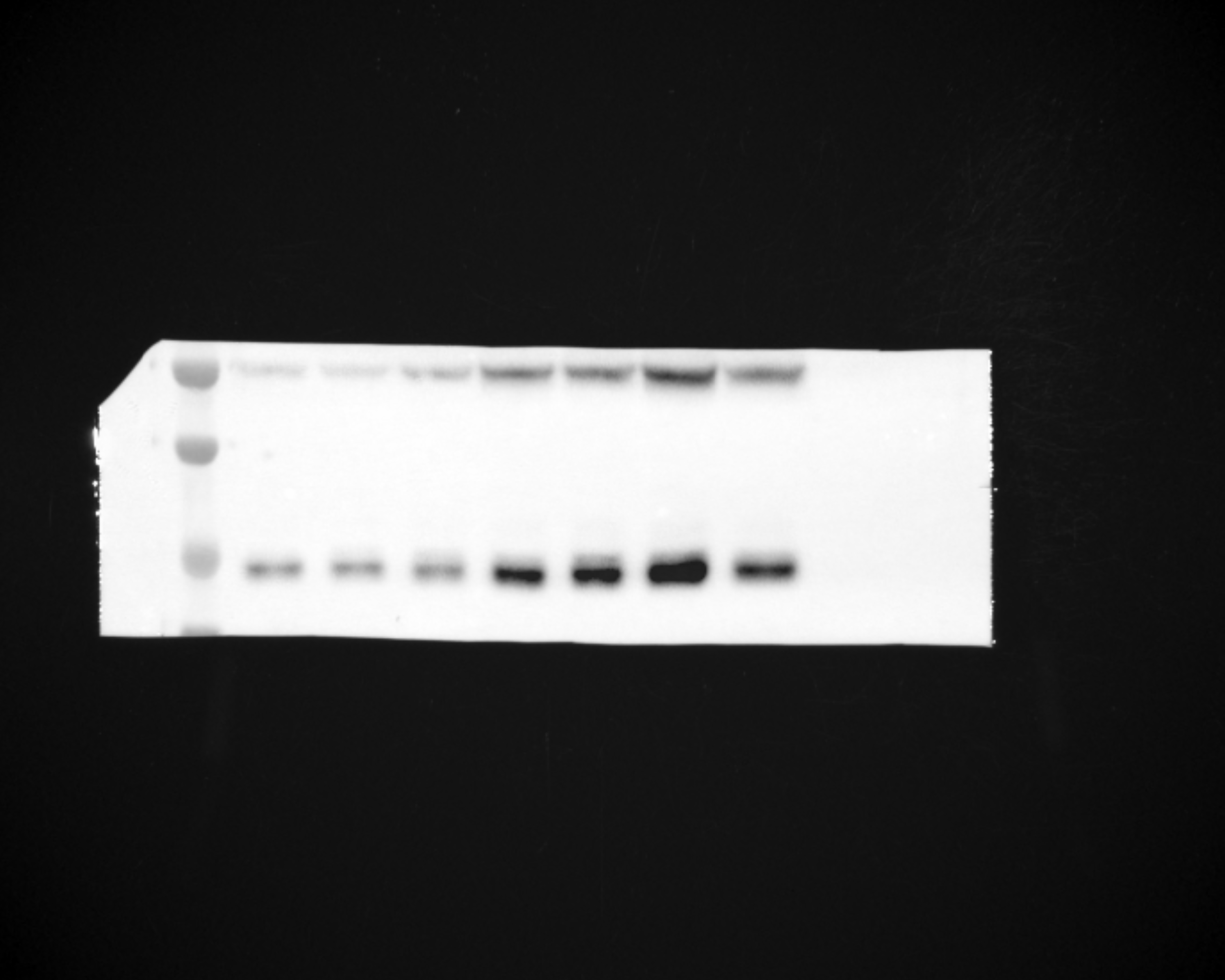

Supplement: Supplementary file 4 — Source data Fig. 1 [file 44321_2025_337_MOESM4_ESM.zip › Figure 1/Western blot Figure 1I/Western CHOP.tif]

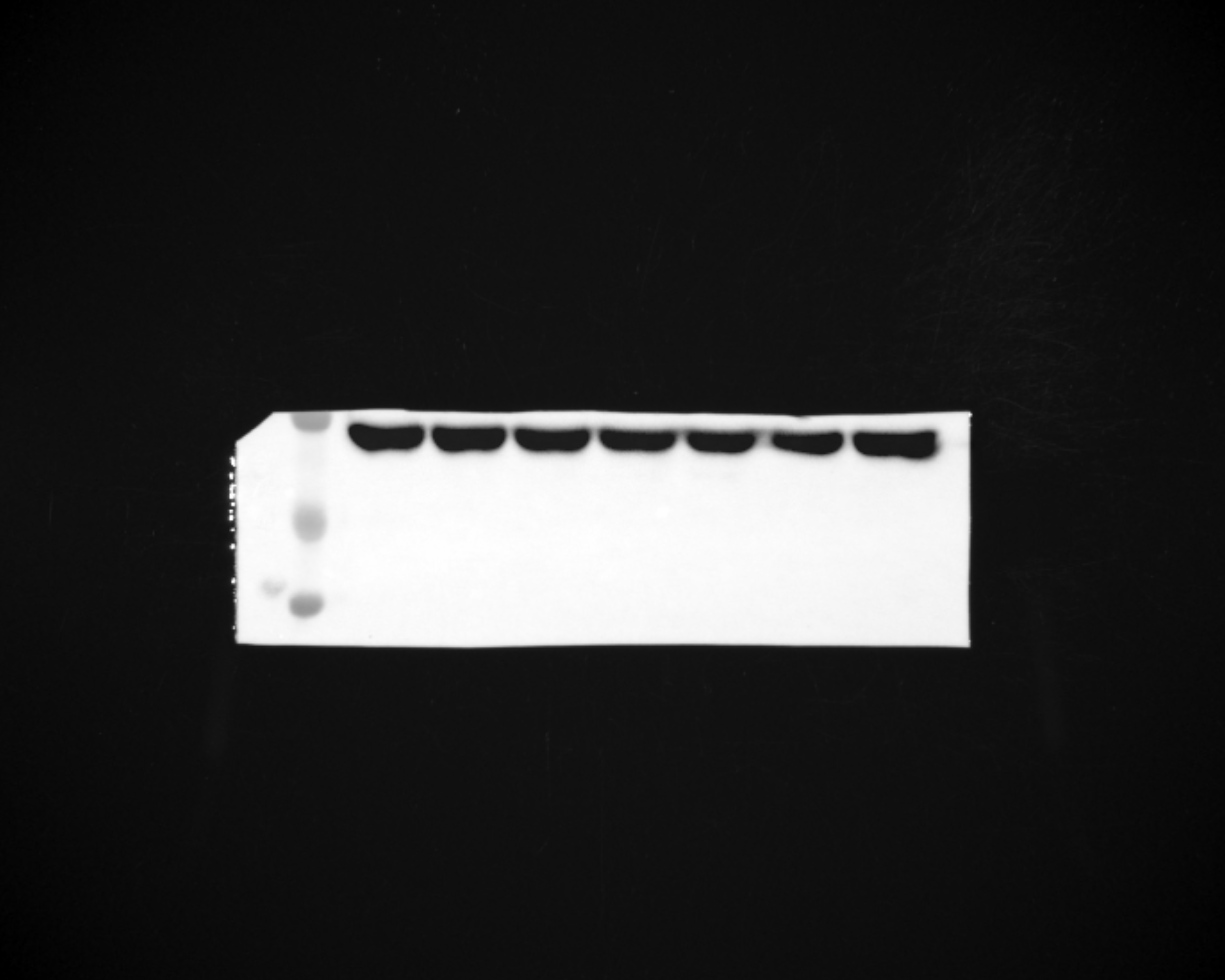

Supplement: Supplementary file 4 — Source data Fig. 1 [file 44321_2025_337_MOESM4_ESM.zip › Figure 1/Western blot Figure 1I/Western GAPDH.tif]

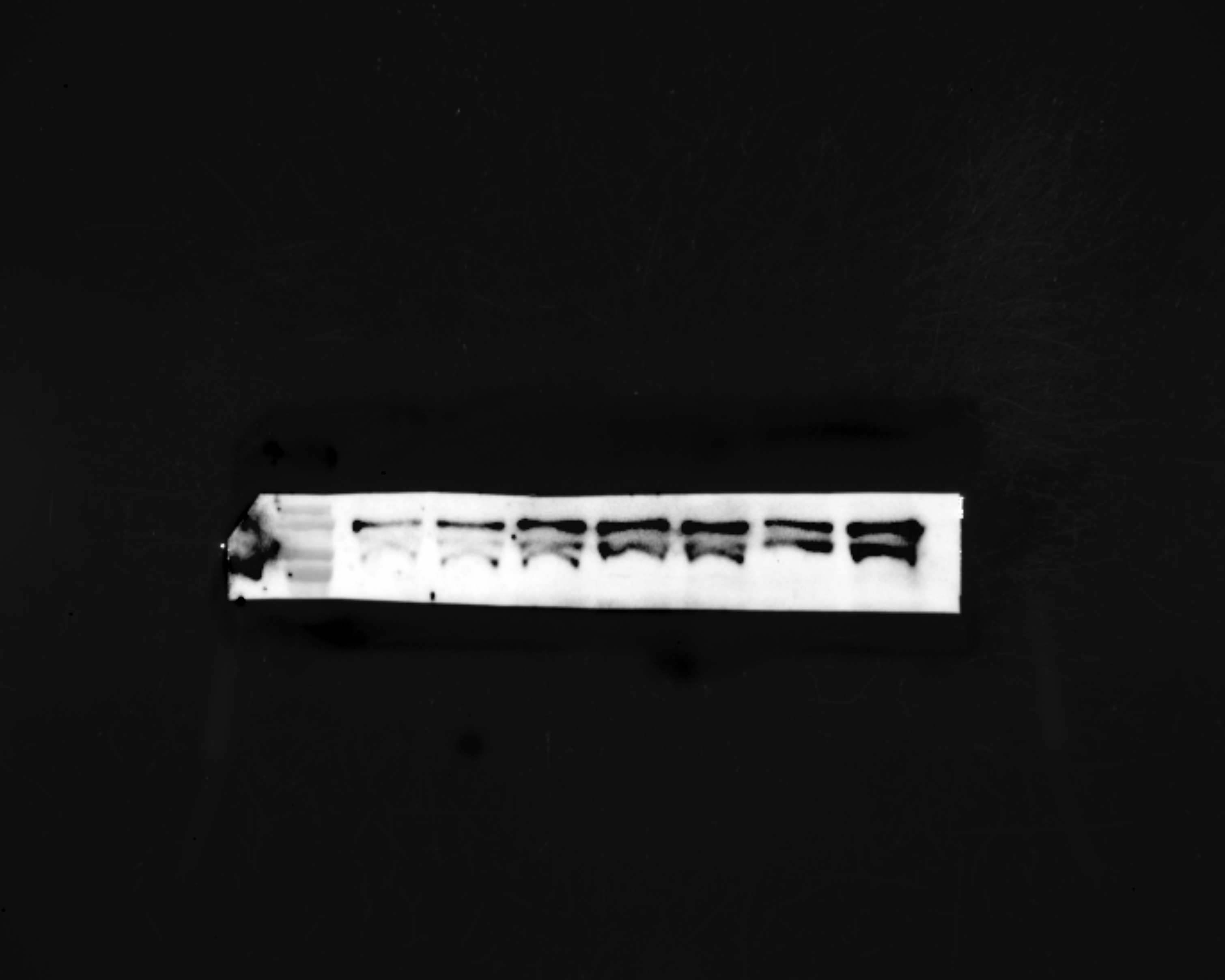

Supplement: Supplementary file 4 — Source data Fig. 1 [file 44321_2025_337_MOESM4_ESM.zip › Figure 1/Western blot Figure 1I/Western IRE1a.tif]

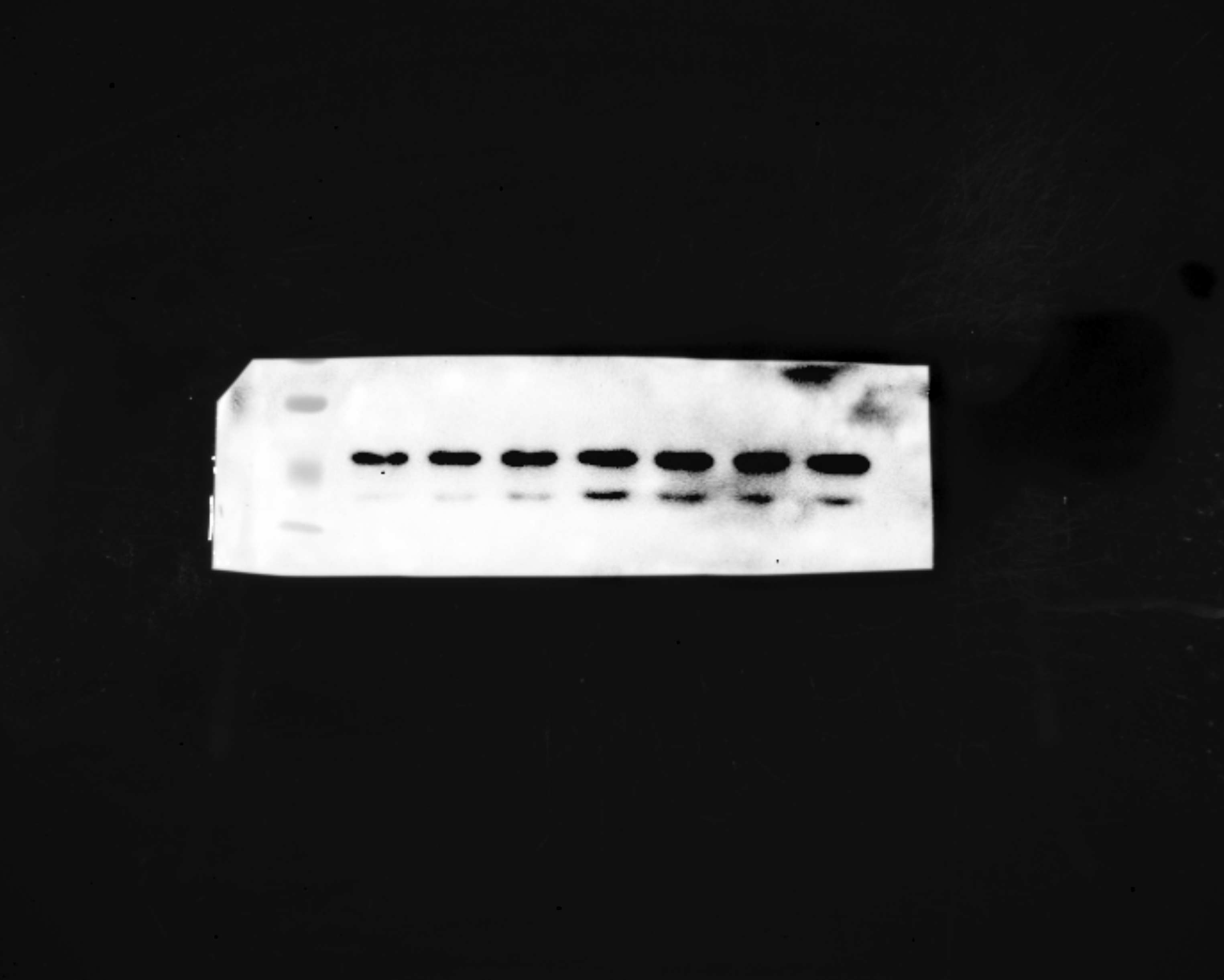

Supplement: Supplementary file 4 — Source data Fig. 1 [file 44321_2025_337_MOESM4_ESM.zip › Figure 1/Western blot Figure 1I/Western LC3B.tif]

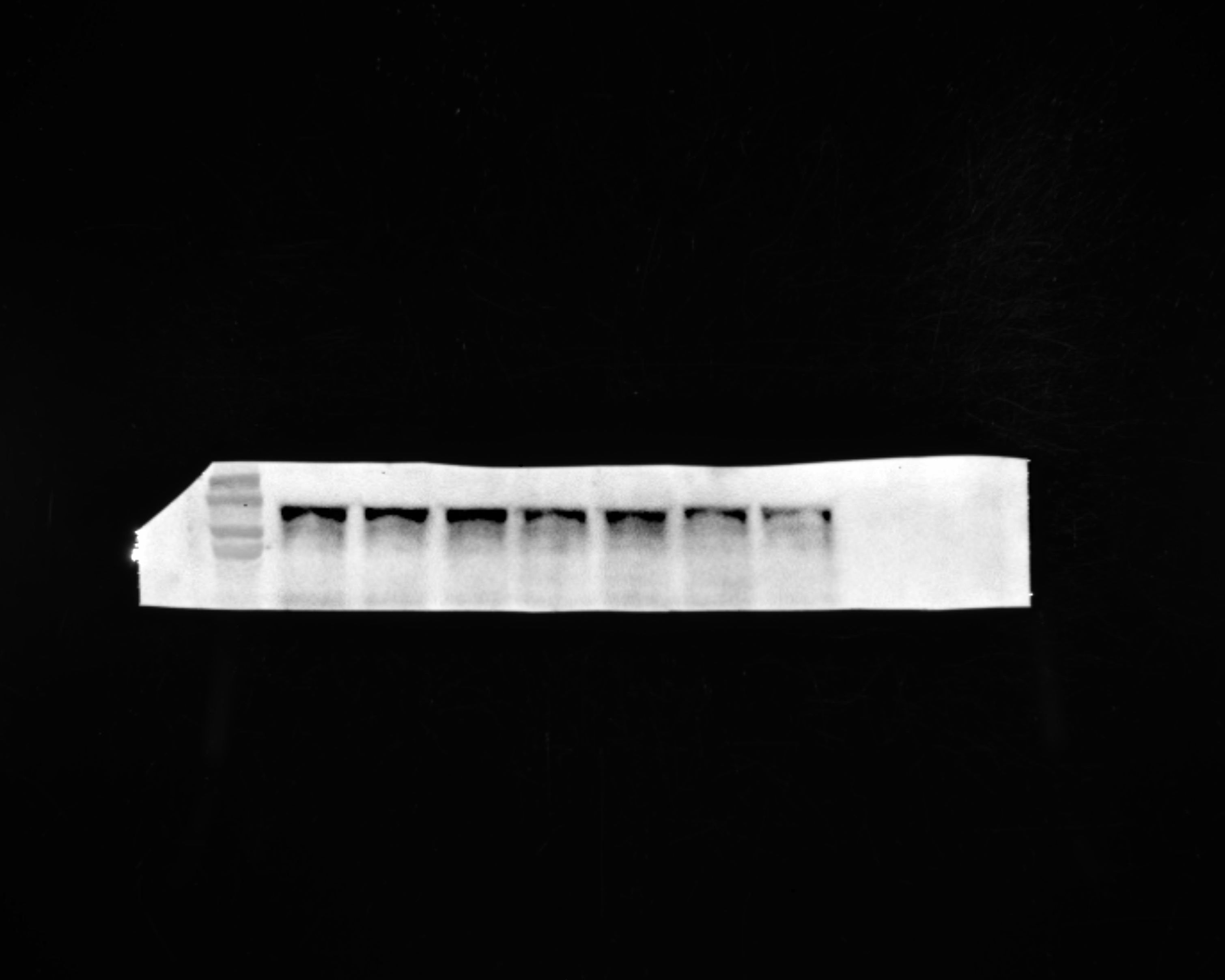

Supplement: Supplementary file 4 — Source data Fig. 1 [file 44321_2025_337_MOESM4_ESM.zip › Figure 1/Western blot Figure 1I/Western PERK.tif]

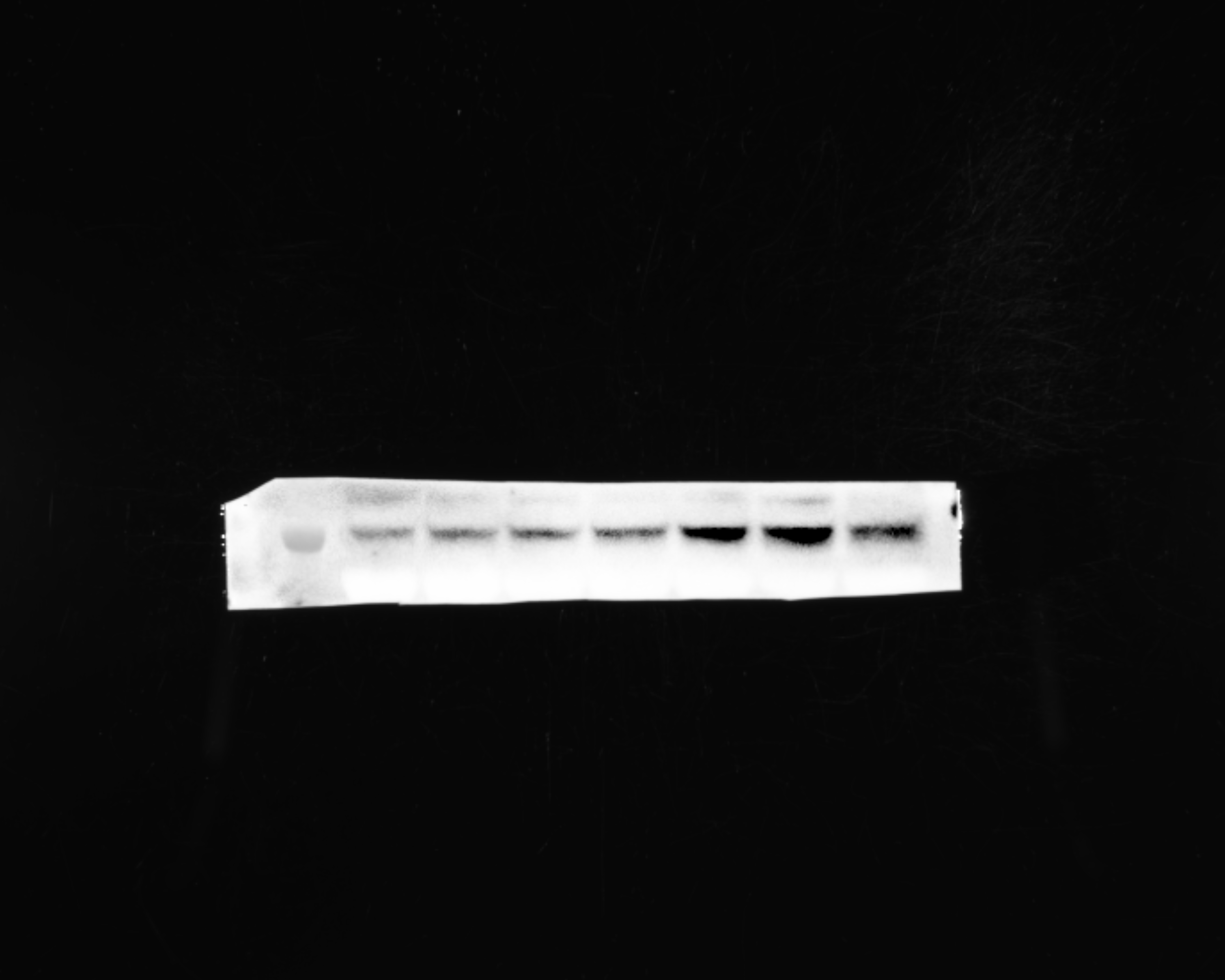

Supplement: Supplementary file 4 — Source data Fig. 1 [file 44321_2025_337_MOESM4_ESM.zip › Figure 1/Western blot Figure 1I/Western sXBP1.tif]

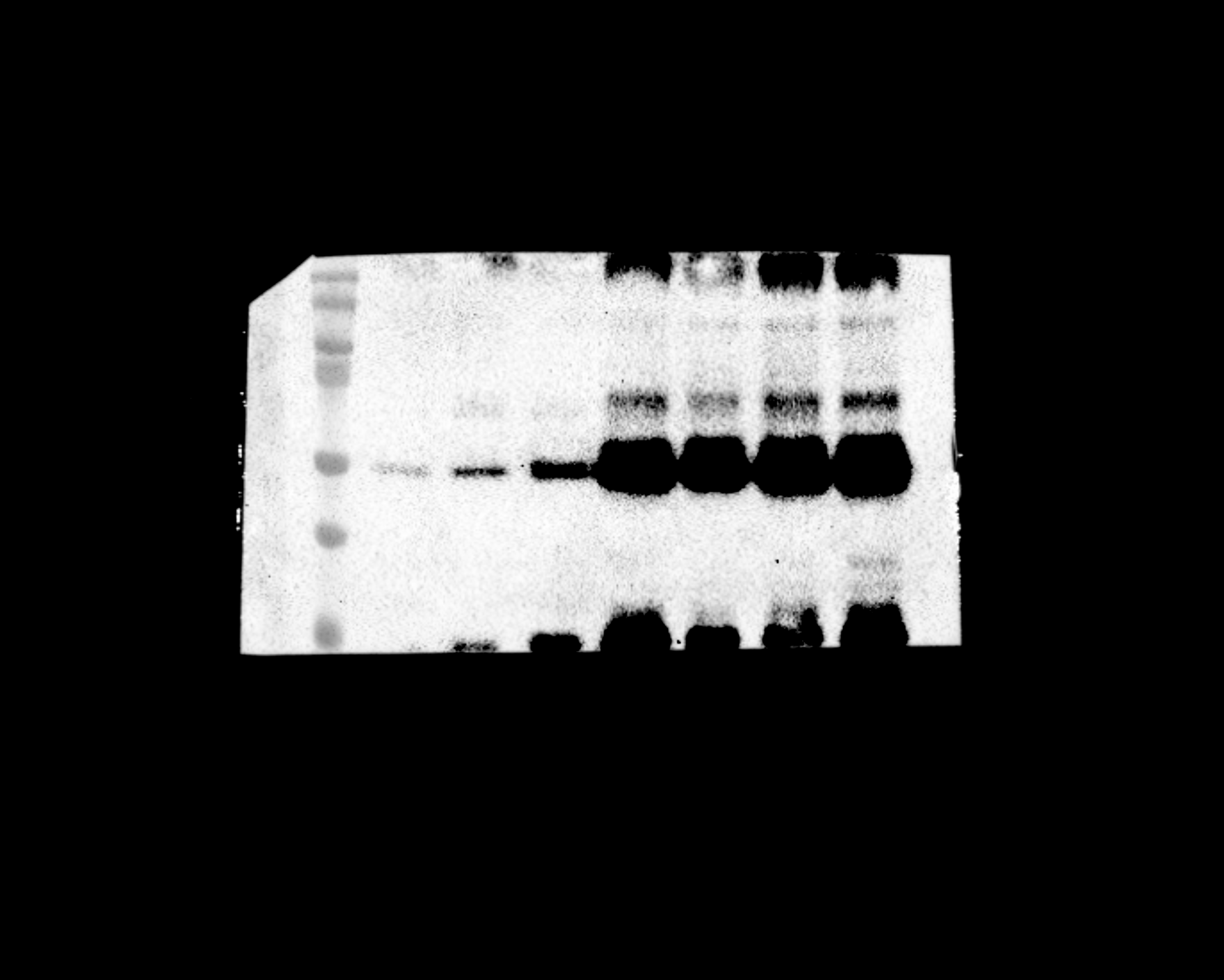

Supplement: Supplementary file 4 — Source data Fig. 1 [file 44321_2025_337_MOESM4_ESM.zip › Figure 1/Western blot Figure 1I/Western Ub-conjugated proteins.tif]

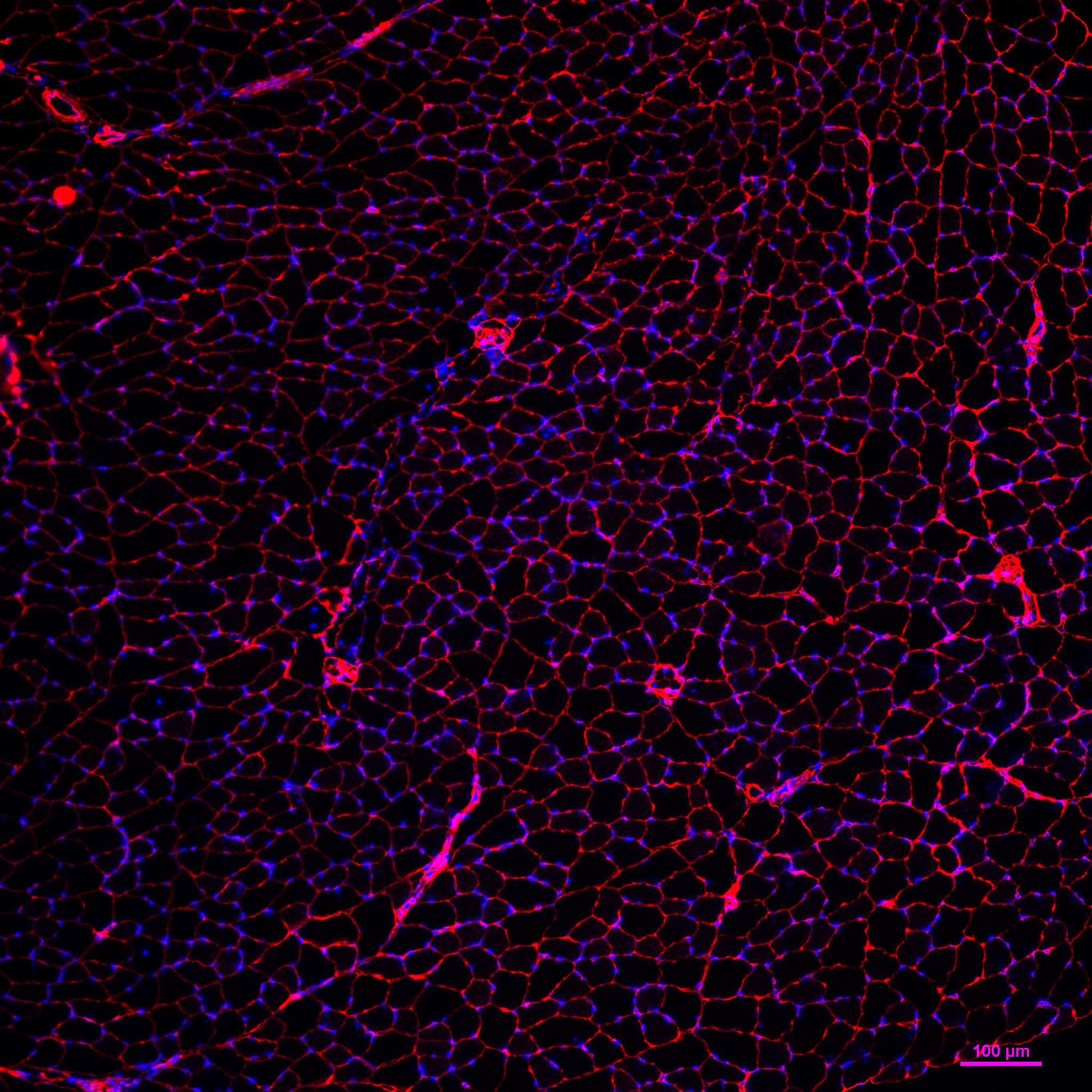

Supplement: Supplementary file 5 — Source data Fig. 2 [file 44321_2025_337_MOESM5_ESM.zip › Figure 2/Fig2G/TA muscle_Laminin-DAPI staining representative images/Xbp1-flfl-KPC.png]

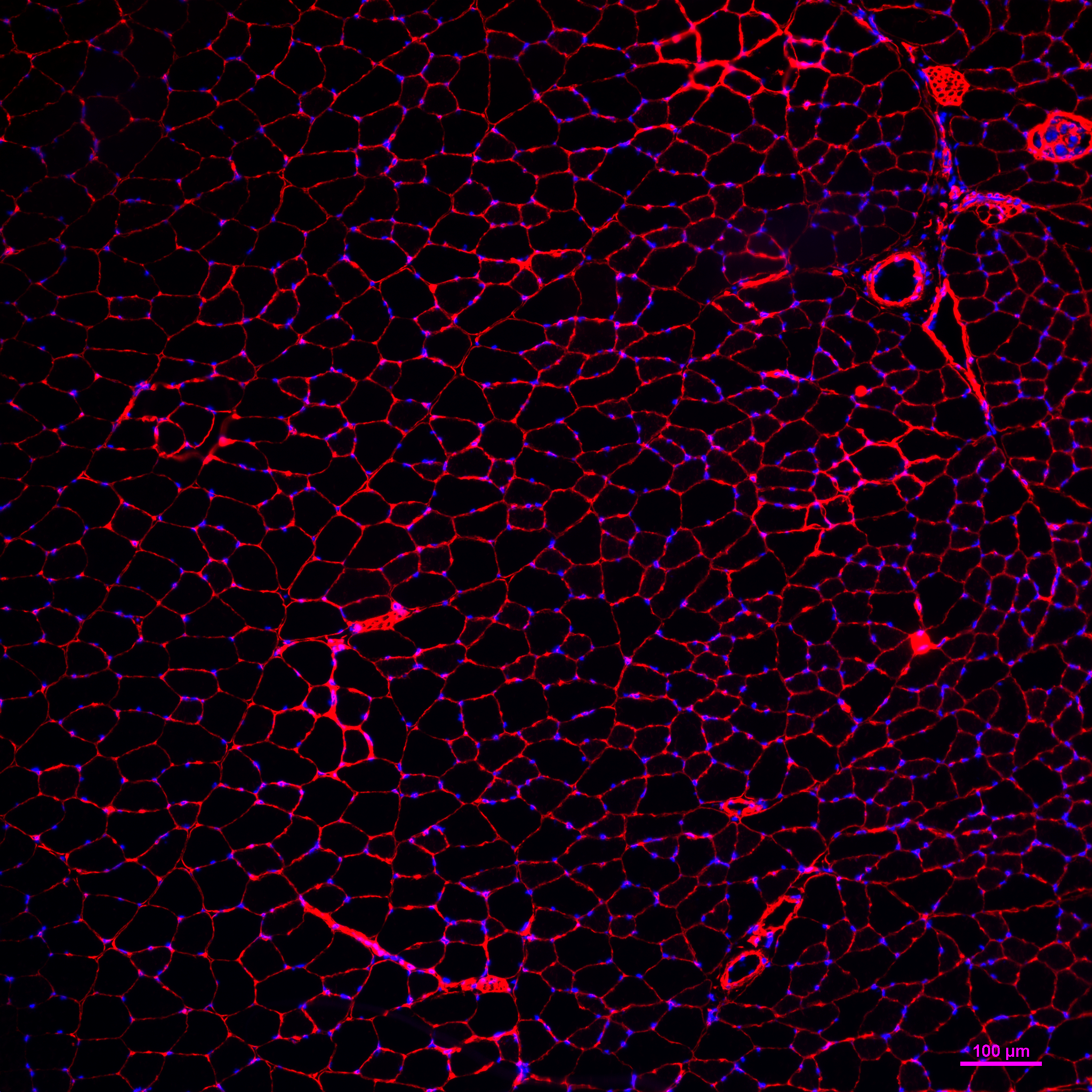

Supplement: Supplementary file 5 — Source data Fig. 2 [file 44321_2025_337_MOESM5_ESM.zip › Figure 2/Fig2G/TA muscle_Laminin-DAPI staining representative images/Xbp1-flfl-PBS.png]

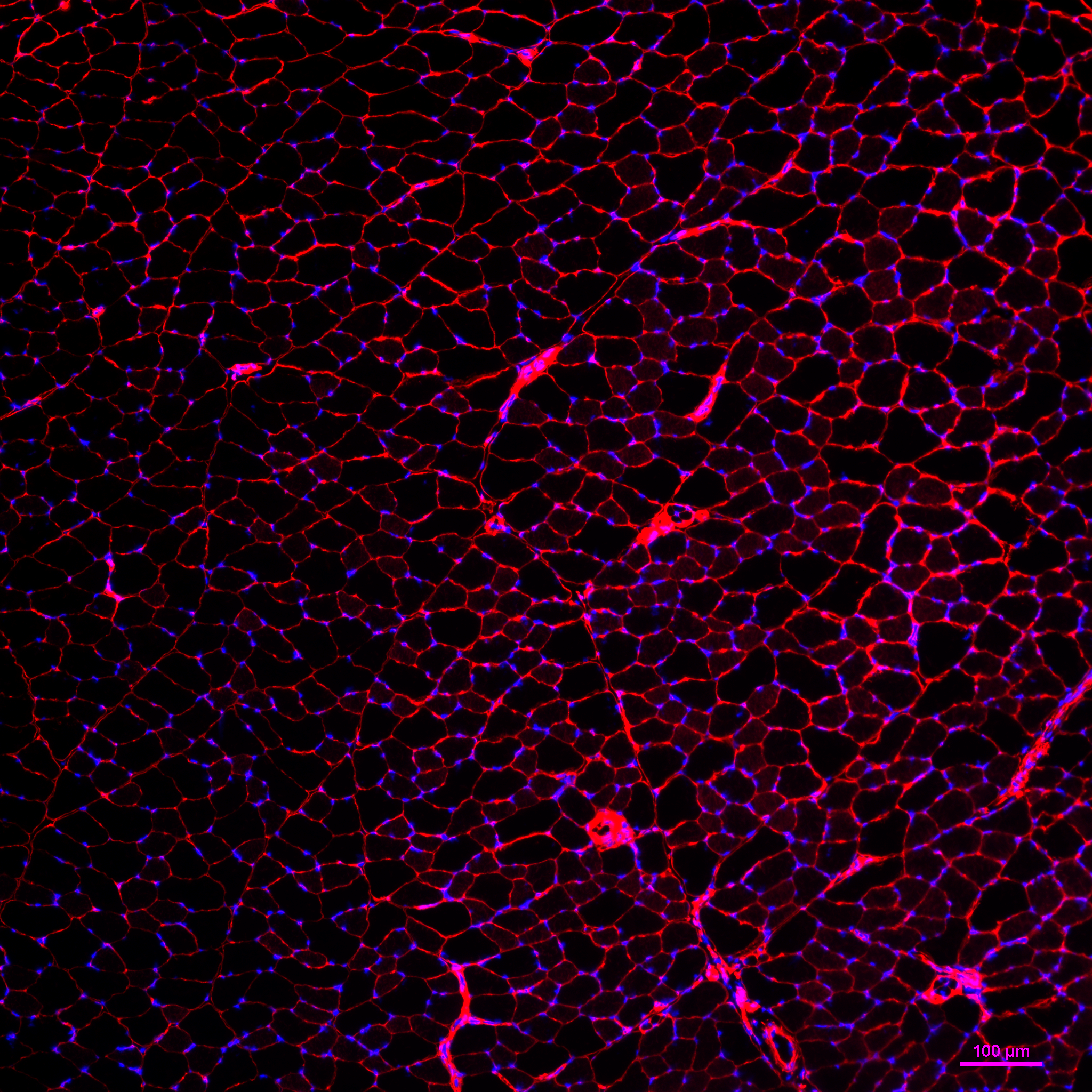

Supplement: Supplementary file 5 — Source data Fig. 2 [file 44321_2025_337_MOESM5_ESM.zip › Figure 2/Fig2G/TA muscle_Laminin-DAPI staining representative images/Xbp1-mKO-KPC.png]

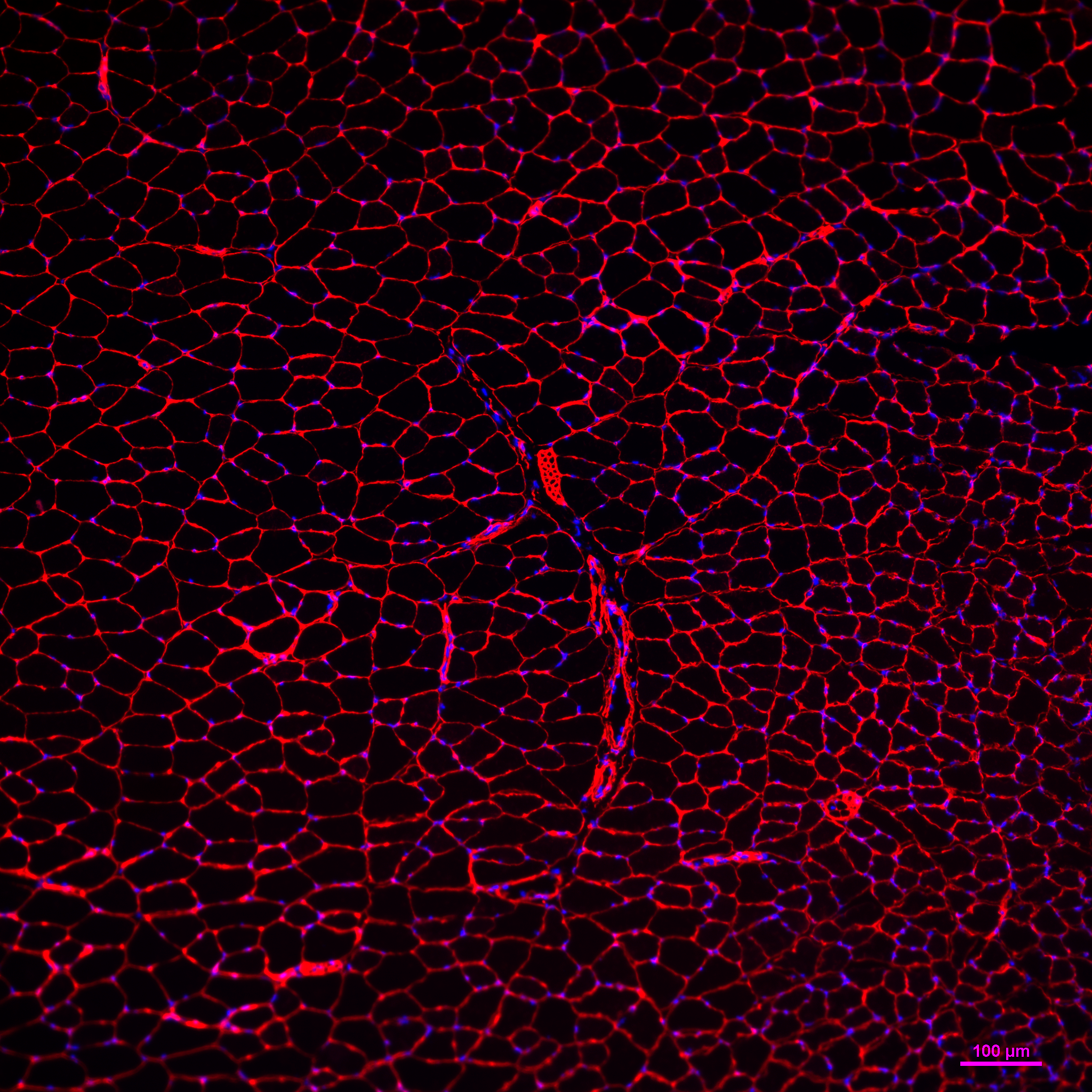

Supplement: Supplementary file 5 — Source data Fig. 2 [file 44321_2025_337_MOESM5_ESM.zip › Figure 2/Fig2G/TA muscle_Laminin-DAPI staining representative images/Xbp1-mKO-PBS.png]

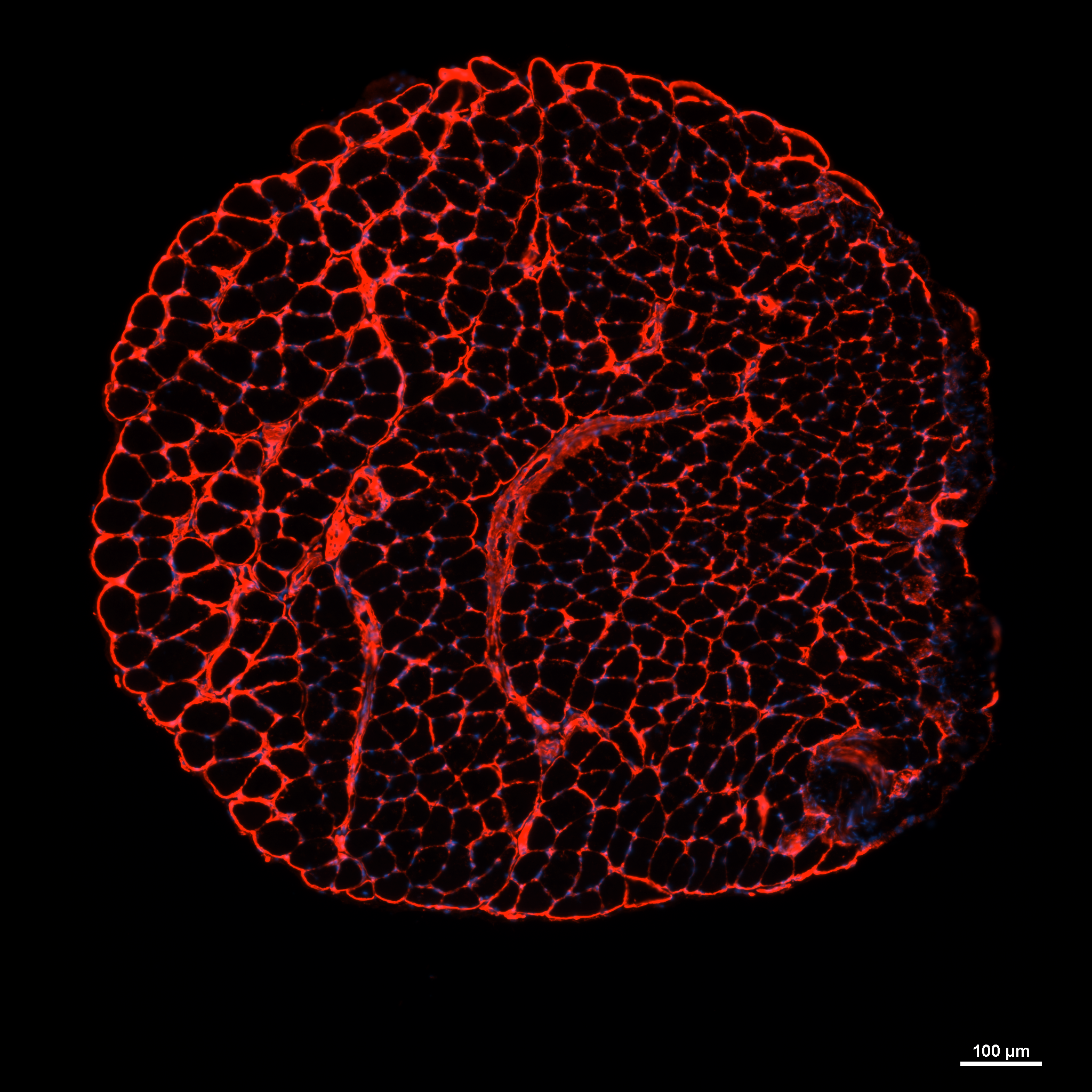

Supplement: Supplementary file 5 — Source data Fig. 2 [file 44321_2025_337_MOESM5_ESM.zip › Figure 2/Fig2K/Soleus muscle_Laminin-DAPI staining representative images/Xbp1_flfl_KPC.tif]

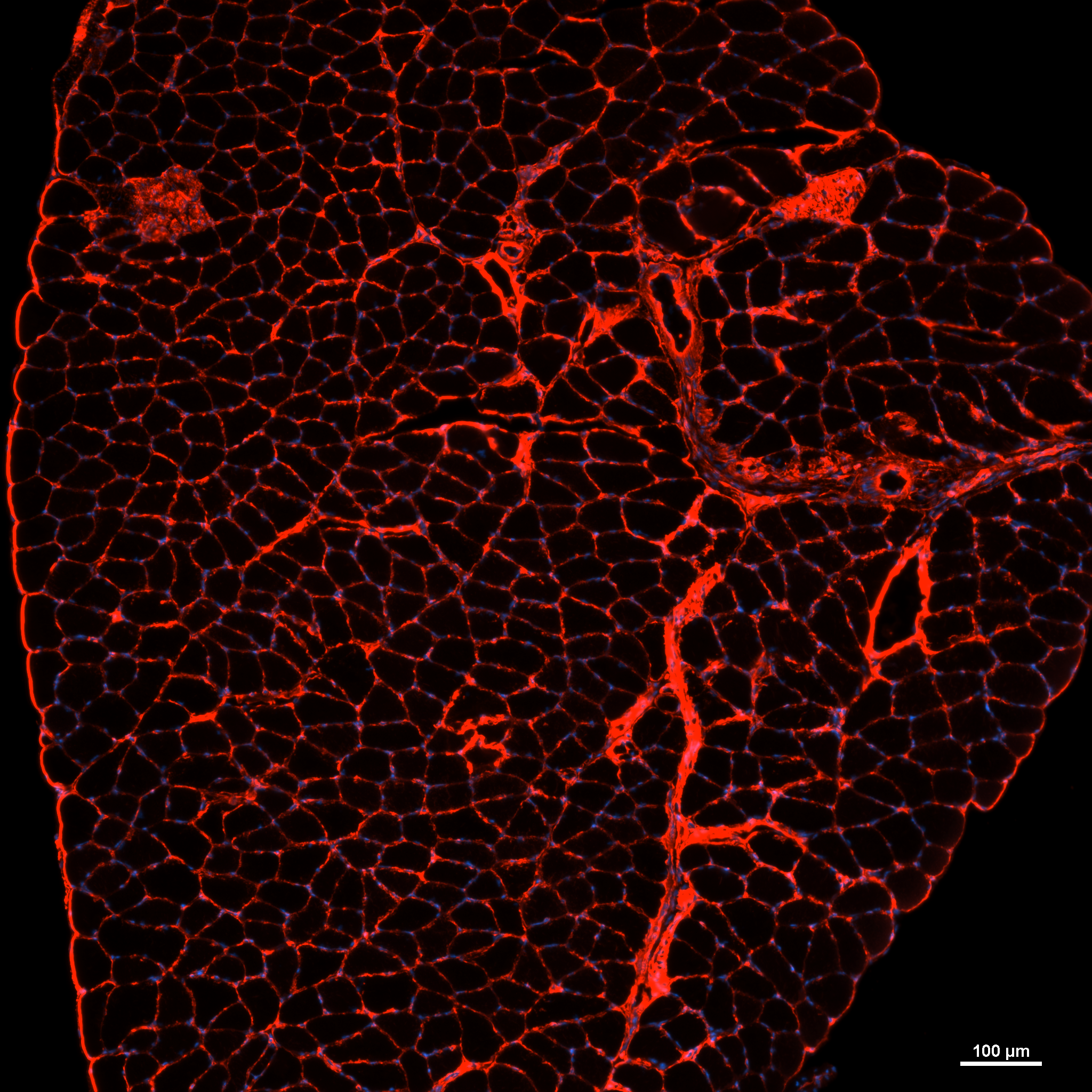

Supplement: Supplementary file 5 — Source data Fig. 2 [file 44321_2025_337_MOESM5_ESM.zip › Figure 2/Fig2K/Soleus muscle_Laminin-DAPI staining representative images/Xbp1_flfl_PBS.tif]

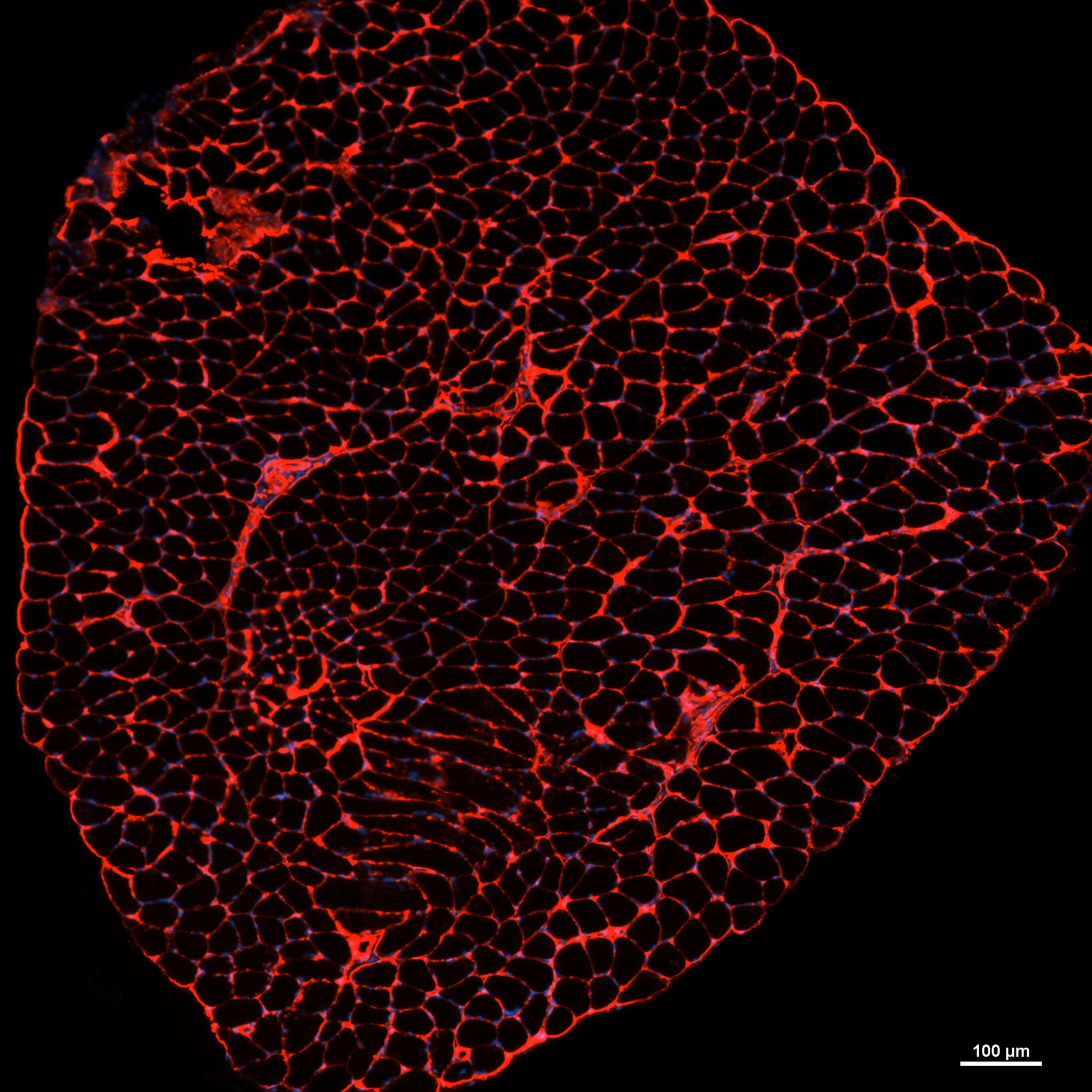

Supplement: Supplementary file 5 — Source data Fig. 2 [file 44321_2025_337_MOESM5_ESM.zip › Figure 2/Fig2K/Soleus muscle_Laminin-DAPI staining representative images/Xbp1_mKO_KPC.tif]

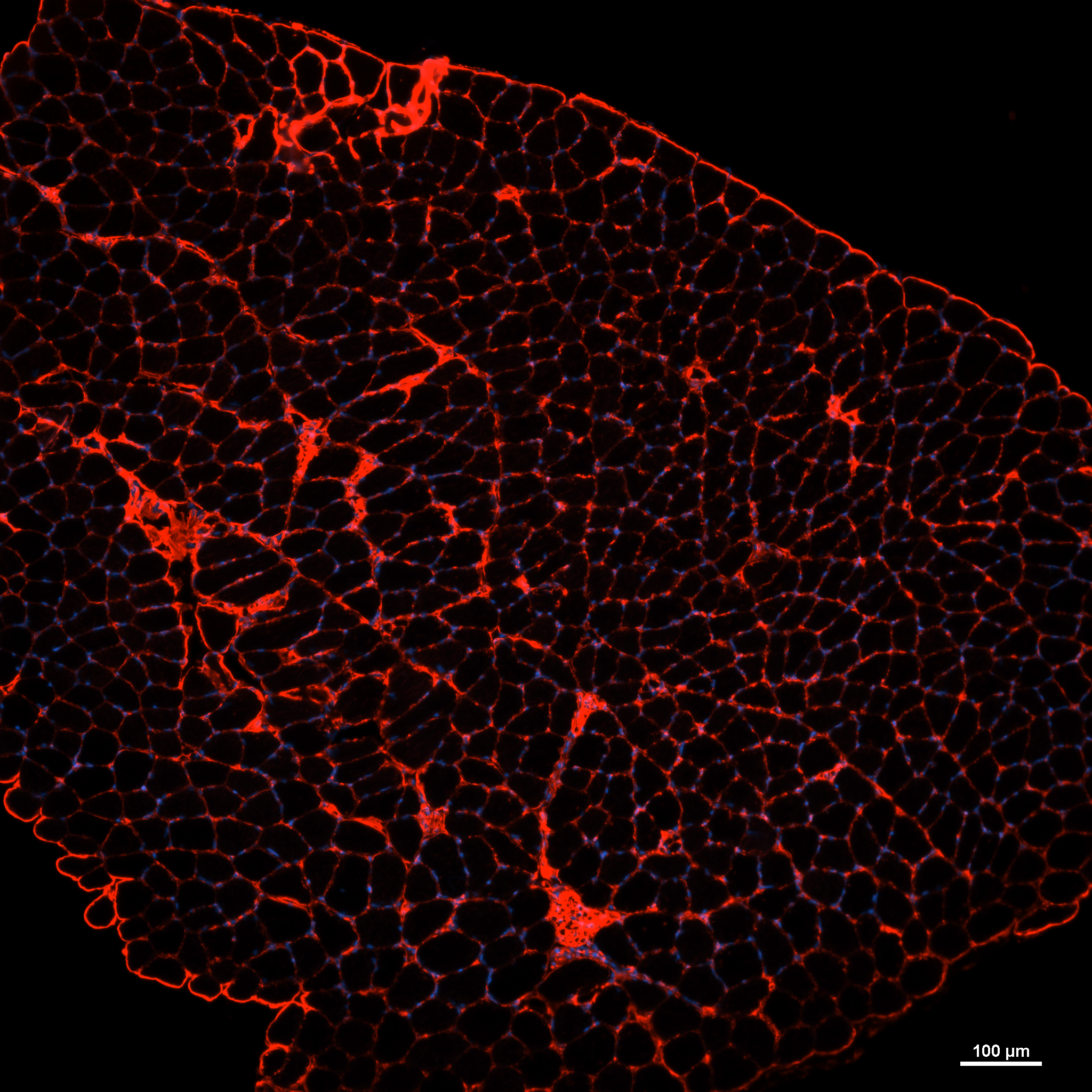

Supplement: Supplementary file 5 — Source data Fig. 2 [file 44321_2025_337_MOESM5_ESM.zip › Figure 2/Fig2K/Soleus muscle_Laminin-DAPI staining representative images/Xbp1_mKO_PBS.tif]

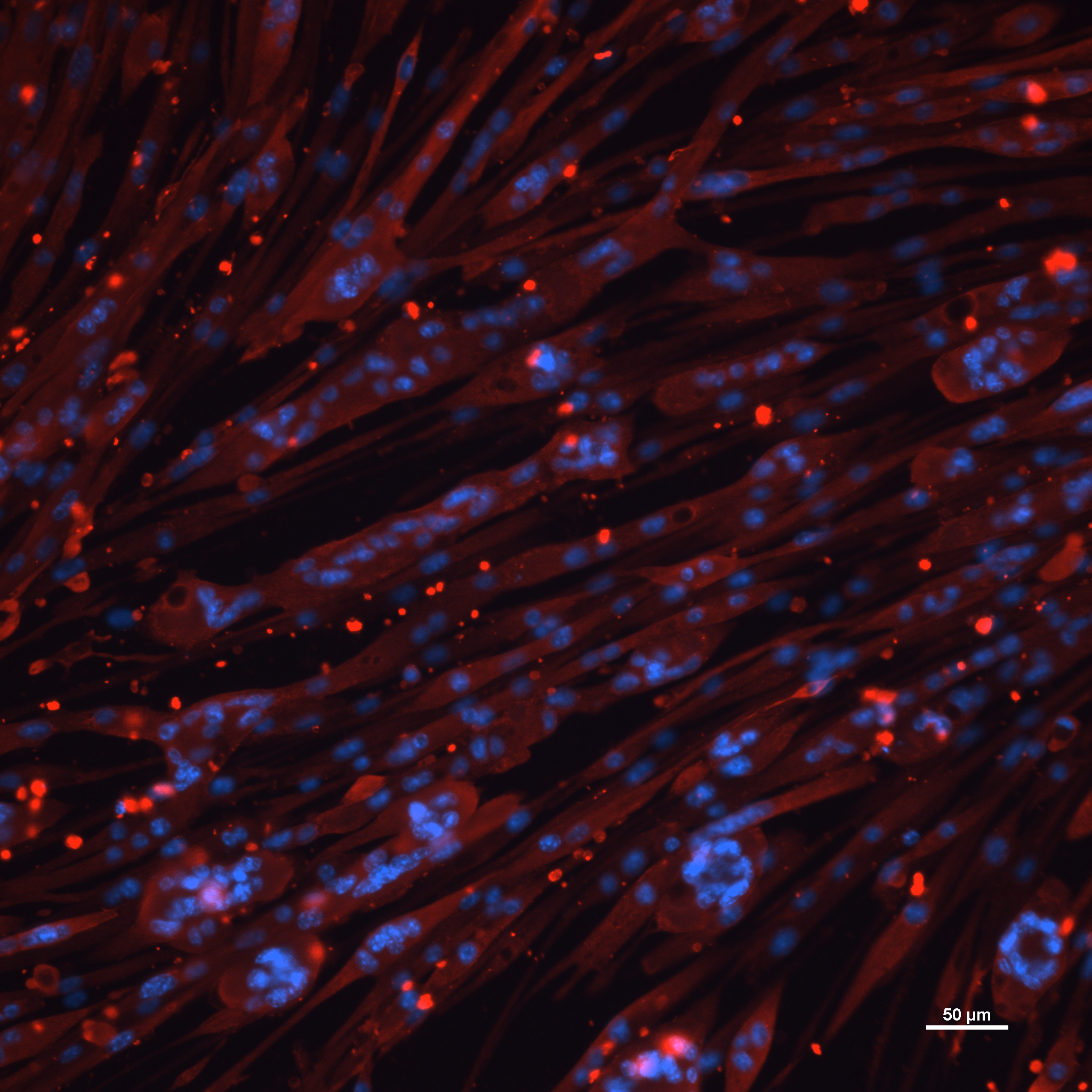

Supplement: Supplementary file 6 — Source data Fig. 3 [file 44321_2025_337_MOESM6_ESM.zip › Figure 3/Fig3A/Fig3A_Representative images_MyHC staining/Control siRNA-Control.tif]

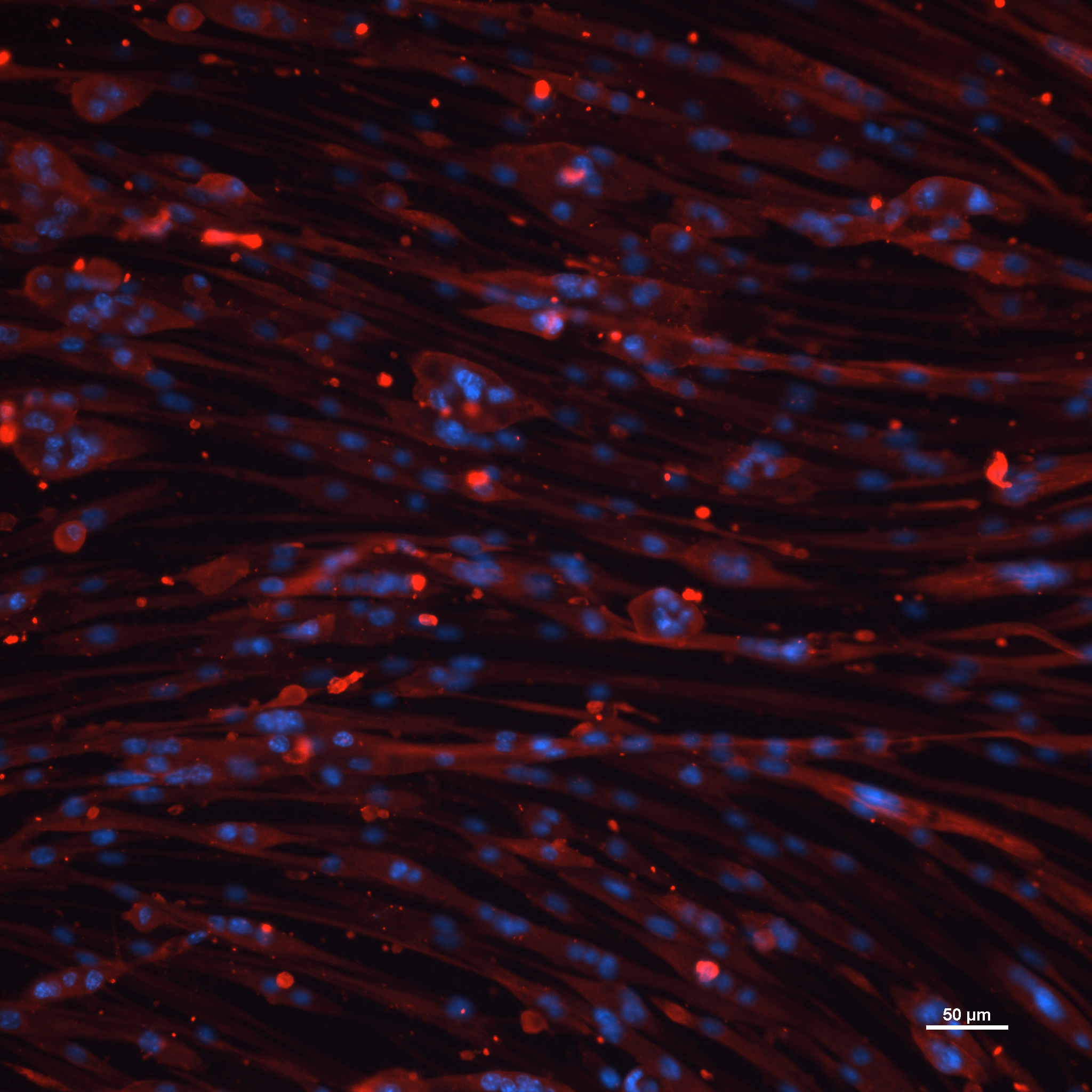

Supplement: Supplementary file 6 — Source data Fig. 3 [file 44321_2025_337_MOESM6_ESM.zip › Figure 3/Fig3A/Fig3A_Representative images_MyHC staining/Control siRNA-KPC_CM.tif]

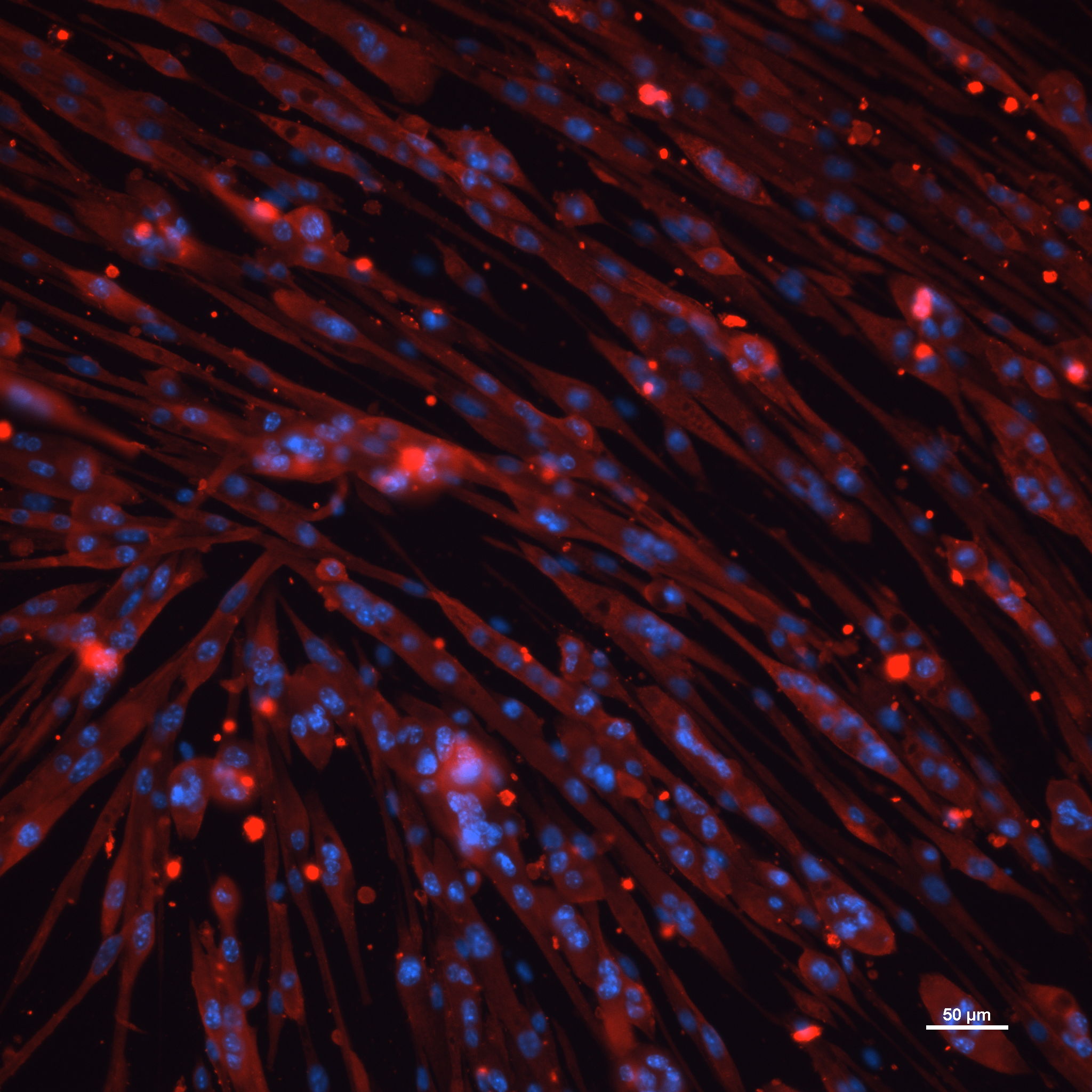

Supplement: Supplementary file 6 — Source data Fig. 3 [file 44321_2025_337_MOESM6_ESM.zip › Figure 3/Fig3A/Fig3A_Representative images_MyHC staining/XBP1 siRNA-Control.tif]

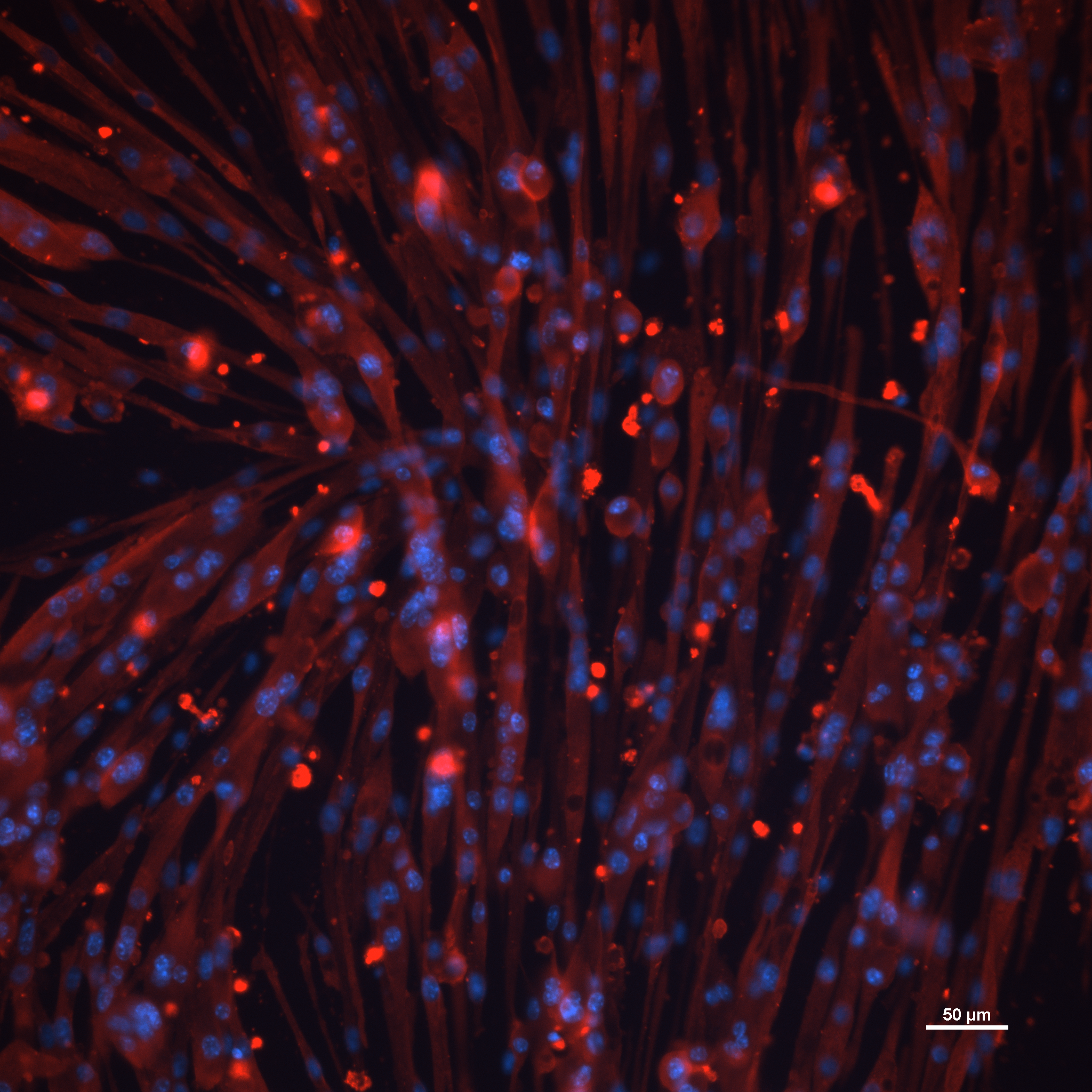

Supplement: Supplementary file 6 — Source data Fig. 3 [file 44321_2025_337_MOESM6_ESM.zip › Figure 3/Fig3A/Fig3A_Representative images_MyHC staining/XBP1 siRNA-KPC-CM.tif]

## Slide 1
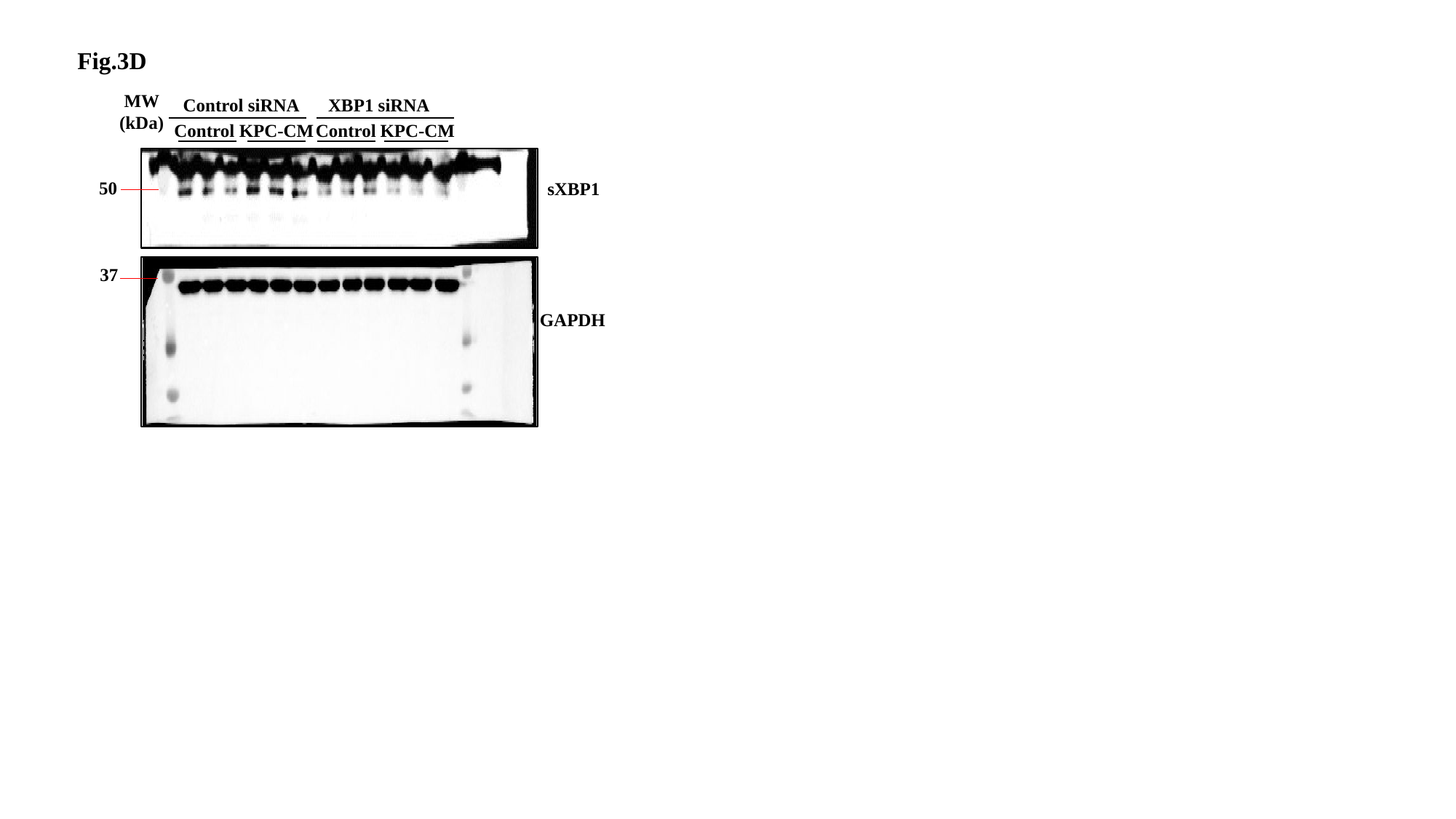

Fig.3D
MW (kDa)
Control siRNA
XBP1 siRNA
Control
KPC-CM
Control
KPC-CM
50
sXBP1
37
GAPDH

Supplement: Supplementary file 6 — Source data Fig. 3 [file 44321_2025_337_MOESM6_ESM.zip › Figure 3/Fig3D_J_Western blot/Fig3D_Western blot images/Fig3D_Western blot.pptx]

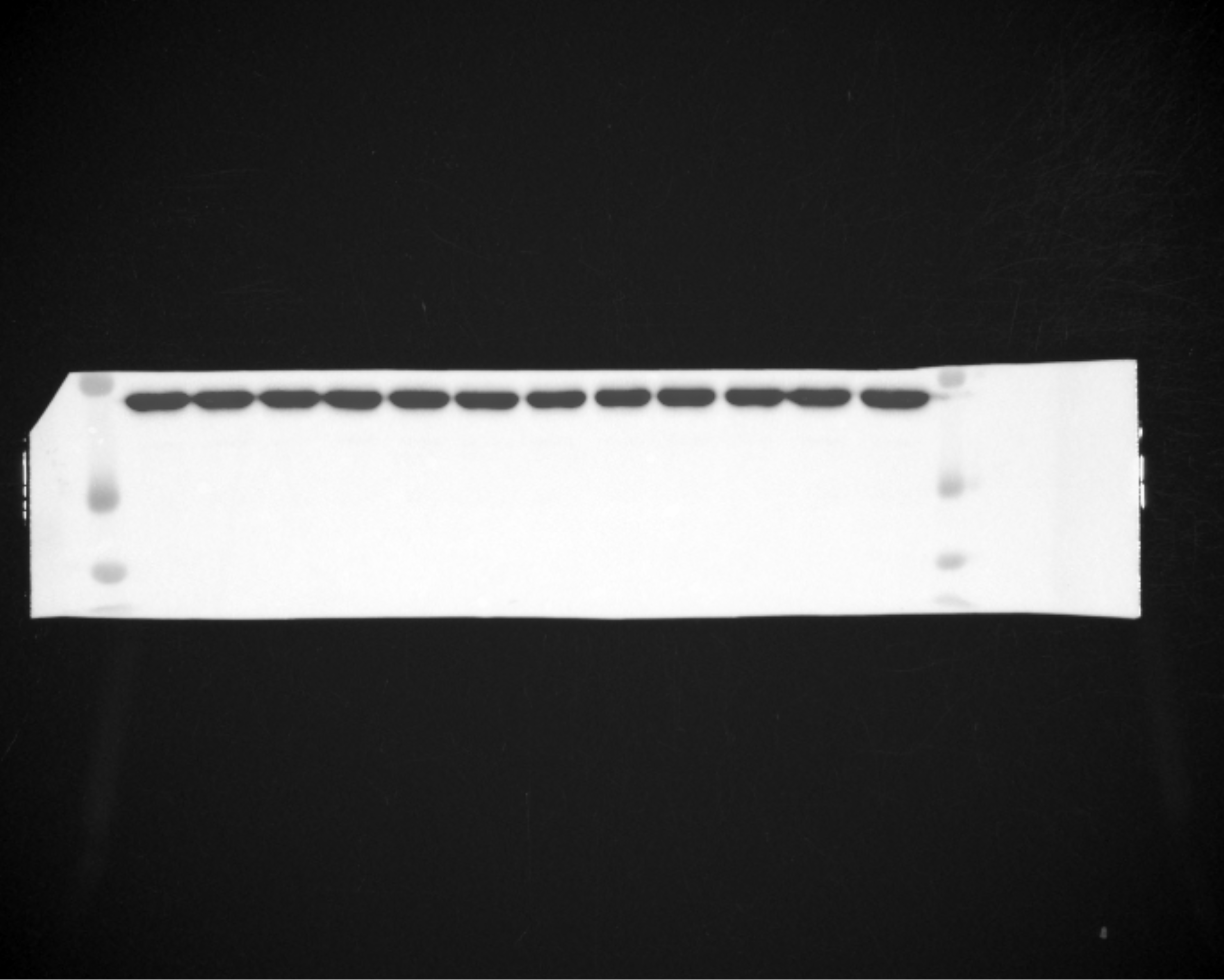

Supplement: Supplementary file 6 — Source data Fig. 3 [file 44321_2025_337_MOESM6_ESM.zip › Figure 3/Fig3D_J_Western blot/Fig3D_Western blot images/Western GAPDH.tif]

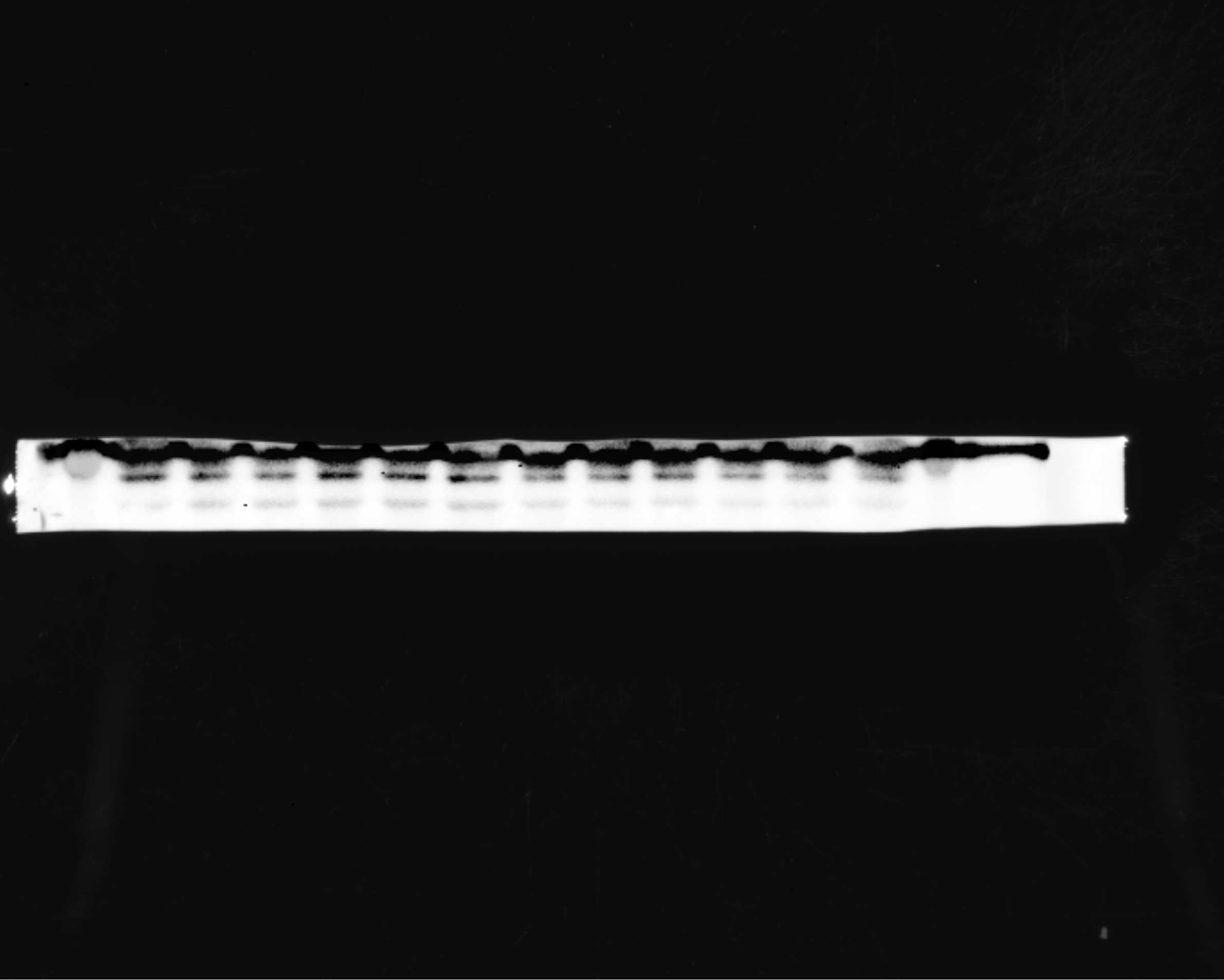

Supplement: Supplementary file 6 — Source data Fig. 3 [file 44321_2025_337_MOESM6_ESM.zip › Figure 3/Fig3D_J_Western blot/Fig3D_Western blot images/Western sXBP1.tif]

## Slide 1
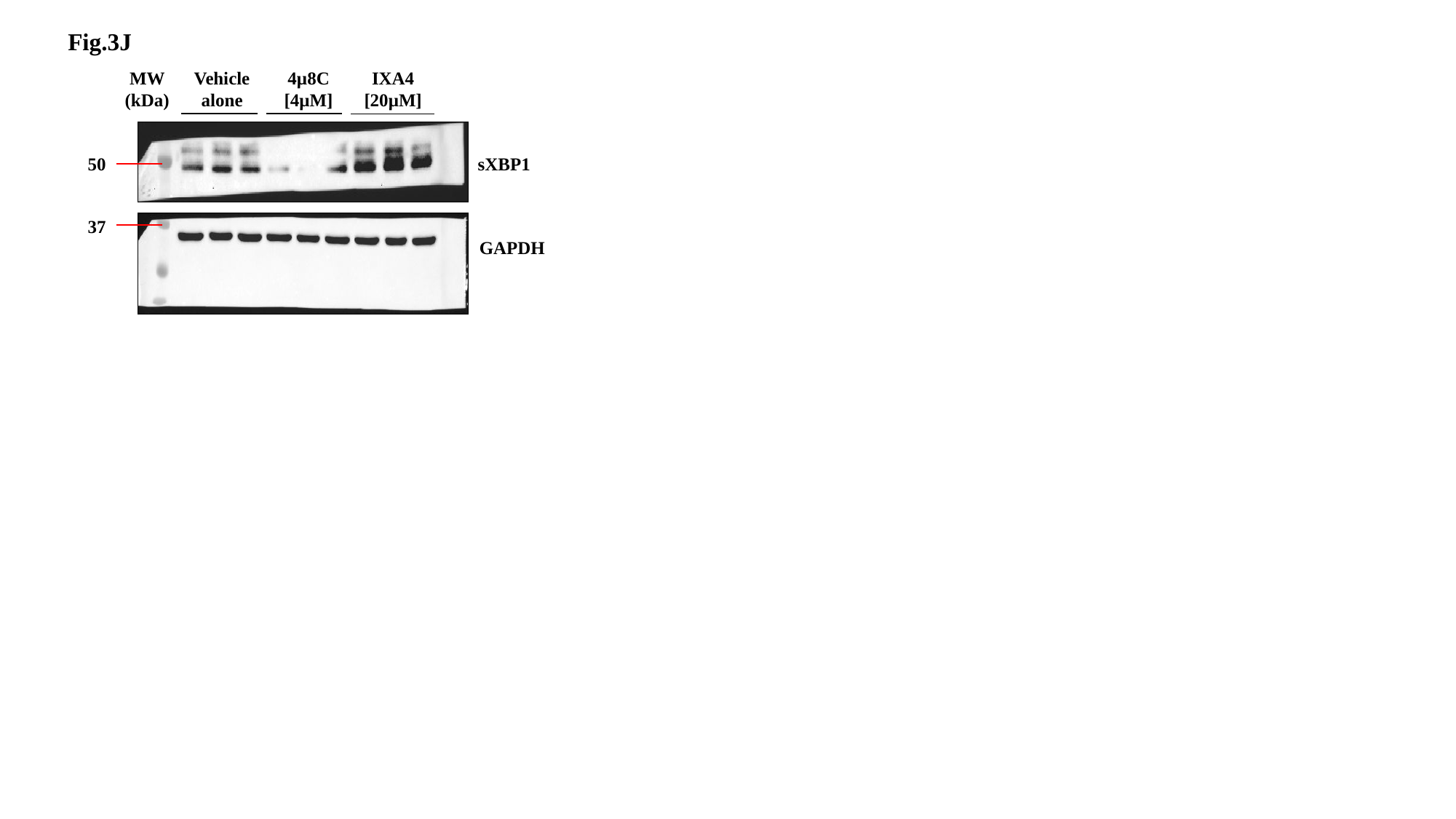

Fig.3J
MW (kDa)
Vehicle alone
4μ8C [4μM]
IXA4 [20μM]
50
sXBP1
37
GAPDH

Supplement: Supplementary file 6 — Source data Fig. 3 [file 44321_2025_337_MOESM6_ESM.zip › Figure 3/Fig3D_J_Western blot/Fig3J_Western blot images/Fig3J_Western blot.pptx]

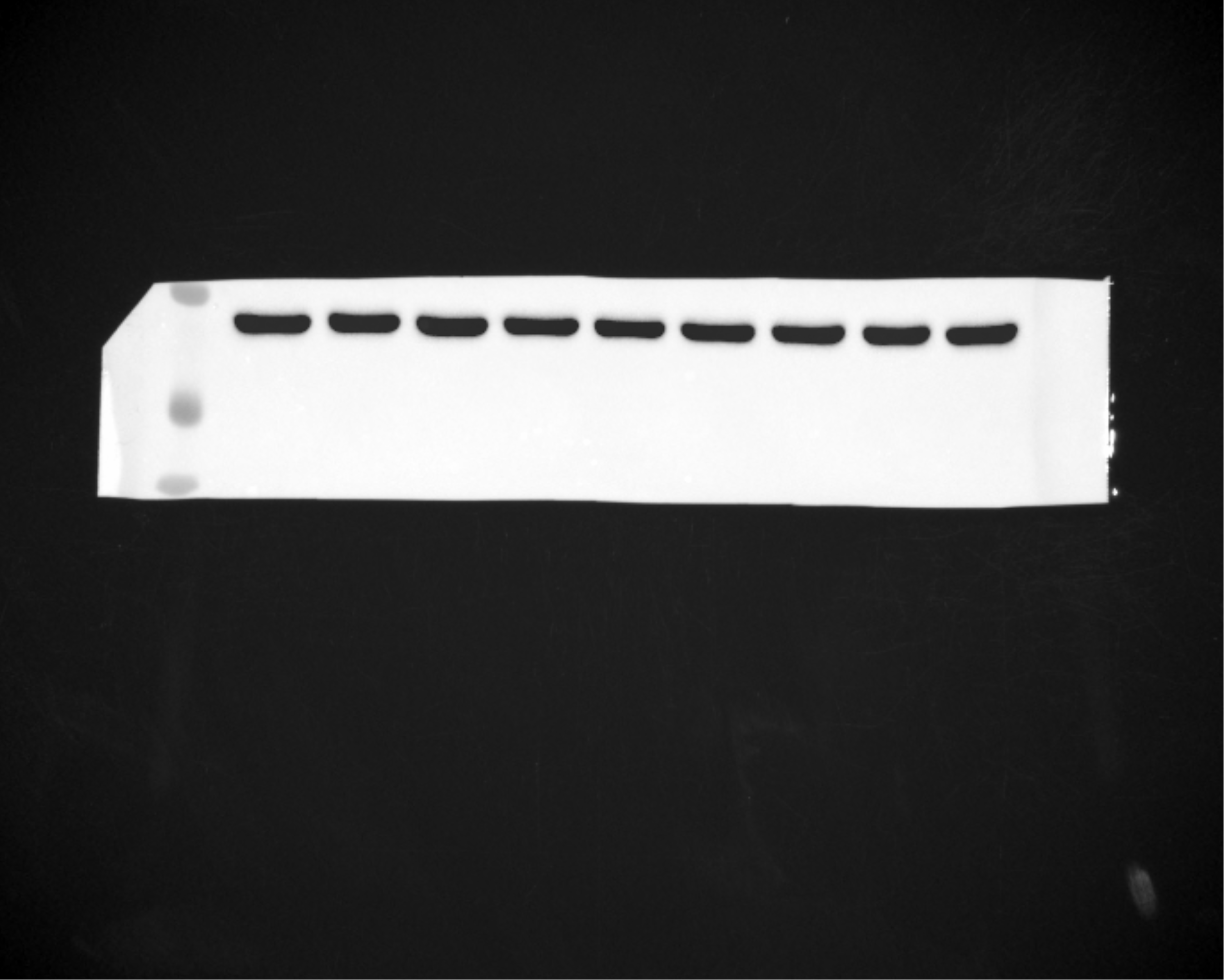

Supplement: Supplementary file 6 — Source data Fig. 3 [file 44321_2025_337_MOESM6_ESM.zip › Figure 3/Fig3D_J_Western blot/Fig3J_Western blot images/Western GAPDH.tif]

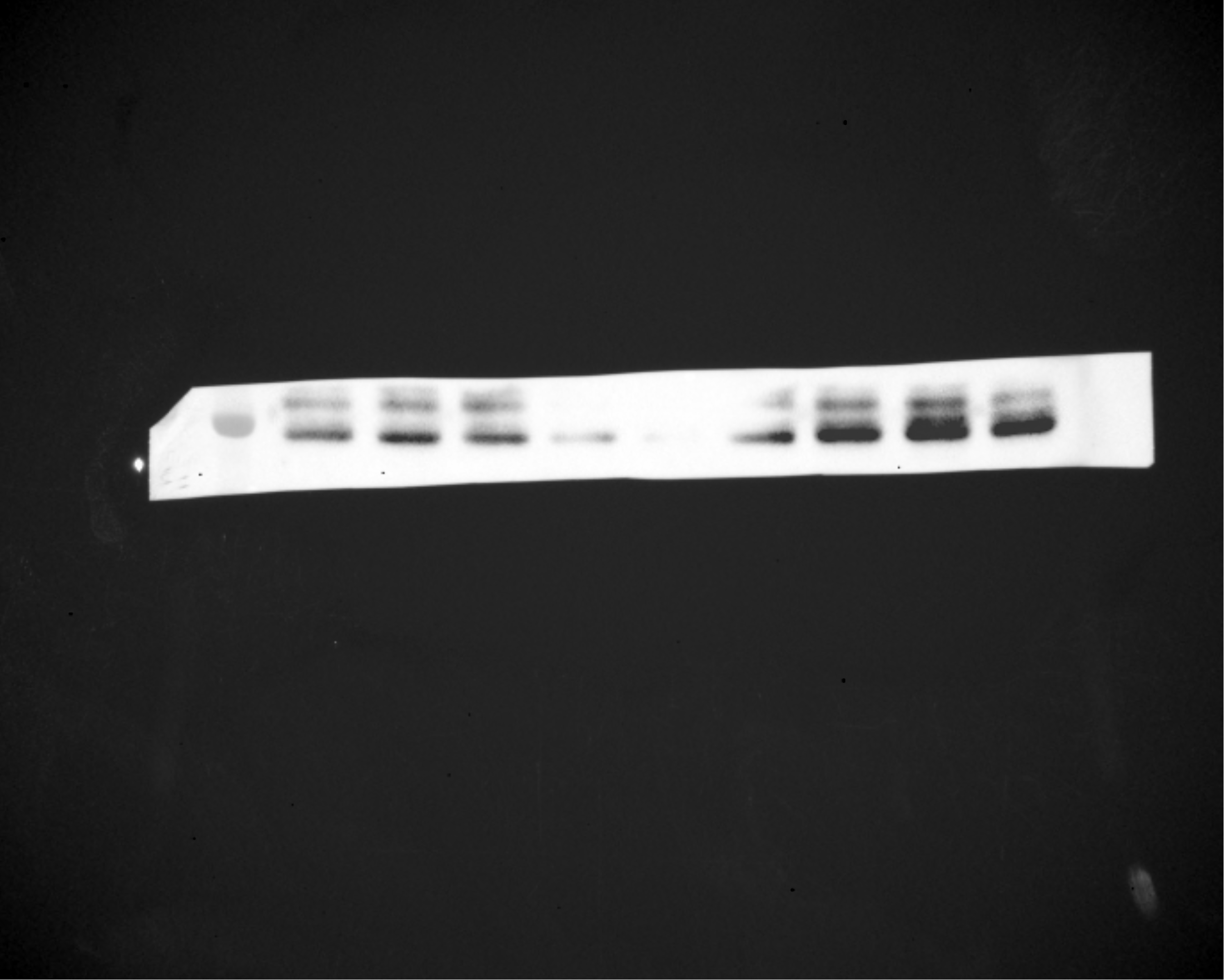

Supplement: Supplementary file 6 — Source data Fig. 3 [file 44321_2025_337_MOESM6_ESM.zip › Figure 3/Fig3D_J_Western blot/Fig3J_Western blot images/Western sXBP1.tif]

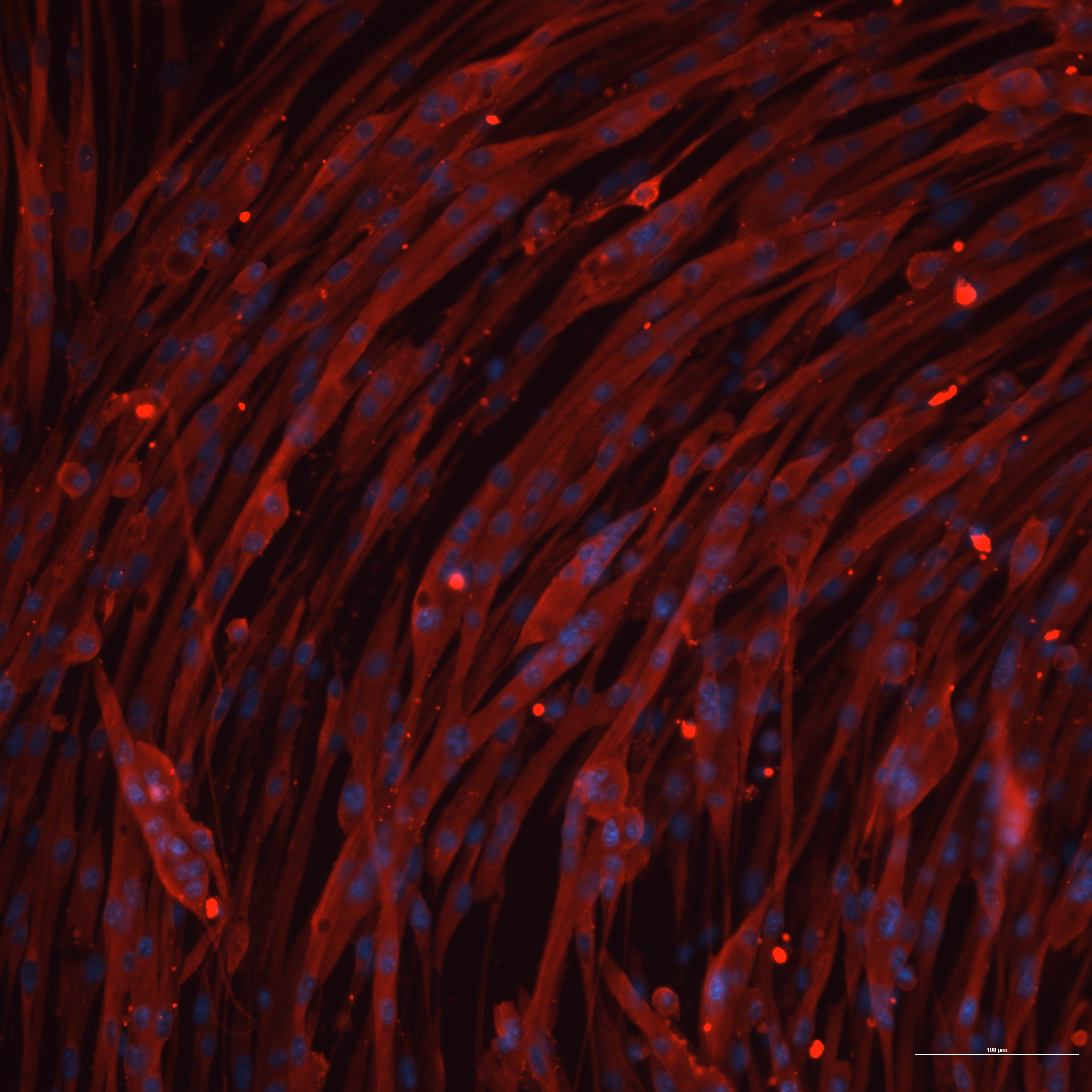

Supplement: Supplementary file 6 — Source data Fig. 3 [file 44321_2025_337_MOESM6_ESM.zip › Figure 3/Fig3F/Fig3F_Representative images_MyHC staining/4u8C-Control.tif]

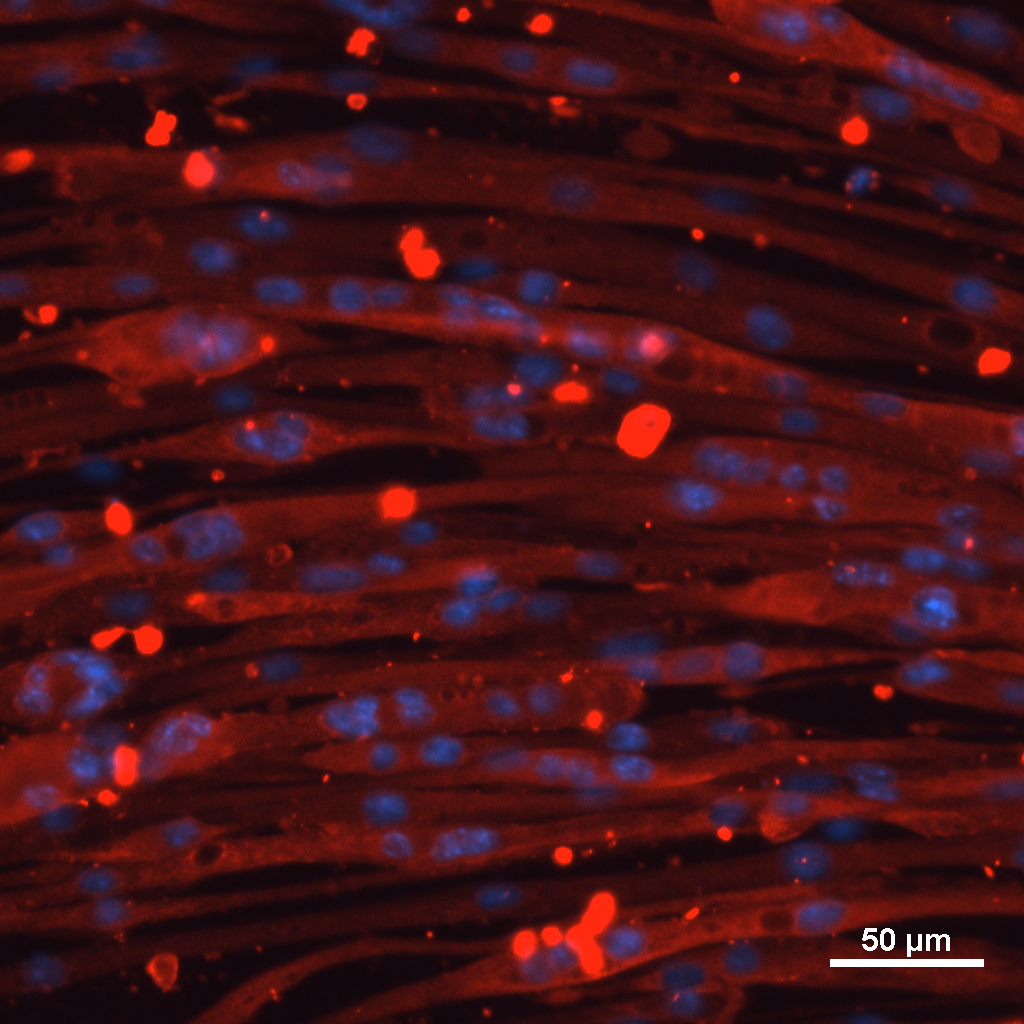

Supplement: Supplementary file 6 — Source data Fig. 3 [file 44321_2025_337_MOESM6_ESM.zip › Figure 3/Fig3F/Fig3F_Representative images_MyHC staining/4u8C-KPC-CM.tif]

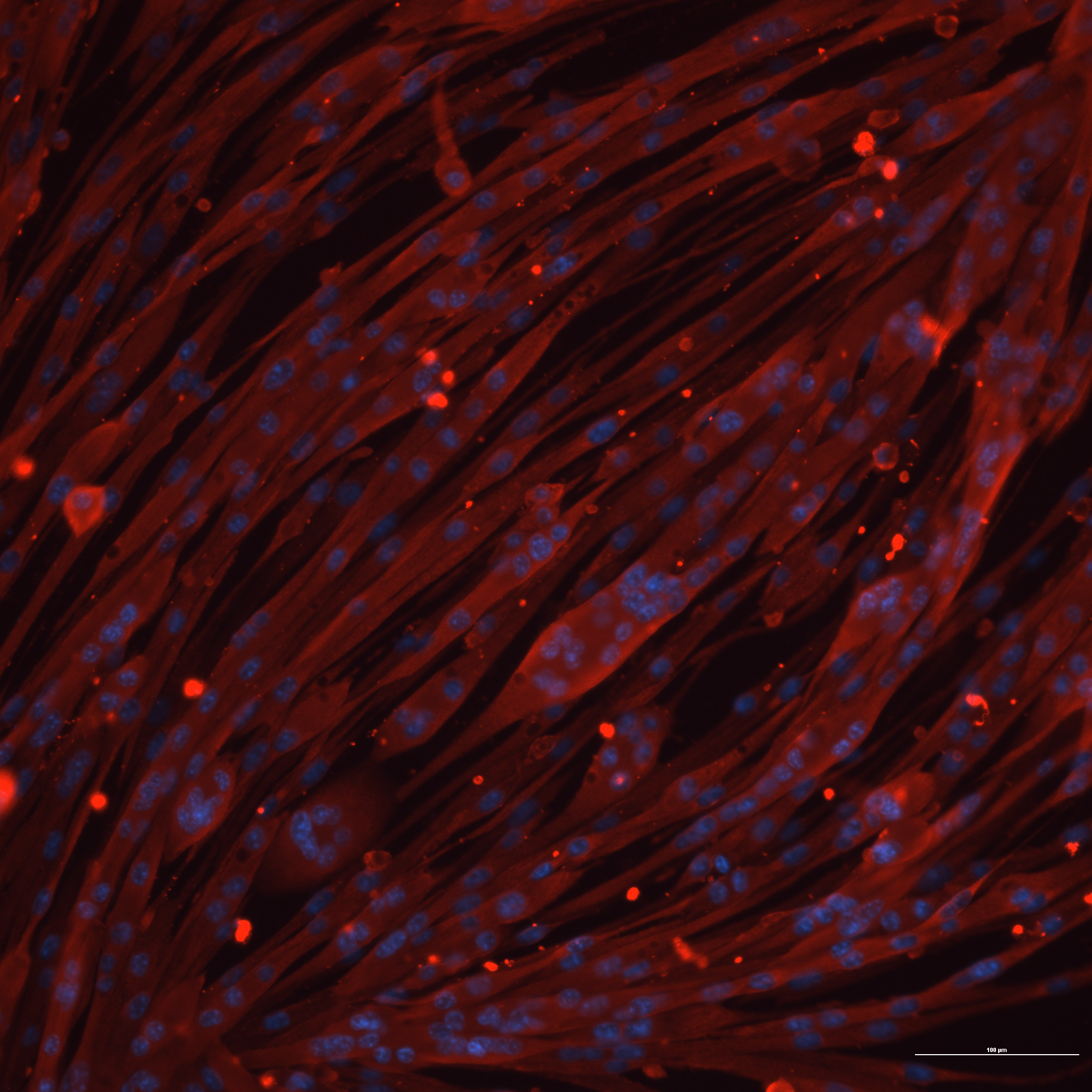

Supplement: Supplementary file 6 — Source data Fig. 3 [file 44321_2025_337_MOESM6_ESM.zip › Figure 3/Fig3F/Fig3F_Representative images_MyHC staining/Vehicle-Control.tif]

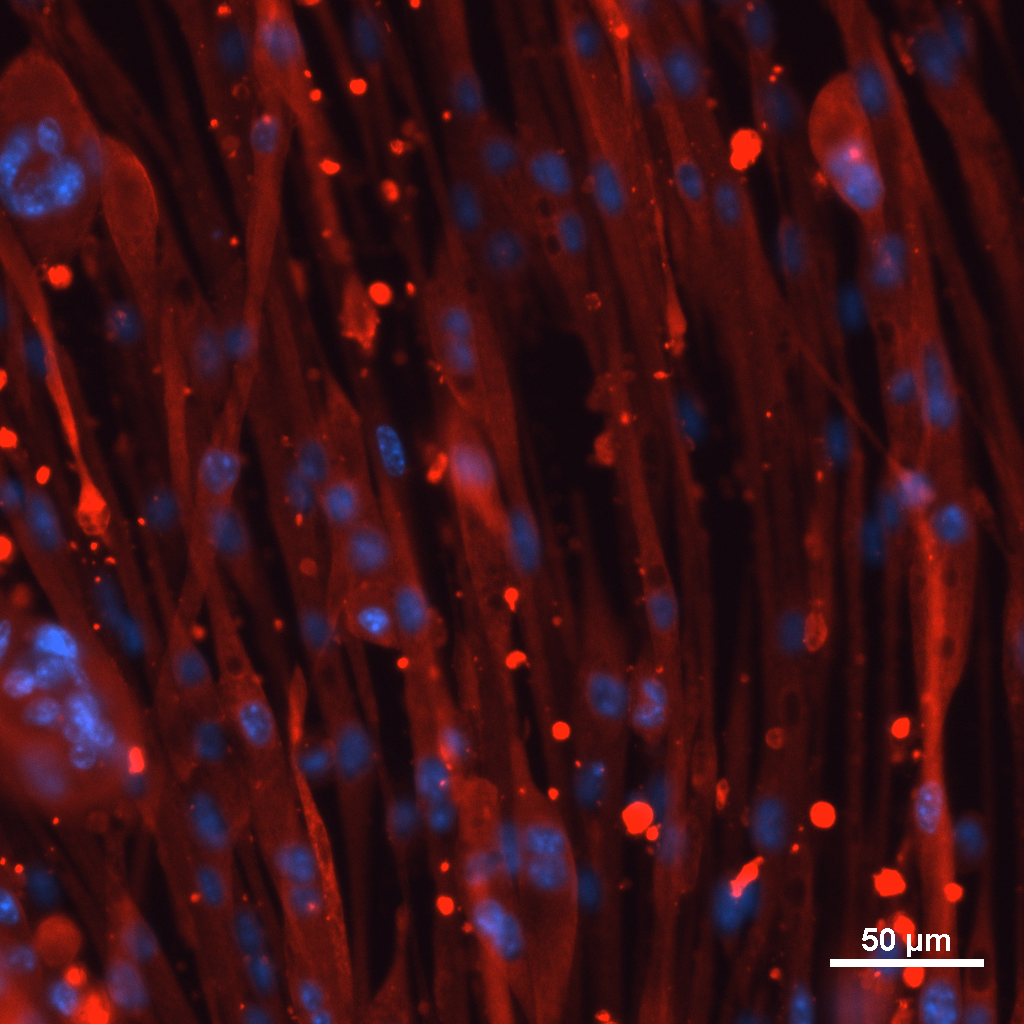

Supplement: Supplementary file 6 — Source data Fig. 3 [file 44321_2025_337_MOESM6_ESM.zip › Figure 3/Fig3F/Fig3F_Representative images_MyHC staining/Vehicle-KPC-CM.tif]

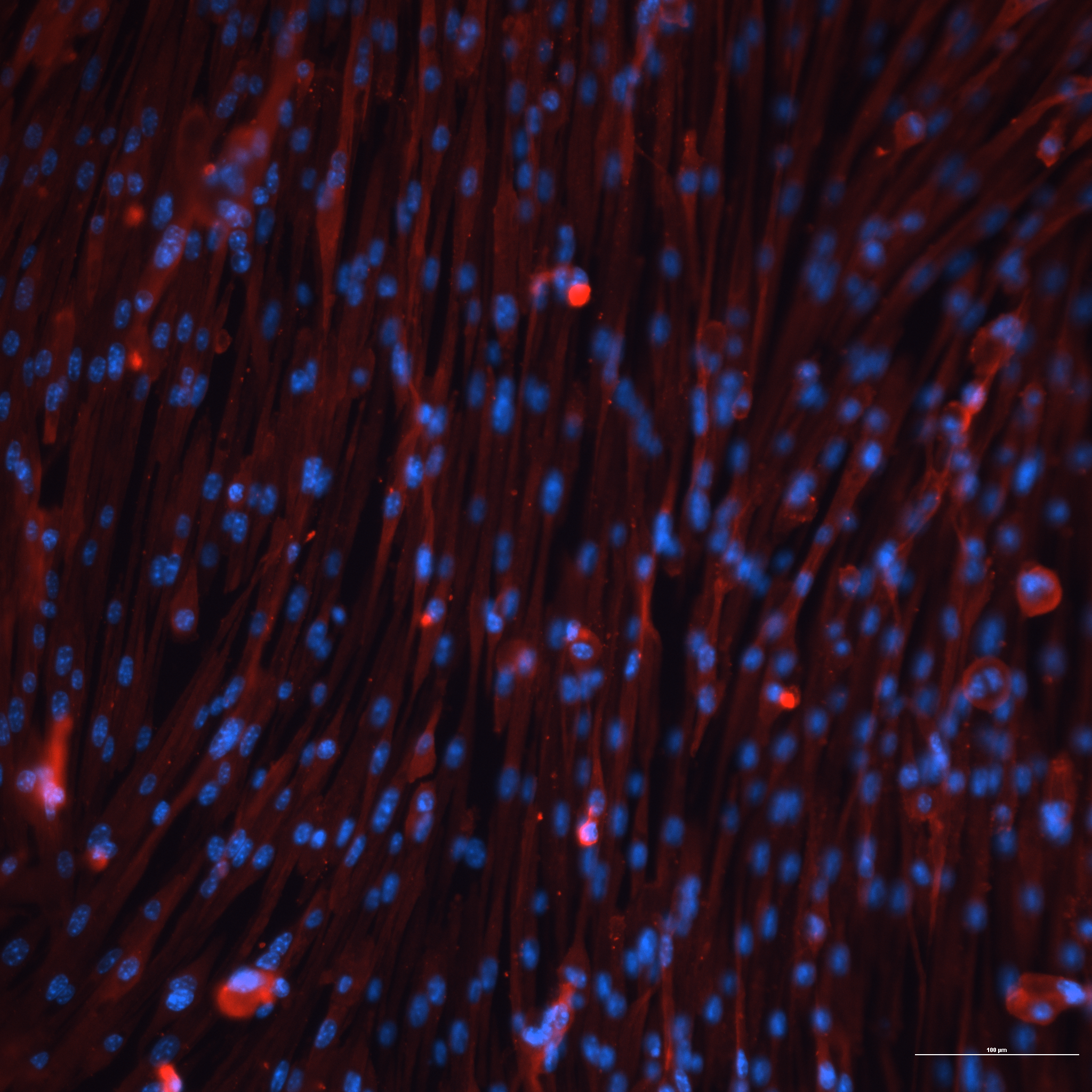

Supplement: Supplementary file 6 — Source data Fig. 3 [file 44321_2025_337_MOESM6_ESM.zip › Figure 3/Fig3H/Fig3H_Representative images_MyHC staining/IXA4-Control.tif]

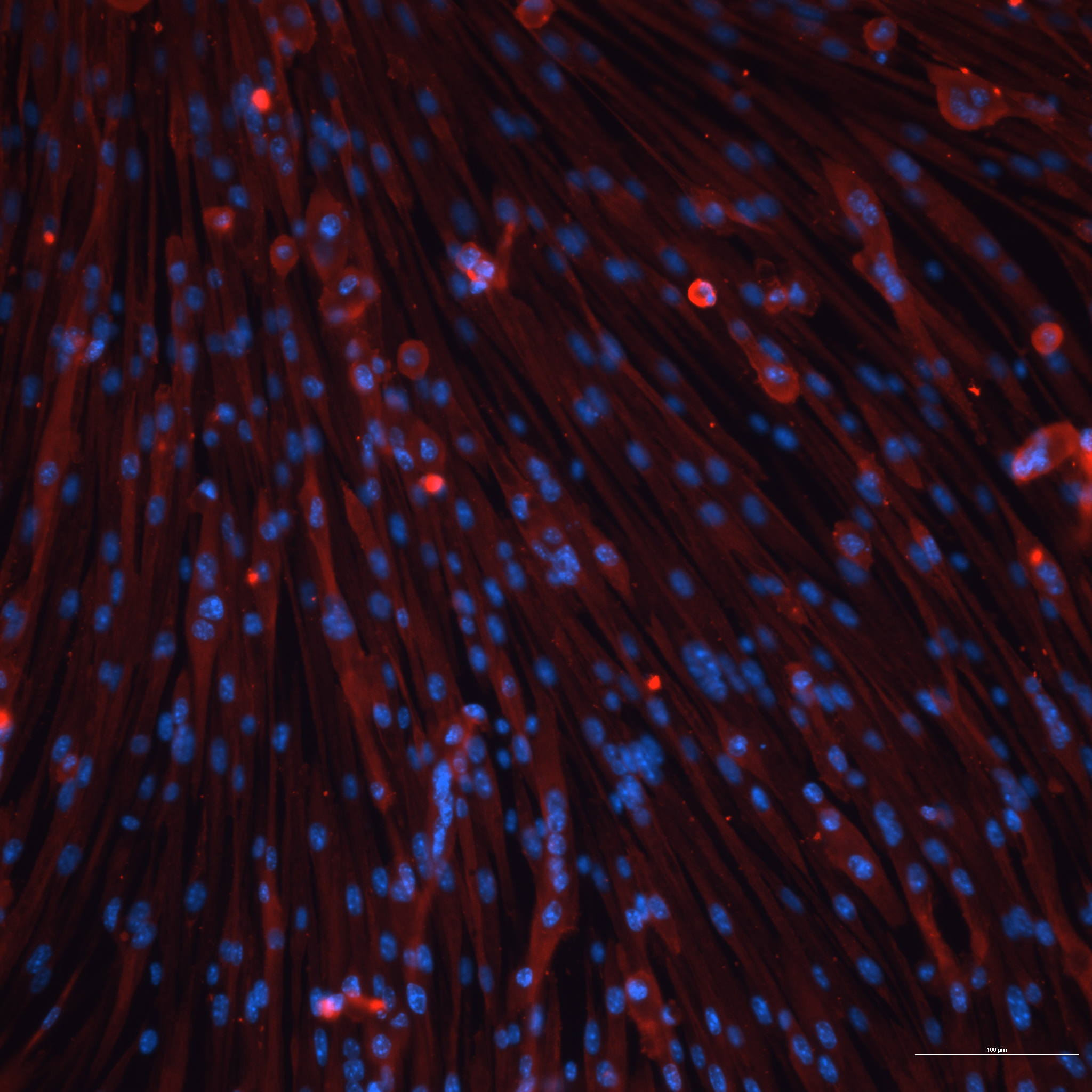

Supplement: Supplementary file 6 — Source data Fig. 3 [file 44321_2025_337_MOESM6_ESM.zip › Figure 3/Fig3H/Fig3H_Representative images_MyHC staining/IXA4-KPC-CM.tif]

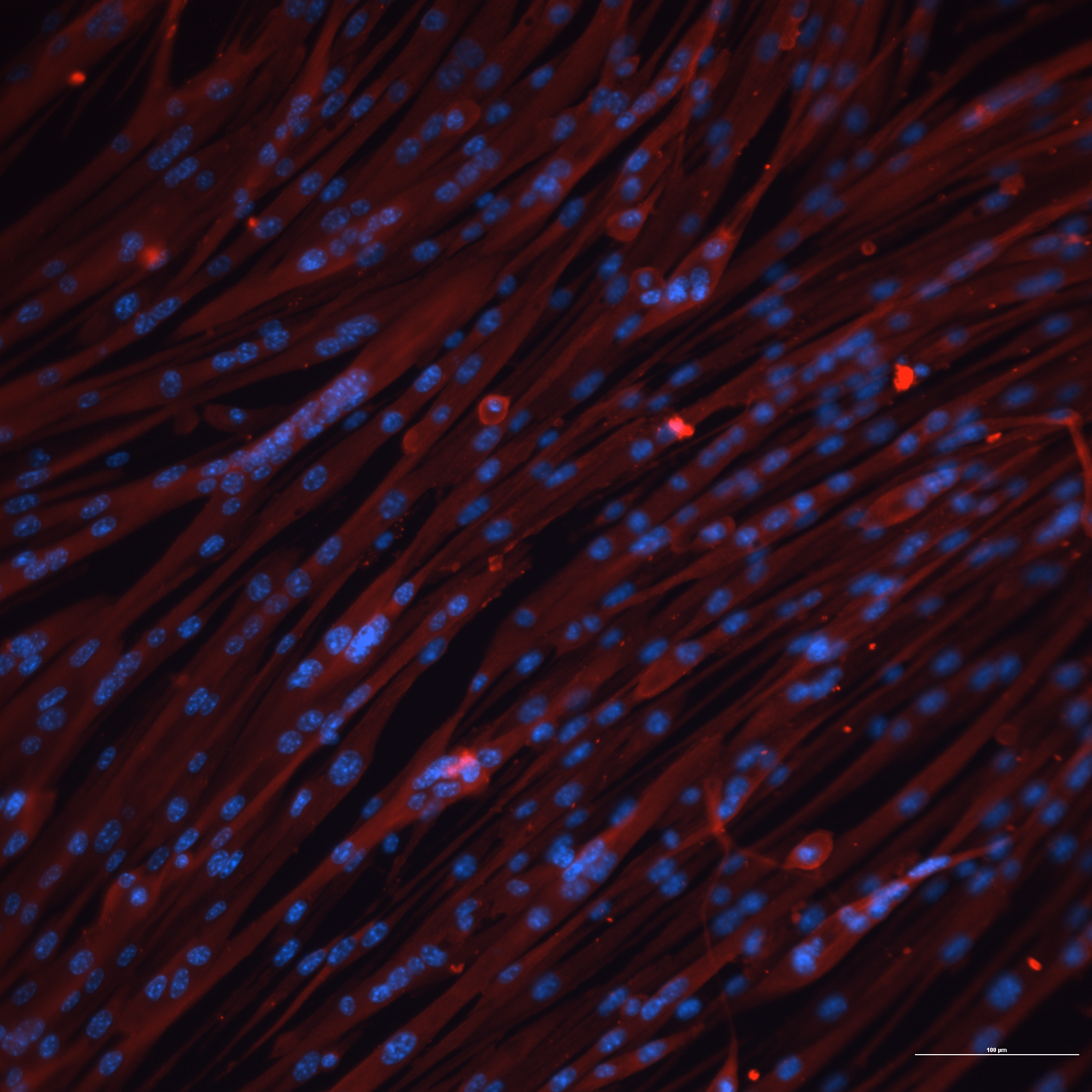

Supplement: Supplementary file 6 — Source data Fig. 3 [file 44321_2025_337_MOESM6_ESM.zip › Figure 3/Fig3H/Fig3H_Representative images_MyHC staining/Vehicle-Control.tif]

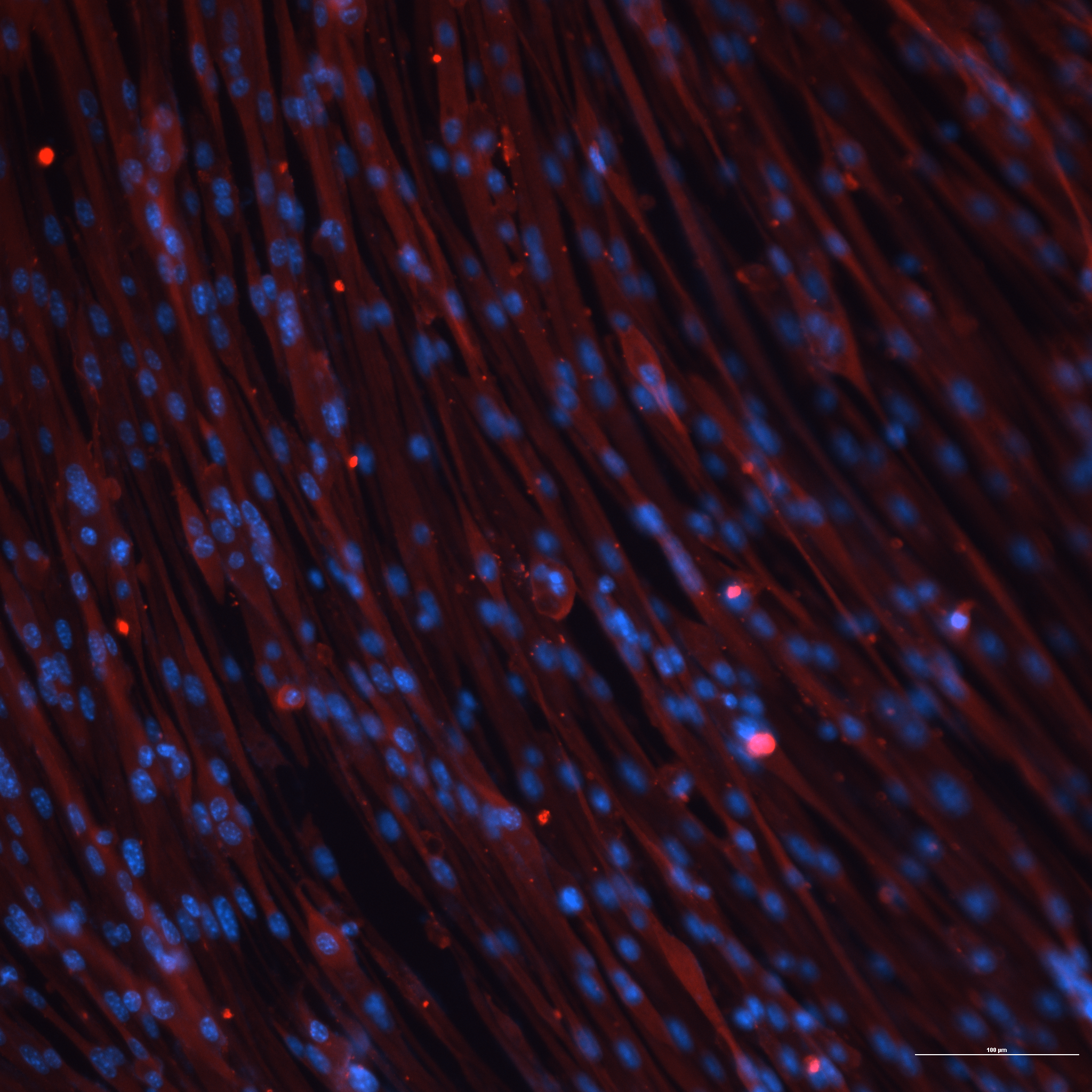

Supplement: Supplementary file 6 — Source data Fig. 3 [file 44321_2025_337_MOESM6_ESM.zip › Figure 3/Fig3H/Fig3H_Representative images_MyHC staining/Vehicle-KPC-CM.tif]

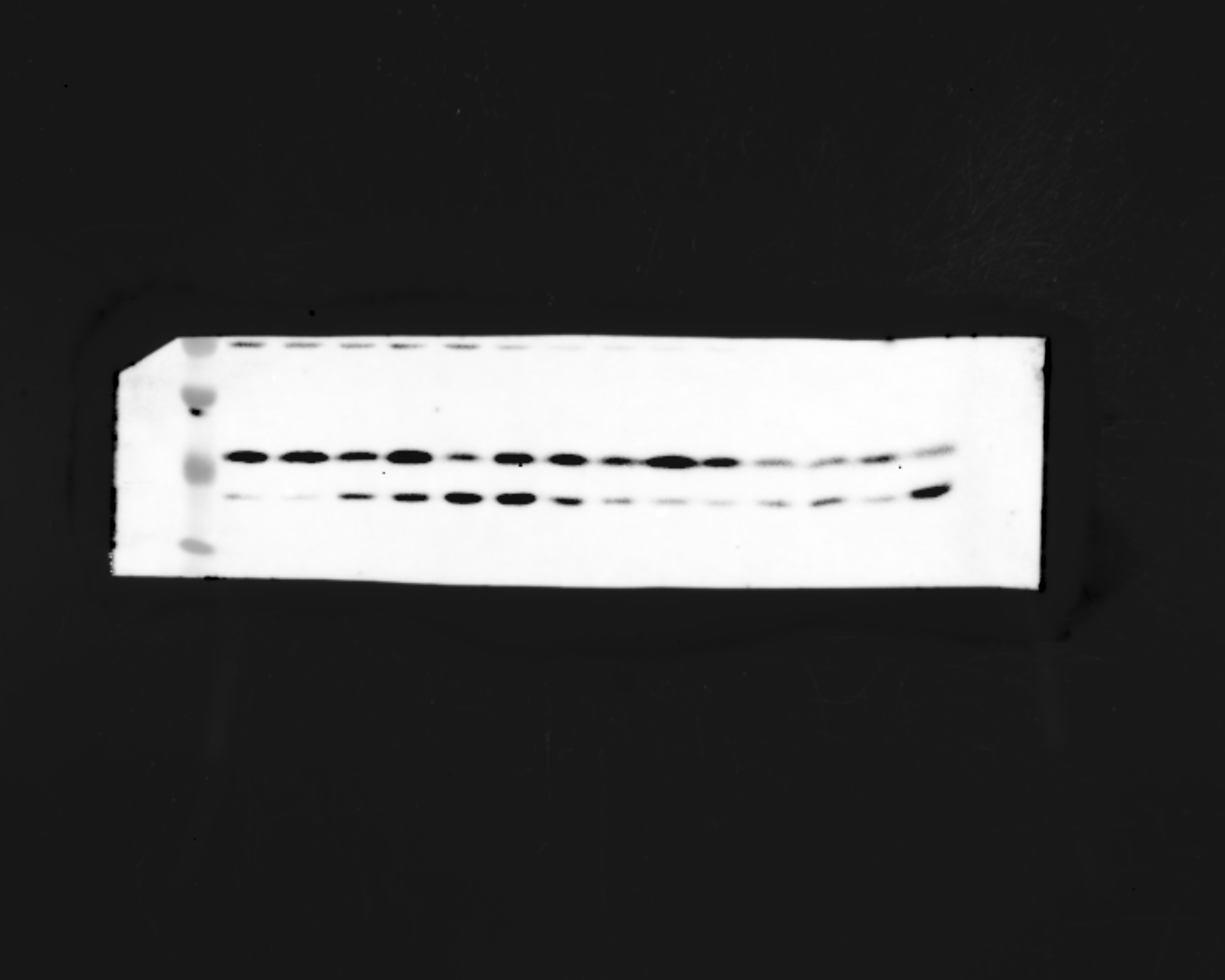

Supplement: Supplementary file 7 — Source data Fig. 4 [file 44321_2025_337_MOESM7_ESM.zip › Figure 4/Fig4C_E_Western blot images/Fig4C_Western blot images/Western LC3B.tif]

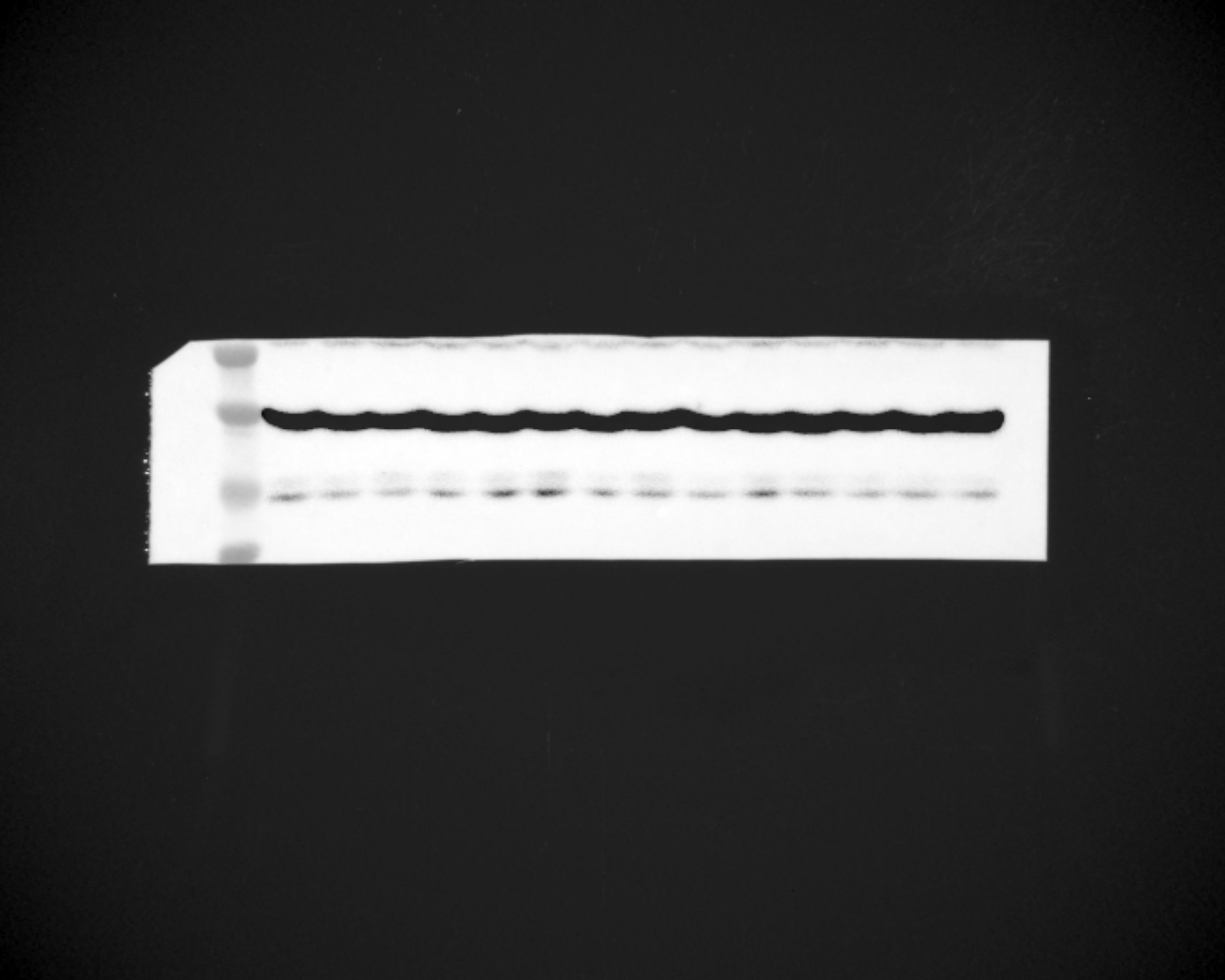

Supplement: Supplementary file 7 — Source data Fig. 4 [file 44321_2025_337_MOESM7_ESM.zip › Figure 4/Fig4C_E_Western blot images/Fig4C_Western blot images/Western MAFbx.tif]

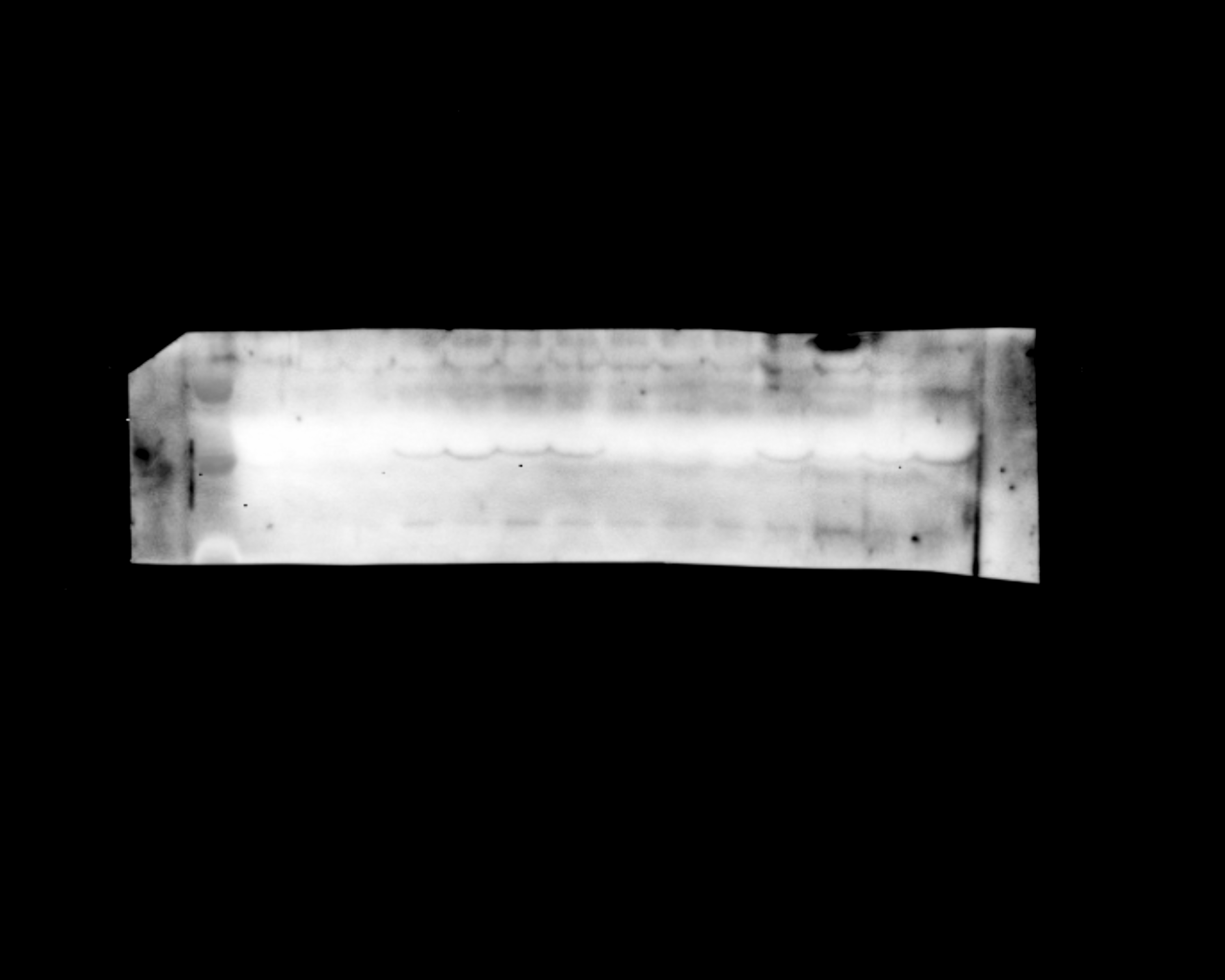

Supplement: Supplementary file 7 — Source data Fig. 4 [file 44321_2025_337_MOESM7_ESM.zip › Figure 4/Fig4C_E_Western blot images/Fig4C_Western blot images/Western MuRF1.tif]

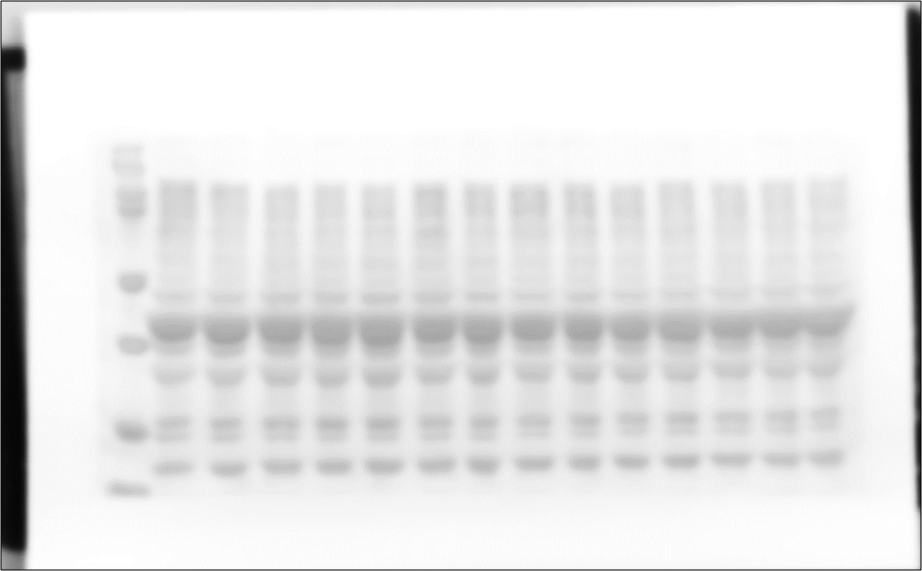

Supplement: Supplementary file 7 — Source data Fig. 4 [file 44321_2025_337_MOESM7_ESM.zip › Figure 4/Fig4C_E_Western blot images/Fig4C_Western blot images/Western Ponceau Stain.tif]

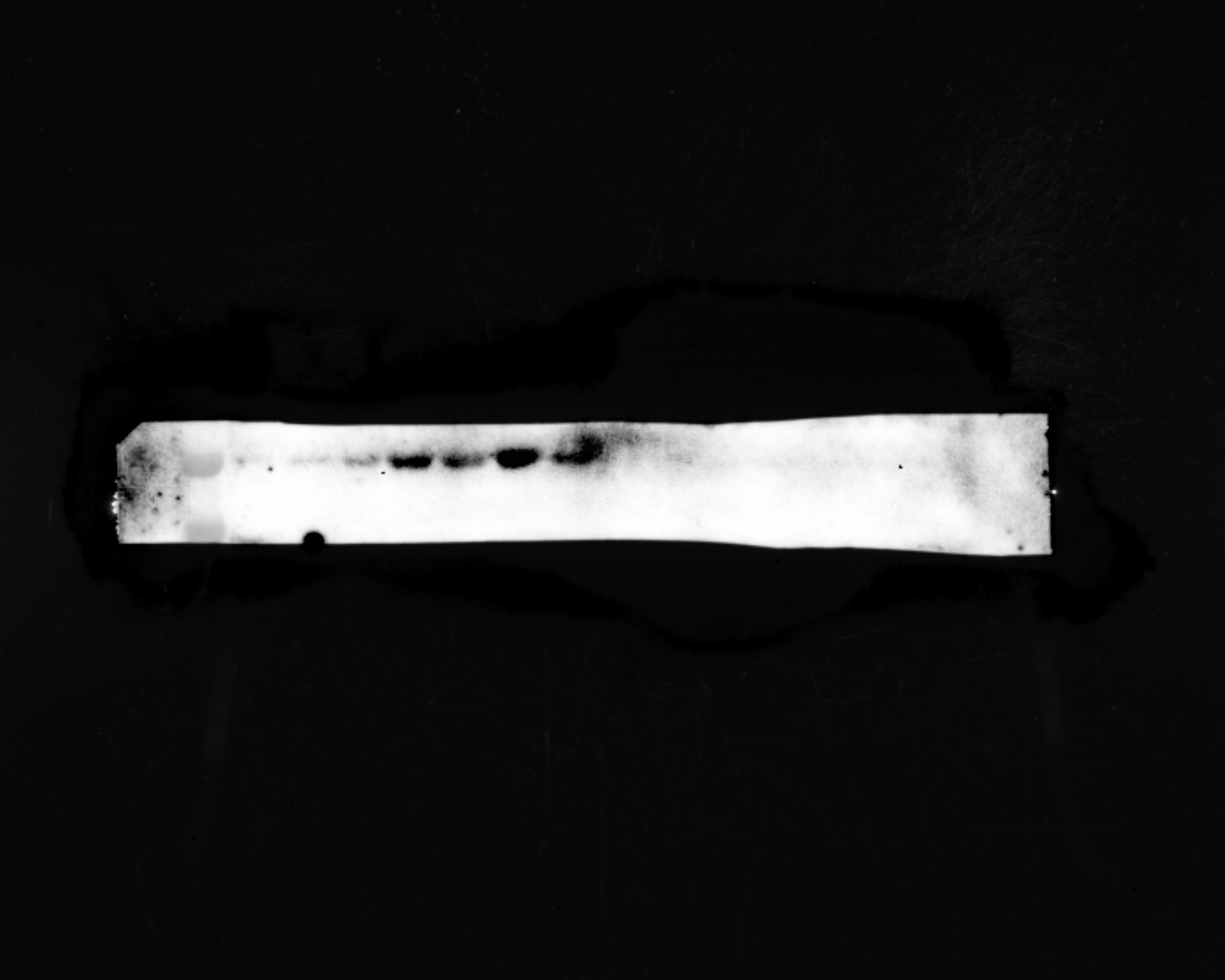

Supplement: Supplementary file 7 — Source data Fig. 4 [file 44321_2025_337_MOESM7_ESM.zip › Figure 4/Fig4C_E_Western blot images/Fig4C_Western blot images/Western sXBP1.tif]

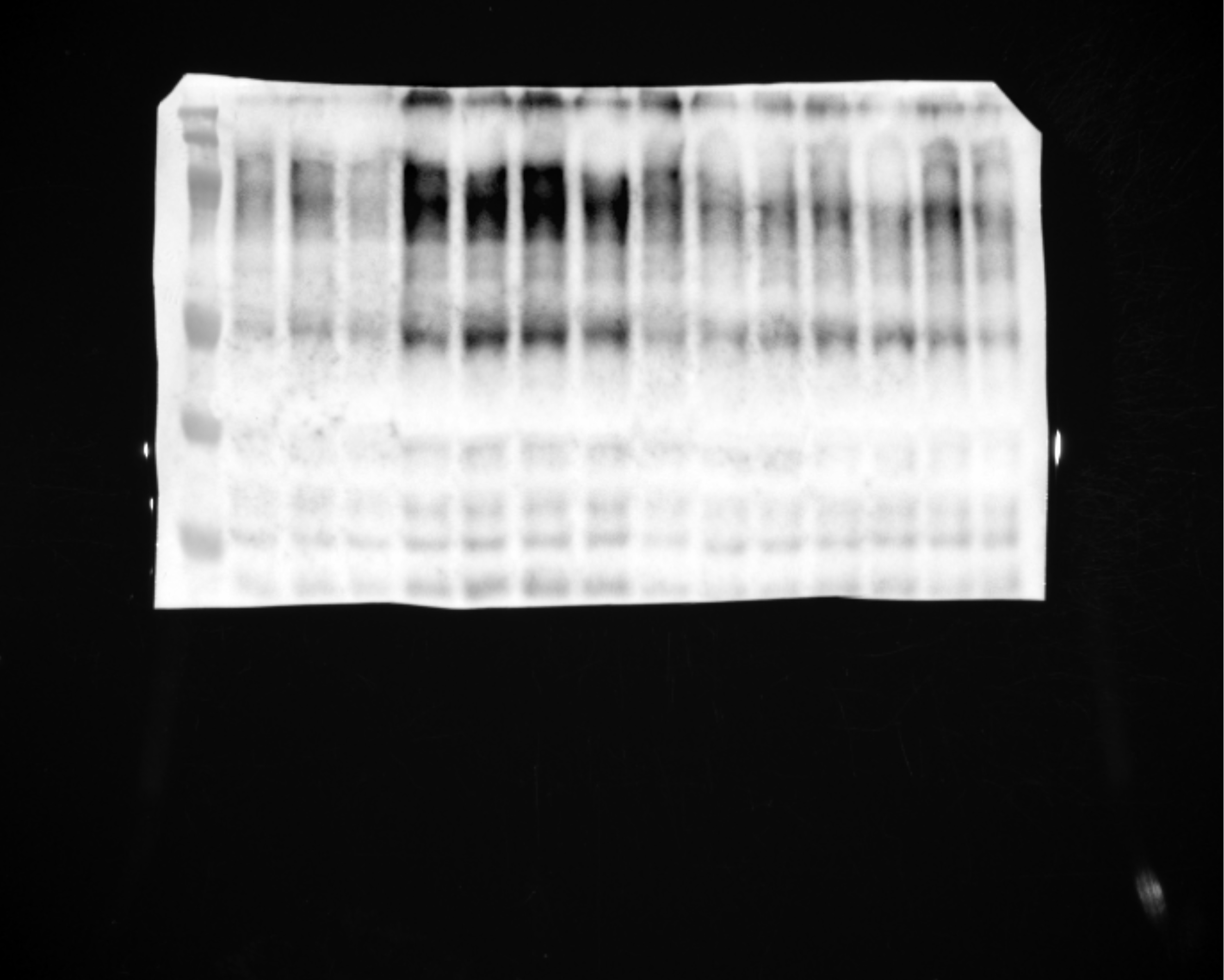

Supplement: Supplementary file 7 — Source data Fig. 4 [file 44321_2025_337_MOESM7_ESM.zip › Figure 4/Fig4C_E_Western blot images/Fig4C_Western blot images/Western Ub-conjugated proteins.tif]

## Slide 1
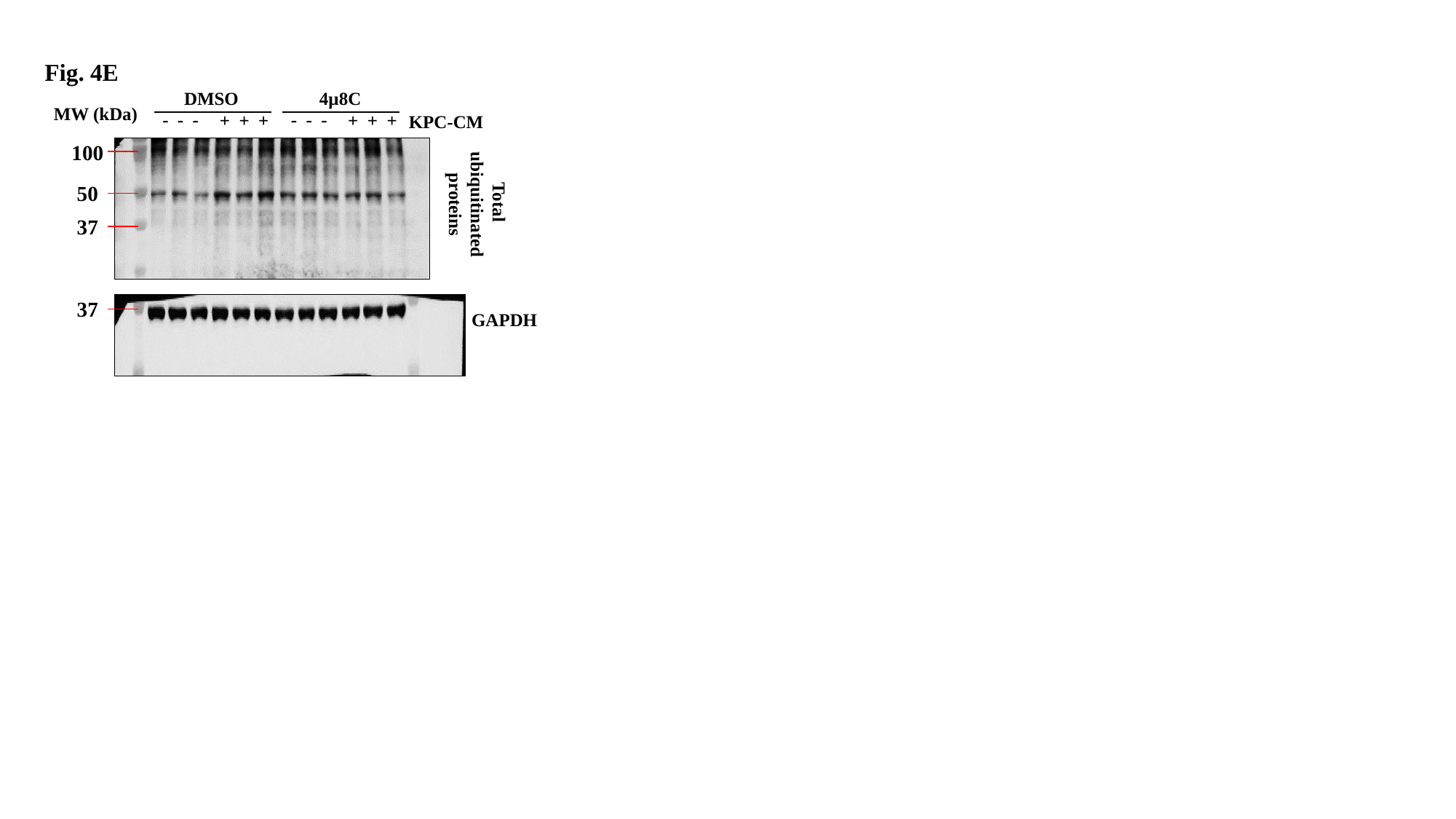

Fig. 4E
DMSO
4μ8C
MW (kDa)
- - -
- - -
+ + +
+ + +
KPC-CM
100
Total
ubiquitinated
proteins
50
37
37
GAPDH

Supplement: Supplementary file 7 — Source data Fig. 4 [file 44321_2025_337_MOESM7_ESM.zip › Figure 4/Fig4C_E_Western blot images/Fig4E_Western blot images/Fig4E_Western blot.pptx]

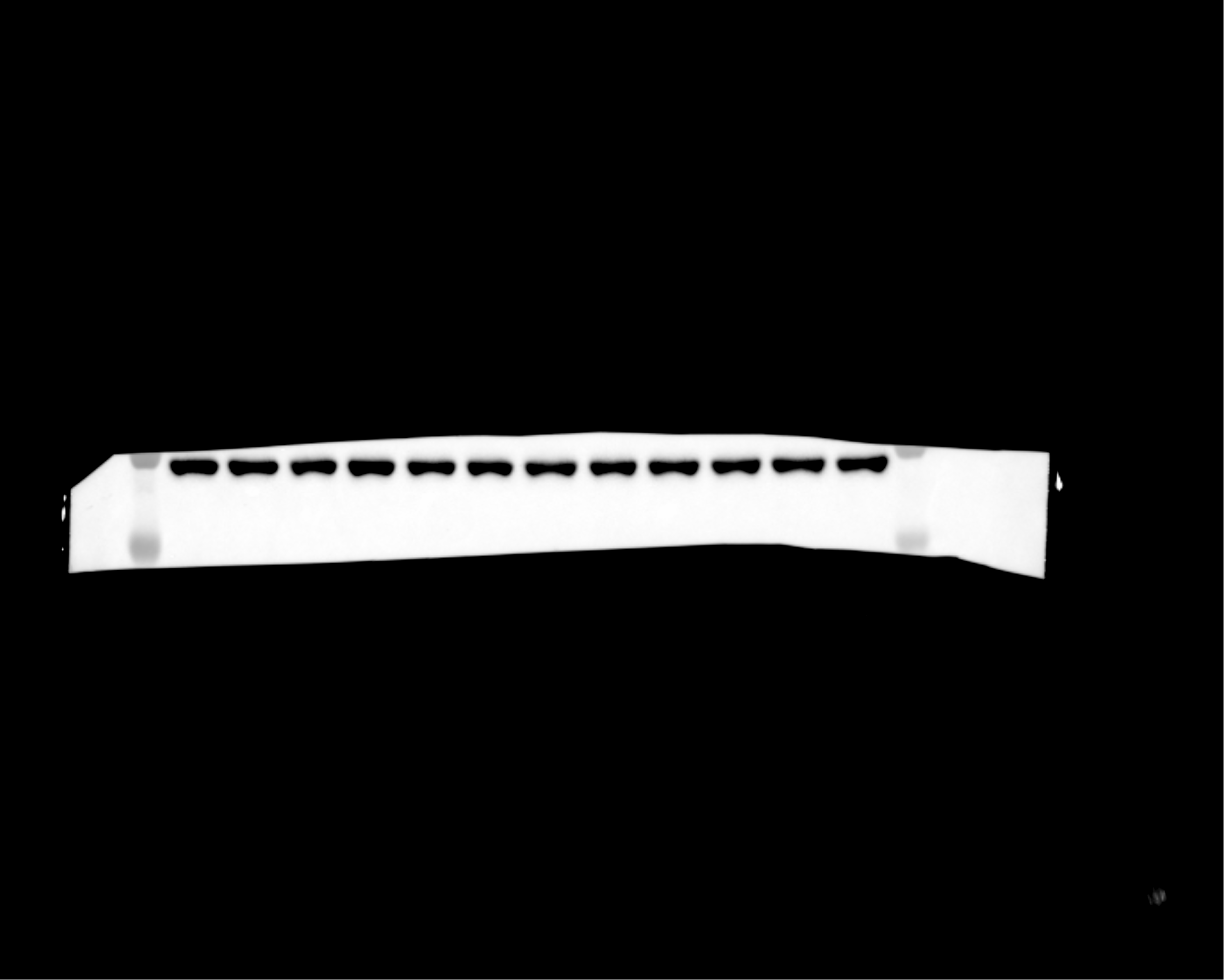

Supplement: Supplementary file 7 — Source data Fig. 4 [file 44321_2025_337_MOESM7_ESM.zip › Figure 4/Fig4C_E_Western blot images/Fig4E_Western blot images/Western GAPDH.tif]

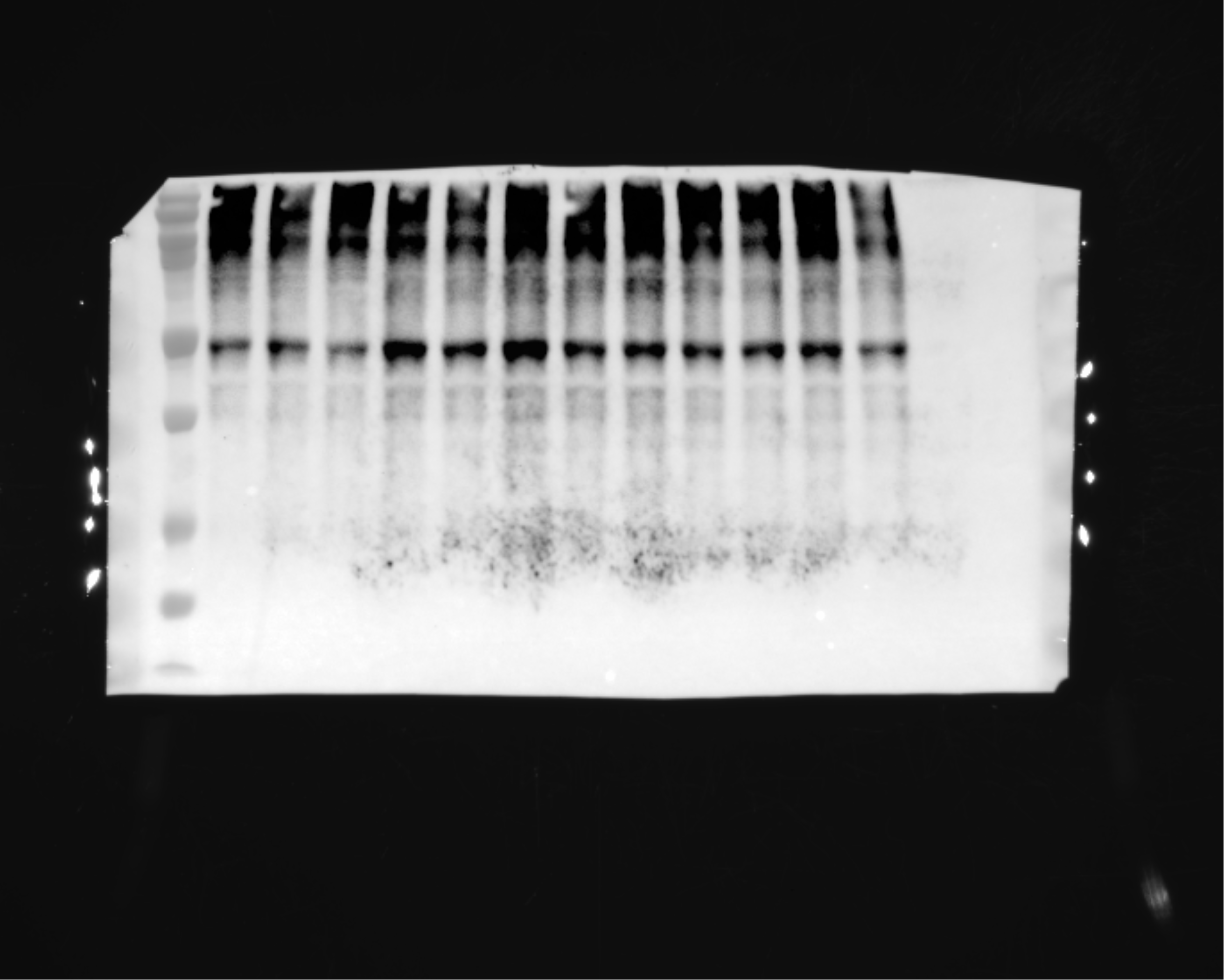

Supplement: Supplementary file 7 — Source data Fig. 4 [file 44321_2025_337_MOESM7_ESM.zip › Figure 4/Fig4C_E_Western blot images/Fig4E_Western blot images/Western Ub-conjugated proteins.tif]

## Slide 1
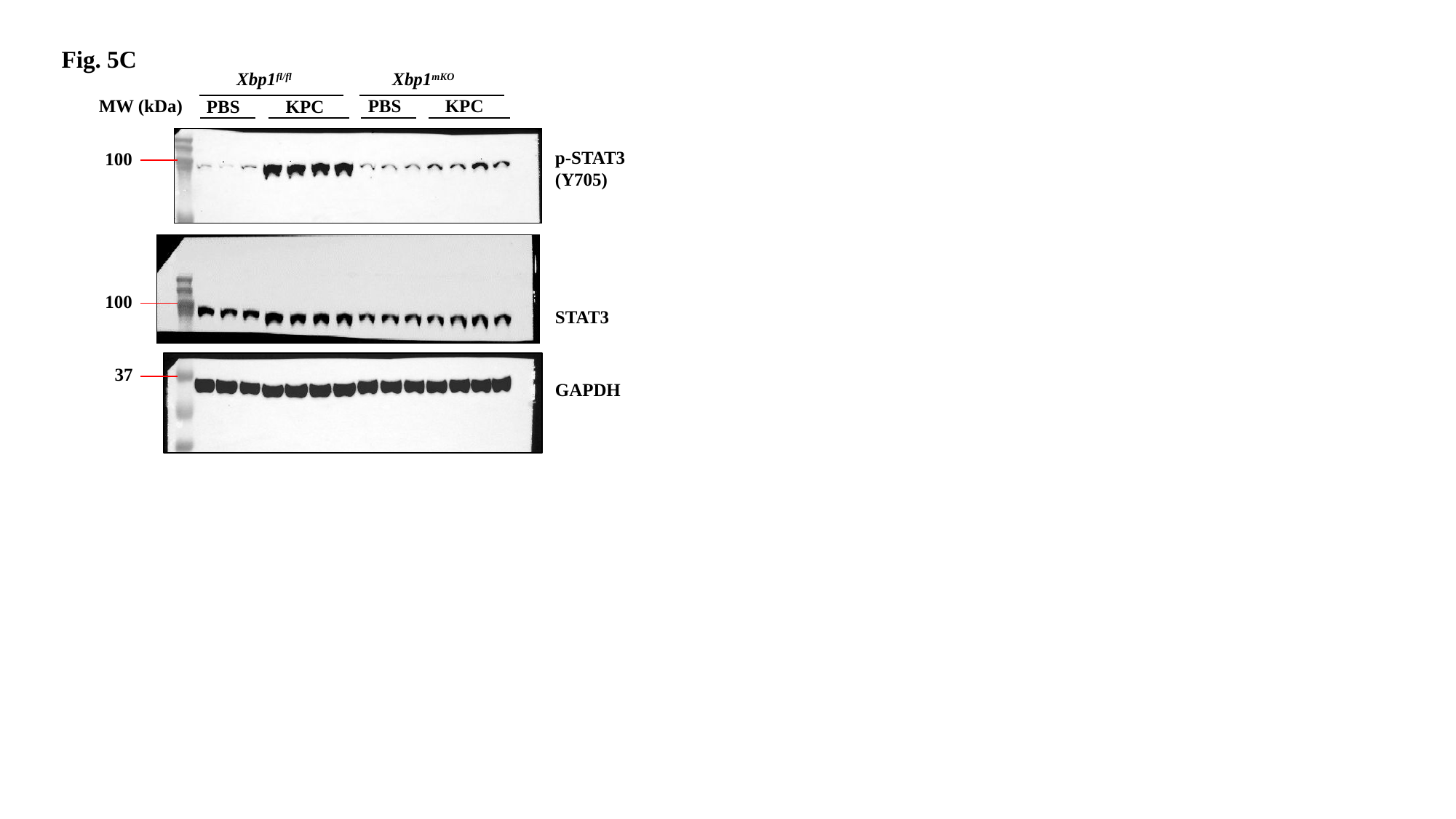

Fig. 5C
Xbp1fl/fl
Xbp1mKO
MW (kDa)
PBS
KPC
PBS
KPC
p-STAT3
(Y705)
100
100
STAT3
37
GAPDH

Supplement: Supplementary file 8 — Source data Fig. 5 [file 44321_2025_337_MOESM8_ESM.zip › Figure 5/Fig5C_E_G_K_Western blot/Fig5C_Western blot/Fig5C_Western blot.pptx]

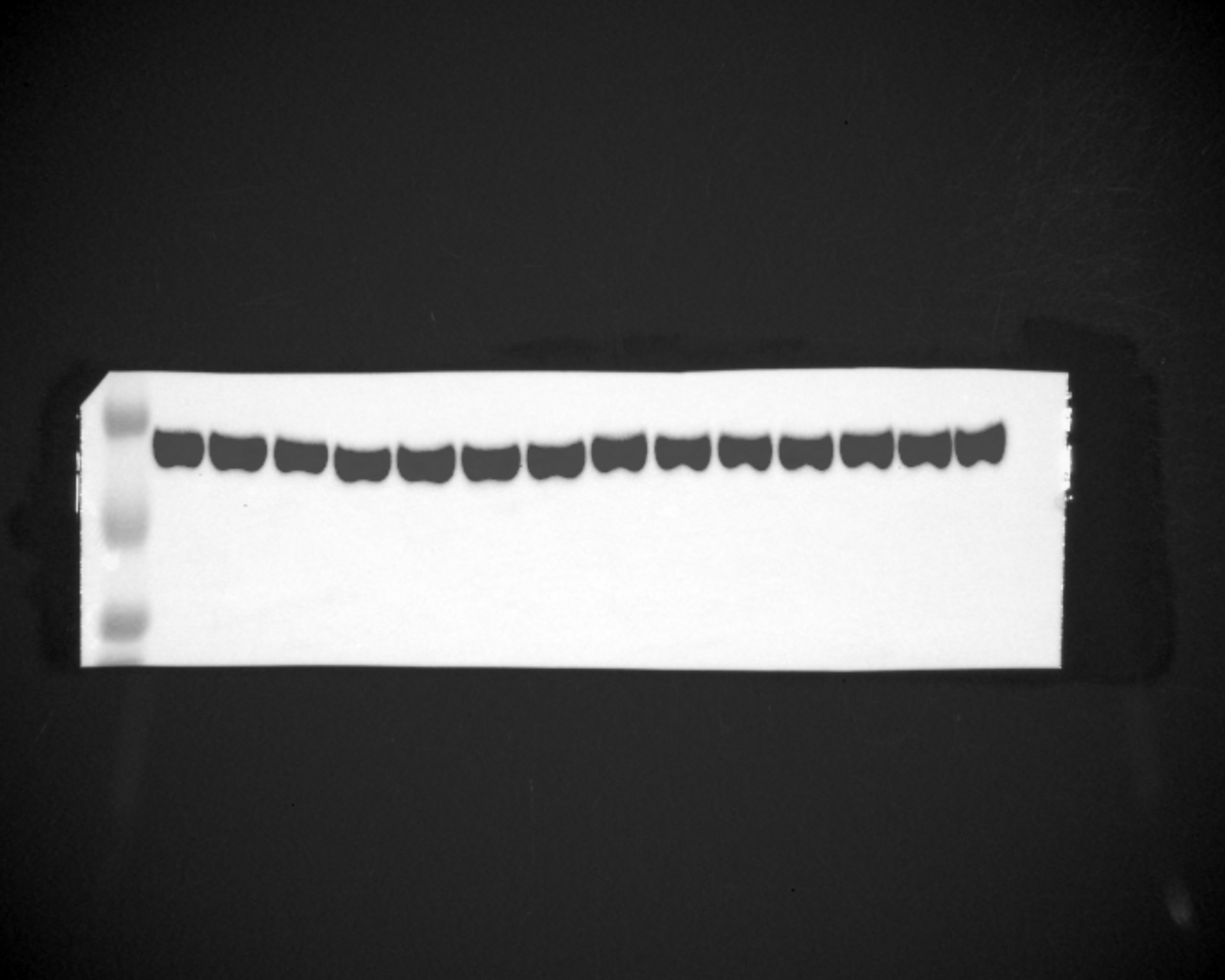

Supplement: Supplementary file 8 — Source data Fig. 5 [file 44321_2025_337_MOESM8_ESM.zip › Figure 5/Fig5C_E_G_K_Western blot/Fig5C_Western blot/Western GAPDH.tif]

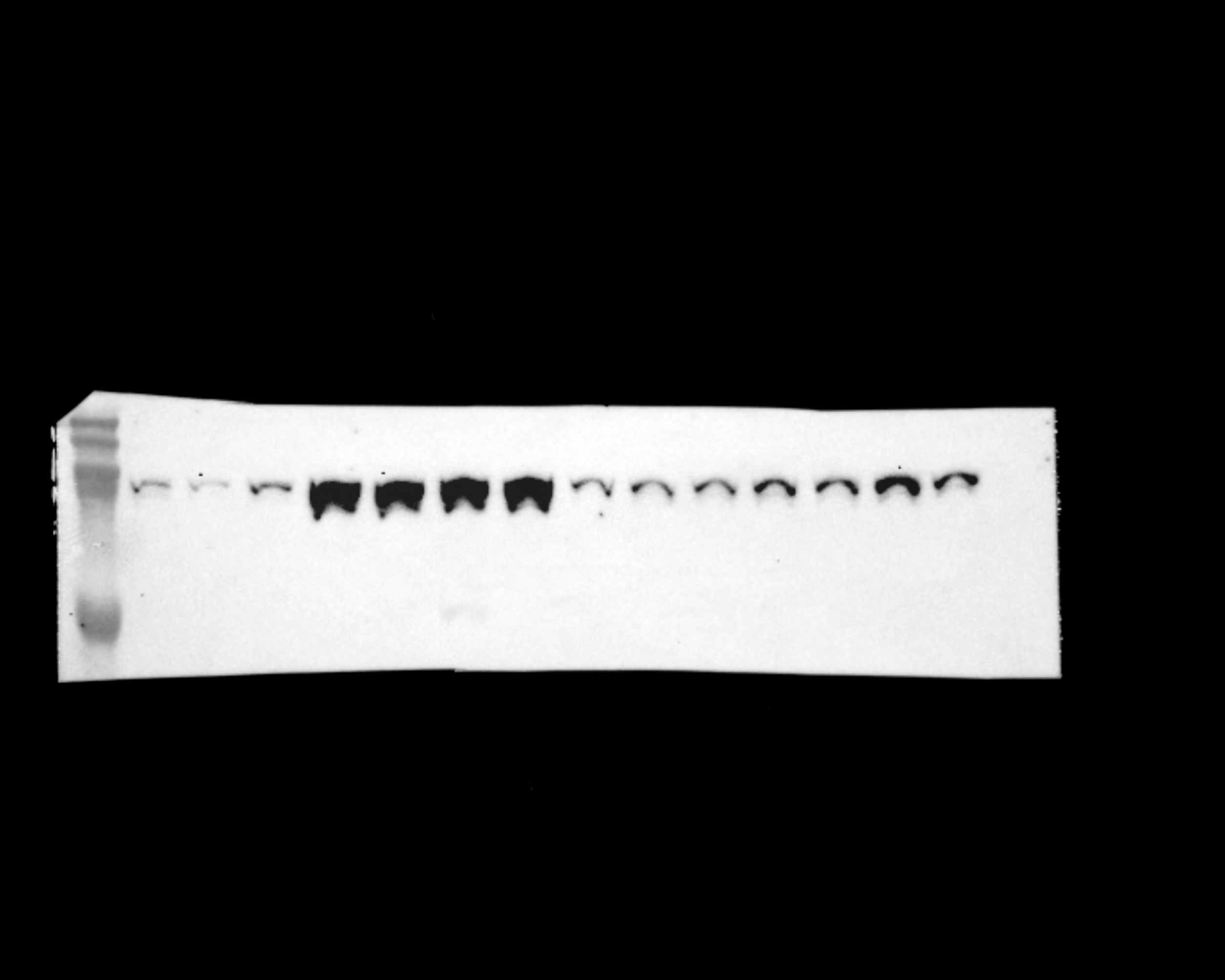

Supplement: Supplementary file 8 — Source data Fig. 5 [file 44321_2025_337_MOESM8_ESM.zip › Figure 5/Fig5C_E_G_K_Western blot/Fig5C_Western blot/Western p-STAT3-Y705.tif]

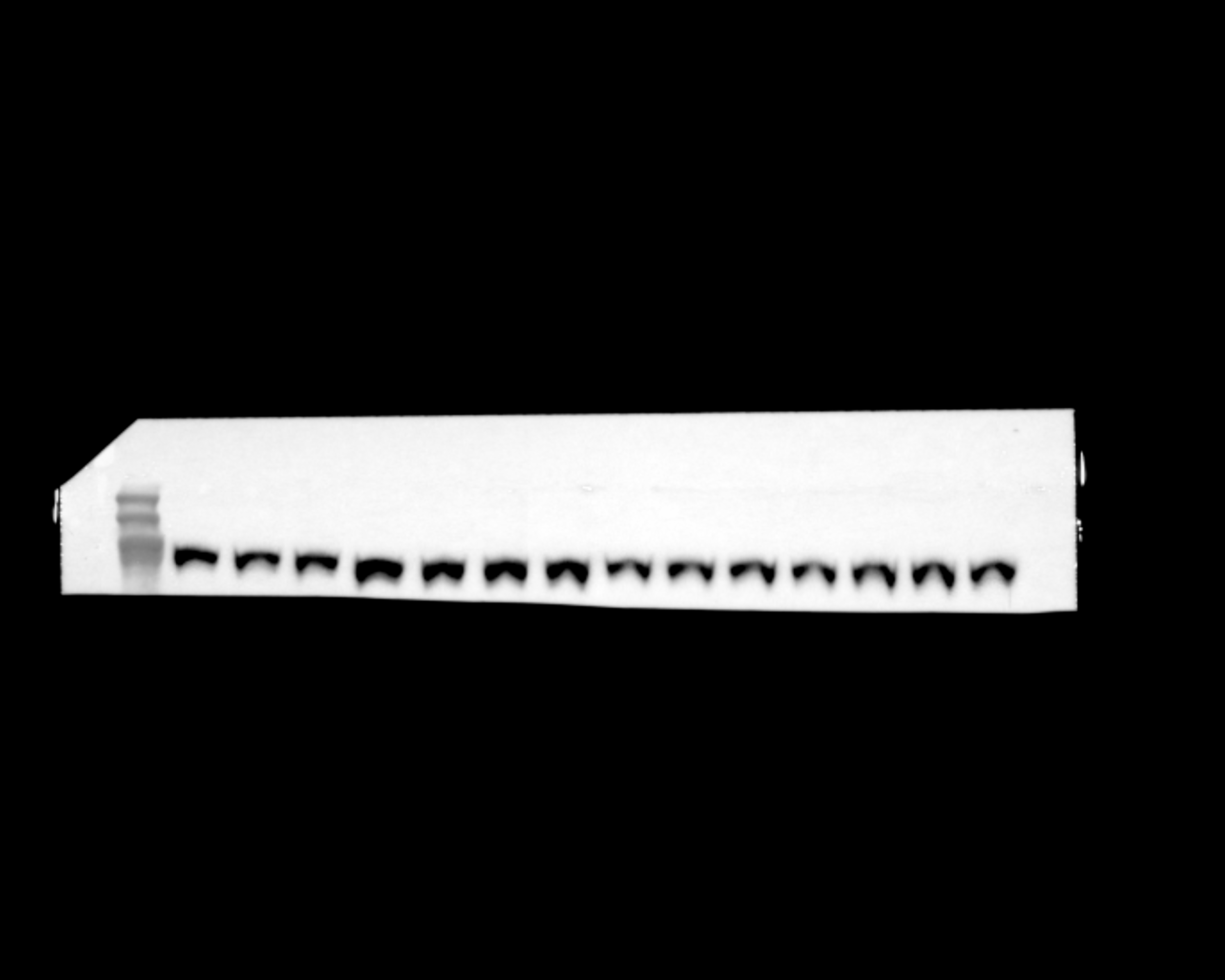

Supplement: Supplementary file 8 — Source data Fig. 5 [file 44321_2025_337_MOESM8_ESM.zip › Figure 5/Fig5C_E_G_K_Western blot/Fig5C_Western blot/Western STAT3.tif]

## Slide 1
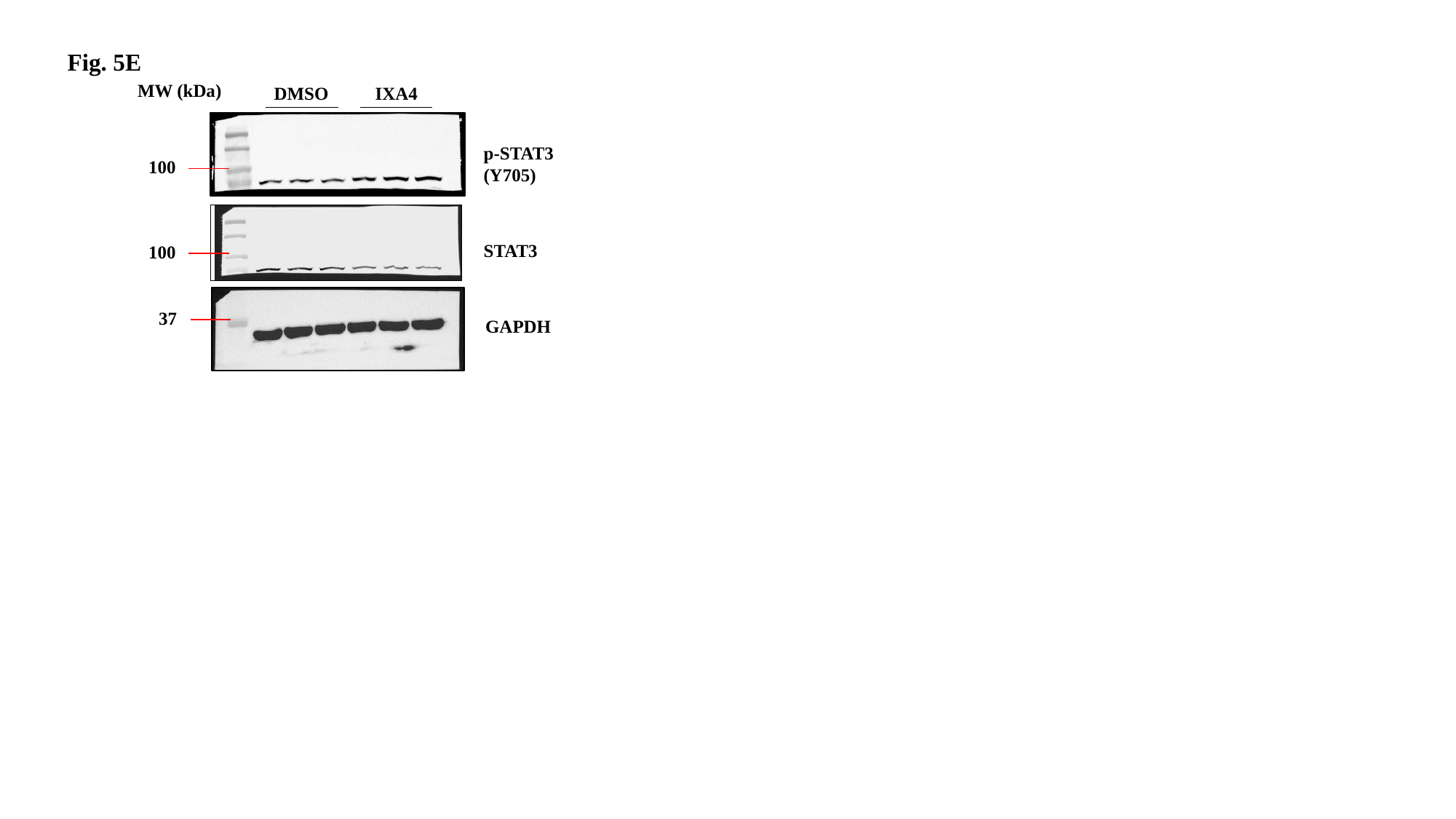

Fig. 5E
MW (kDa)
DMSO
IXA4
p-STAT3
(Y705)
100
STAT3
100
37
GAPDH

Supplement: Supplementary file 8 — Source data Fig. 5 [file 44321_2025_337_MOESM8_ESM.zip › Figure 5/Fig5C_E_G_K_Western blot/Fig5E_Western blot/Fig5E_Western blot.pptx]

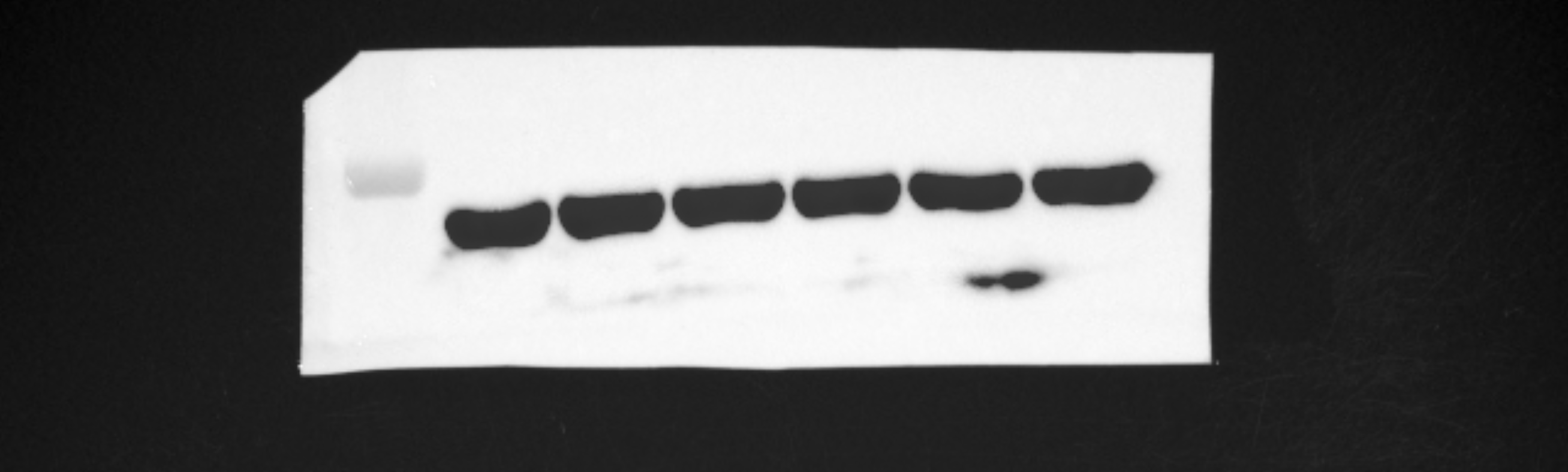

Supplement: Supplementary file 8 — Source data Fig. 5 [file 44321_2025_337_MOESM8_ESM.zip › Figure 5/Fig5C_E_G_K_Western blot/Fig5E_Western blot/Western GAPDH.tif]

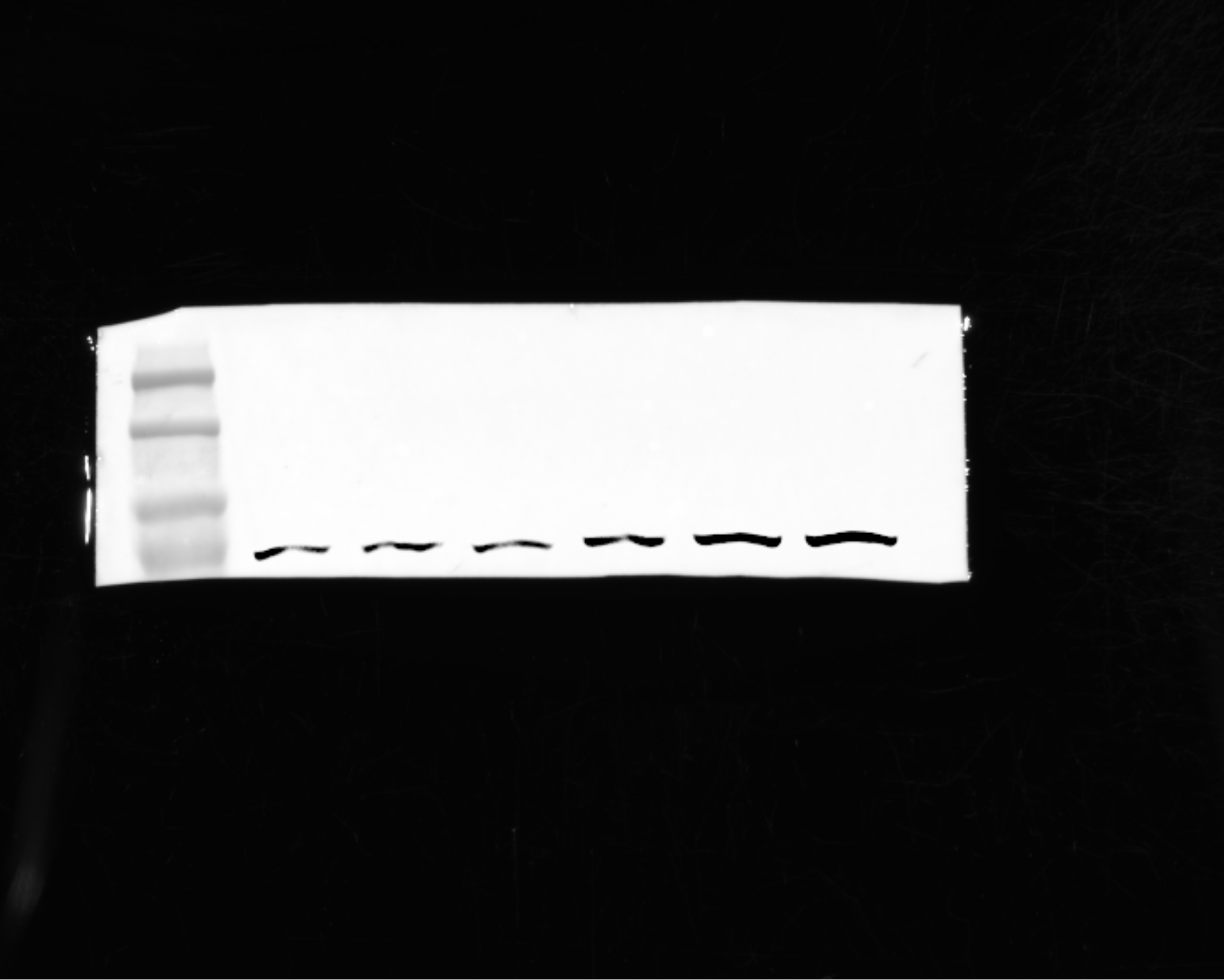

Supplement: Supplementary file 8 — Source data Fig. 5 [file 44321_2025_337_MOESM8_ESM.zip › Figure 5/Fig5C_E_G_K_Western blot/Fig5E_Western blot/Western p-STAT3-Y705.tif]

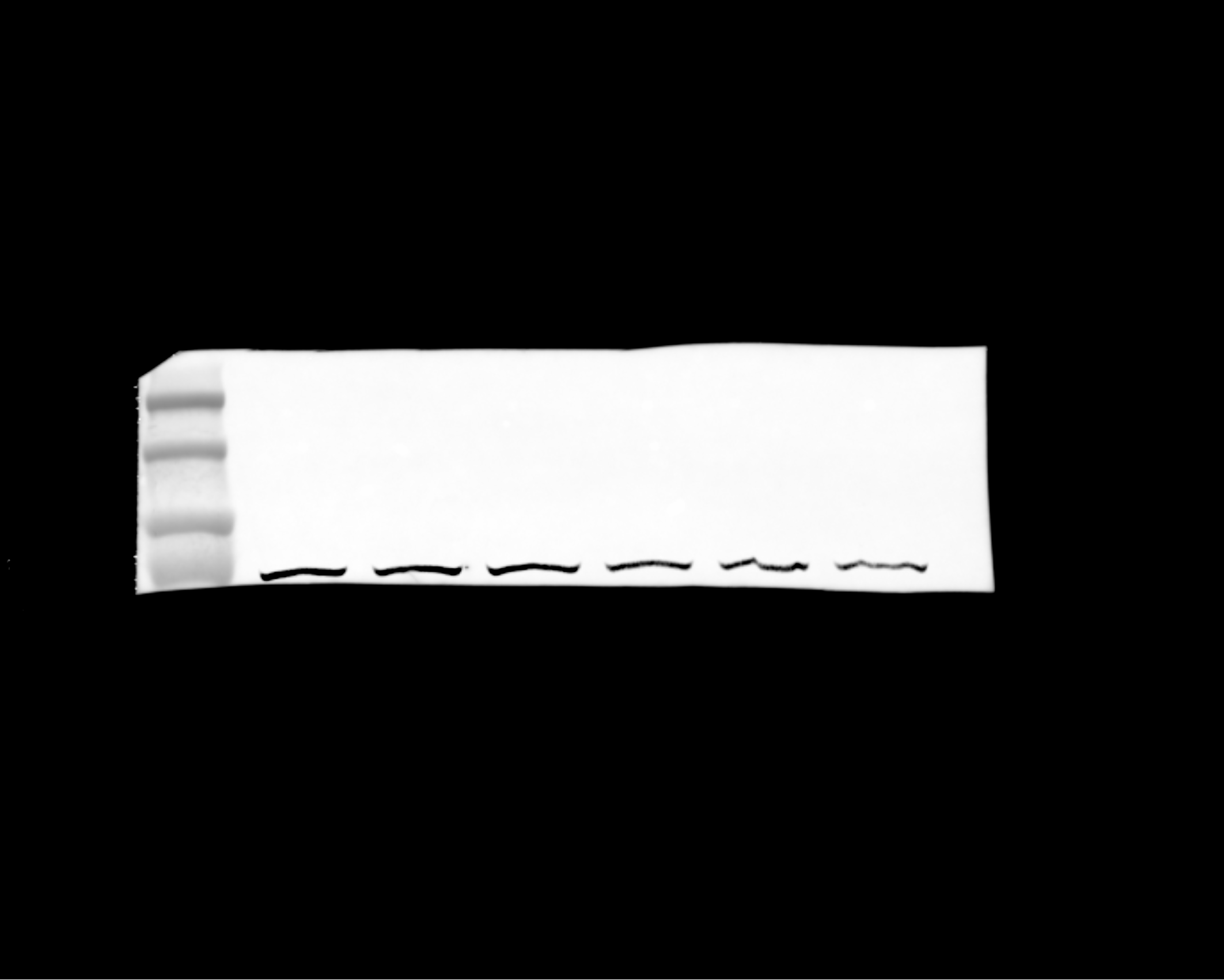

Supplement: Supplementary file 8 — Source data Fig. 5 [file 44321_2025_337_MOESM8_ESM.zip › Figure 5/Fig5C_E_G_K_Western blot/Fig5E_Western blot/Western STAT3.tif]

## Slide 1
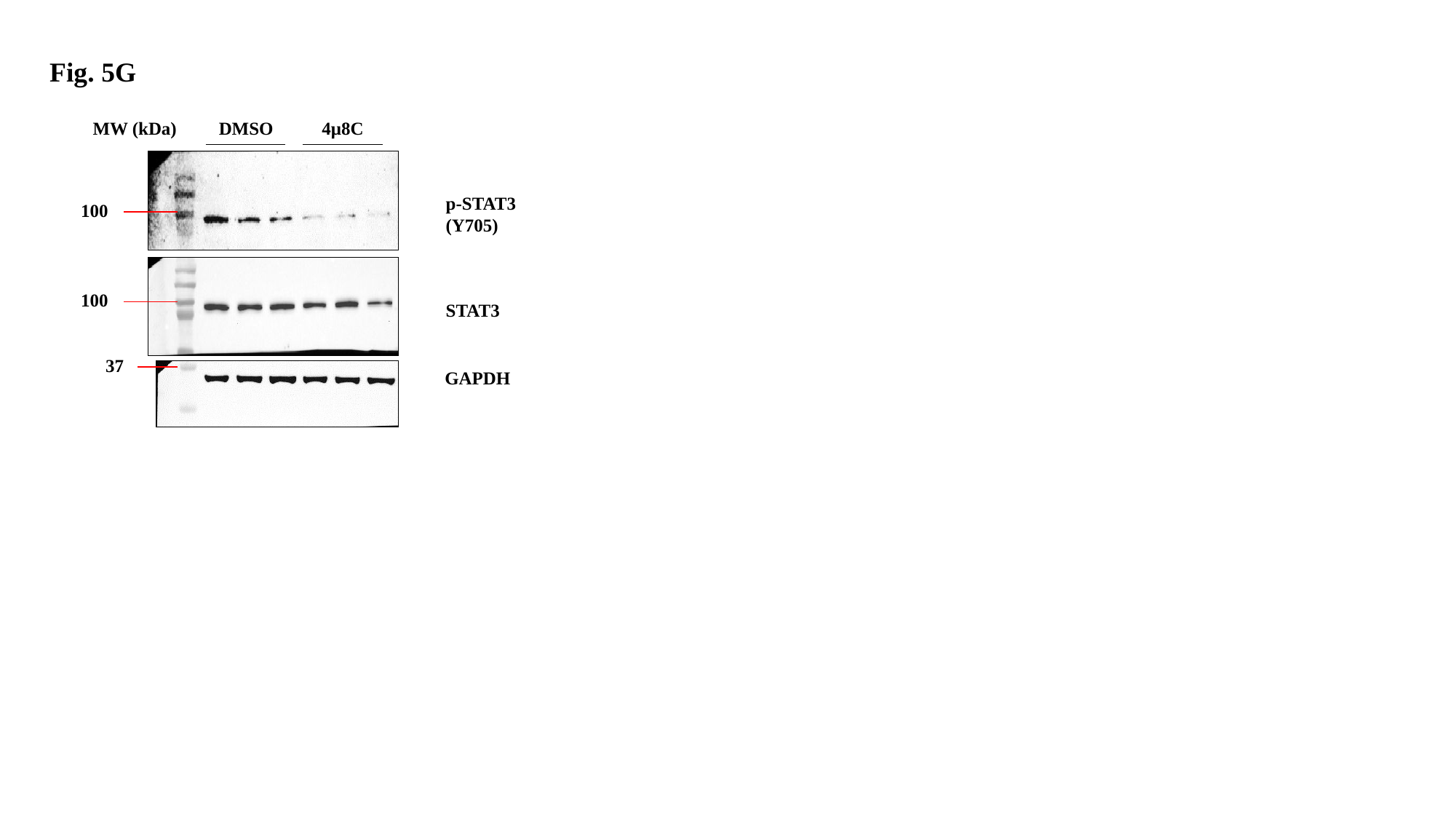

Fig. 5G
MW (kDa)
DMSO
4μ8C
p-STAT3
(Y705)
100
100
STAT3
37
GAPDH

Supplement: Supplementary file 8 — Source data Fig. 5 [file 44321_2025_337_MOESM8_ESM.zip › Figure 5/Fig5C_E_G_K_Western blot/Fig5G_Western blot/Fig5G_Western blot.pptx]

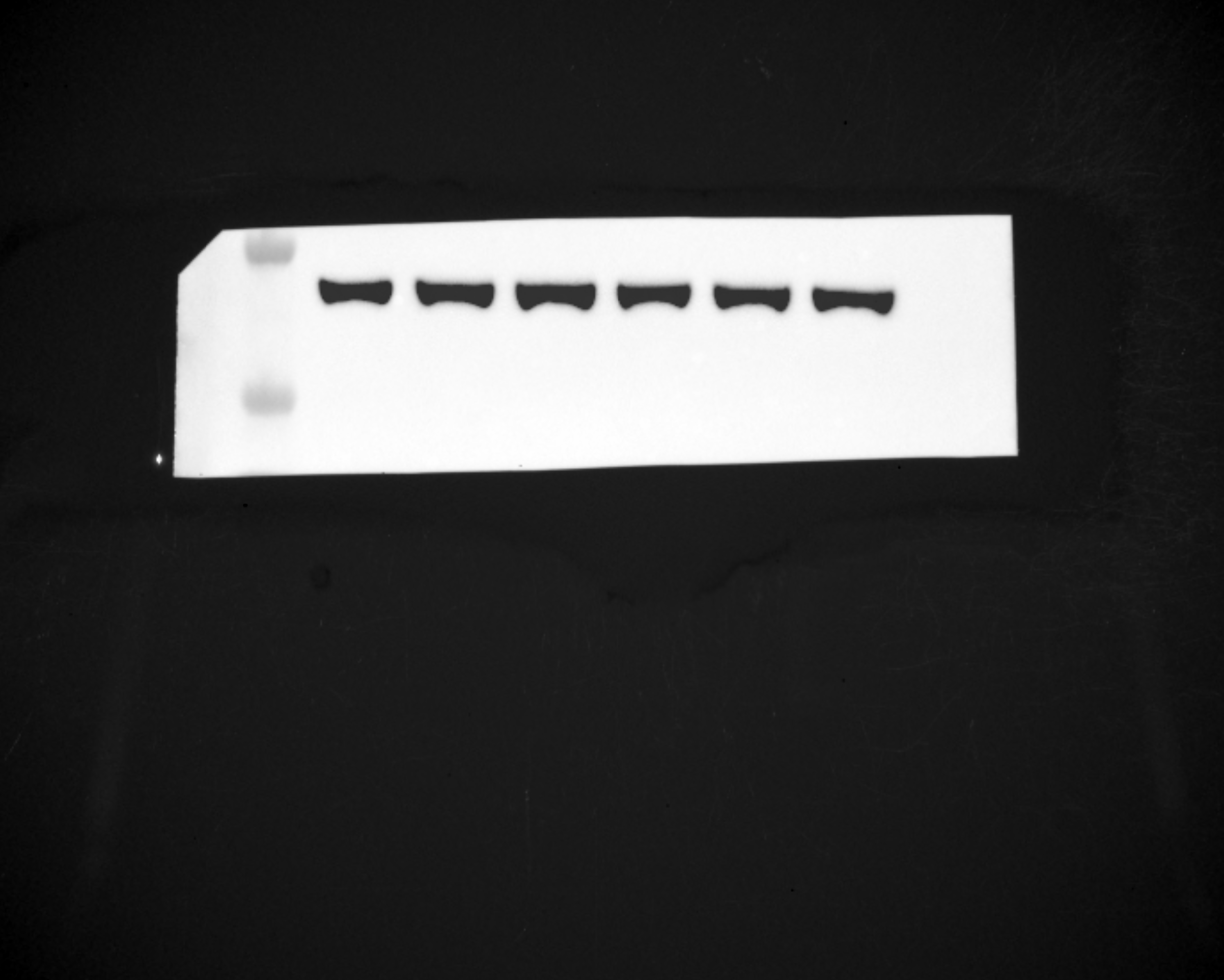

Supplement: Supplementary file 8 — Source data Fig. 5 [file 44321_2025_337_MOESM8_ESM.zip › Figure 5/Fig5C_E_G_K_Western blot/Fig5G_Western blot/Western GAPDH.tif]

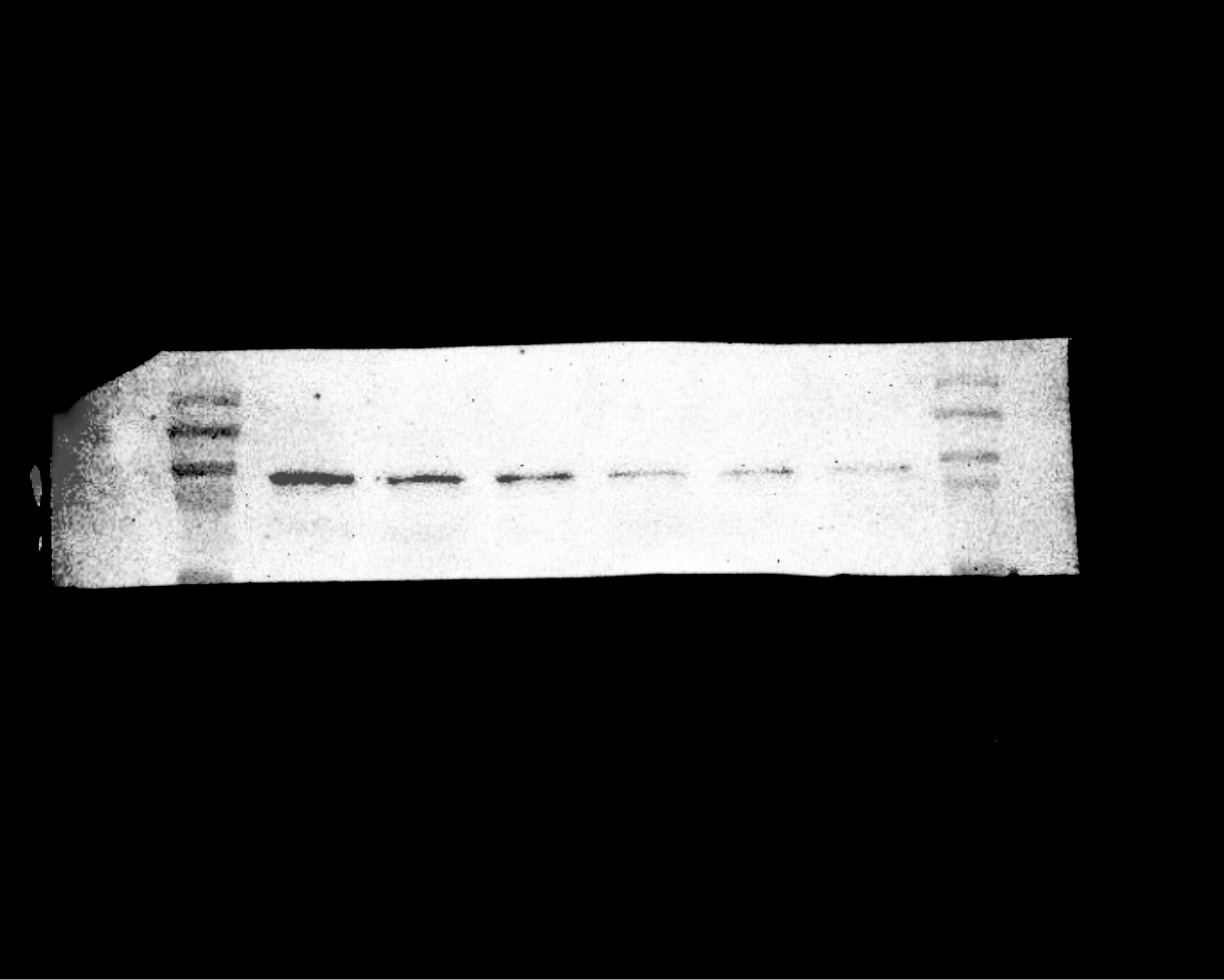

Supplement: Supplementary file 8 — Source data Fig. 5 [file 44321_2025_337_MOESM8_ESM.zip › Figure 5/Fig5C_E_G_K_Western blot/Fig5G_Western blot/Western p-STAT3-Y705.tif]

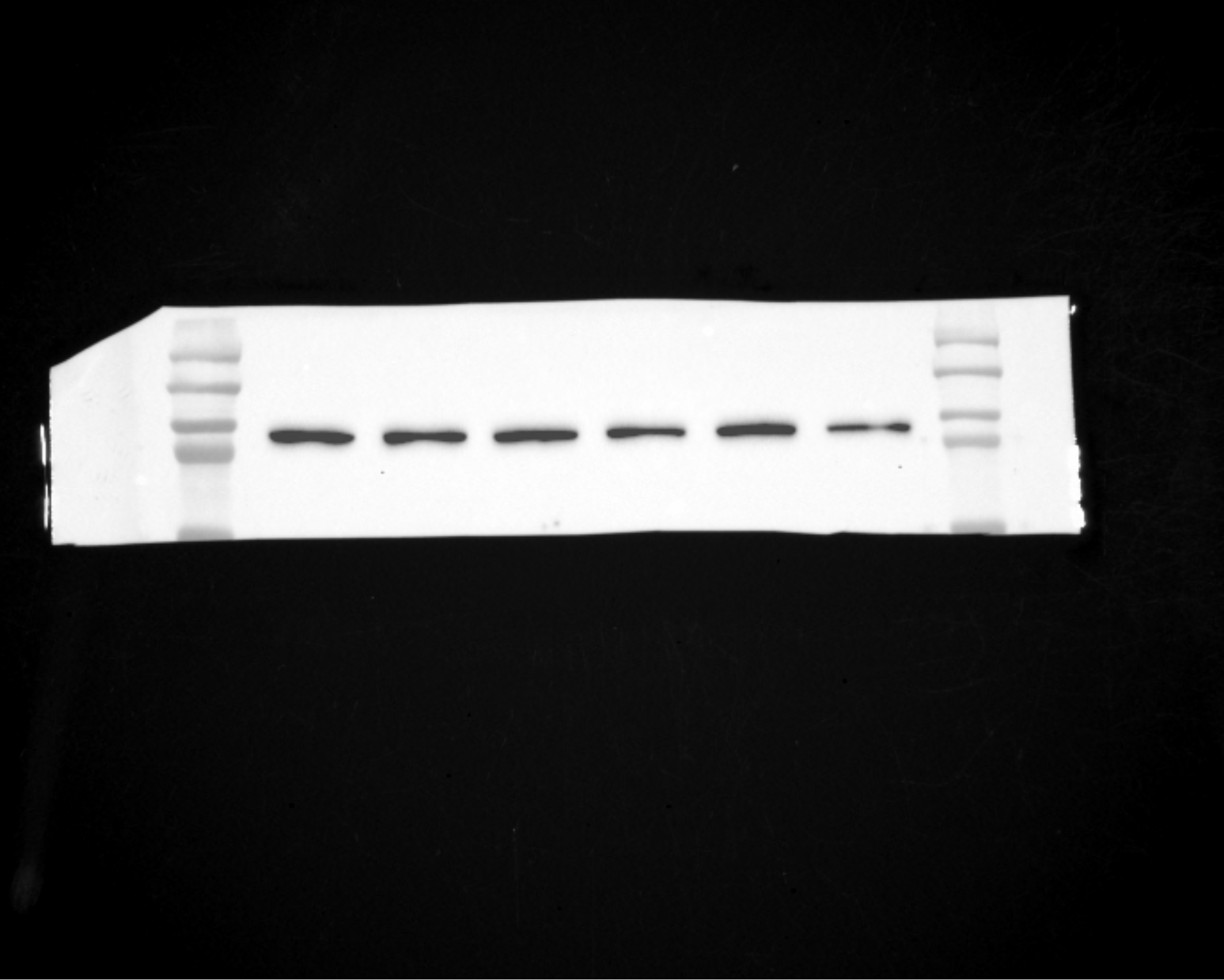

Supplement: Supplementary file 8 — Source data Fig. 5 [file 44321_2025_337_MOESM8_ESM.zip › Figure 5/Fig5C_E_G_K_Western blot/Fig5G_Western blot/Western STAT3.tif]

## Slide 1
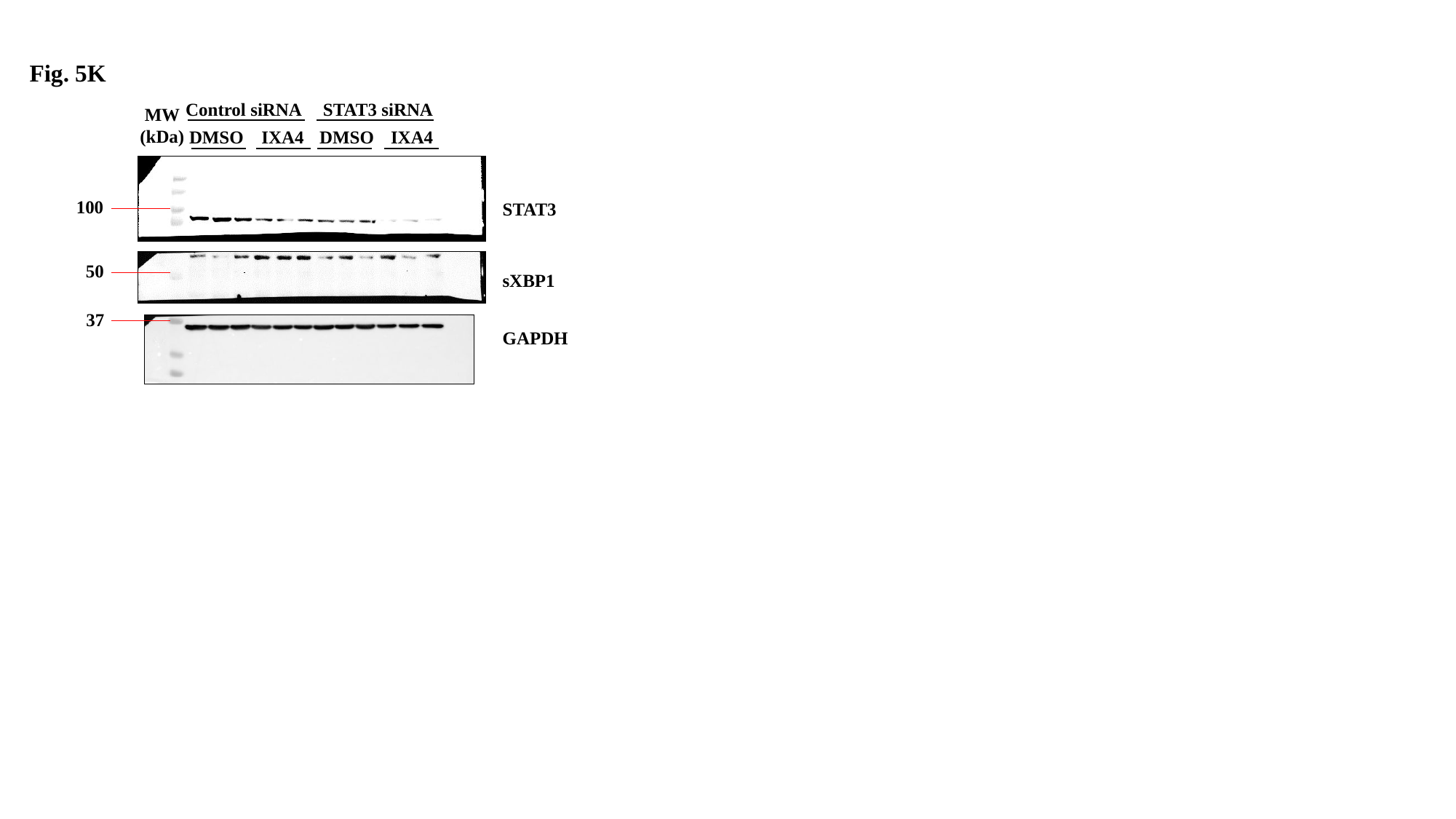

Fig. 5K
Control siRNA
STAT3 siRNA
MW (kDa)
DMSO
IXA4
DMSO
IXA4
100
STAT3
50
sXBP1
37
GAPDH

Supplement: Supplementary file 8 — Source data Fig. 5 [file 44321_2025_337_MOESM8_ESM.zip › Figure 5/Fig5C_E_G_K_Western blot/Fig5K_Western blot/Fig5K_Western blot.pptx]

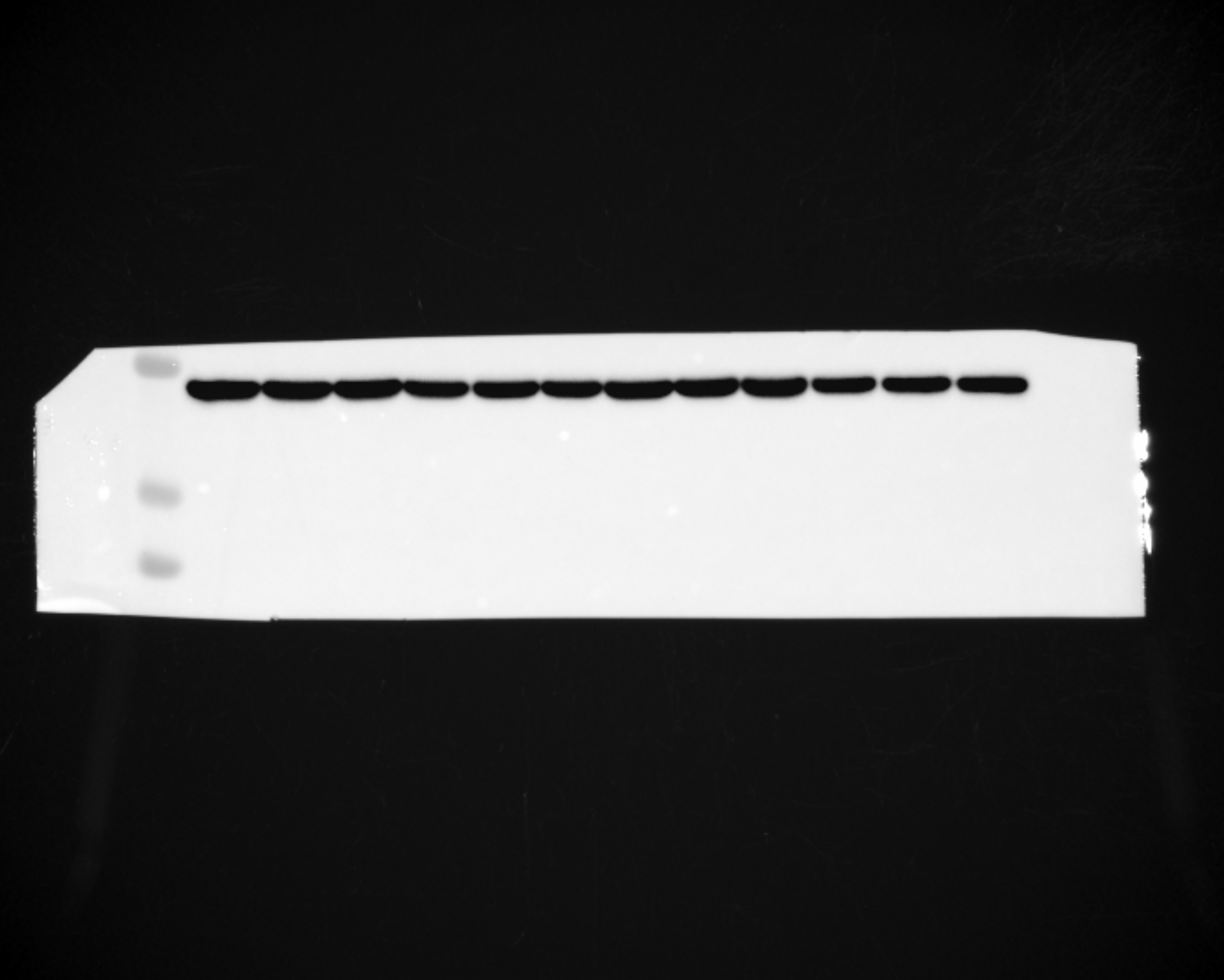

Supplement: Supplementary file 8 — Source data Fig. 5 [file 44321_2025_337_MOESM8_ESM.zip › Figure 5/Fig5C_E_G_K_Western blot/Fig5K_Western blot/Western GAPDH.tif]

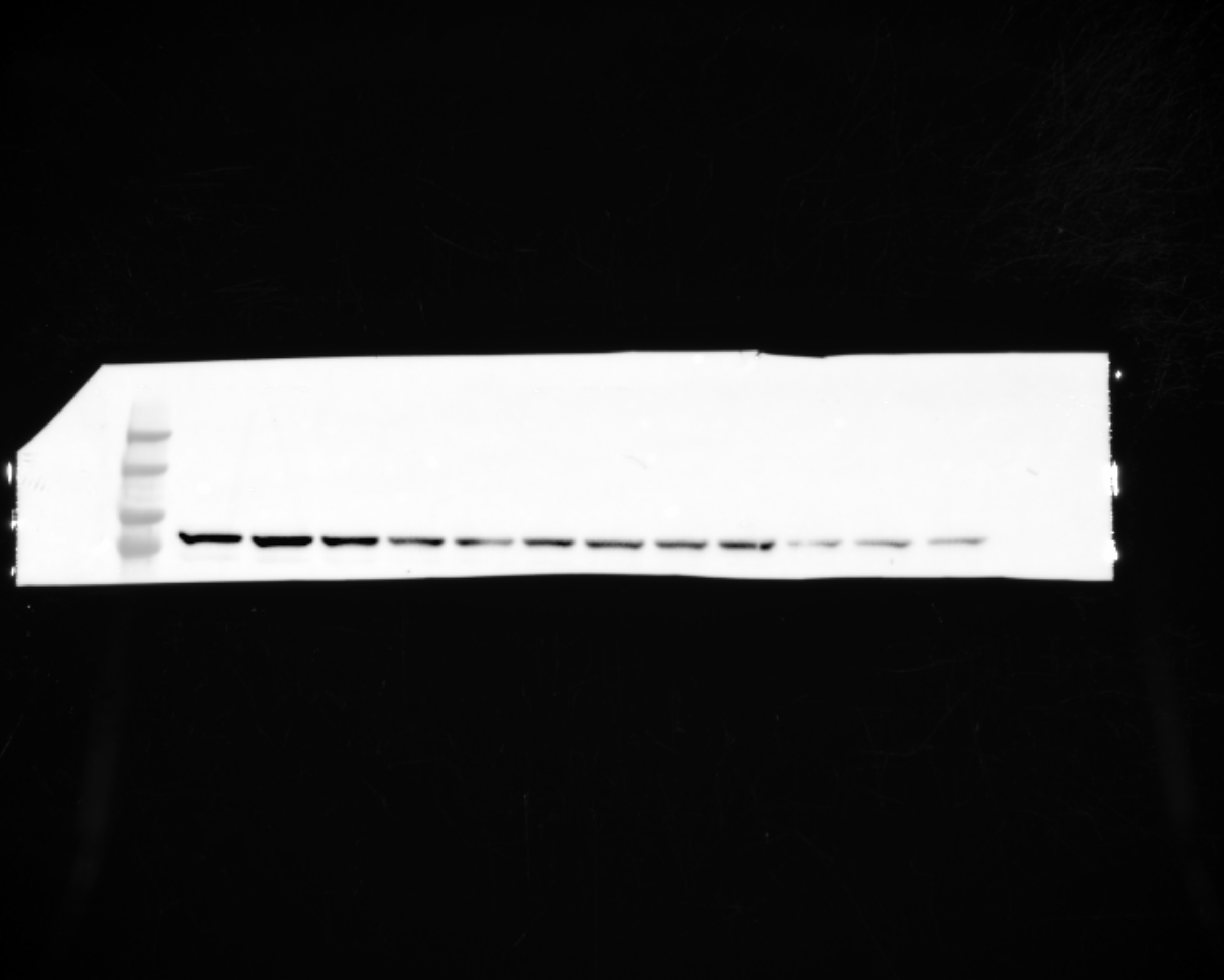

Supplement: Supplementary file 8 — Source data Fig. 5 [file 44321_2025_337_MOESM8_ESM.zip › Figure 5/Fig5C_E_G_K_Western blot/Fig5K_Western blot/Western STAT3.tif]

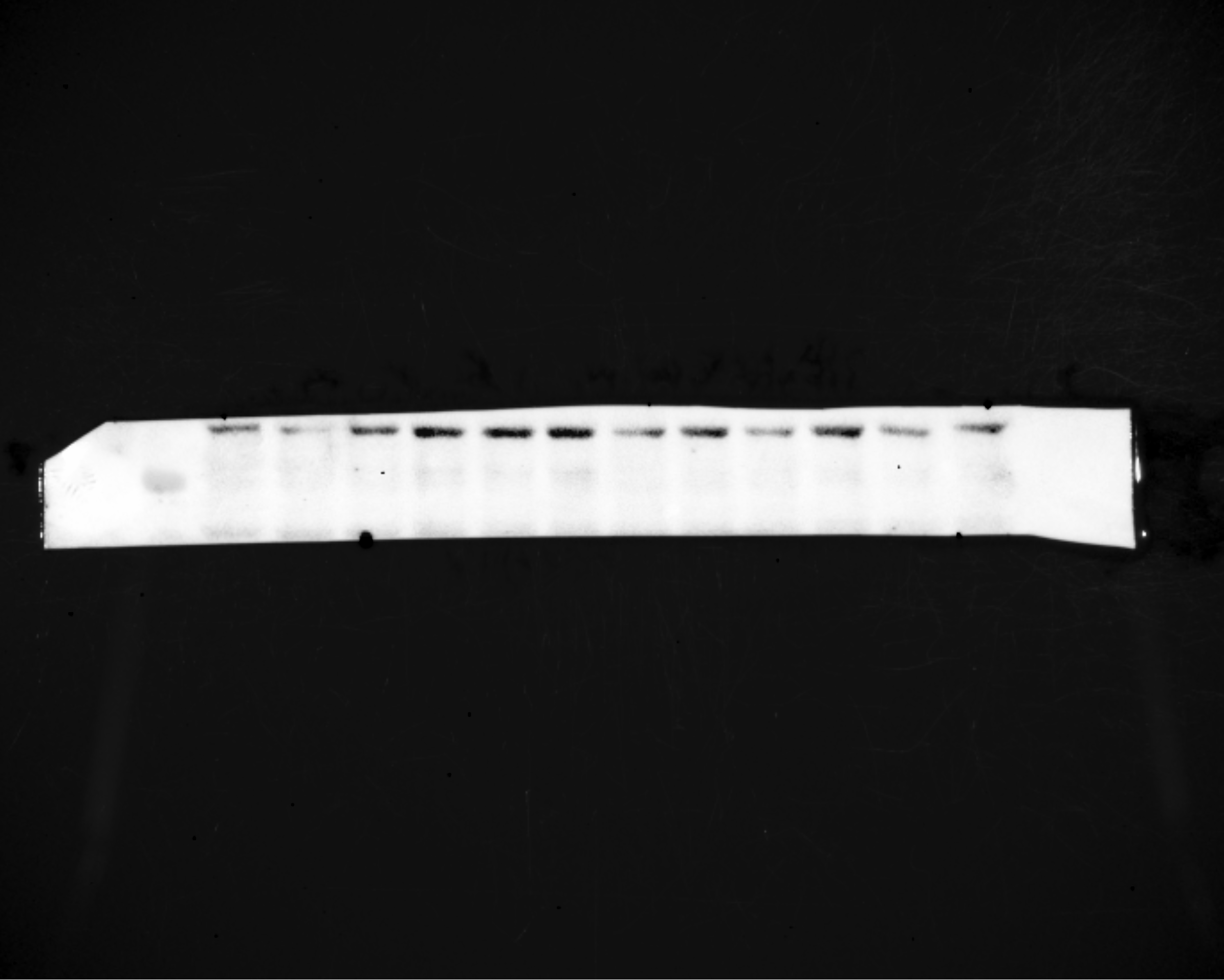

Supplement: Supplementary file 8 — Source data Fig. 5 [file 44321_2025_337_MOESM8_ESM.zip › Figure 5/Fig5C_E_G_K_Western blot/Fig5K_Western blot/Western sXBP1.tif]

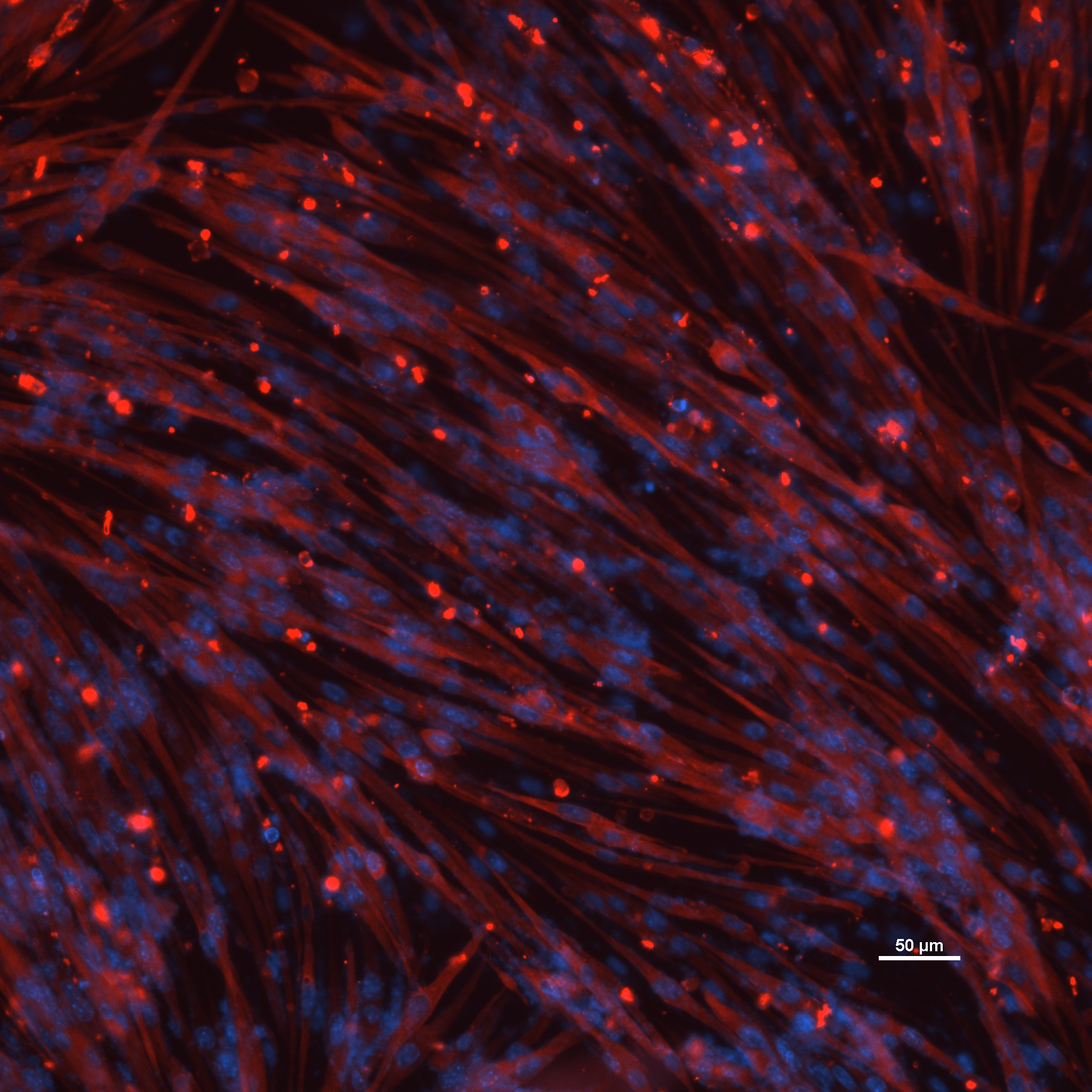

Supplement: Supplementary file 8 — Source data Fig. 5 [file 44321_2025_337_MOESM8_ESM.zip › Figure 5/Fig5I_MyHC staining/Fig5I_MyHC staining_Representative images/IXA4-Control siRNA.tif]

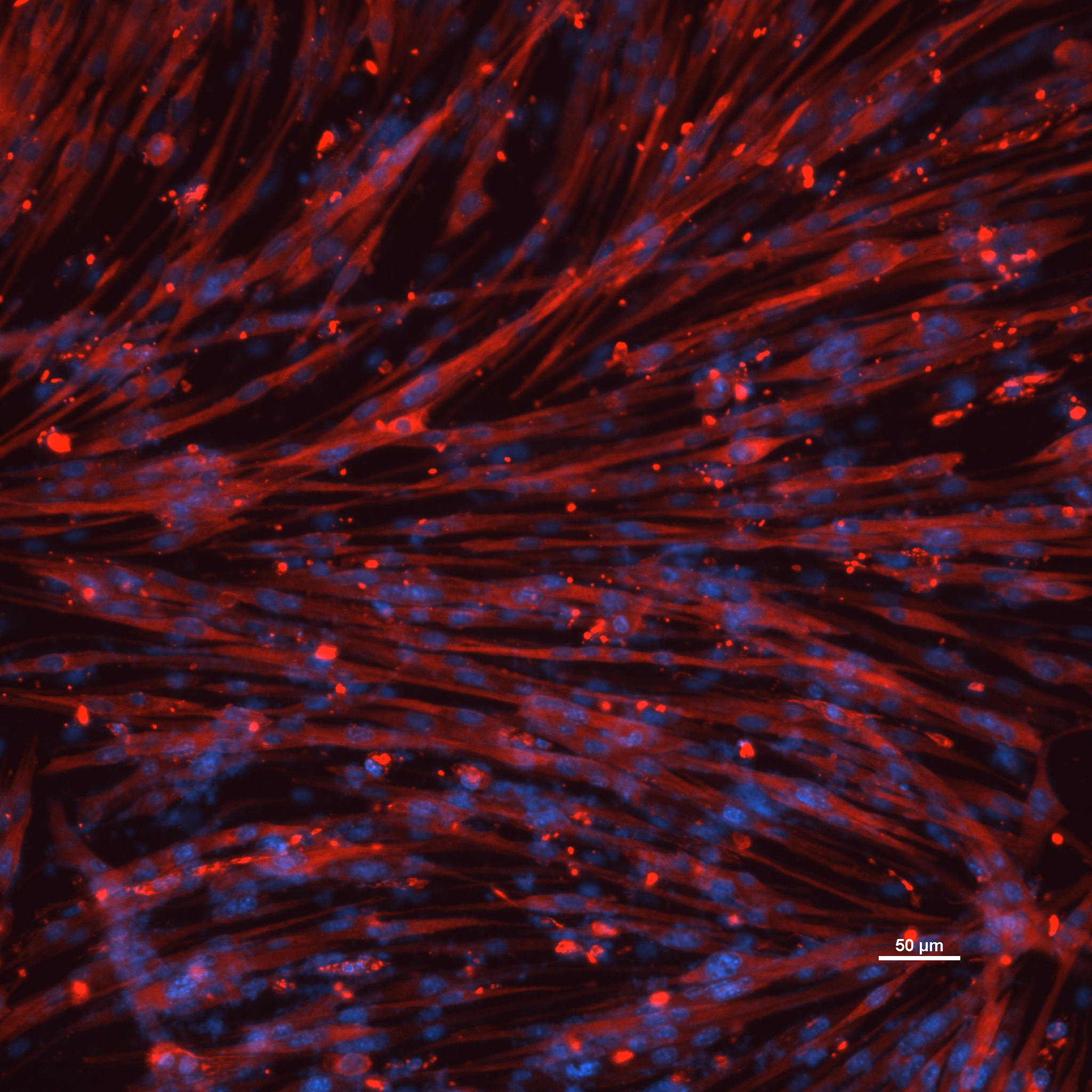

Supplement: Supplementary file 8 — Source data Fig. 5 [file 44321_2025_337_MOESM8_ESM.zip › Figure 5/Fig5I_MyHC staining/Fig5I_MyHC staining_Representative images/IXA4-STAT3 siRNA.tif]

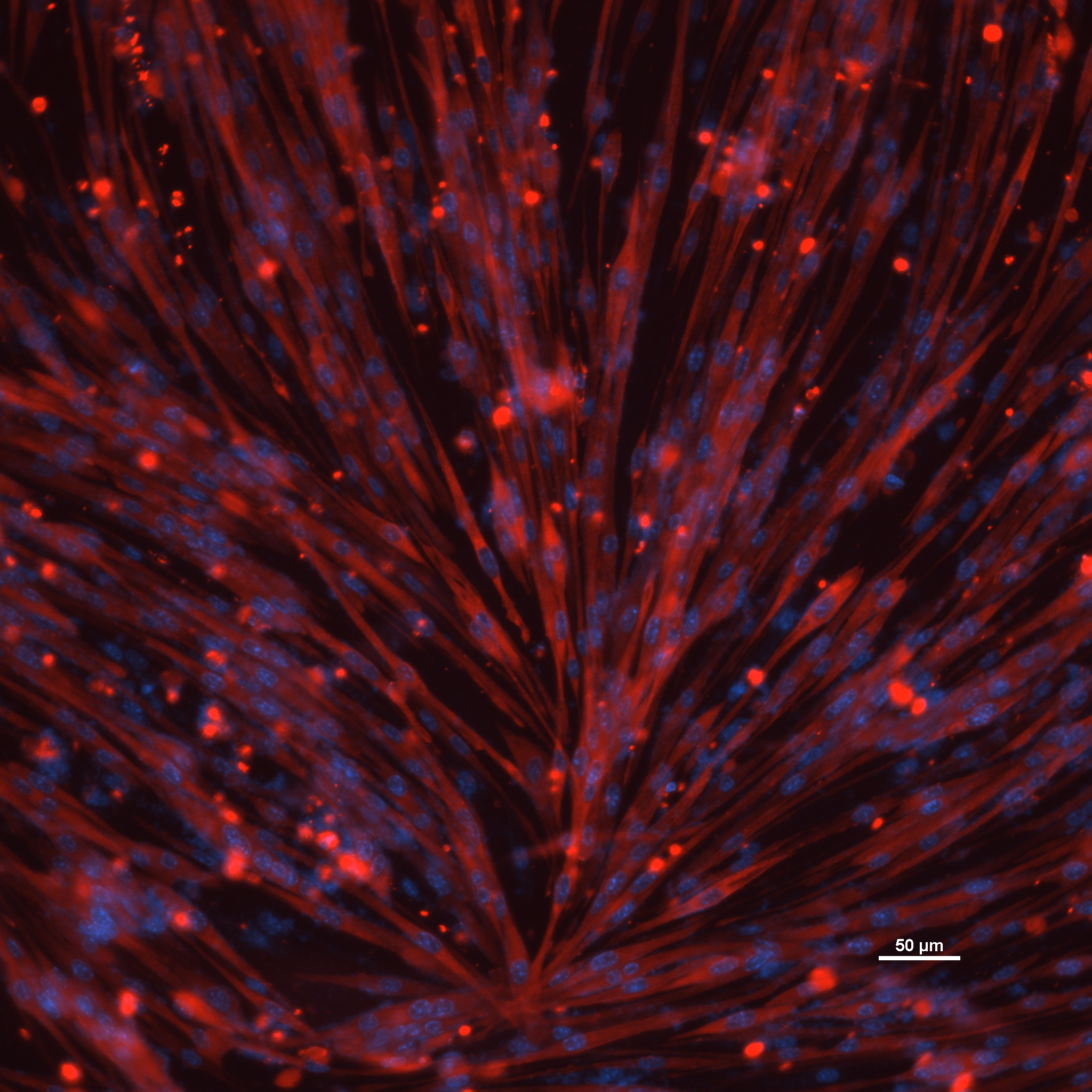

Supplement: Supplementary file 8 — Source data Fig. 5 [file 44321_2025_337_MOESM8_ESM.zip › Figure 5/Fig5I_MyHC staining/Fig5I_MyHC staining_Representative images/Vehicle-Control siRNA.tif]

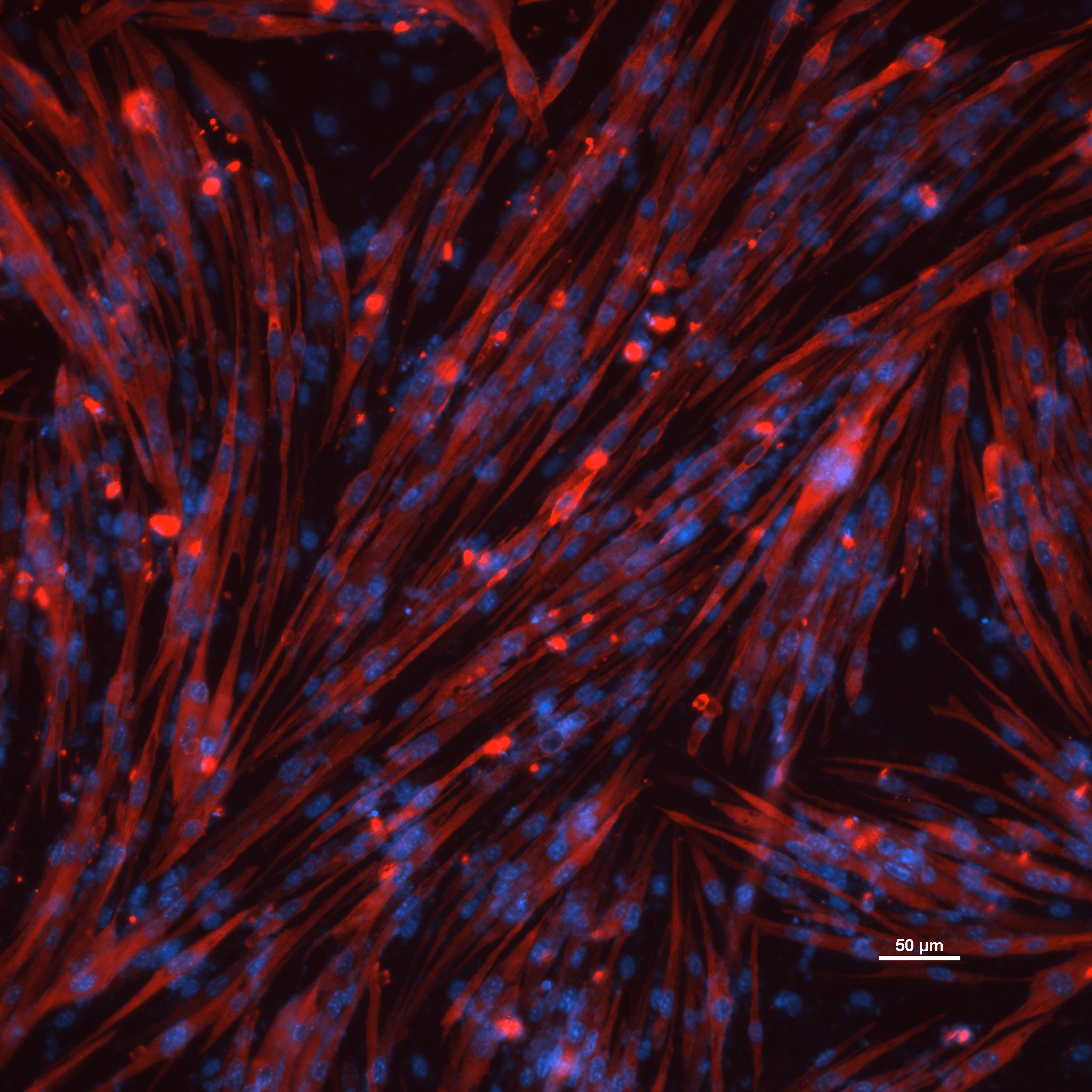

Supplement: Supplementary file 8 — Source data Fig. 5 [file 44321_2025_337_MOESM8_ESM.zip › Figure 5/Fig5I_MyHC staining/Fig5I_MyHC staining_Representative images/Vehicle-STAT3 siRNA.tif]

## Slide 1
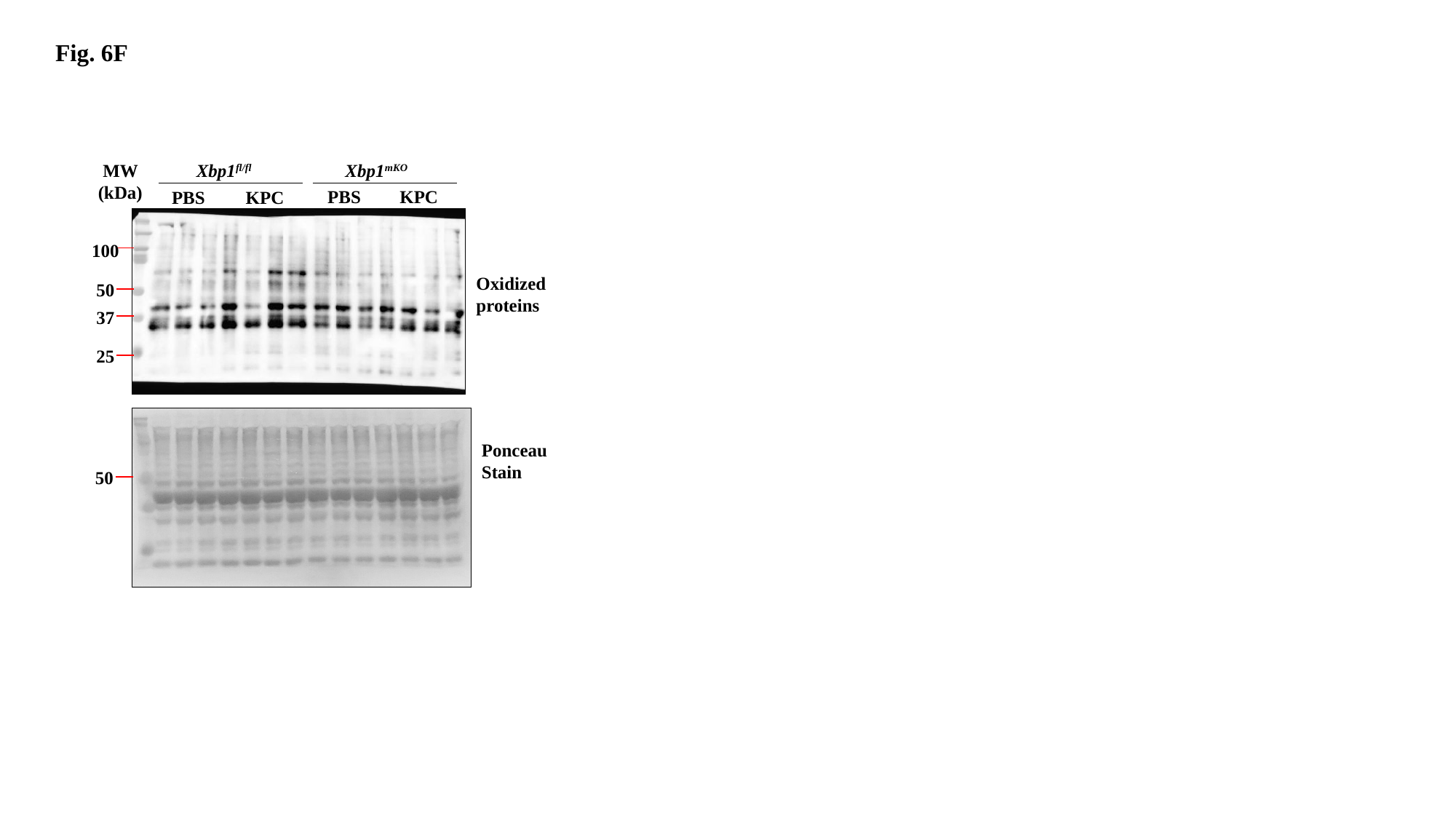

Fig. 6F
Xbp1fl/fl
Xbp1mKO
MW (kDa)
PBS
KPC
PBS
KPC
100
Oxidized proteins
50
37
25
Ponceau Stain
50

Supplement: Supplementary file 9 — Source data Fig. 6 [file 44321_2025_337_MOESM9_ESM.zip › Figure 6/Fig6F_Western blot/Fig6F_Western blot.pptx]

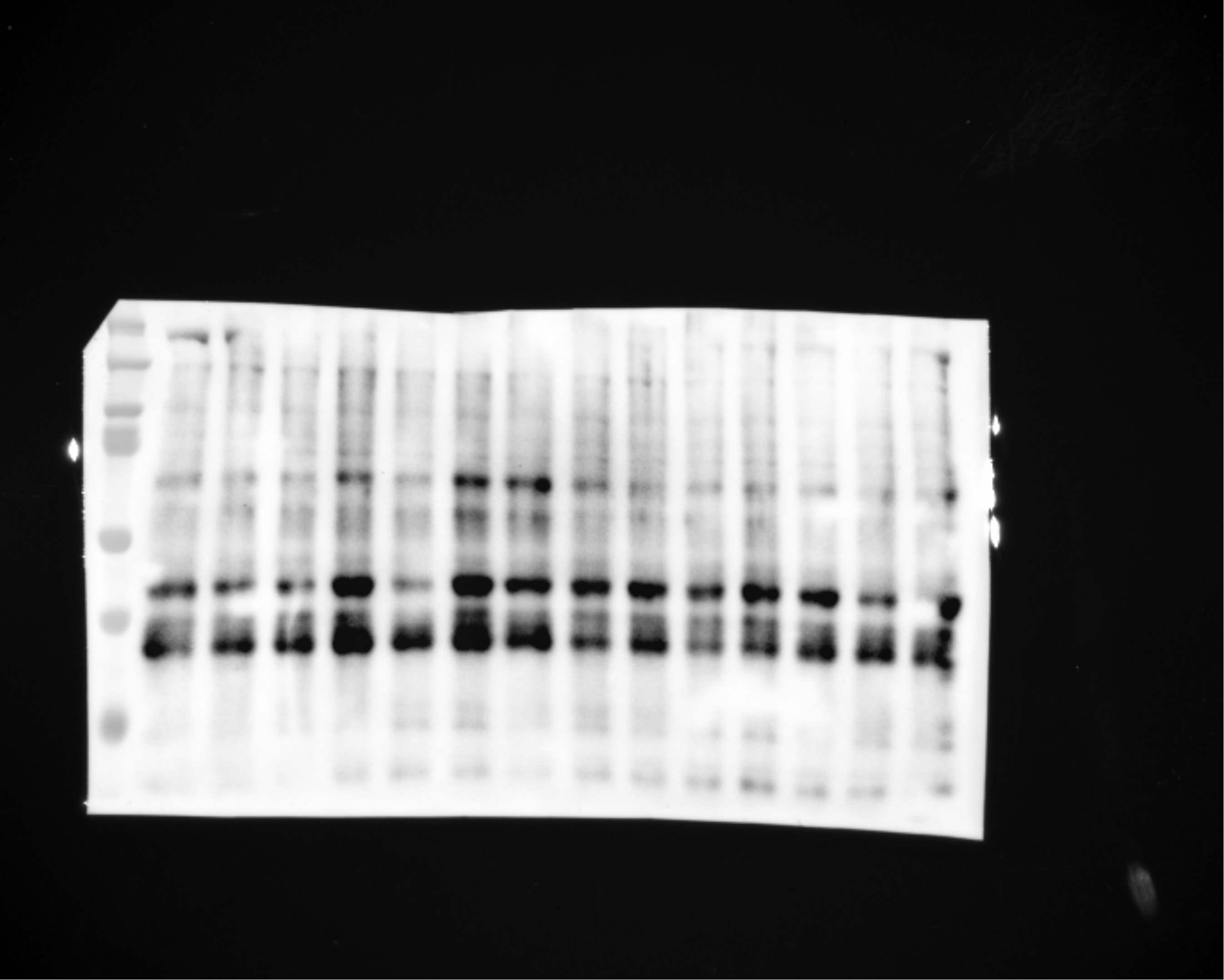

Supplement: Supplementary file 9 — Source data Fig. 6 [file 44321_2025_337_MOESM9_ESM.zip › Figure 6/Fig6F_Western blot/Western Oxidized proteins.tif]

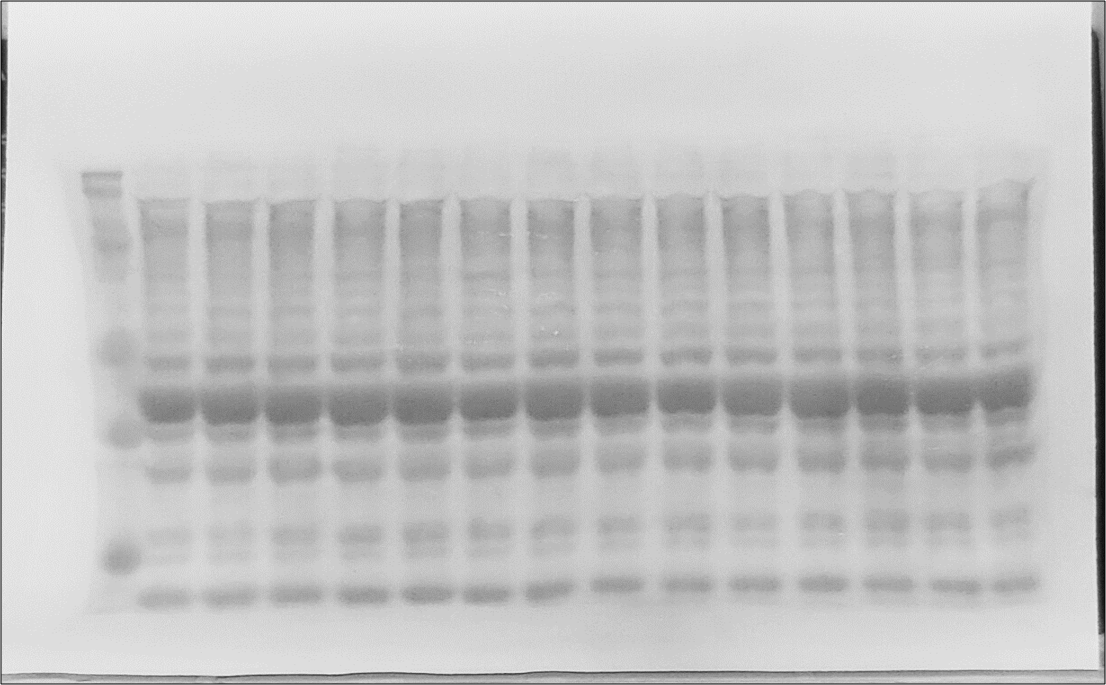

Supplement: Supplementary file 9 — Source data Fig. 6 [file 44321_2025_337_MOESM9_ESM.zip › Figure 6/Fig6F_Western blot/Western Ponceau Stain.tif]

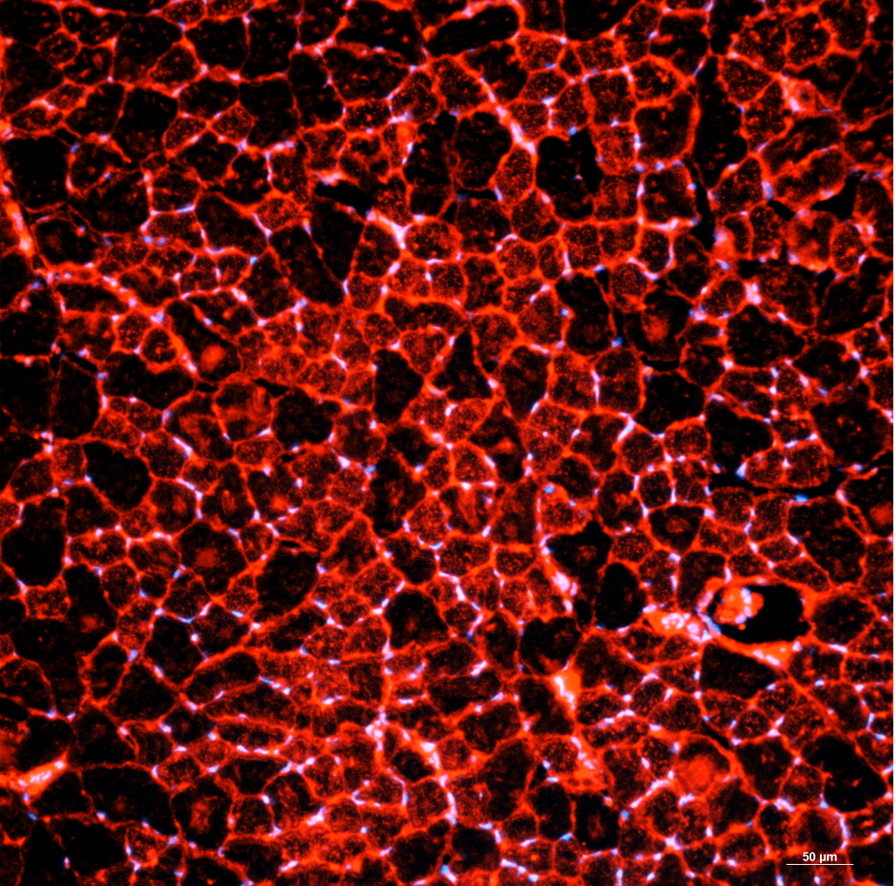

Supplement: Supplementary file 9 — Source data Fig. 6 [file 44321_2025_337_MOESM9_ESM.zip › Figure 6/Fig6H_Plin2 immunstaining/Xbp1 flfl_KPC.tif]

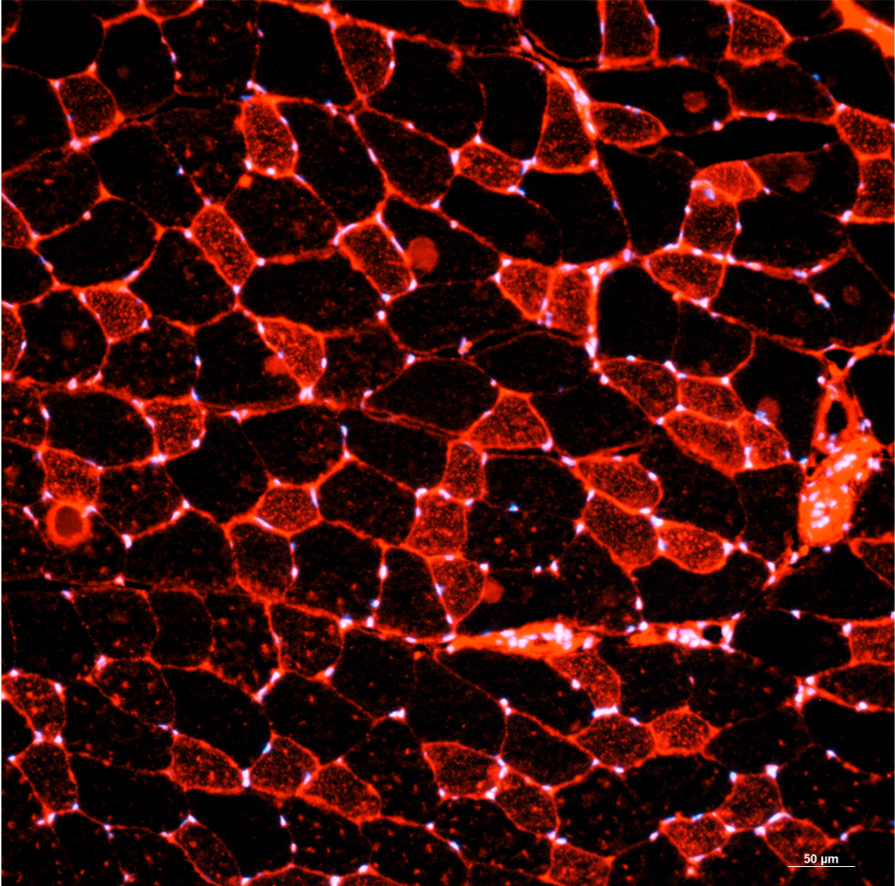

Supplement: Supplementary file 9 — Source data Fig. 6 [file 44321_2025_337_MOESM9_ESM.zip › Figure 6/Fig6H_Plin2 immunstaining/Xbp1 flfl_PBS.tif]

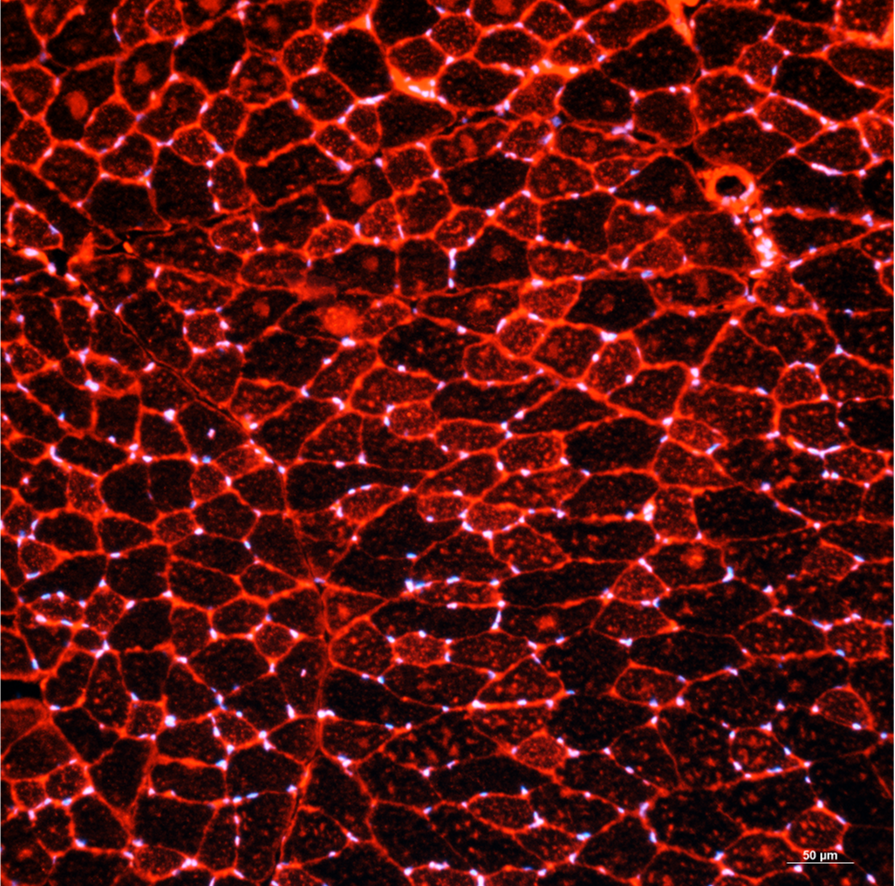

Supplement: Supplementary file 9 — Source data Fig. 6 [file 44321_2025_337_MOESM9_ESM.zip › Figure 6/Fig6H_Plin2 immunstaining/Xbp1 mKO_KPC.tif]

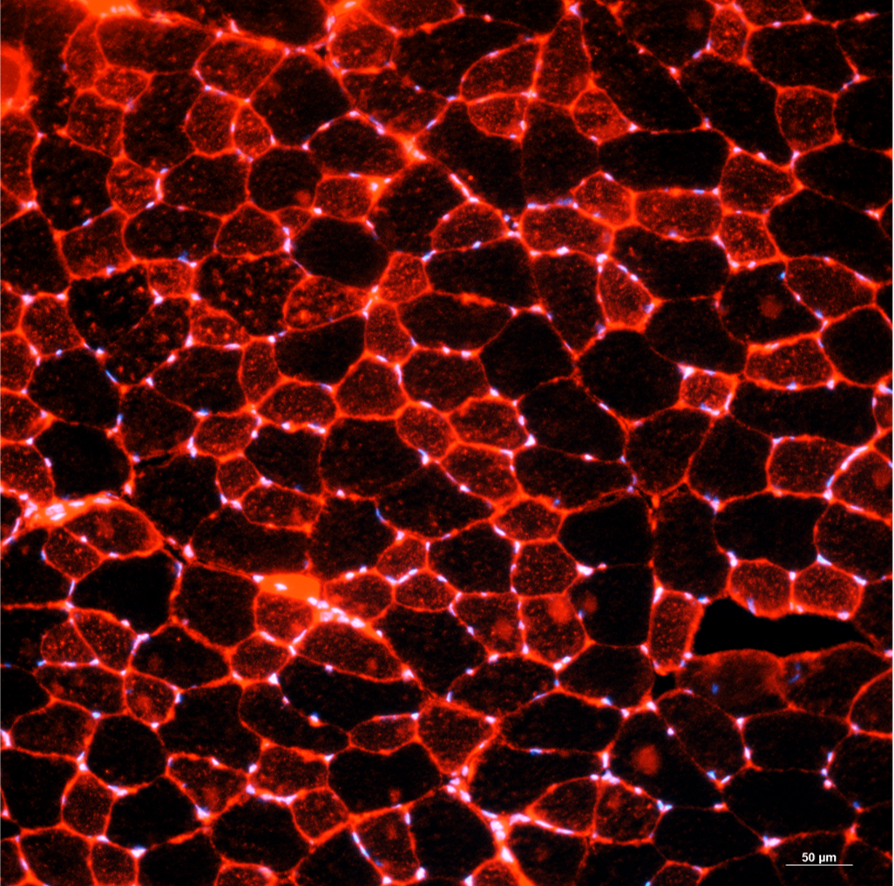

Supplement: Supplementary file 9 — Source data Fig. 6 [file 44321_2025_337_MOESM9_ESM.zip › Figure 6/Fig6H_Plin2 immunstaining/Xbp1 mKO_PBS.tif]

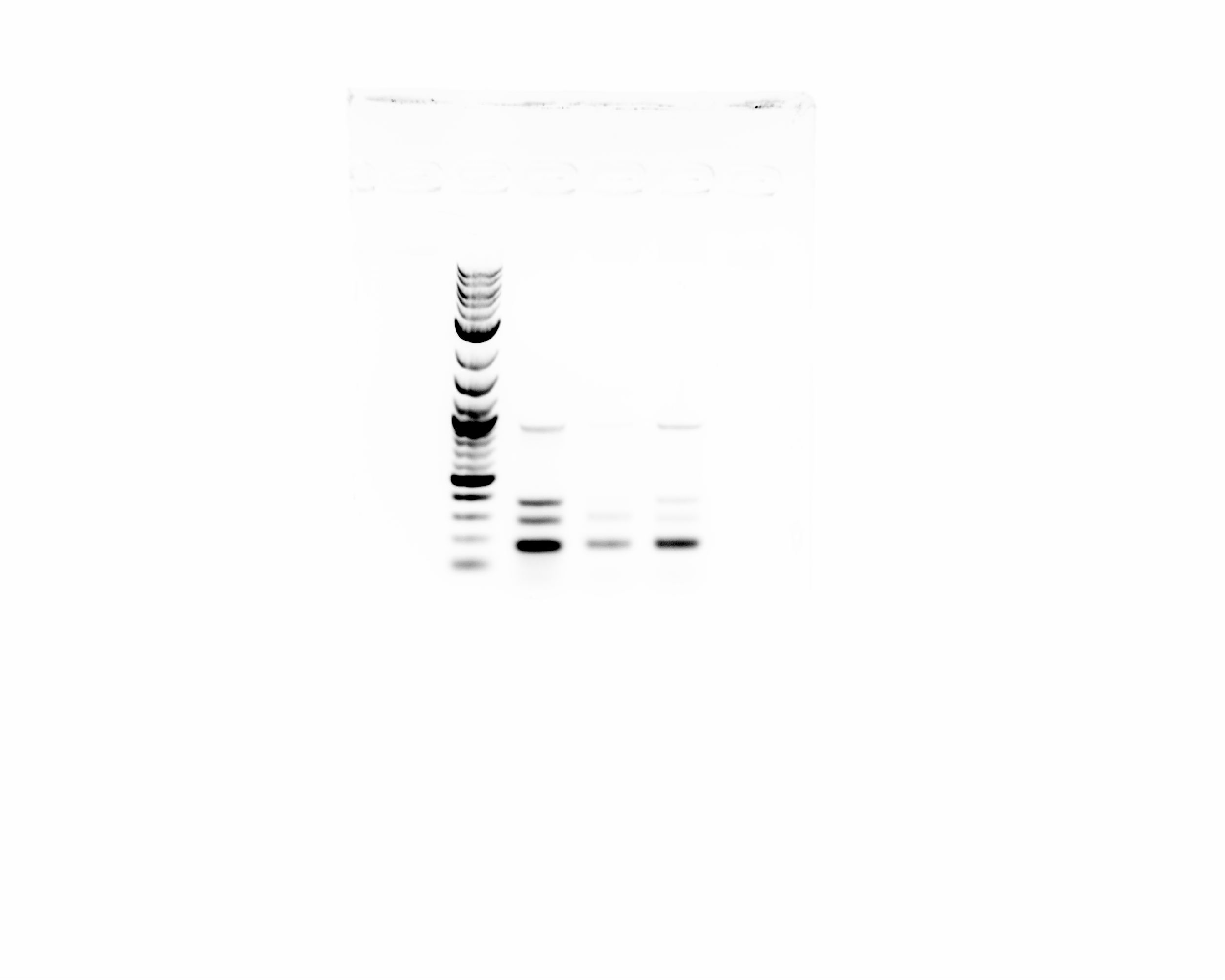

Supplement: Supplementary file 10 — Source data Fig. 7 [file 44321_2025_337_MOESM10_ESM.zip › Figure 7/Fig7C_E_G_I_ChIP-PCR/Fig7C_E_G_I_ChIP-PCR_Agarose gel images/Fig7C_E/Agarose ChIP_Atg5_Myotubes.tif]

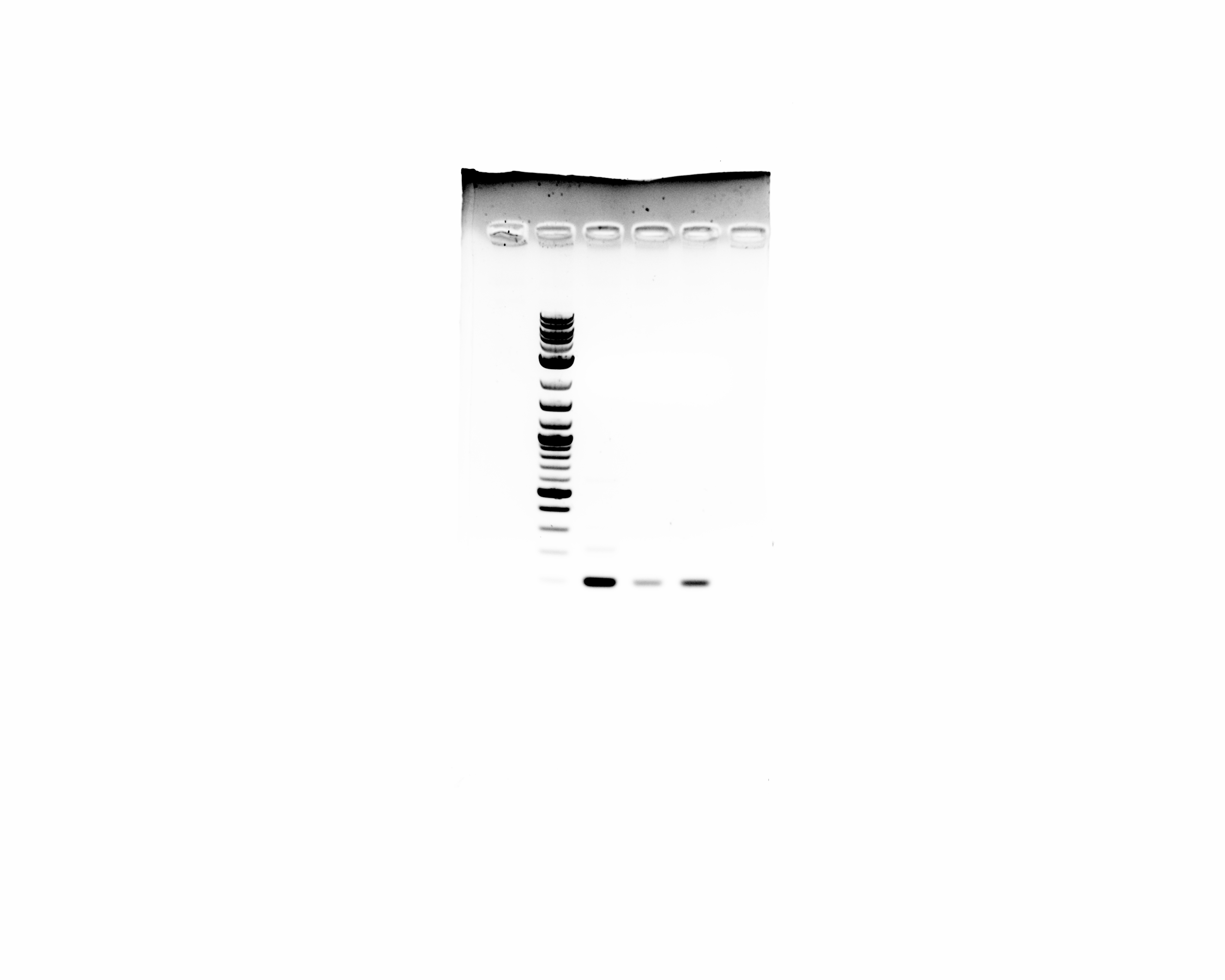

Supplement: Supplementary file 10 — Source data Fig. 7 [file 44321_2025_337_MOESM10_ESM.zip › Figure 7/Fig7C_E_G_I_ChIP-PCR/Fig7C_E_G_I_ChIP-PCR_Agarose gel images/Fig7C_E/Agarose ChIP_Dnajb9_Myotubes.tif]

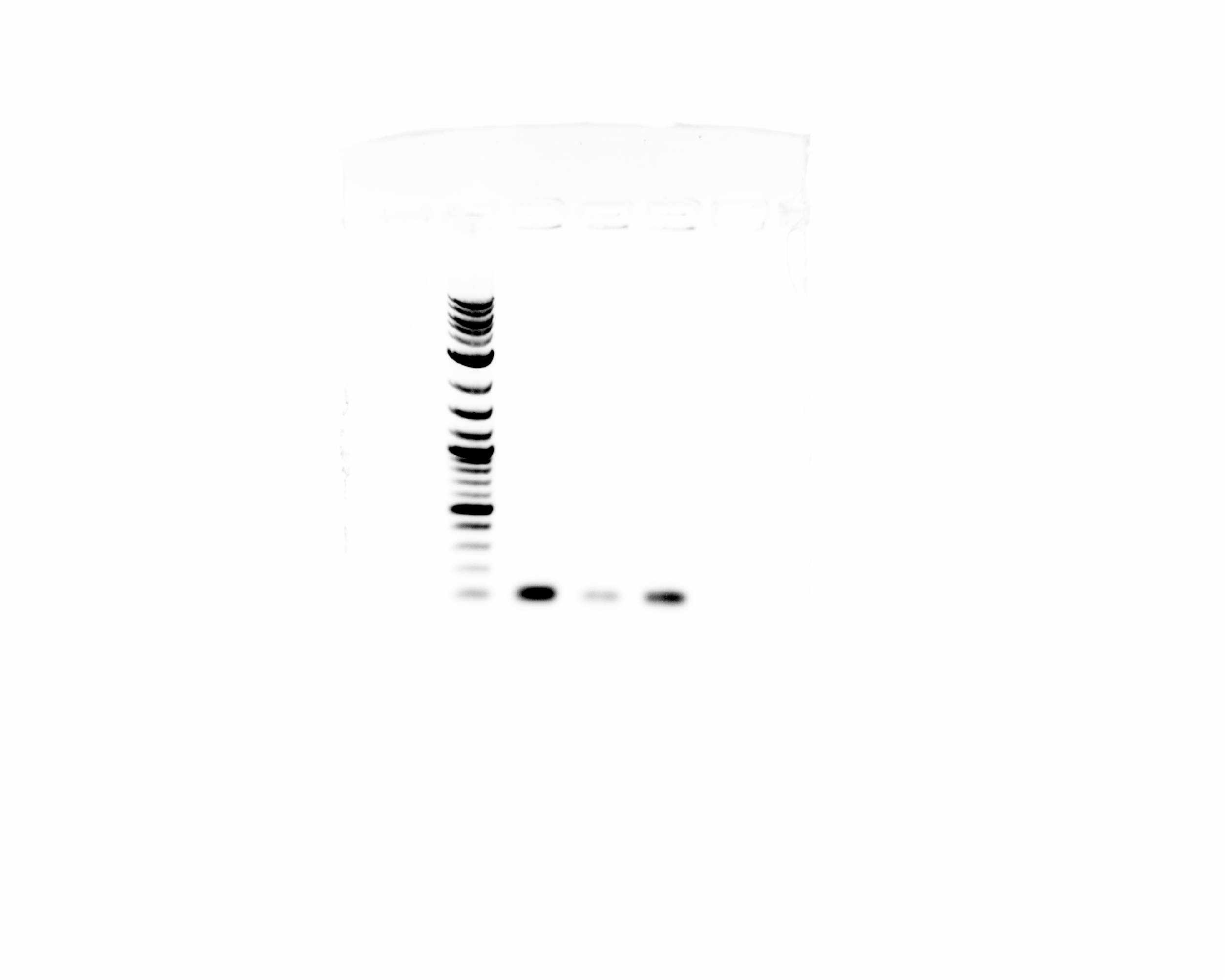

Supplement: Supplementary file 10 — Source data Fig. 7 [file 44321_2025_337_MOESM10_ESM.zip › Figure 7/Fig7C_E_G_I_ChIP-PCR/Fig7C_E_G_I_ChIP-PCR_Agarose gel images/Fig7C_E/Agarose ChIP_Fbxo32_Myotubes.tif]

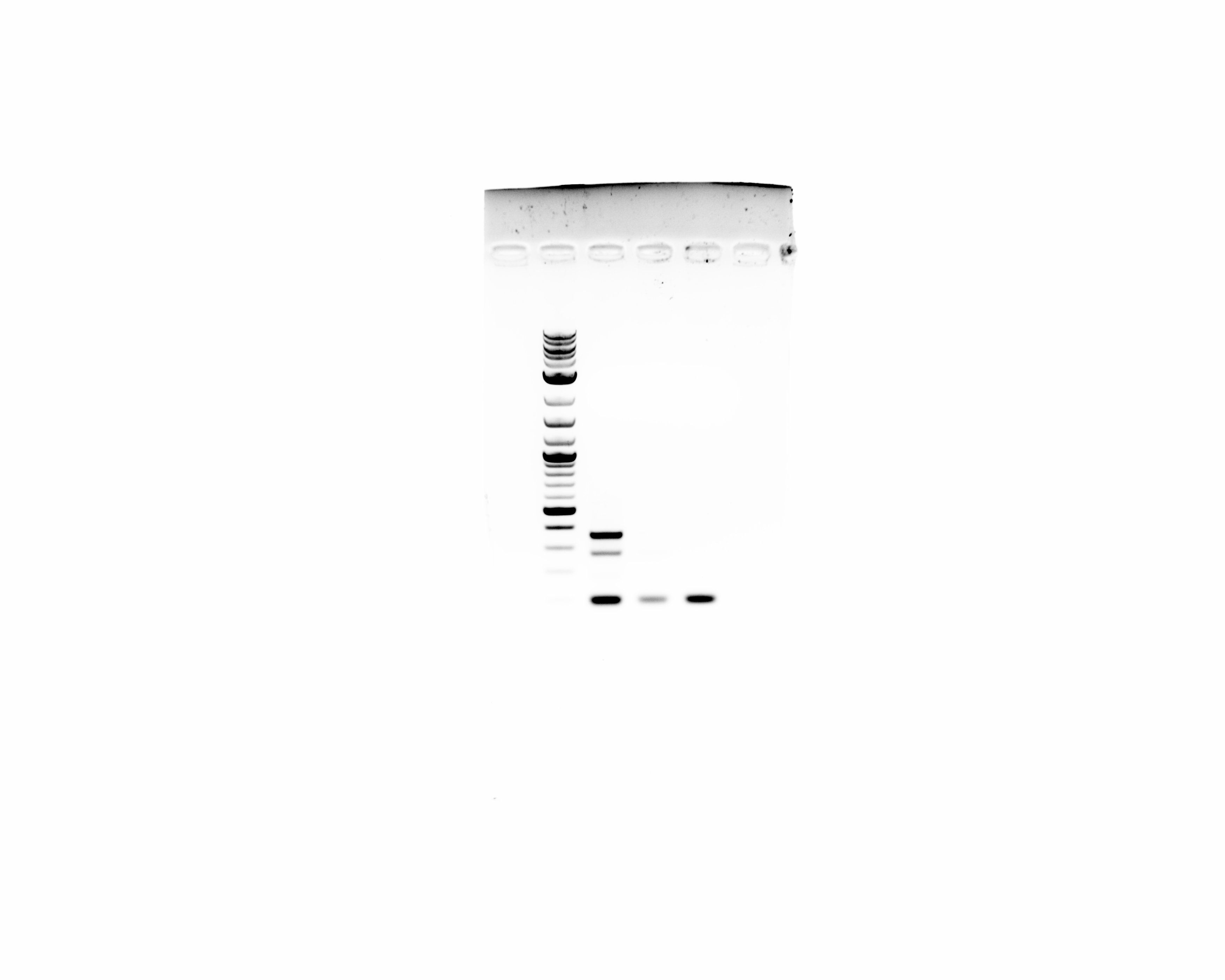

Supplement: Supplementary file 10 — Source data Fig. 7 [file 44321_2025_337_MOESM10_ESM.zip › Figure 7/Fig7C_E_G_I_ChIP-PCR/Fig7C_E_G_I_ChIP-PCR_Agarose gel images/Fig7C_E/Agarose ChIP_Hspa5_Myotubes.tif]

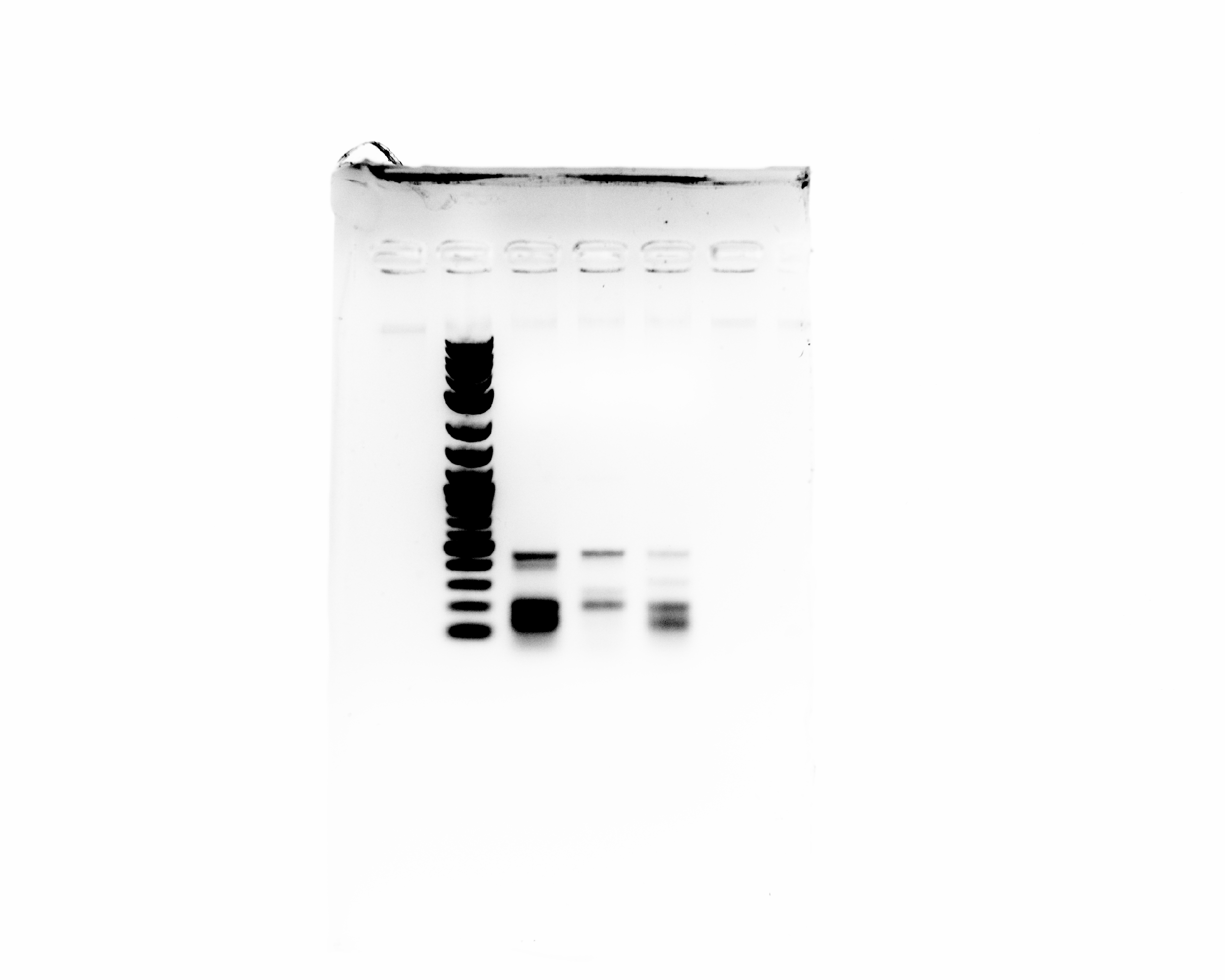

Supplement: Supplementary file 10 — Source data Fig. 7 [file 44321_2025_337_MOESM10_ESM.zip › Figure 7/Fig7C_E_G_I_ChIP-PCR/Fig7C_E_G_I_ChIP-PCR_Agarose gel images/Fig7C_E/Agarose ChIP_Map1lc3b_Myotubes.tif]

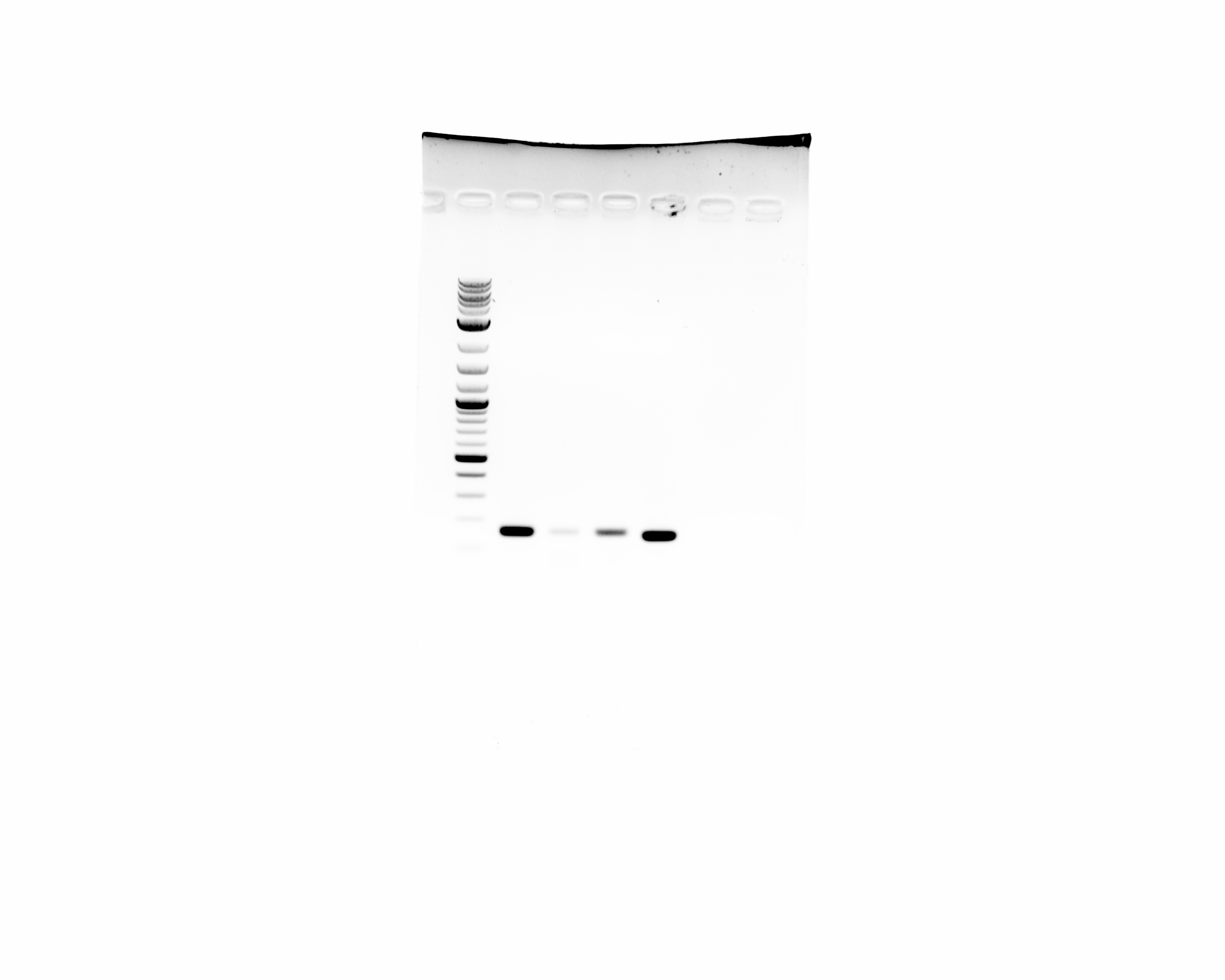

Supplement: Supplementary file 10 — Source data Fig. 7 [file 44321_2025_337_MOESM10_ESM.zip › Figure 7/Fig7C_E_G_I_ChIP-PCR/Fig7C_E_G_I_ChIP-PCR_Agarose gel images/Fig7C_E/Agarose ChIP_Rpl30_Myotubes.tif]

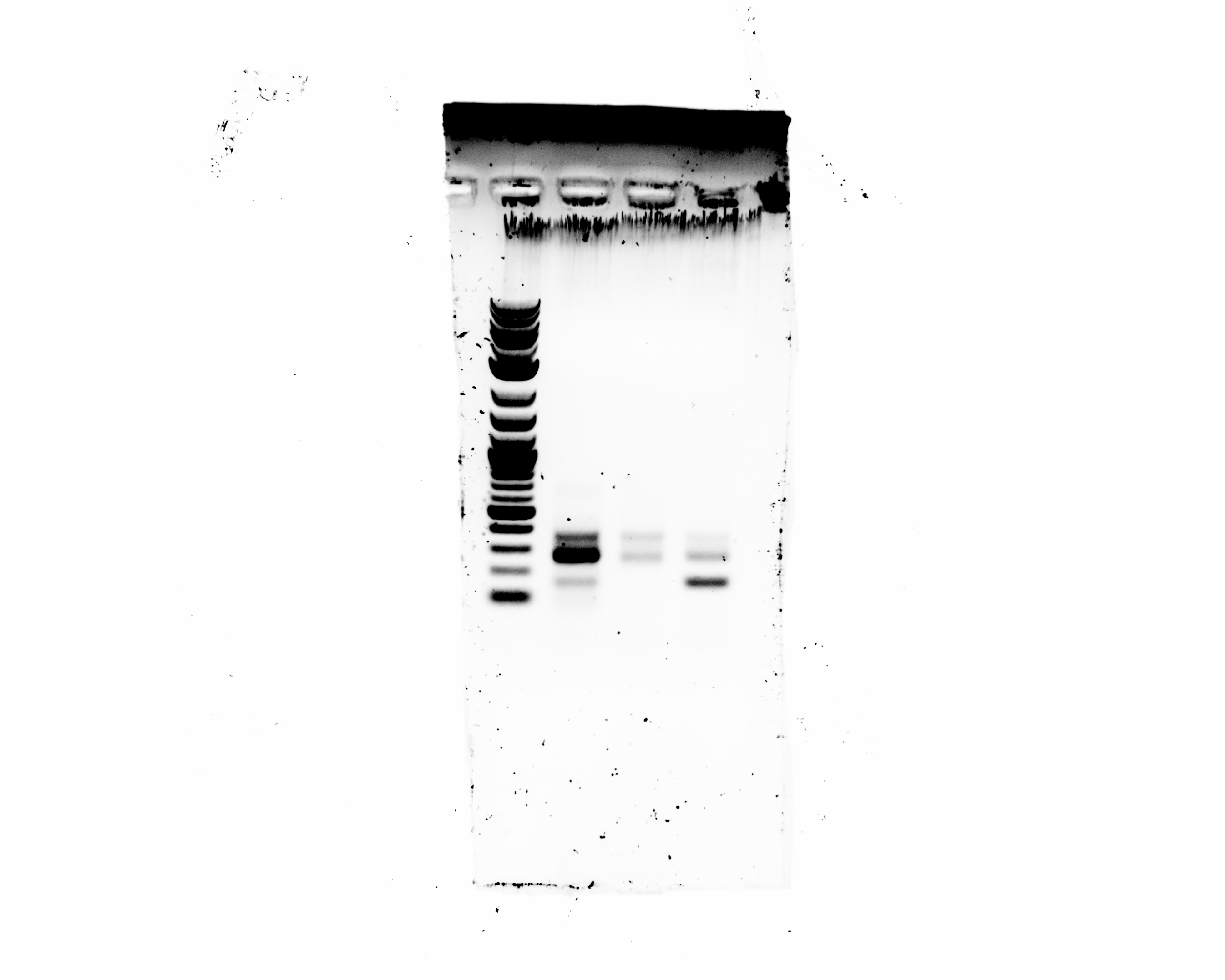

Supplement: Supplementary file 10 — Source data Fig. 7 [file 44321_2025_337_MOESM10_ESM.zip › Figure 7/Fig7C_E_G_I_ChIP-PCR/Fig7C_E_G_I_ChIP-PCR_Agarose gel images/Fig7C_E/Agarose ChIP_Xbp1_Myotubes.tif]

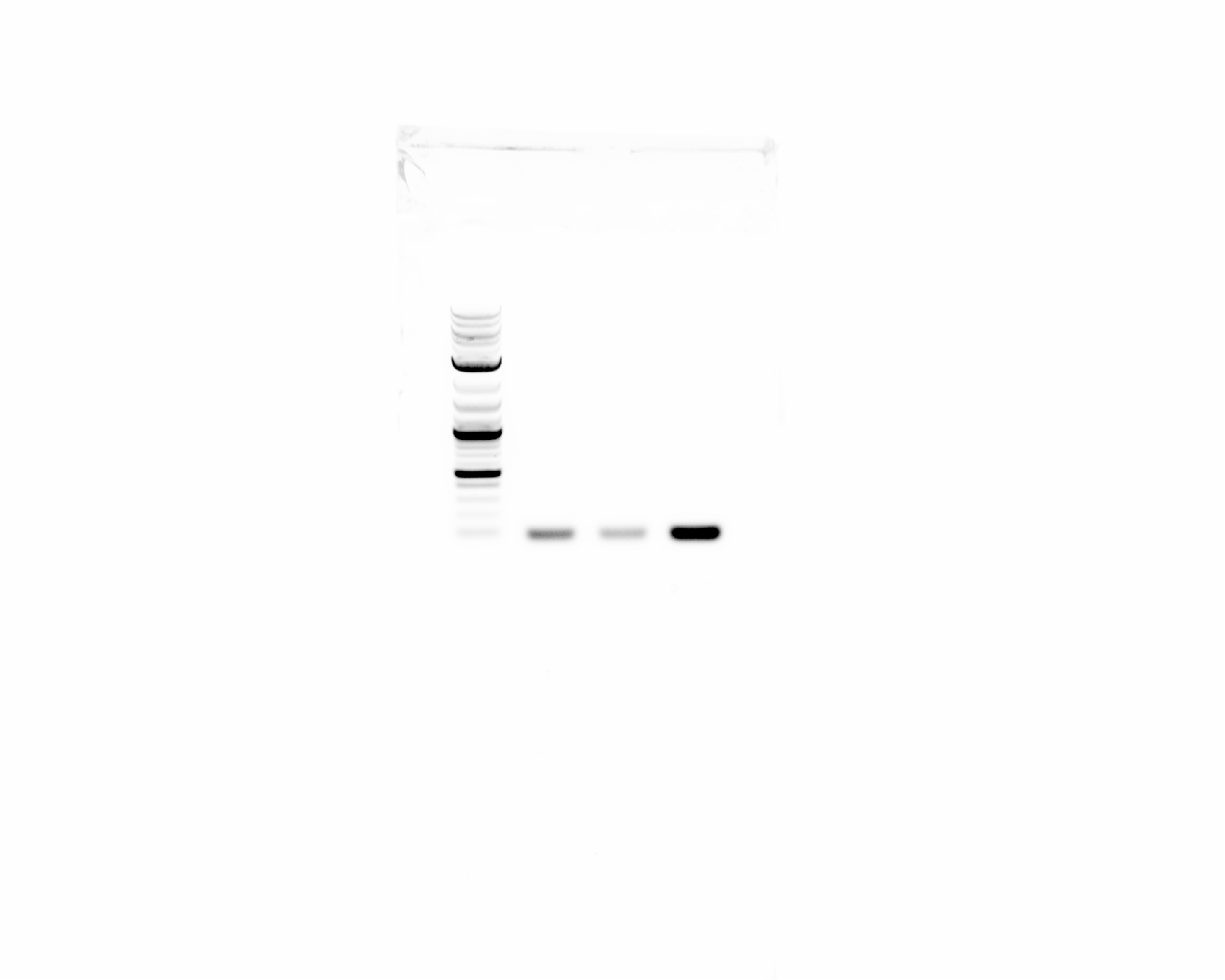

Supplement: Supplementary file 10 — Source data Fig. 7 [file 44321_2025_337_MOESM10_ESM.zip › Figure 7/Fig7C_E_G_I_ChIP-PCR/Fig7C_E_G_I_ChIP-PCR_Agarose gel images/Fig7G_I/Agarose ChIP_Dnajb9_Muscle tissue.tif]

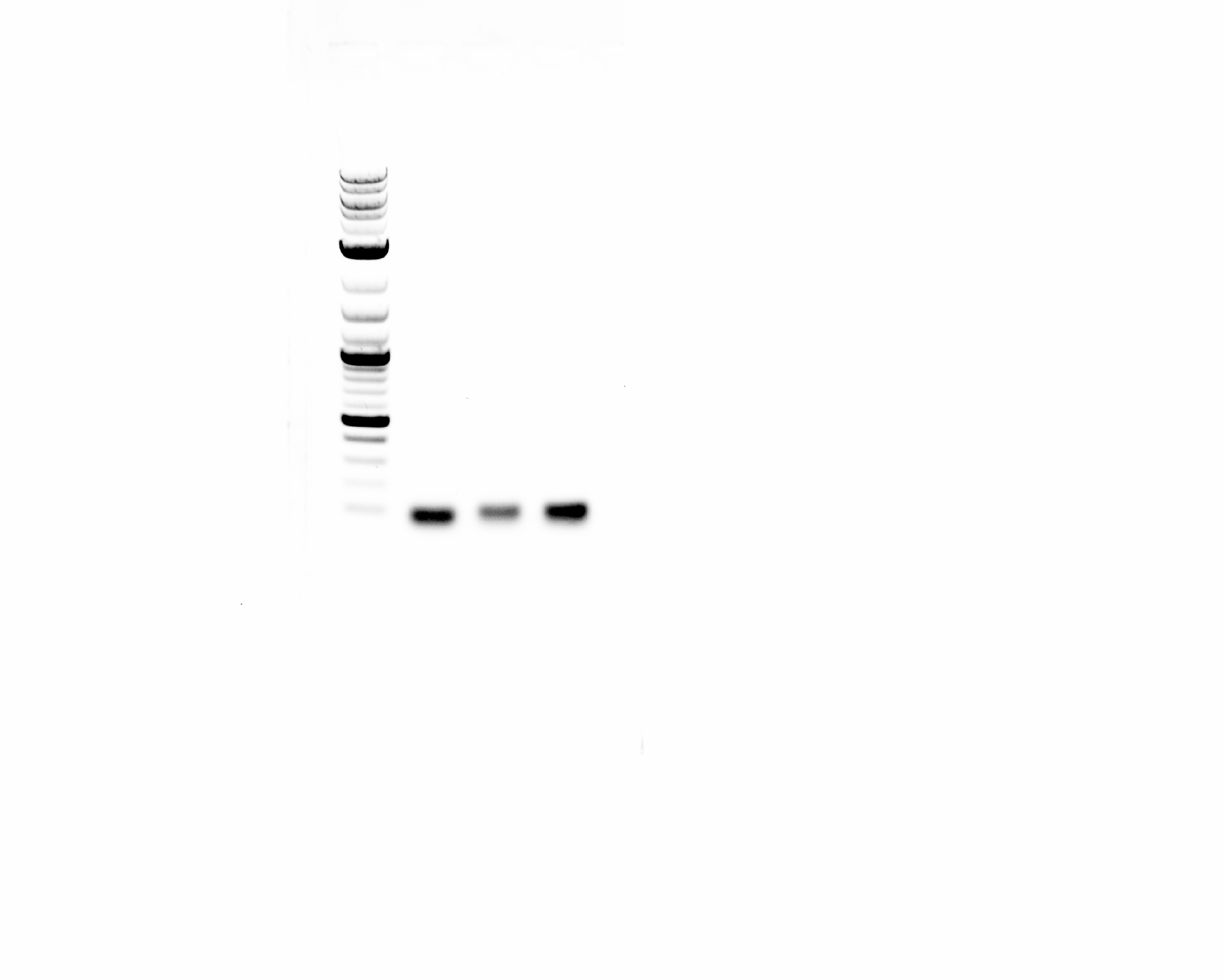

Supplement: Supplementary file 10 — Source data Fig. 7 [file 44321_2025_337_MOESM10_ESM.zip › Figure 7/Fig7C_E_G_I_ChIP-PCR/Fig7C_E_G_I_ChIP-PCR_Agarose gel images/Fig7G_I/Agarose ChIP_Fbxo32_Muscle tissue.tif]

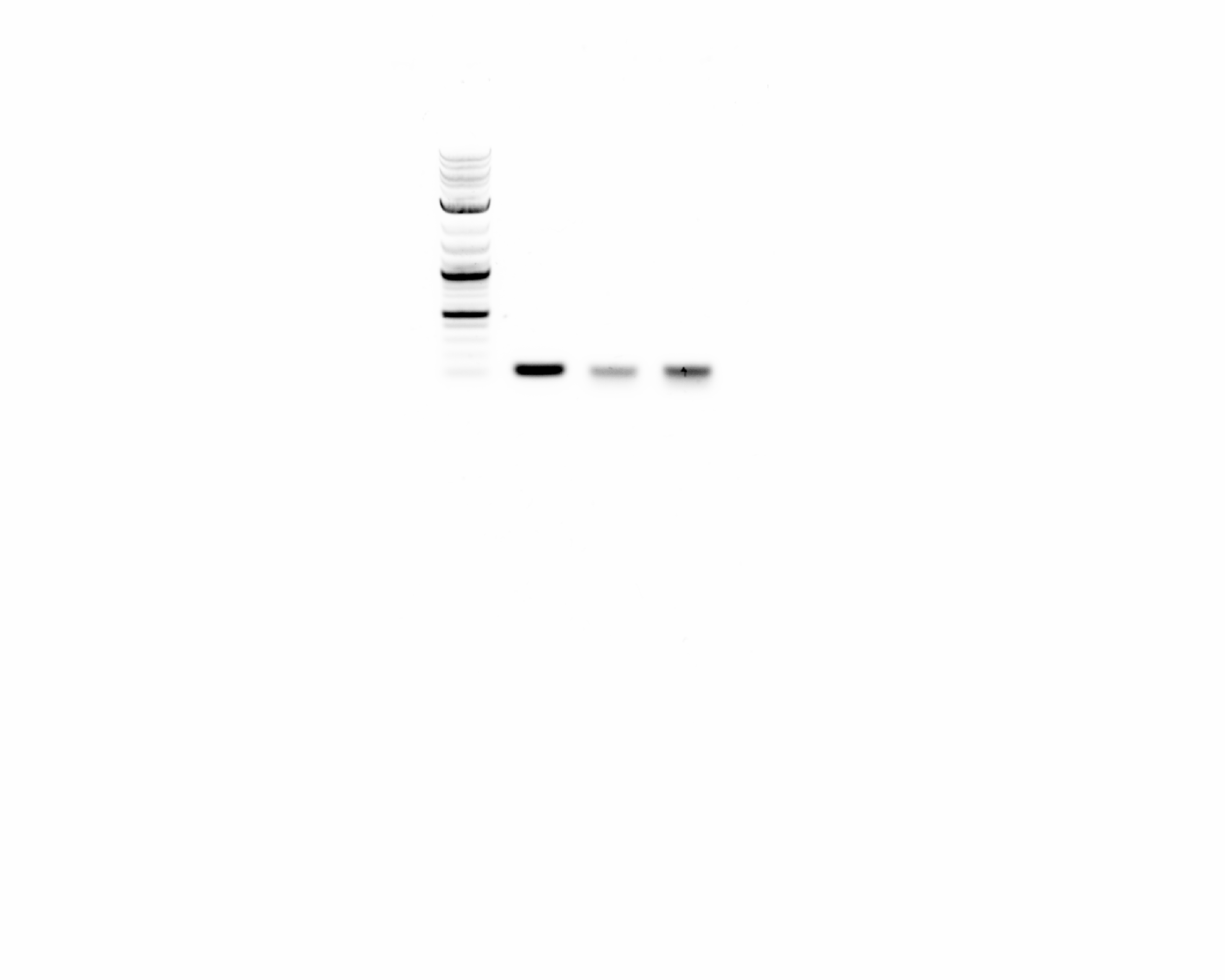

Supplement: Supplementary file 10 — Source data Fig. 7 [file 44321_2025_337_MOESM10_ESM.zip › Figure 7/Fig7C_E_G_I_ChIP-PCR/Fig7C_E_G_I_ChIP-PCR_Agarose gel images/Fig7G_I/Agarose ChIP_Il6_Muscle tissue.tif]

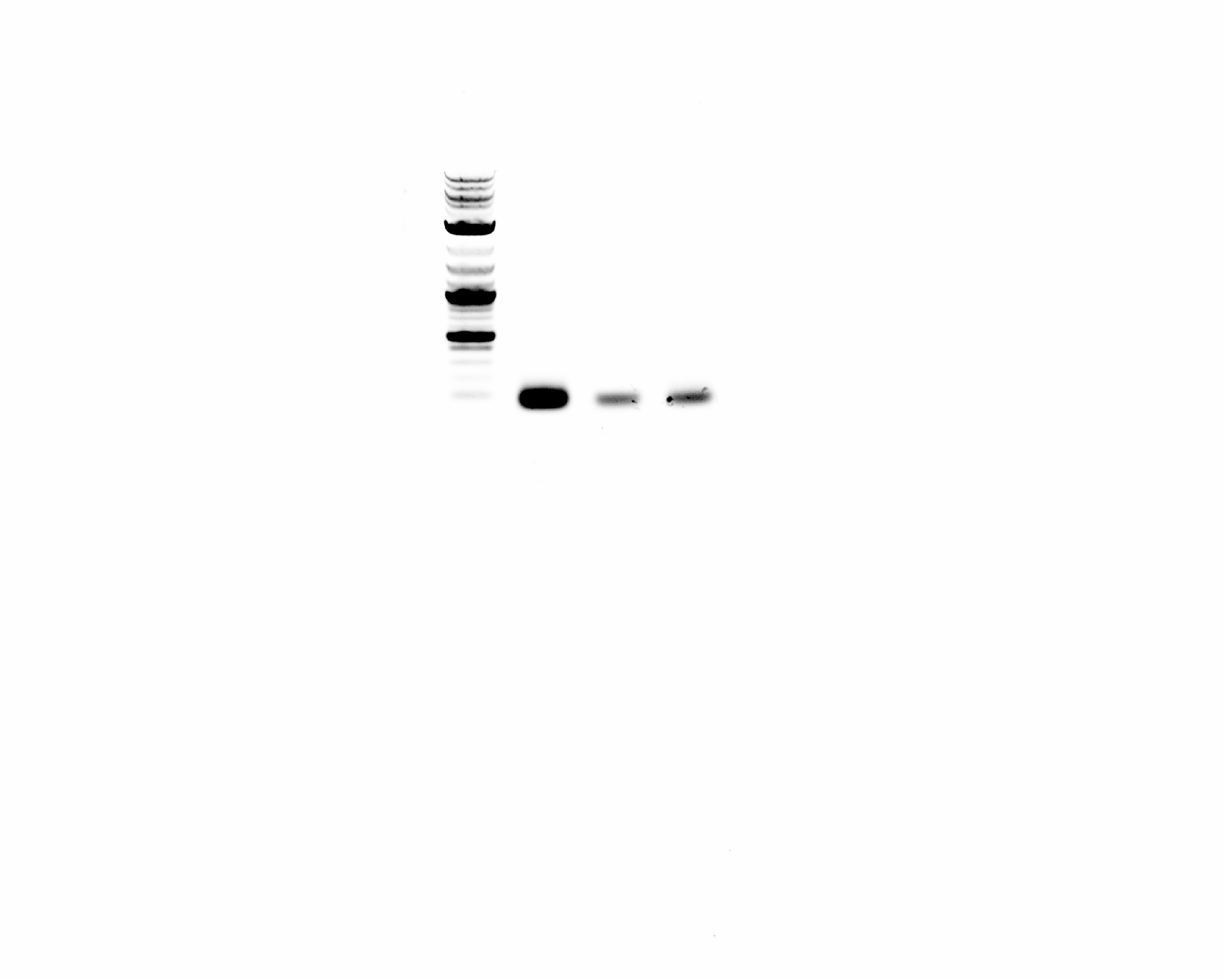

Supplement: Supplementary file 10 — Source data Fig. 7 [file 44321_2025_337_MOESM10_ESM.zip › Figure 7/Fig7C_E_G_I_ChIP-PCR/Fig7C_E_G_I_ChIP-PCR_Agarose gel images/Fig7G_I/Agarose ChIP_Pdk4_Muscle tissue.tif]

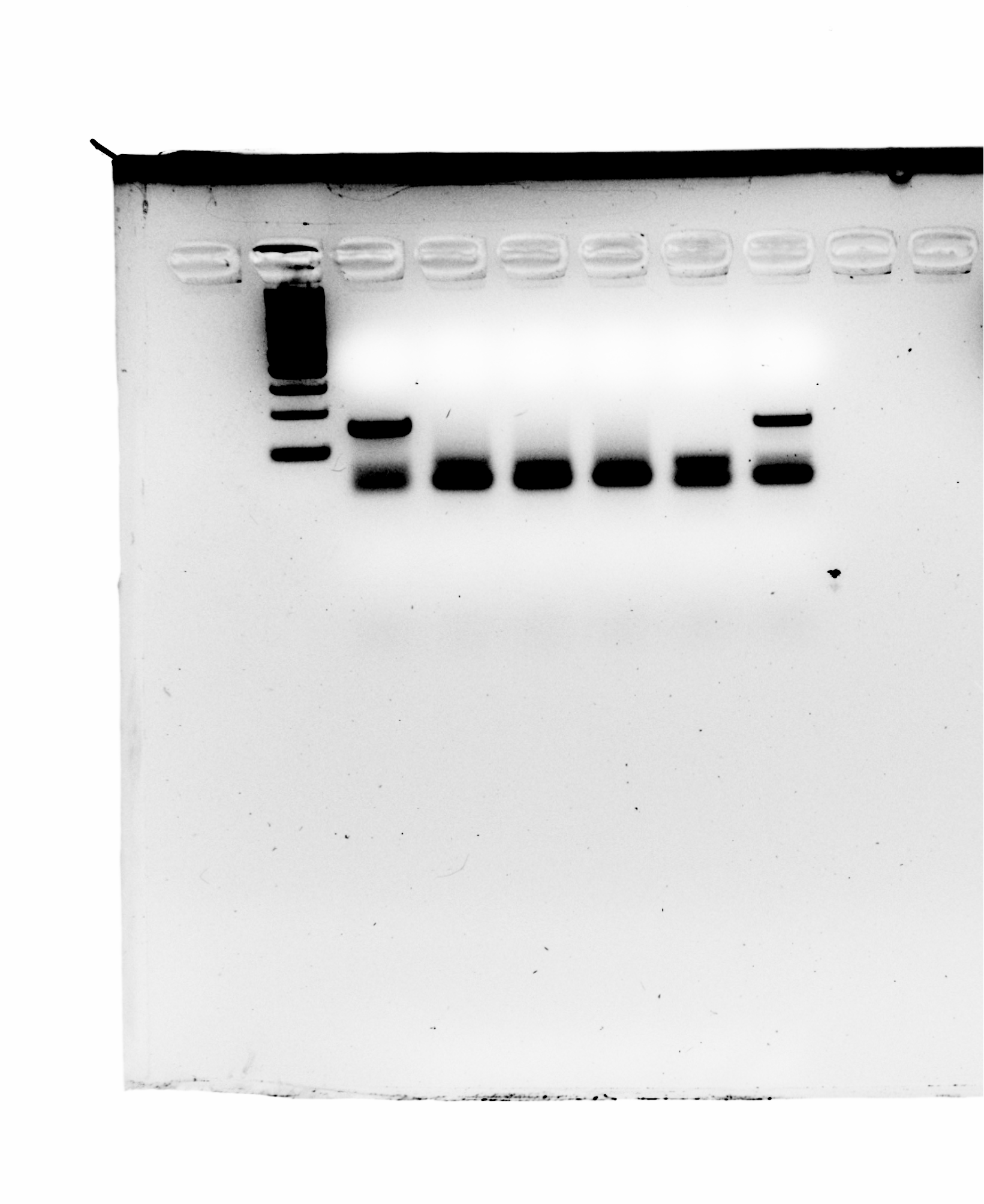

Supplement: Supplementary file 10 — Source data Fig. 7 [file 44321_2025_337_MOESM10_ESM.zip › Figure 7/Fig7C_E_G_I_ChIP-PCR/Fig7C_E_G_I_ChIP-PCR_Agarose gel images/Fig7G_I/Agarose ChIP_Rpl30_Muscle tissue.tif]

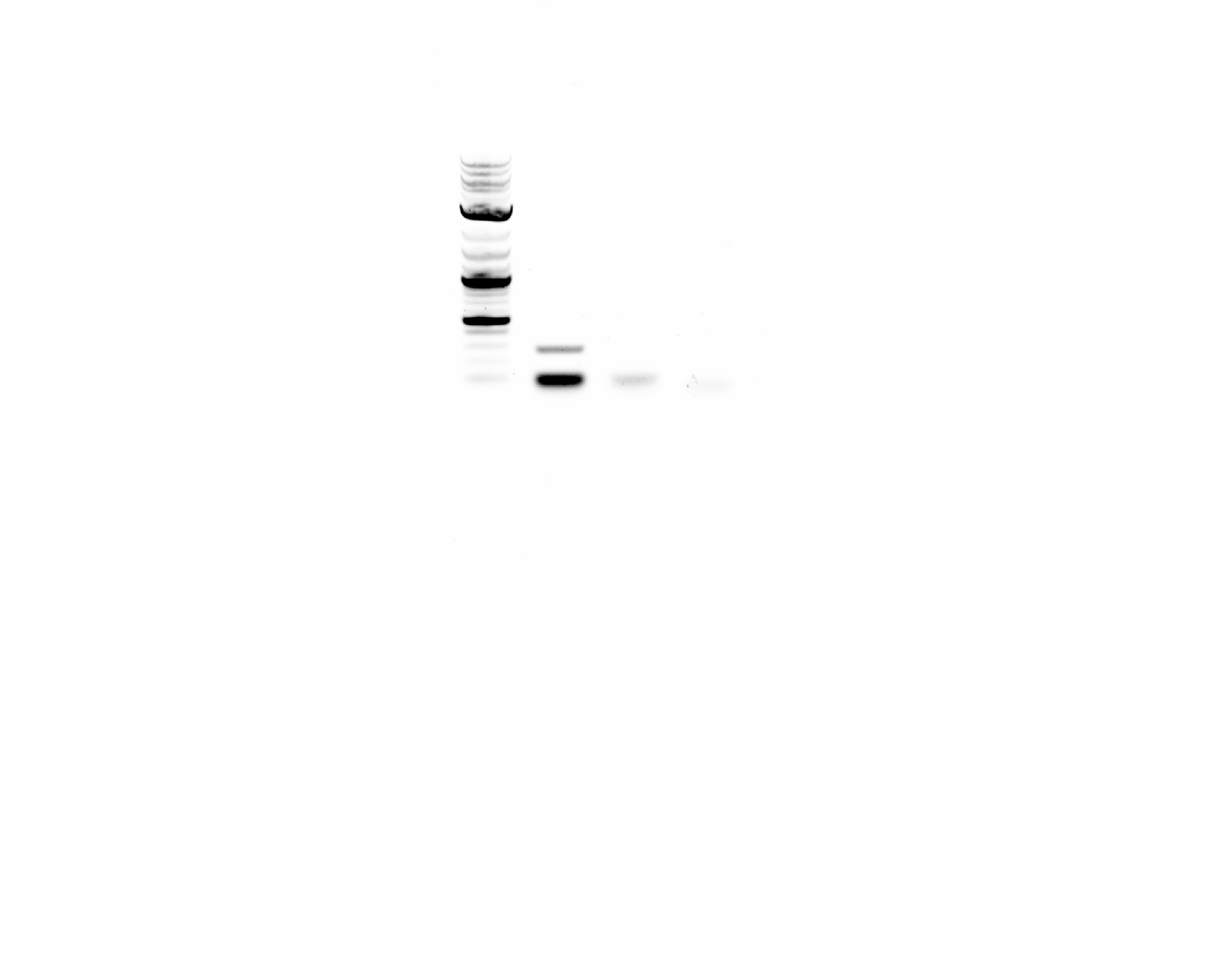

Supplement: Supplementary file 10 — Source data Fig. 7 [file 44321_2025_337_MOESM10_ESM.zip › Figure 7/Fig7C_E_G_I_ChIP-PCR/Fig7C_E_G_I_ChIP-PCR_Agarose gel images/Fig7G_I/Agarose ChIP_Stat3_Muscle tissue.tif]

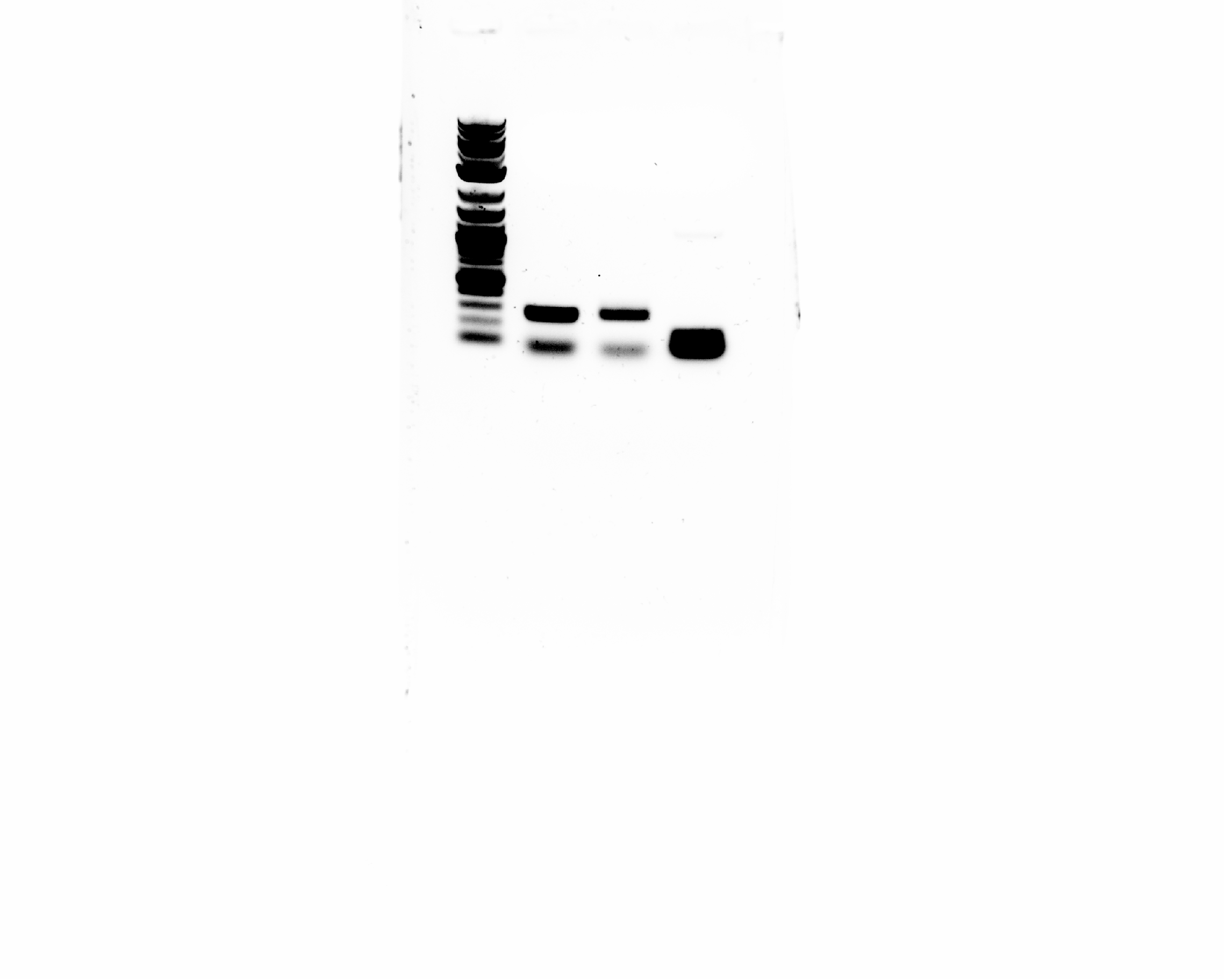

Supplement: Supplementary file 10 — Source data Fig. 7 [file 44321_2025_337_MOESM10_ESM.zip › Figure 7/Fig7C_E_G_I_ChIP-PCR/Fig7C_E_G_I_ChIP-PCR_Agarose gel images/Fig7G_I/Agarose ChIP_Xbp1_Muscle tissue.tif]

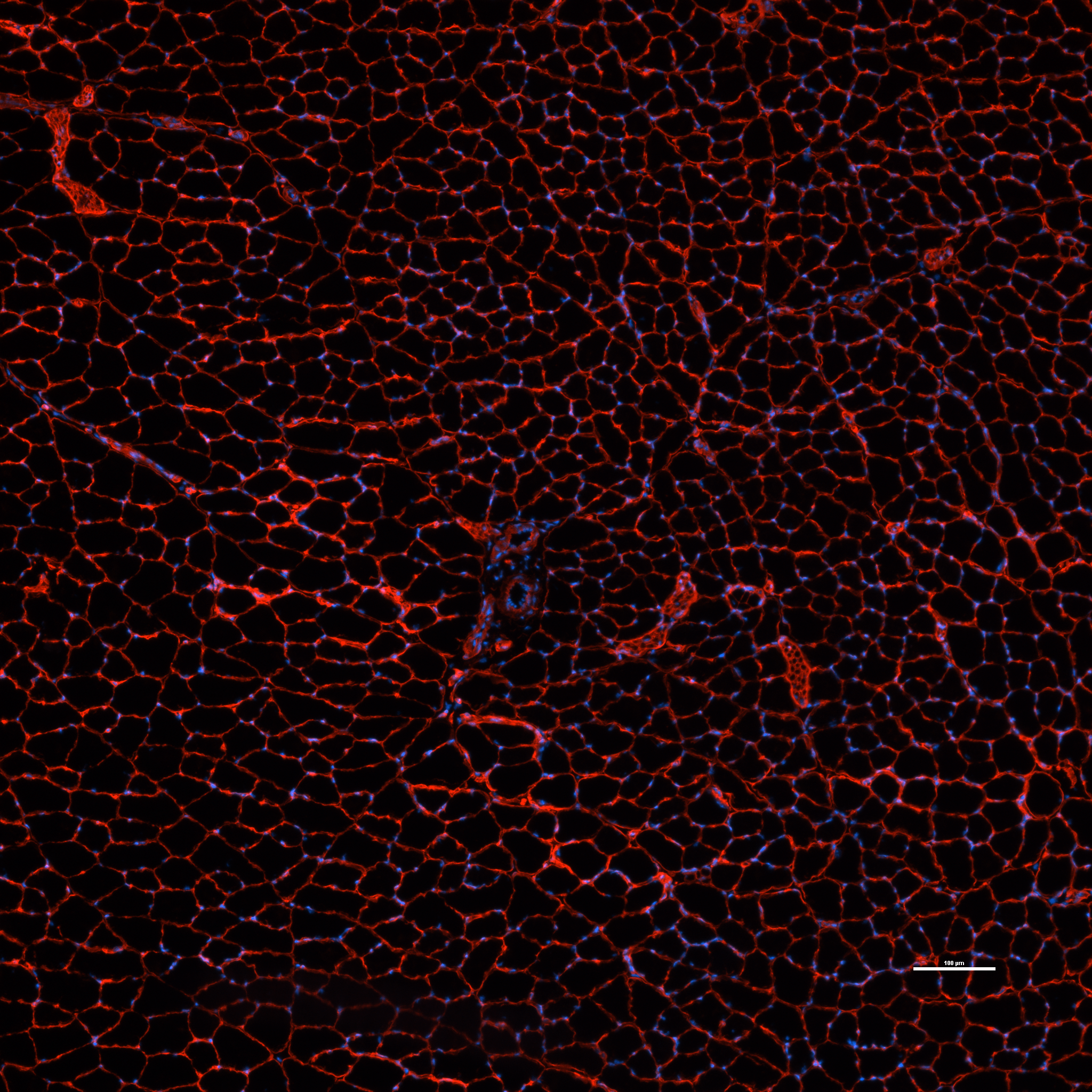

Supplement: Supplementary file 11 — Source data Fig. 8 [file 44321_2025_337_MOESM11_ESM.zip › Figure 8/Fig8E_Laminin-DAPI-staining_Representative images/TA muscle_ Vehicle_KPC.tif]

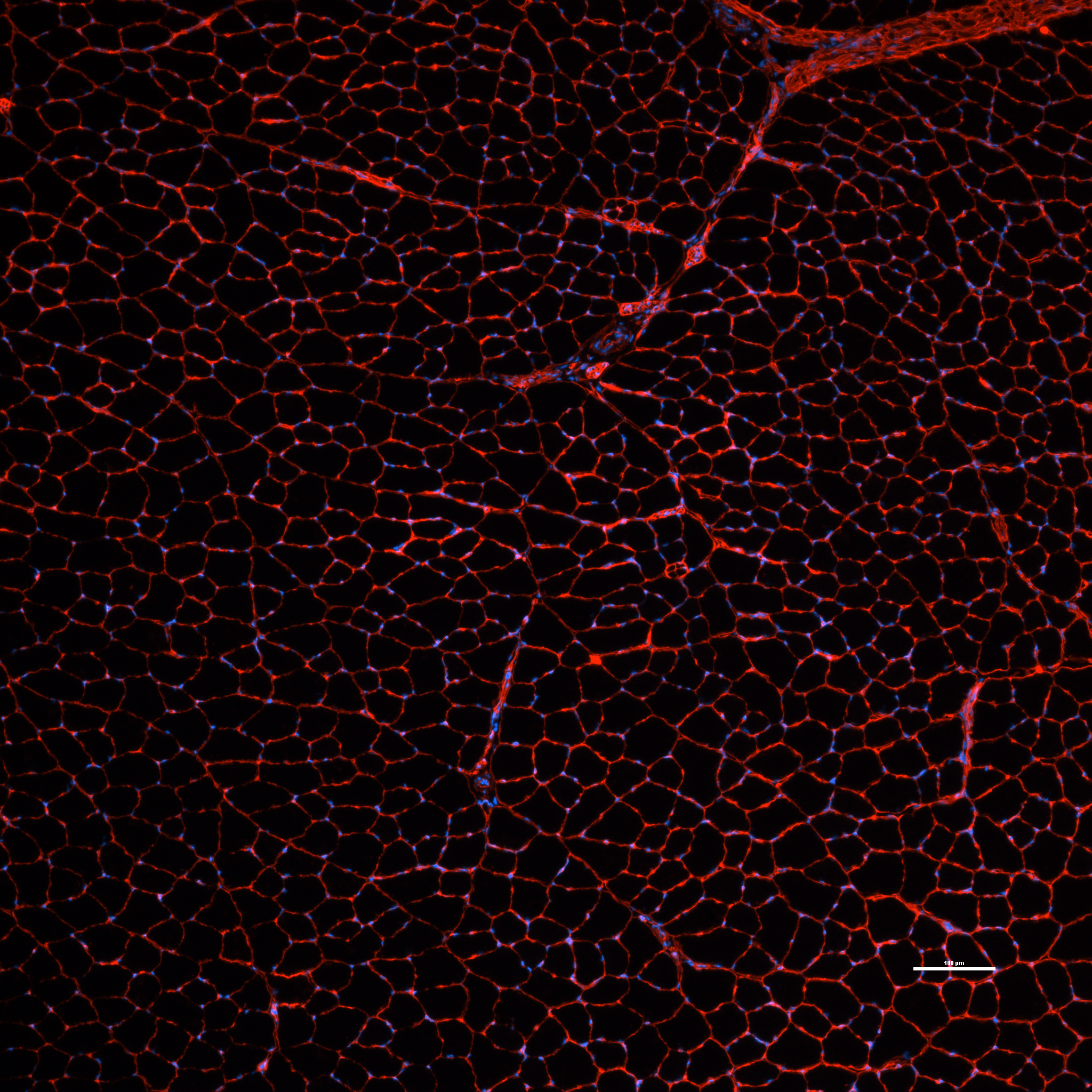

Supplement: Supplementary file 11 — Source data Fig. 8 [file 44321_2025_337_MOESM11_ESM.zip › Figure 8/Fig8E_Laminin-DAPI-staining_Representative images/TA muscle_4u8c_KPC.tif]

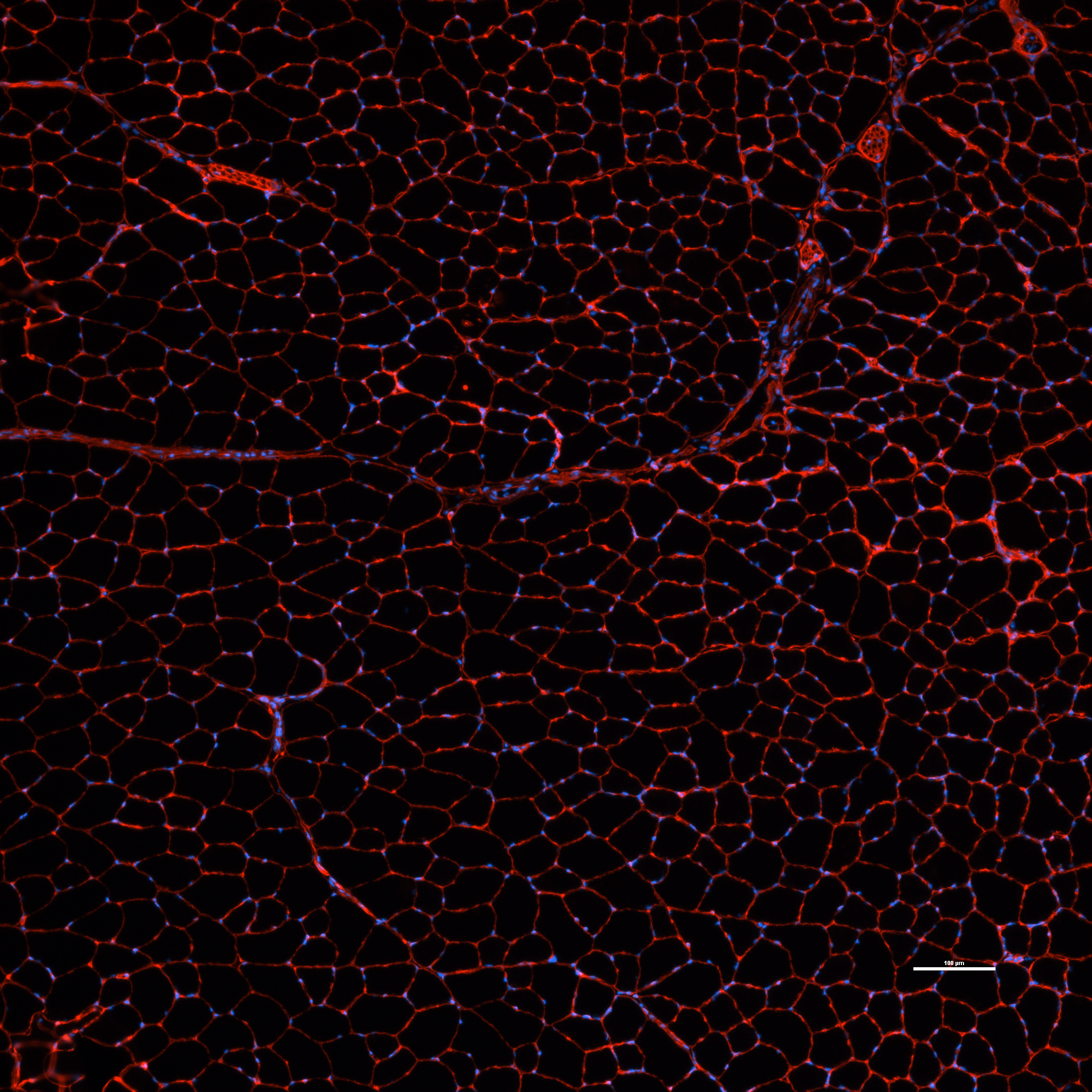

Supplement: Supplementary file 11 — Source data Fig. 8 [file 44321_2025_337_MOESM11_ESM.zip › Figure 8/Fig8E_Laminin-DAPI-staining_Representative images/TA muscle_PBS.tif]

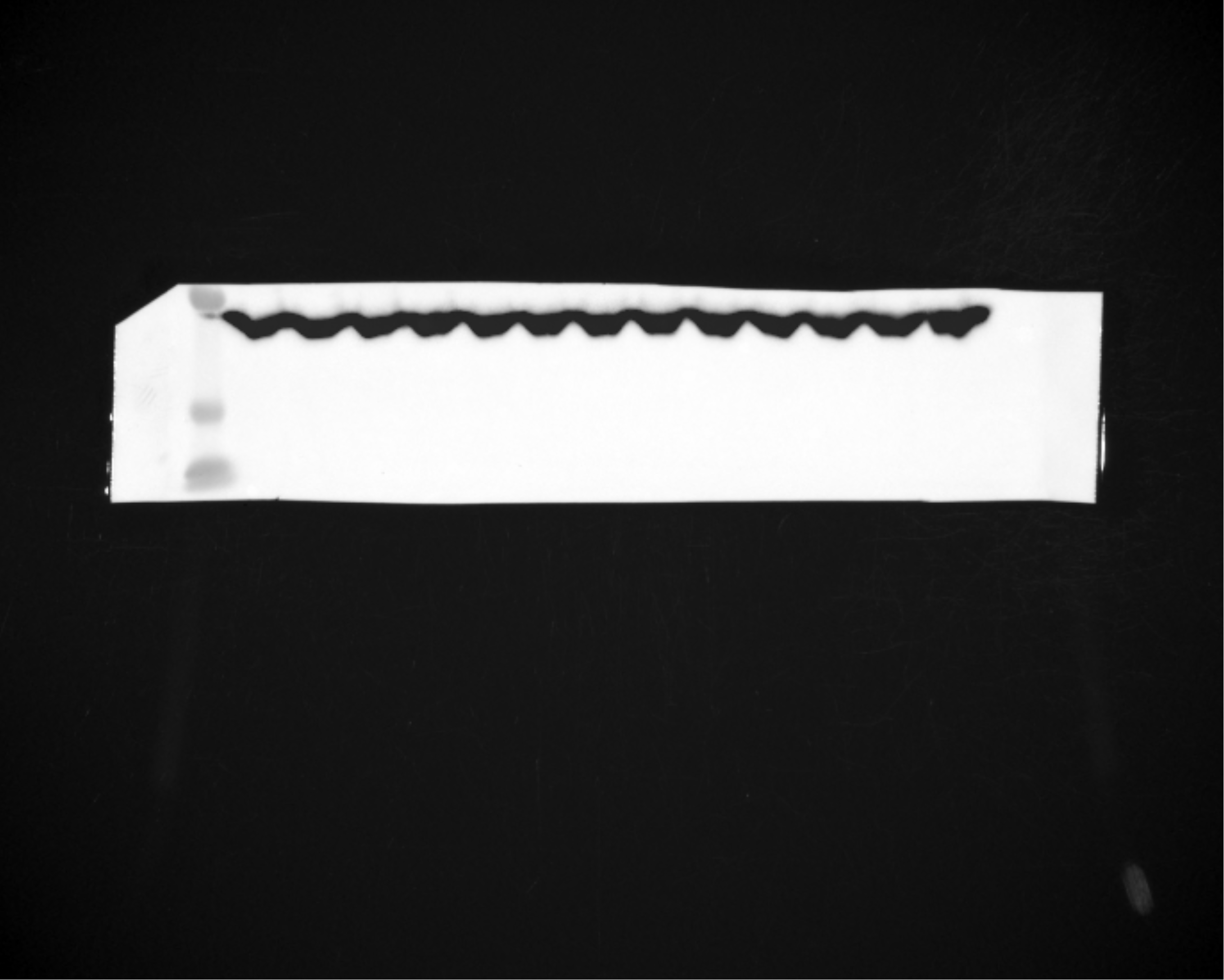

Supplement: Supplementary file 11 — Source data Fig. 8 [file 44321_2025_337_MOESM11_ESM.zip › Figure 8/Fig8I-L_Western blot/Fig8I_Western blot images/Western GAPDH.tif]

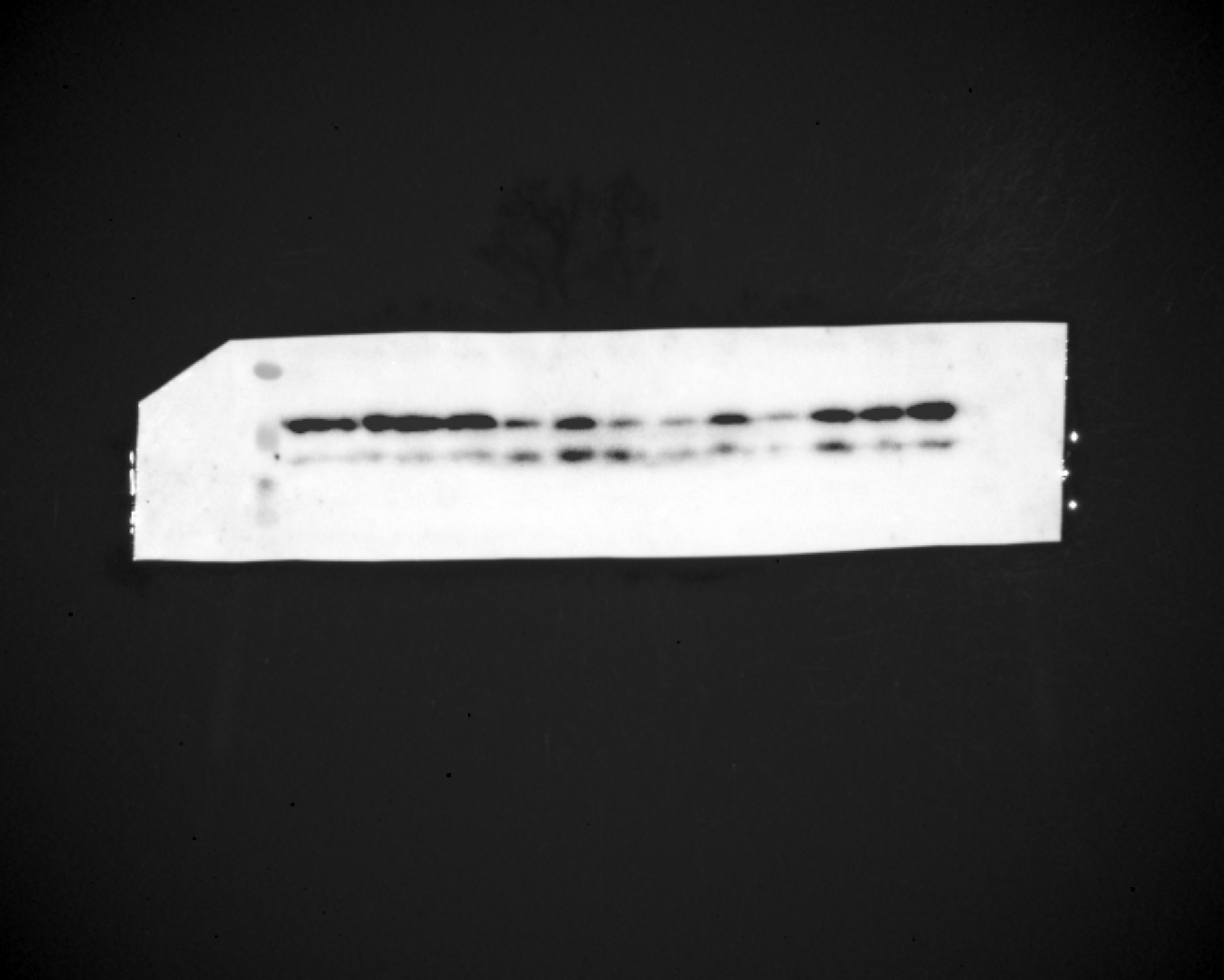

Supplement: Supplementary file 11 — Source data Fig. 8 [file 44321_2025_337_MOESM11_ESM.zip › Figure 8/Fig8I-L_Western blot/Fig8I_Western blot images/Western LC3B.tif]

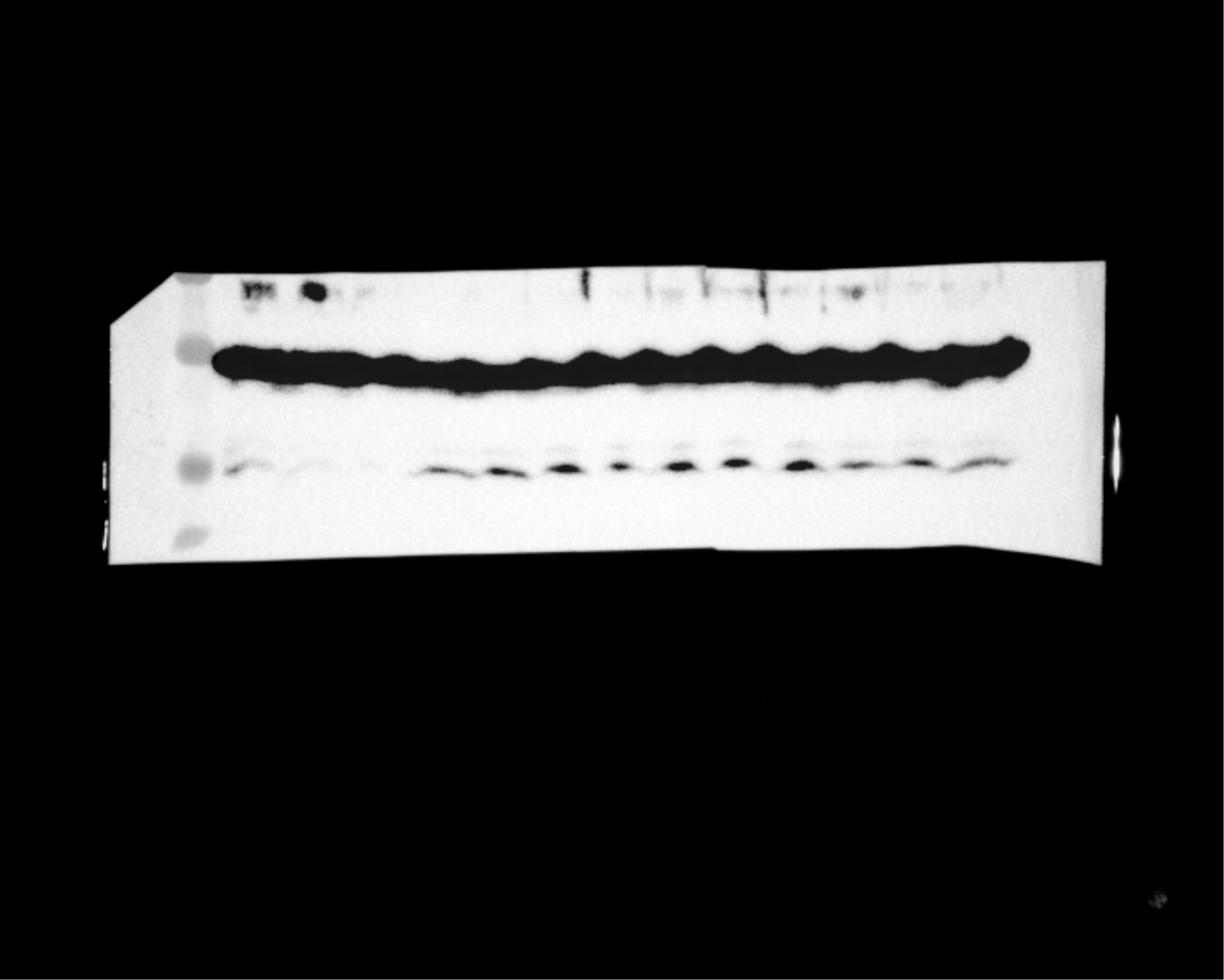

Supplement: Supplementary file 11 — Source data Fig. 8 [file 44321_2025_337_MOESM11_ESM.zip › Figure 8/Fig8I-L_Western blot/Fig8I_Western blot images/Western MAFbx.tif]

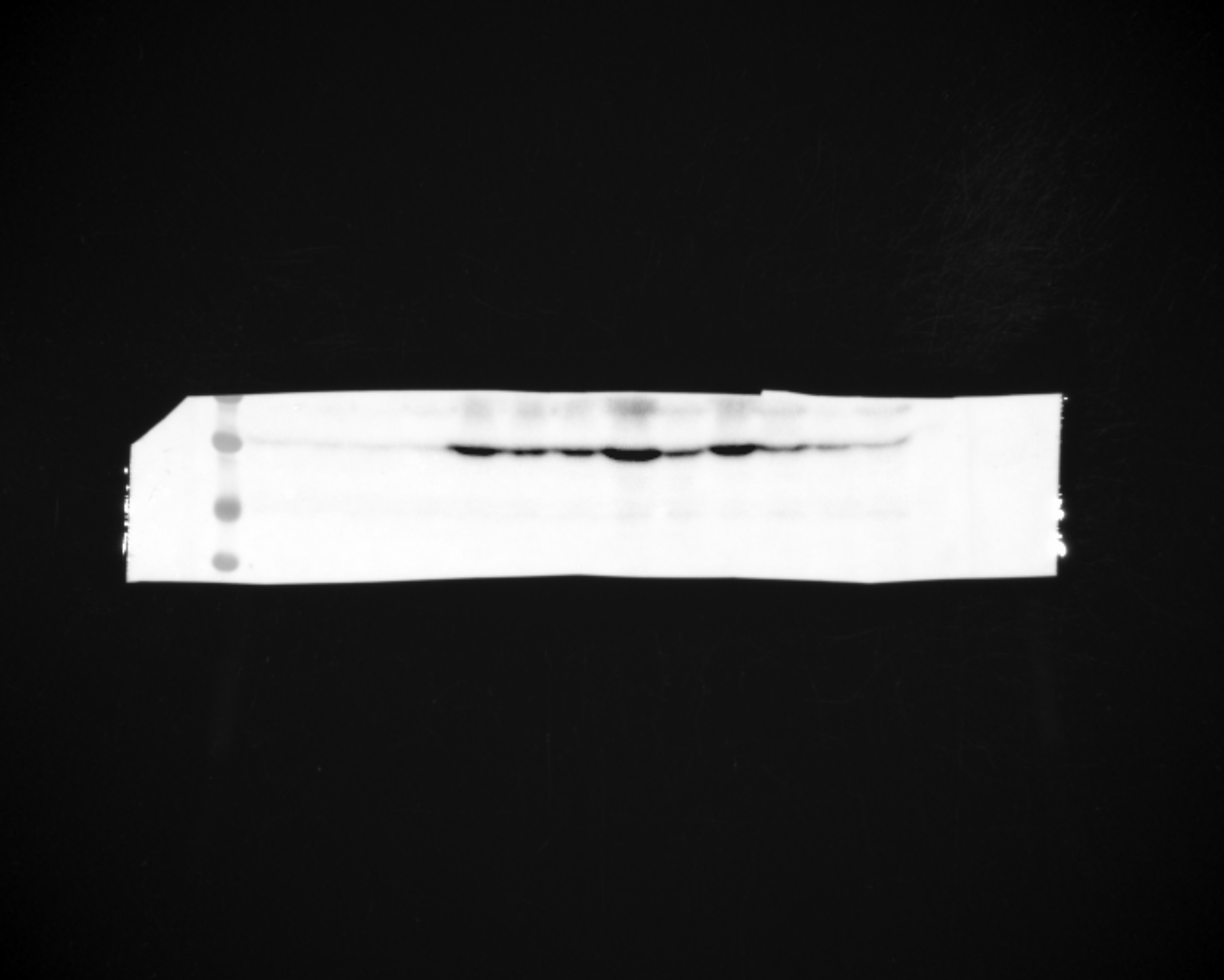

Supplement: Supplementary file 11 — Source data Fig. 8 [file 44321_2025_337_MOESM11_ESM.zip › Figure 8/Fig8I-L_Western blot/Fig8I_Western blot images/Western MuRF1.tif]

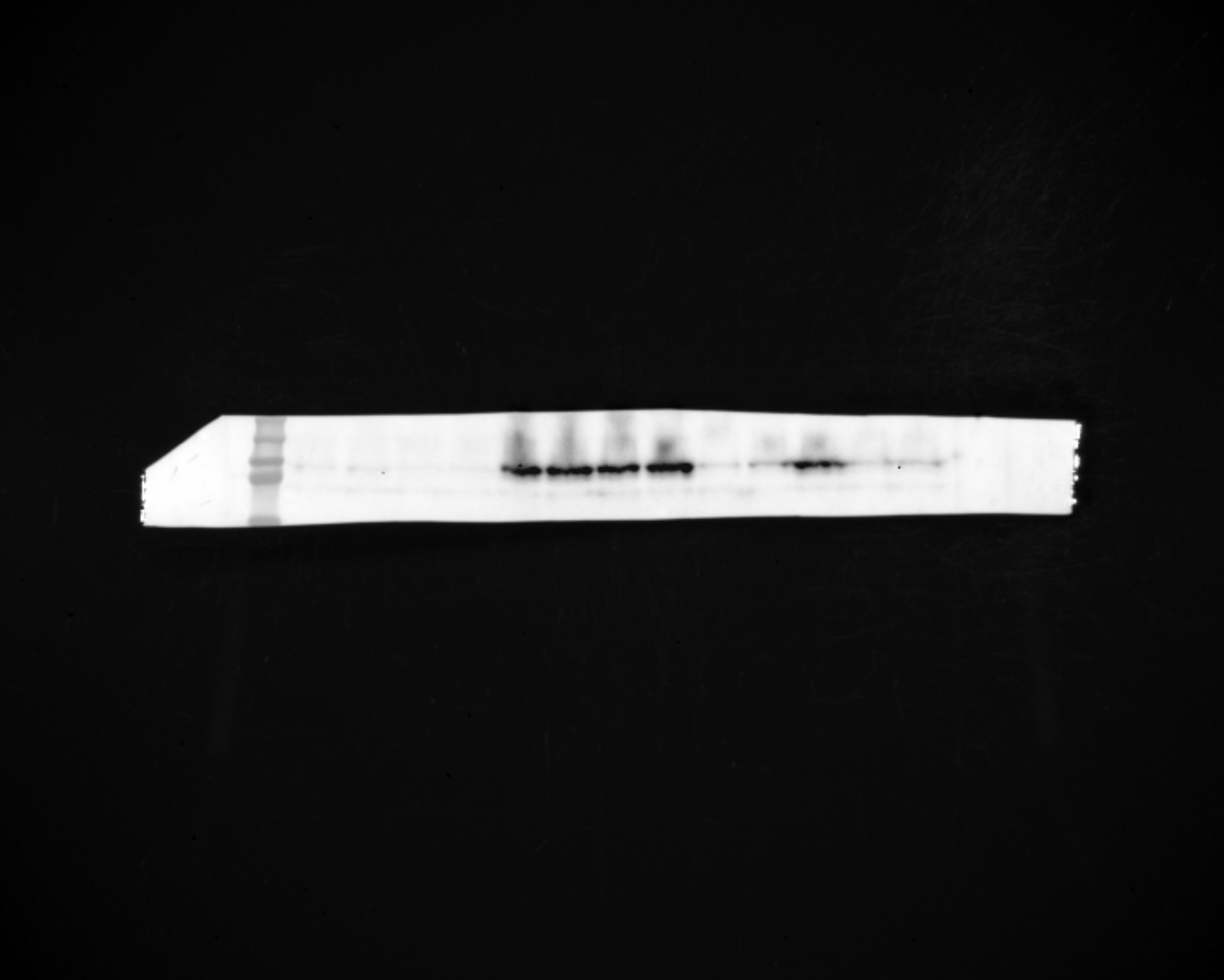

Supplement: Supplementary file 11 — Source data Fig. 8 [file 44321_2025_337_MOESM11_ESM.zip › Figure 8/Fig8I-L_Western blot/Fig8I_Western blot images/Western p-STAT3.tif]

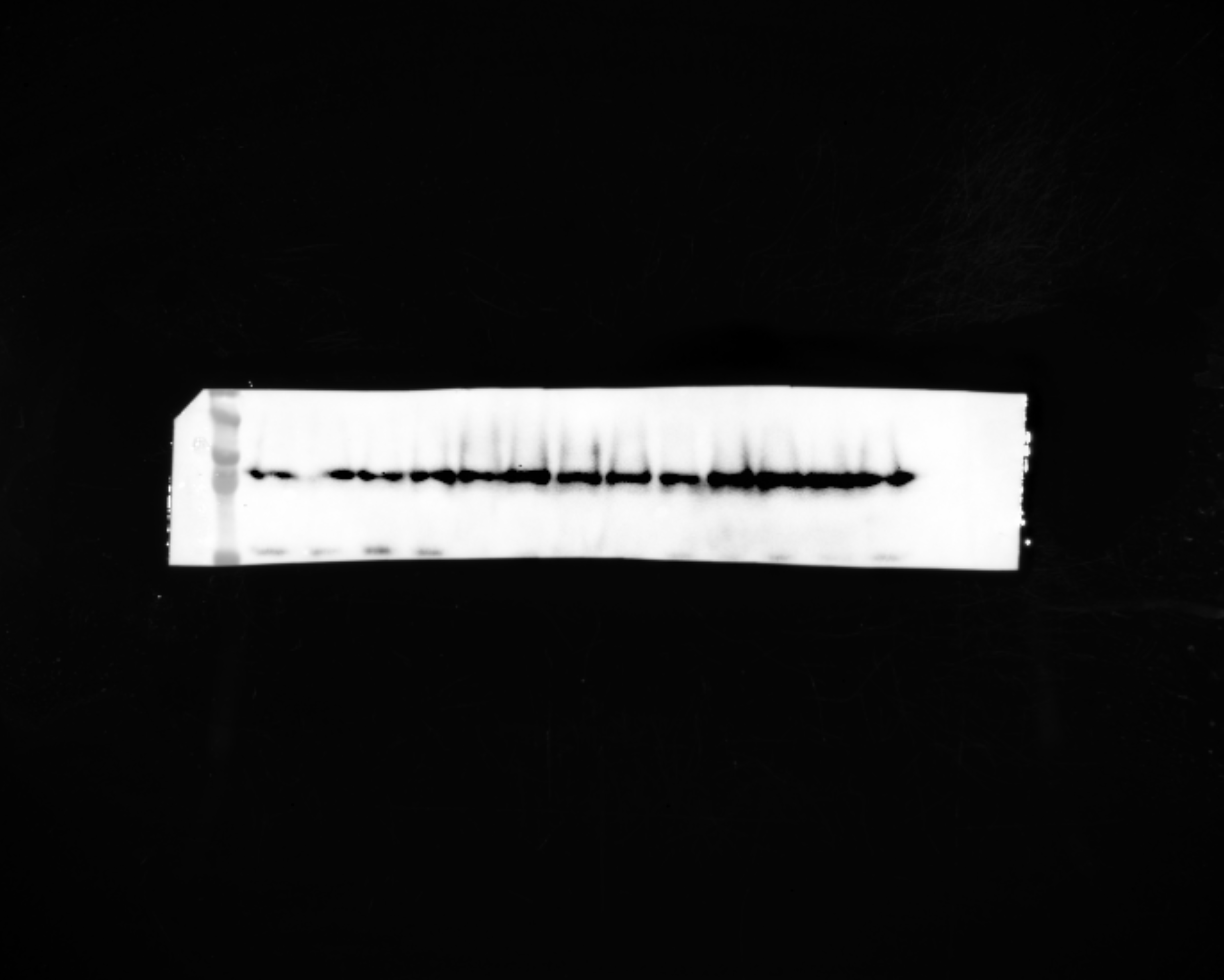

Supplement: Supplementary file 11 — Source data Fig. 8 [file 44321_2025_337_MOESM11_ESM.zip › Figure 8/Fig8I-L_Western blot/Fig8I_Western blot images/Western STAT3.tif]

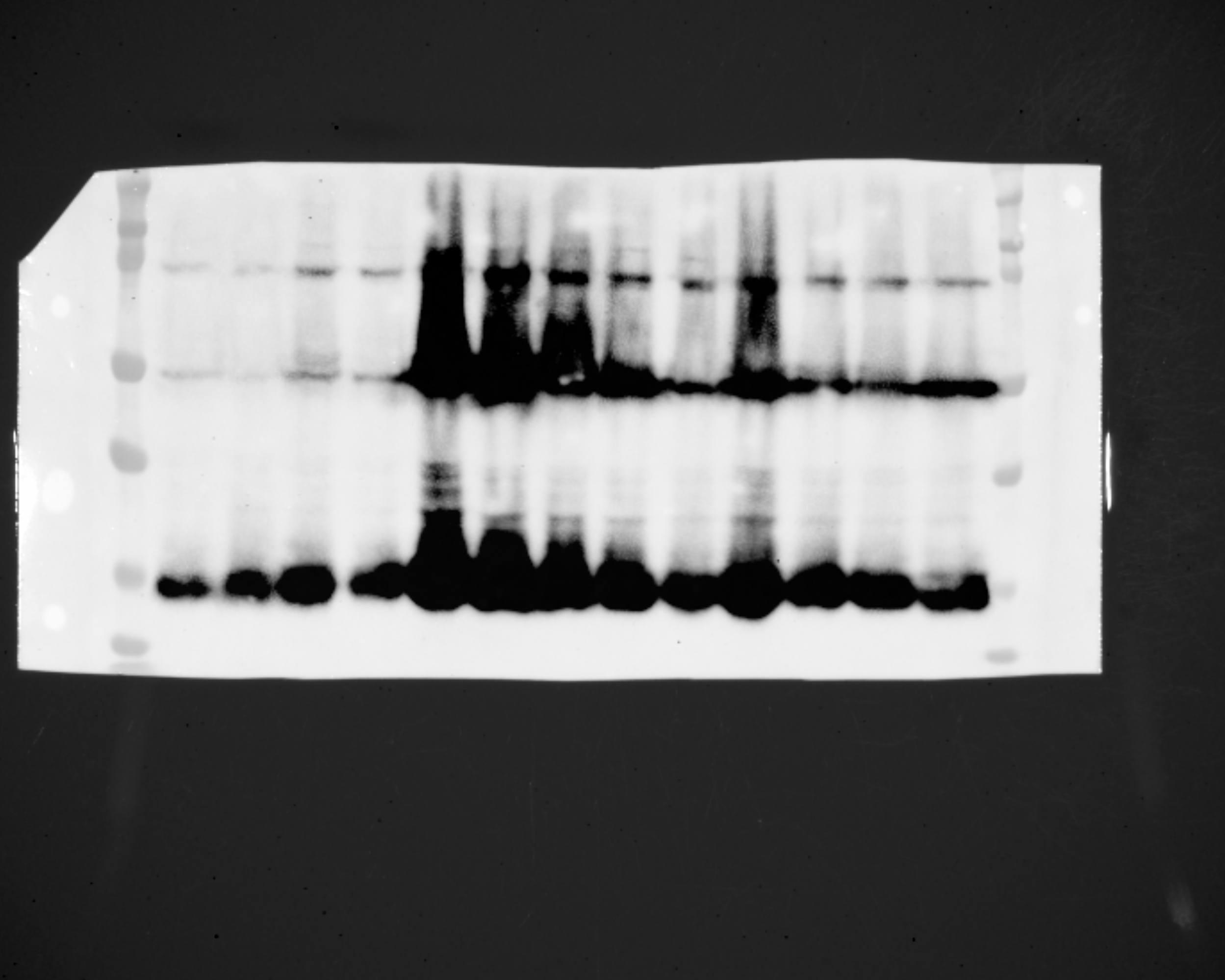

Supplement: Supplementary file 11 — Source data Fig. 8 [file 44321_2025_337_MOESM11_ESM.zip › Figure 8/Fig8I-L_Western blot/Fig8I_Western blot images/Western Ub-conjugated proteins.tif]

## Slide 1
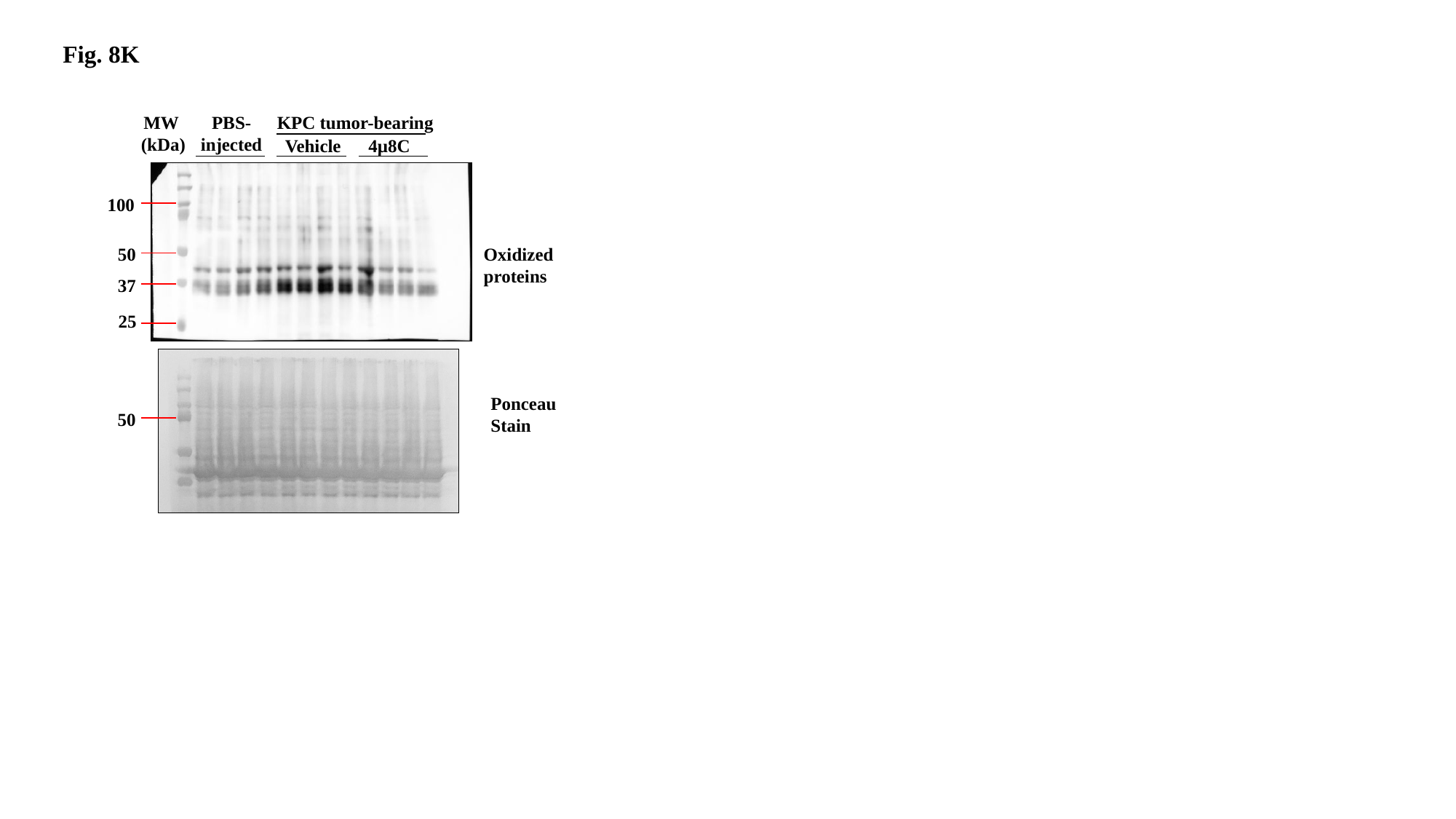

Fig. 8K
MW
(kDa)
PBS-injected
KPC tumor-bearing
Vehicle
4μ8C
100
50
Oxidized proteins
37
25
Ponceau Stain
50

Supplement: Supplementary file 11 — Source data Fig. 8 [file 44321_2025_337_MOESM11_ESM.zip › Figure 8/Fig8I-L_Western blot/Fig8K_Western blot images/Fig8K_Western blot.pptx]

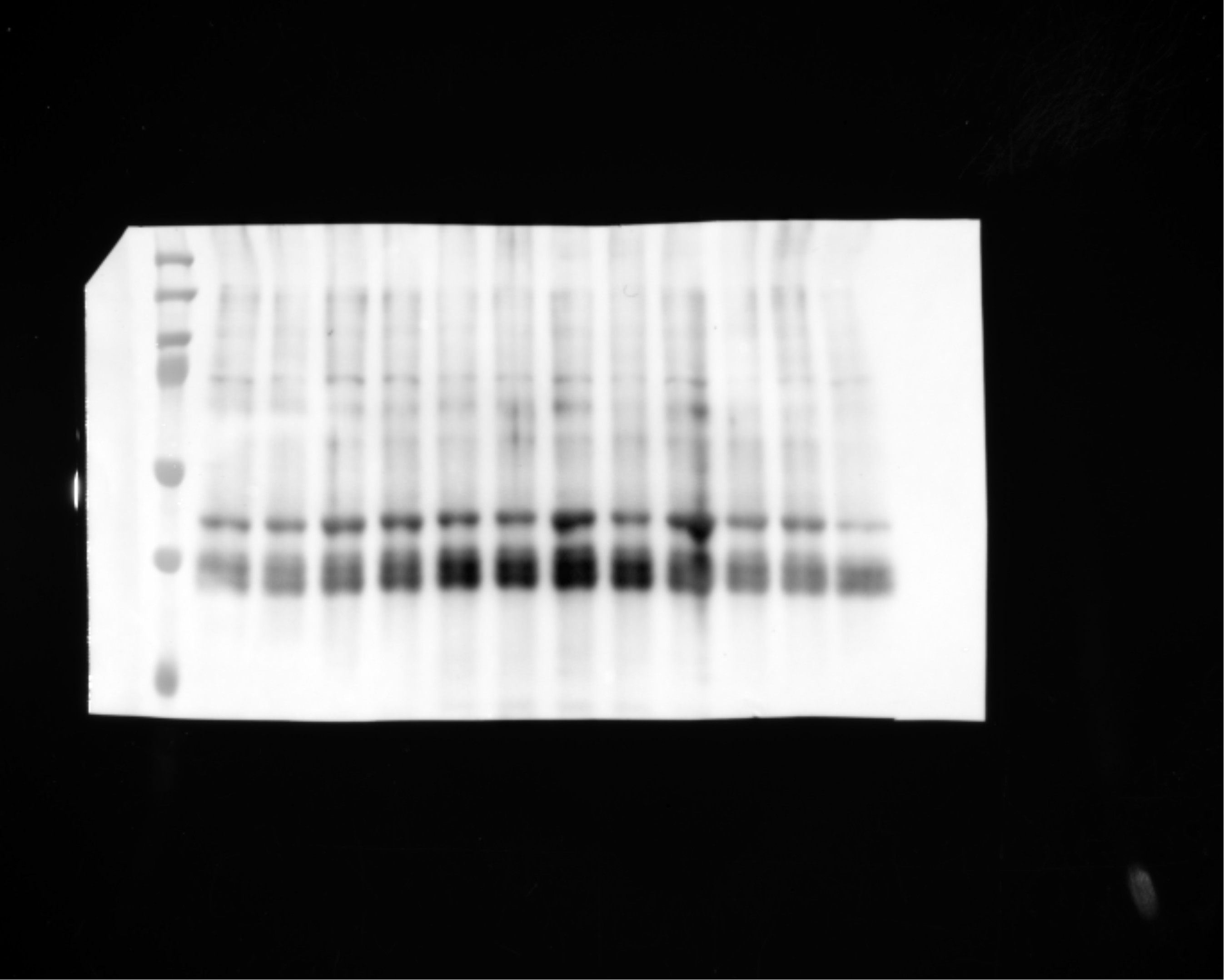

Supplement: Supplementary file 11 — Source data Fig. 8 [file 44321_2025_337_MOESM11_ESM.zip › Figure 8/Fig8I-L_Western blot/Fig8K_Western blot images/Western Oxidized proteins.tif]

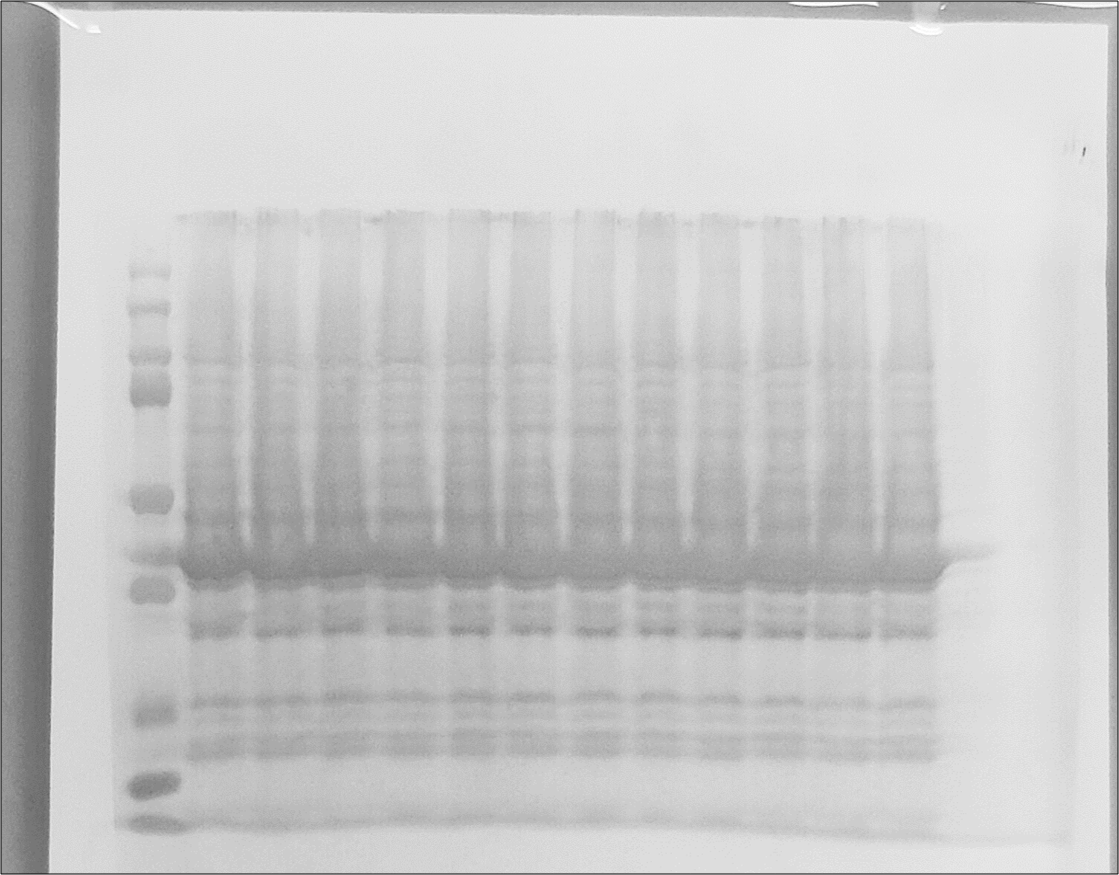

Supplement: Supplementary file 11 — Source data Fig. 8 [file 44321_2025_337_MOESM11_ESM.zip › Figure 8/Fig8I-L_Western blot/Fig8K_Western blot images/Western Ponceau Stain.tif]

## Slide 1
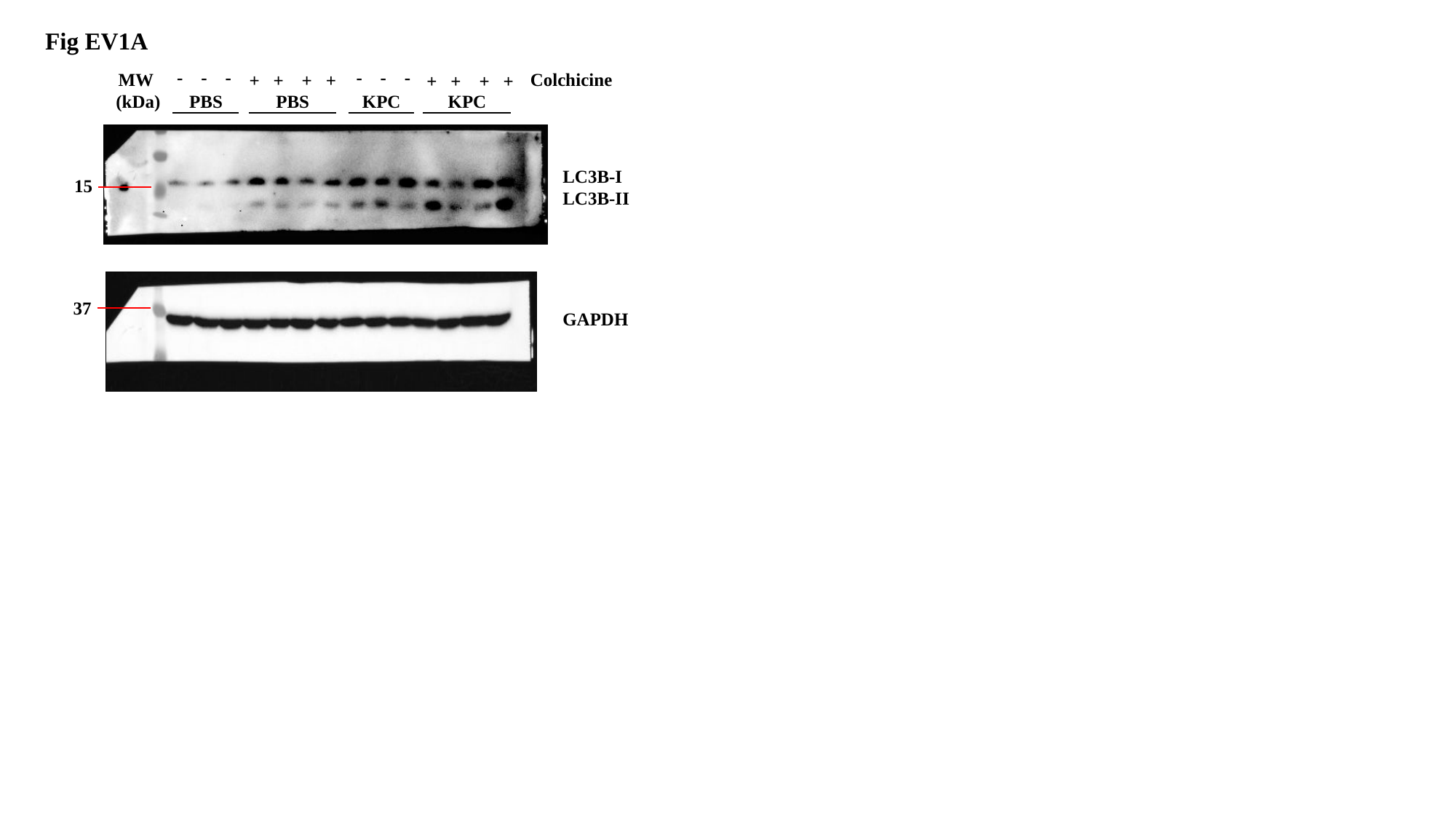

Fig EV1A
- - -
- - -
MW
(kDa)
Colchicine
+ + + +
+ + + +
PBS
PBS
KPC
KPC
LC3B-I
LC3B-II
15
37
GAPDH

Supplement: Supplementary file 12 — Figure EV1 Source Data [file 44321_2025_337_MOESM12_ESM.zip › Figure EV1/Fig EV1A_Western blot/Fig EV1A Western blot.pptx]

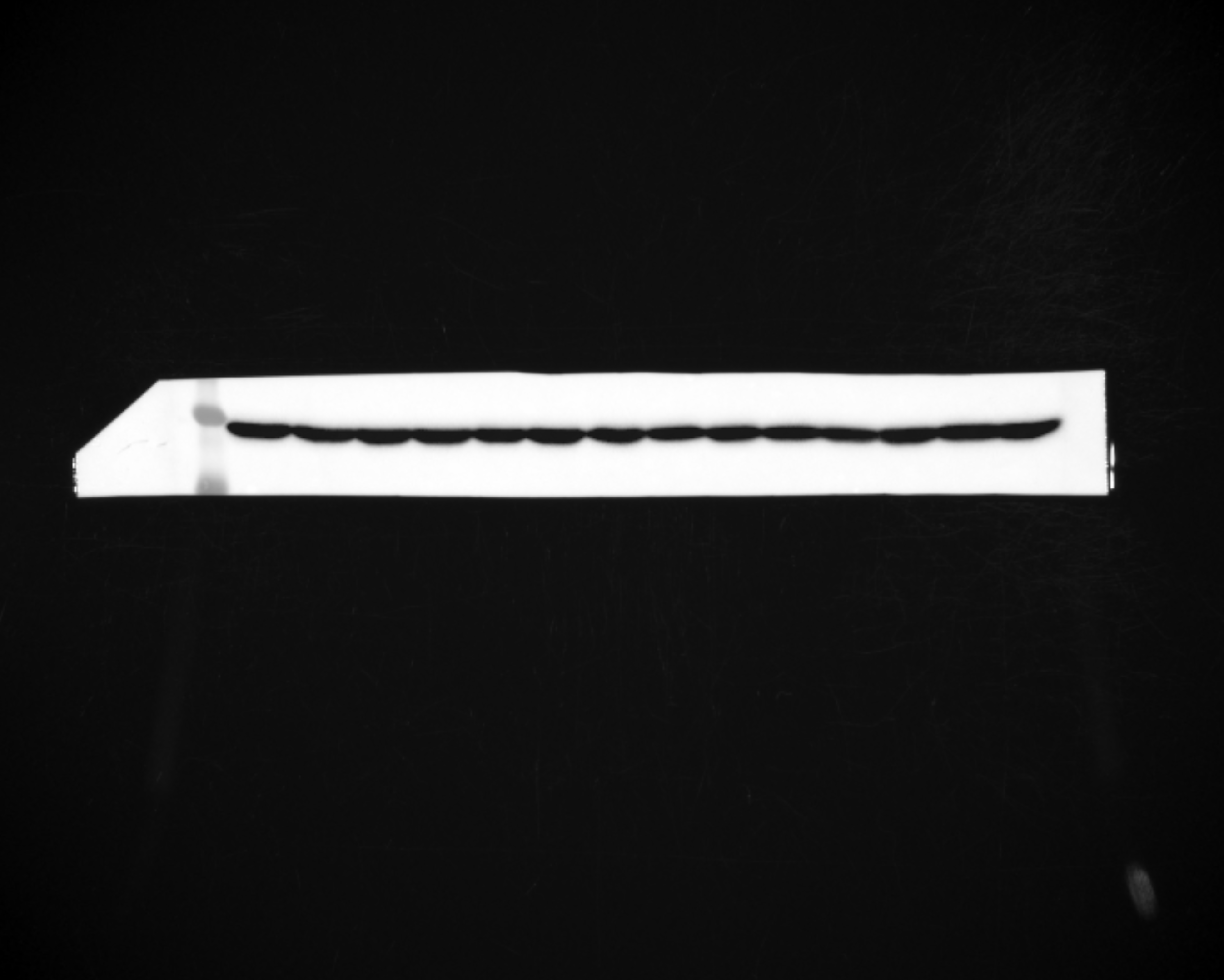

Supplement: Supplementary file 12 — Figure EV1 Source Data [file 44321_2025_337_MOESM12_ESM.zip › Figure EV1/Fig EV1A_Western blot/Western GAPDH.tif]

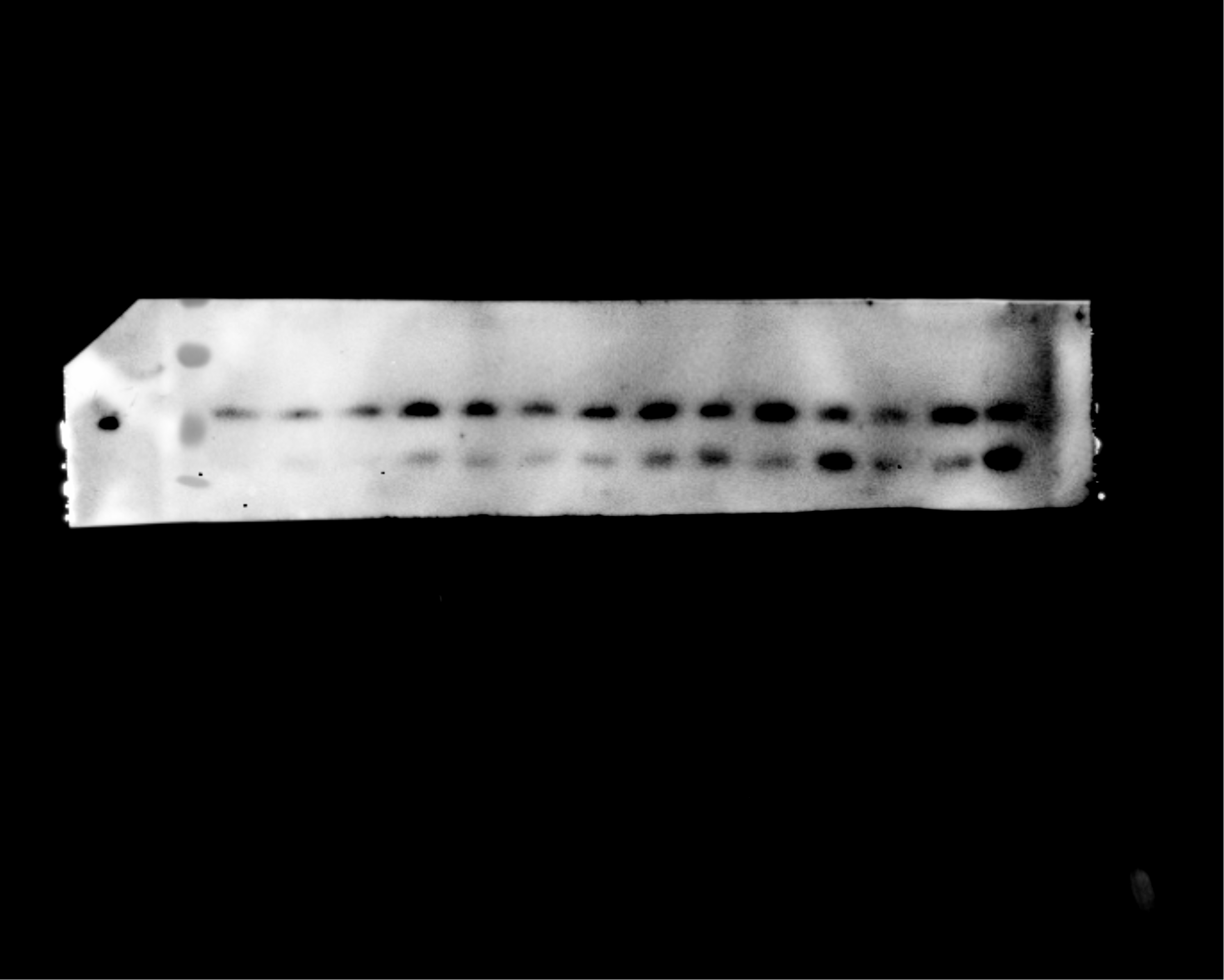

Supplement: Supplementary file 12 — Figure EV1 Source Data [file 44321_2025_337_MOESM12_ESM.zip › Figure EV1/Fig EV1A_Western blot/Western LC3B.tif]
