# Supplementary material for: The canonical ER stress IRE1α/XBP1 pathway mediates skeletal muscle wasting during pancreatic cancer cachexia
Source: EMBO Mol Med. 2025 Nov 17;17(12):3607–35. doi: 10.1038/s44321-025-00337-w (PMC12686462; doi:10.1038/s44321-025-00337-w)
Supplement: Supplementary file 7 — Source data Fig. 4 [file 44321_2025_337_MOESM7_ESM.zip › Figure 4/Fig4C_E_Western blot images/Fig4C_Western blot images/Fig4C_Western blot.pptx]

## Slide 1
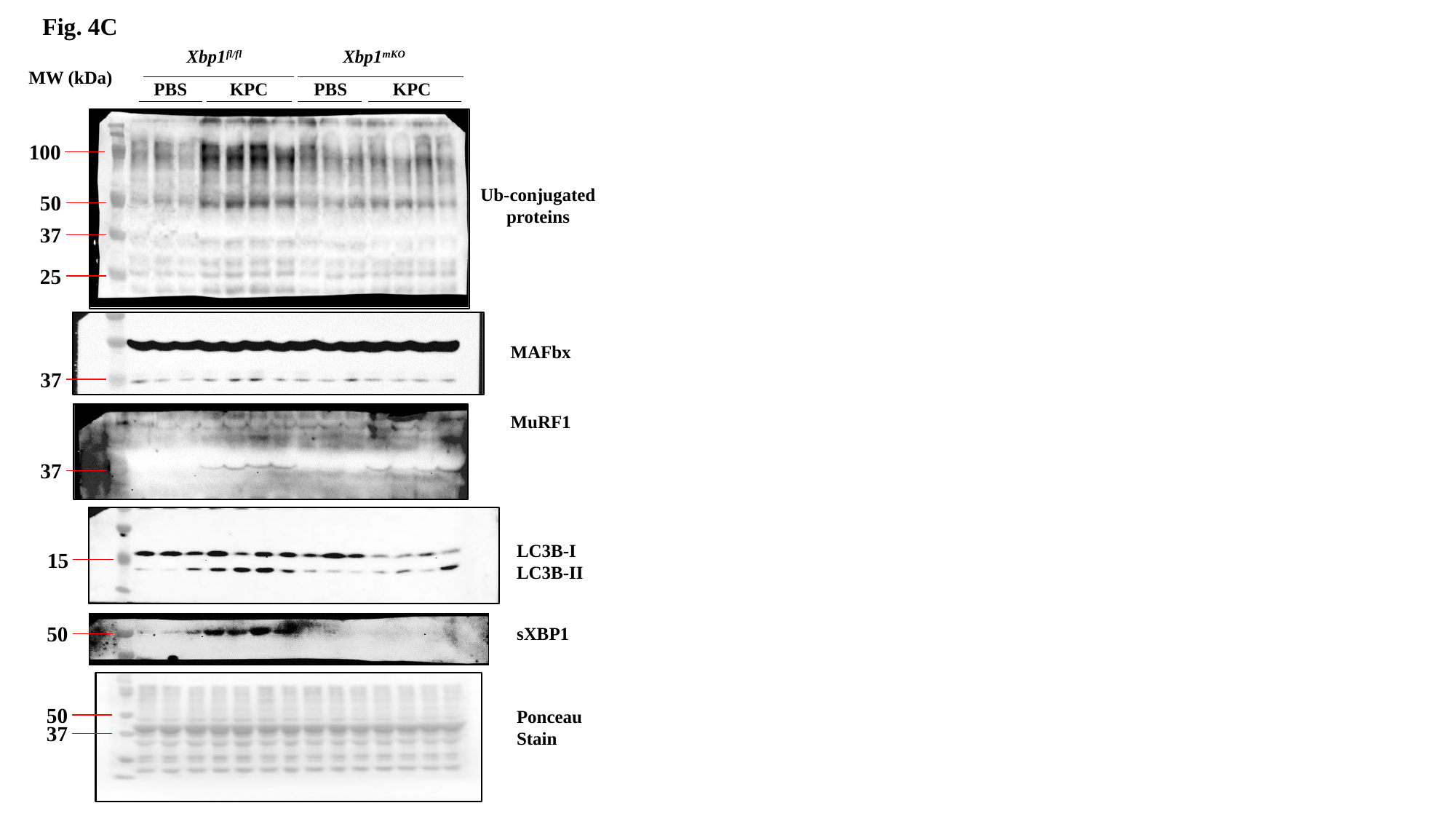

Fig. 4C
Xbp1fl/fl
Xbp1mKO
MW (kDa)
PBS
KPC
PBS
KPC
100
Ub-conjugated proteins
50
37
25
MAFbx
37
MuRF1
37
LC3B-I
LC3B-II
15
50
sXBP1
50
Ponceau
Stain
37
