# Supplementary material for: The canonical ER stress IRE1α/XBP1 pathway mediates skeletal muscle wasting during pancreatic cancer cachexia
Source: EMBO Mol Med. 2025 Nov 17;17(12):3607–35. doi: 10.1038/s44321-025-00337-w (PMC12686462; doi:10.1038/s44321-025-00337-w)
Supplement: Supplementary file 10 — Source data Fig. 7 [file 44321_2025_337_MOESM10_ESM.zip › Figure 7/Fig7C_E_G_I_ChIP-PCR/Fig7C_E_G_I_ChIP-PCR_Agarose gel images/Fig7C_E_G_I_ChIP-PCR_Agarsoe gel images.pptx]

## Slide 1
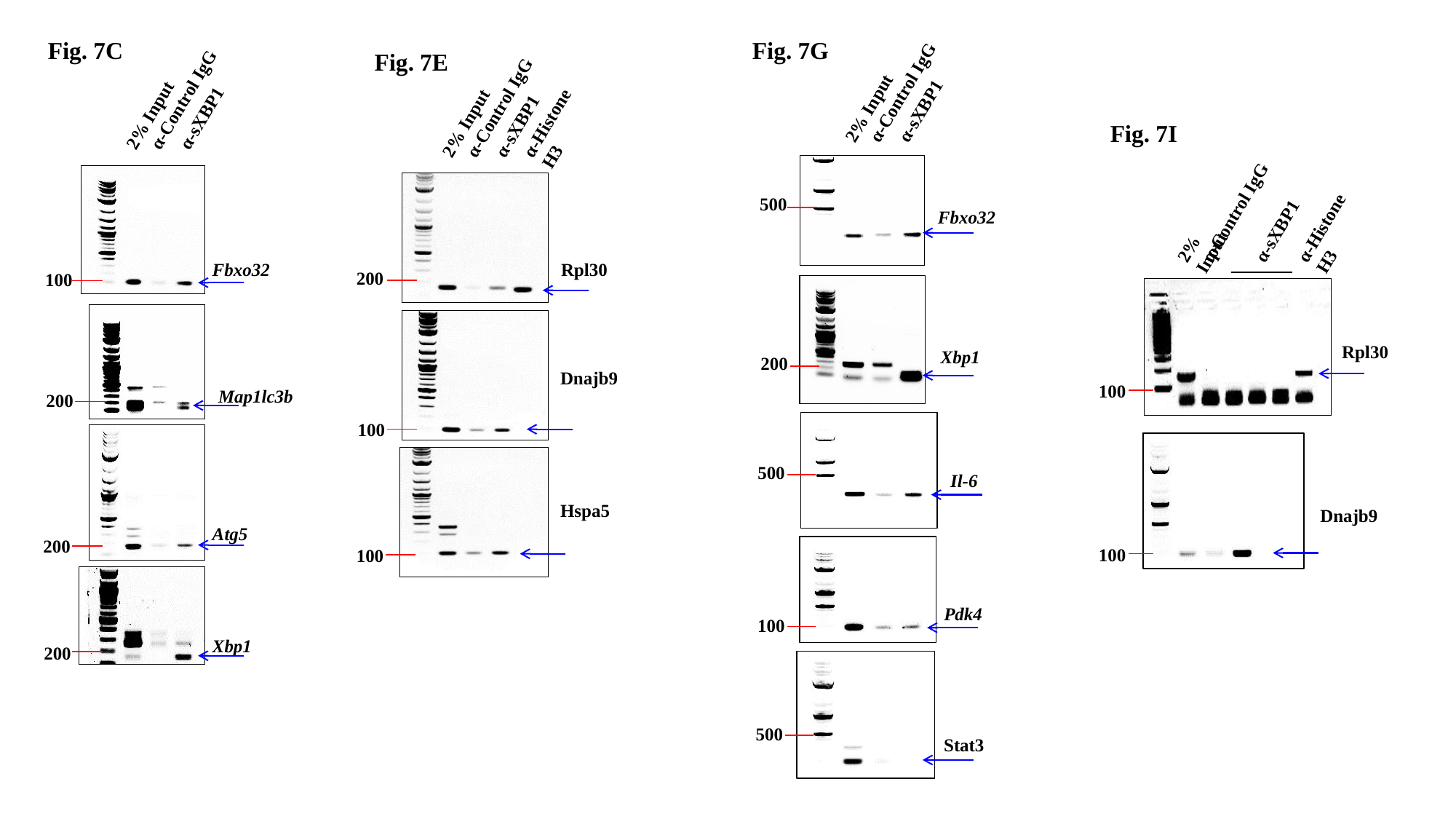

α-Control IgG
α-Control IgG
Fig. 7C
Fig. 7G
α-Control IgG
Fig. 7E
α-sXBP1
α-Histone H3
α-sXBP1
2% Input
α-sXBP1
2% Input
2% Input
Fig. 7I
α-Control IgG
α-Histone H3
α-sXBP1
2% Input
500
Fbxo32
Rpl30
Fbxo32
200
100
Rpl30
Xbp1
200
Dnajb9
100
Map1lc3b
200
100
500
Il-6
Hspa5
Dnajb9
Atg5
200
100
100
Pdk4
100
Xbp1
200
500
Stat3
