# Supplementary material for: The canonical ER stress IRE1α/XBP1 pathway mediates skeletal muscle wasting during pancreatic cancer cachexia
Source: EMBO Mol Med. 2025 Nov 17;17(12):3607–35. doi: 10.1038/s44321-025-00337-w (PMC12686462; doi:10.1038/s44321-025-00337-w)
Supplement: Supplementary file 11 — Source data Fig. 8 [file 44321_2025_337_MOESM11_ESM.zip › Figure 8/Fig8I-L_Western blot/Fig8I_Western blot images/Fig8I_Western blot.pptx]

## Slide 1
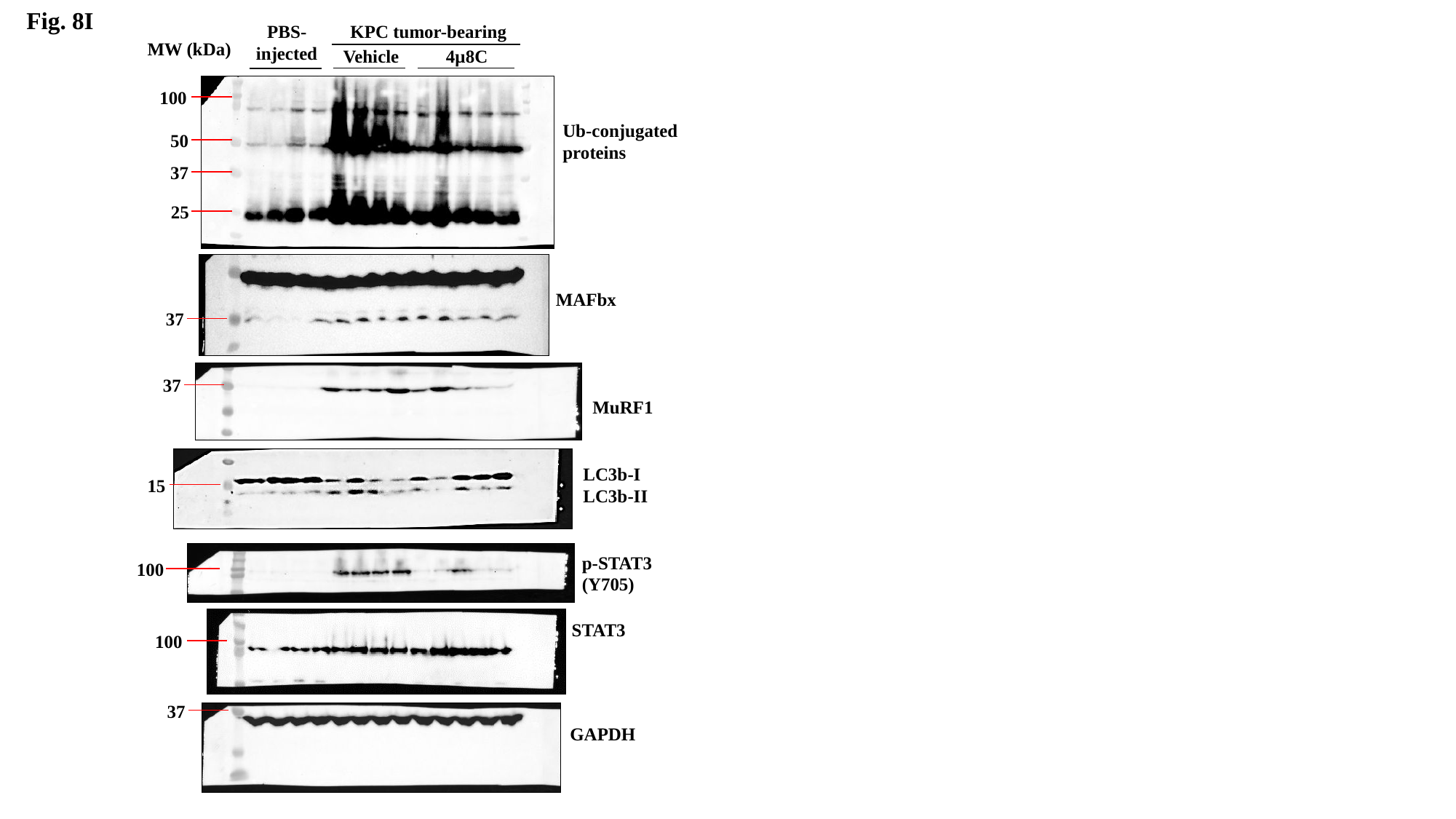

Fig. 8I
PBS-injected
KPC tumor-bearing
MW (kDa)
Vehicle
4μ8C
100
Ub-conjugated proteins
50
37
25
MAFbx
37
37
MuRF1
LC3b-I
LC3b-II
15
p-STAT3
(Y705)
100
STAT3
100
37
GAPDH
