# Supplementary figures and images for: The canonical ER stress IRE1α/XBP1 pathway mediates skeletal muscle wasting during pancreatic cancer cachexia (part 2 of 2)
Source: EMBO Mol Med. 2025 Nov 17;17(12):3607–35. doi: 10.1038/s44321-025-00337-w (PMC12686462; doi:10.1038/s44321-025-00337-w)

## Slide 1
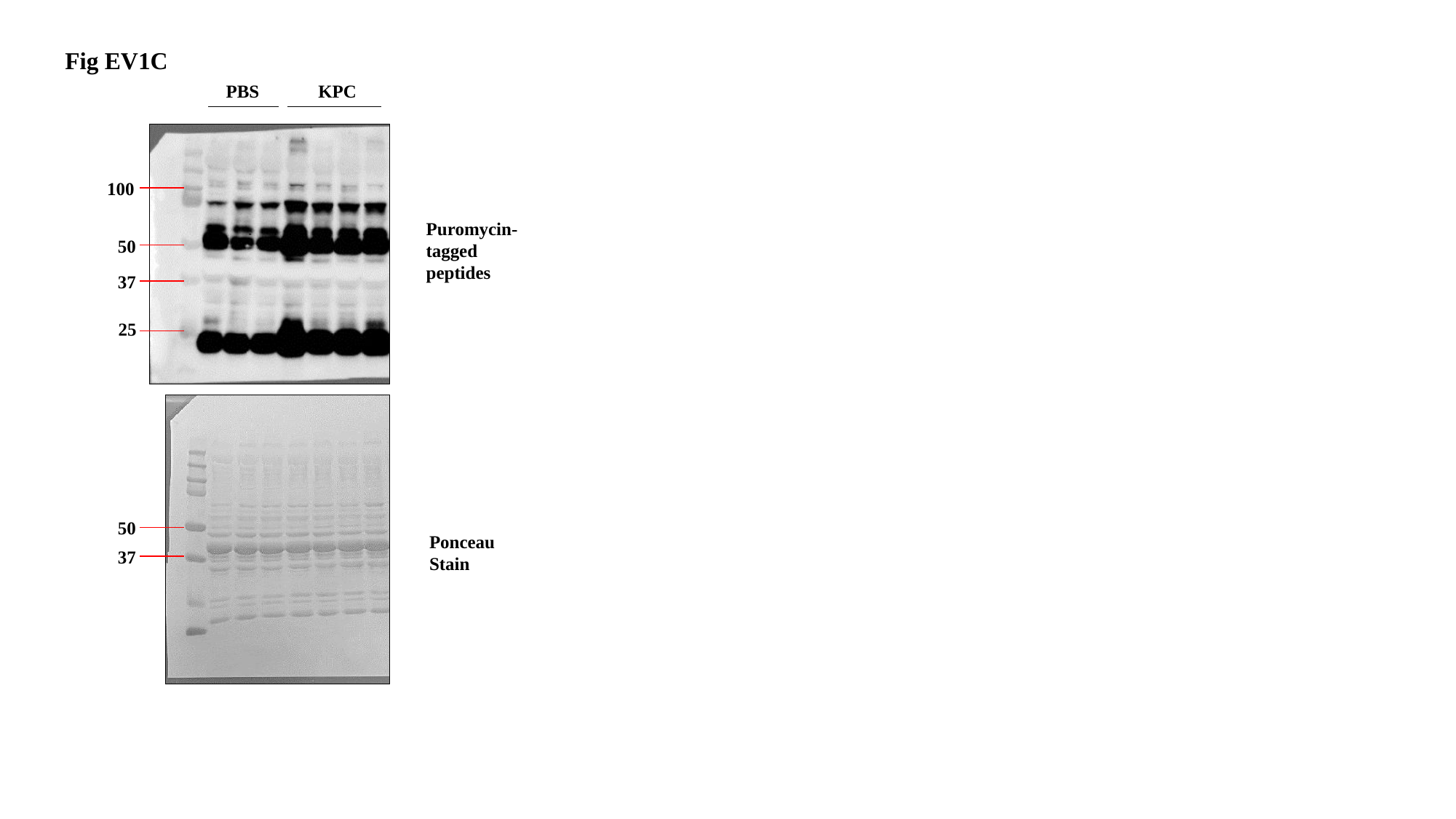

Fig EV1C
PBS
KPC
100
Puromycin- tagged peptides
50
37
25
50
Ponceau Stain
37

Supplement: Supplementary file 12 — Figure EV1 Source Data [file 44321_2025_337_MOESM12_ESM.zip › Figure EV1/Fig EV1C_Western blot/Fig EV1C Western blot.pptx]

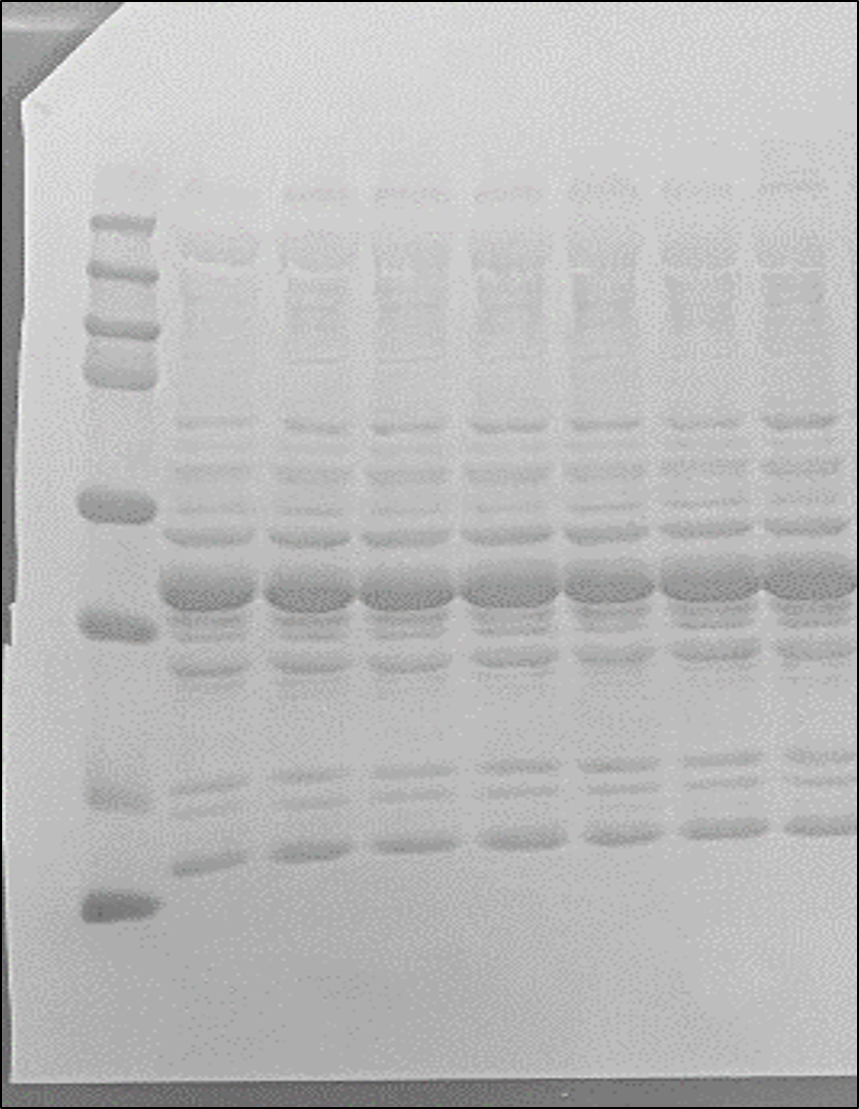

Supplement: Supplementary file 12 — Figure EV1 Source Data [file 44321_2025_337_MOESM12_ESM.zip › Figure EV1/Fig EV1C_Western blot/Western Ponceau Stain.tif]

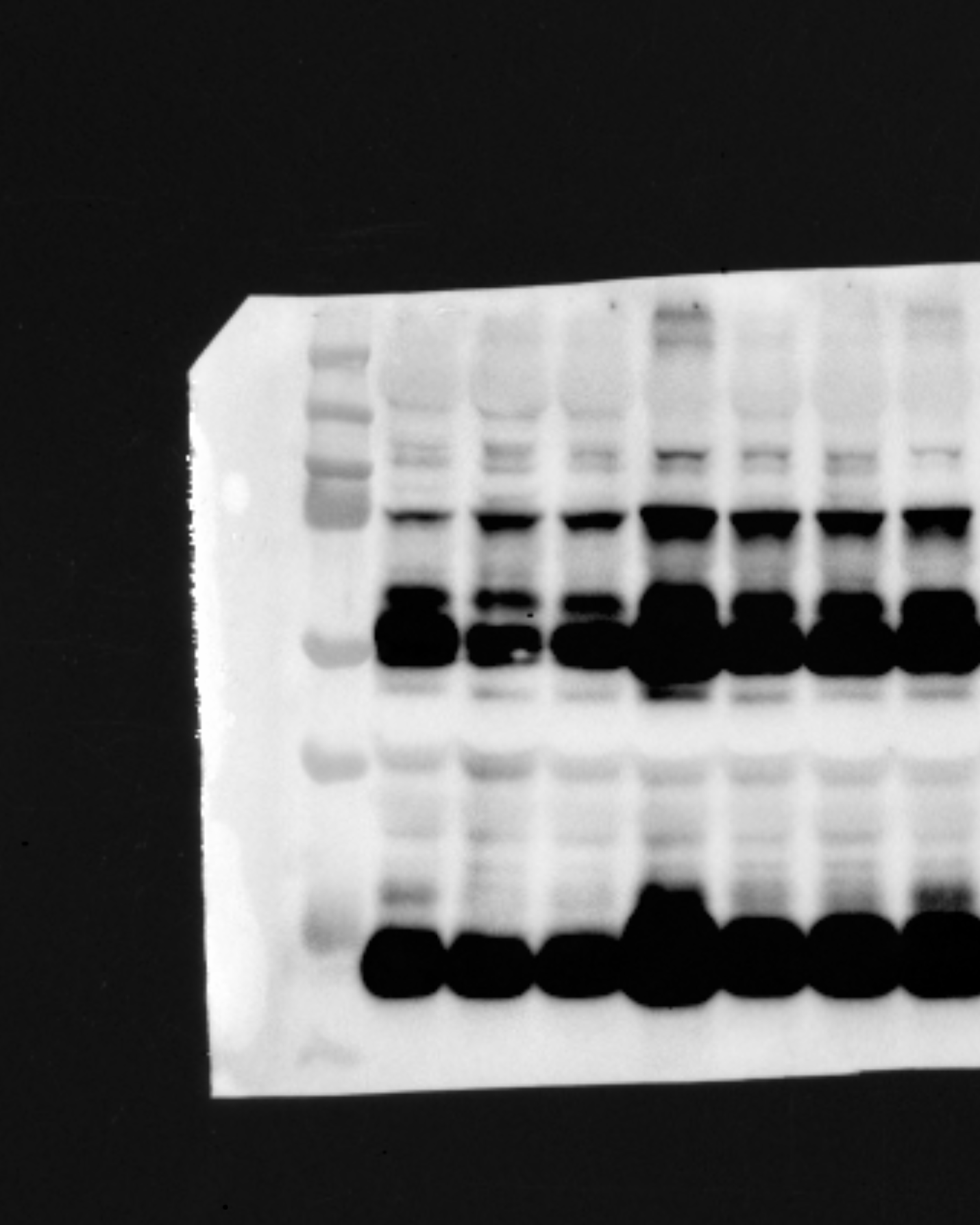

Supplement: Supplementary file 12 — Figure EV1 Source Data [file 44321_2025_337_MOESM12_ESM.zip › Figure EV1/Fig EV1C_Western blot/Western Puromycin-tagged peptides.tif]

## Slide 1
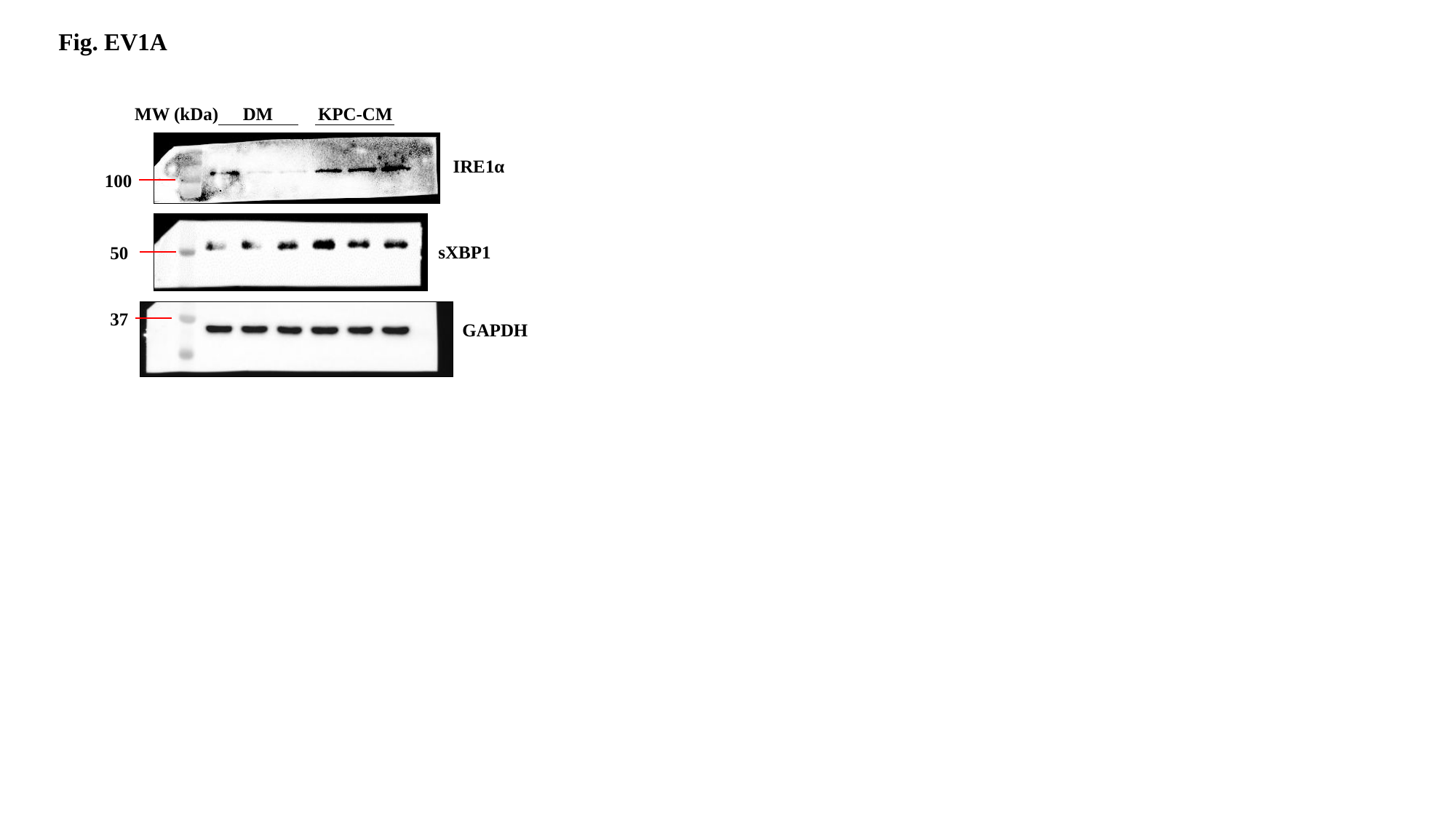

Fig. EV1A
MW (kDa)
DM
KPC-CM
IRE1α
100
sXBP1
50
37
GAPDH

Supplement: Supplementary file 12 — Figure EV1 Source Data [file 44321_2025_337_MOESM12_ESM.zip › Figure EV1/Fig EV1E_Western blot/Fig EV1E Western blot.pptx]

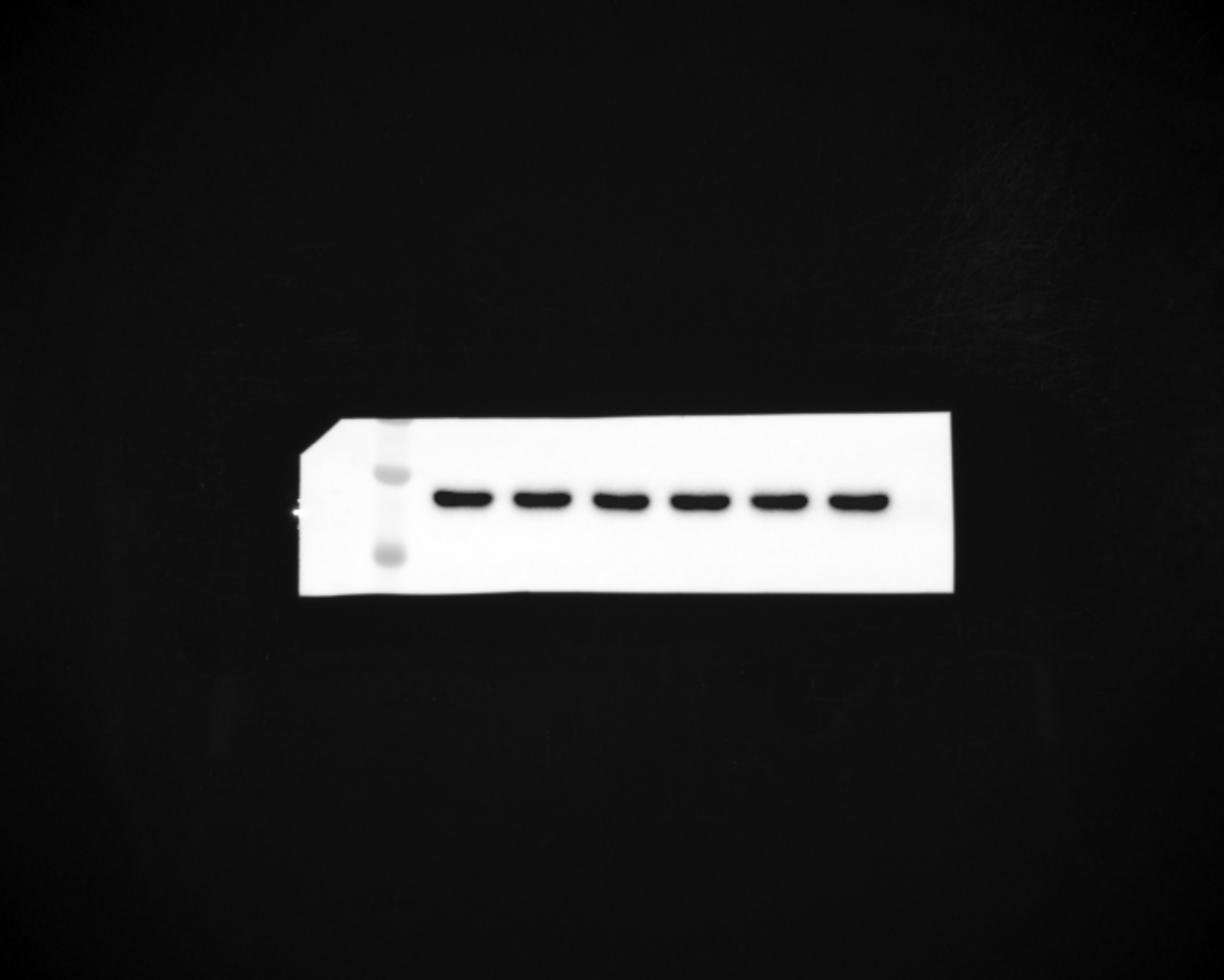

Supplement: Supplementary file 12 — Figure EV1 Source Data [file 44321_2025_337_MOESM12_ESM.zip › Figure EV1/Fig EV1E_Western blot/Western GAPDH.tif]

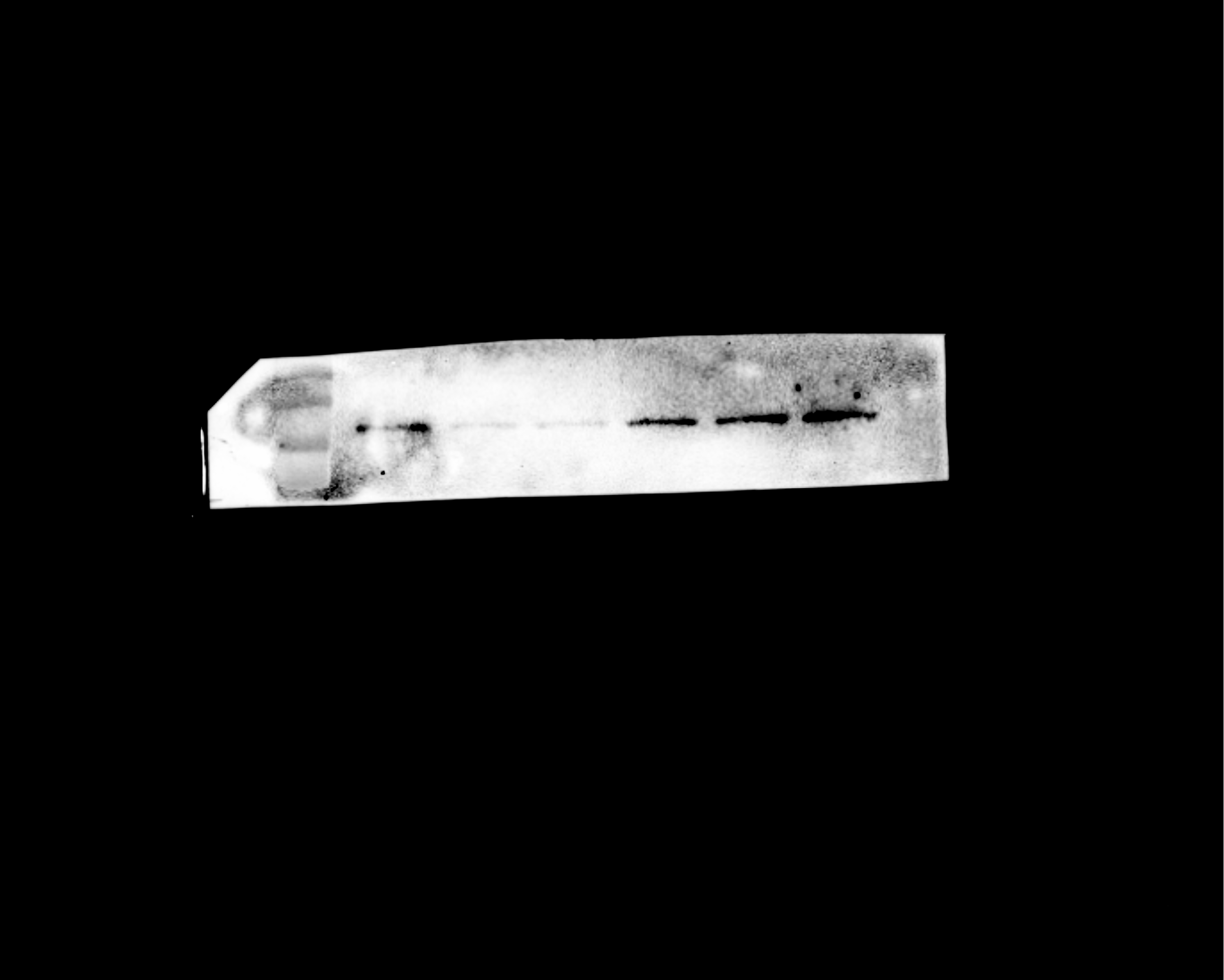

Supplement: Supplementary file 12 — Figure EV1 Source Data [file 44321_2025_337_MOESM12_ESM.zip › Figure EV1/Fig EV1E_Western blot/Western IRE1a.tif]

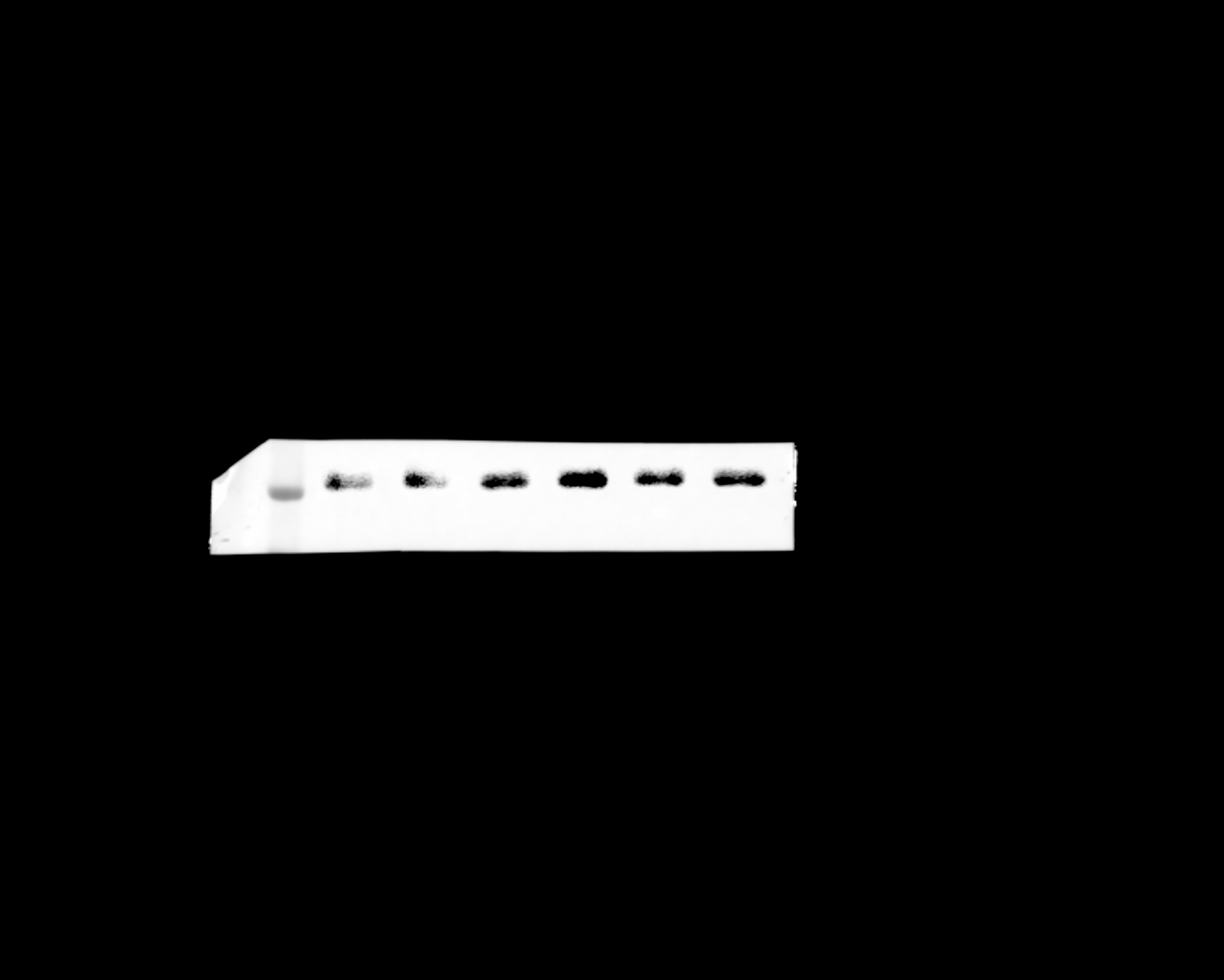

Supplement: Supplementary file 12 — Figure EV1 Source Data [file 44321_2025_337_MOESM12_ESM.zip › Figure EV1/Fig EV1E_Western blot/Western sXBP1.tif]

## Slide 1
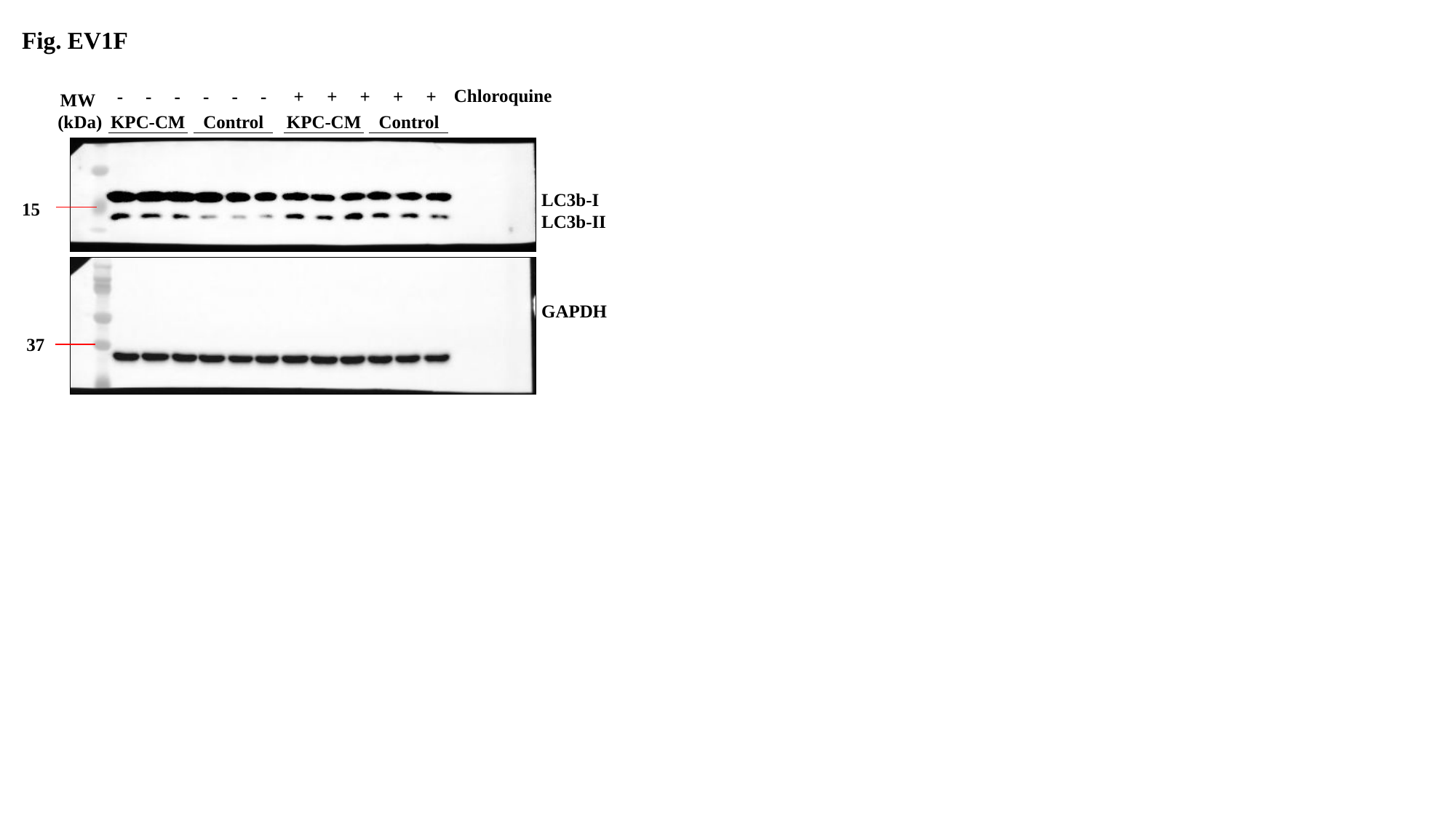

Fig. EV1F
Chloroquine
- - - - - - + + + + +
MW
(kDa)
KPC-CM
Control
KPC-CM
Control
LC3b-I
LC3b-II
15
GAPDH
37

Supplement: Supplementary file 12 — Figure EV1 Source Data [file 44321_2025_337_MOESM12_ESM.zip › Figure EV1/Fig EV1G_Western blot/Fig EV1G_Western blot images.pptx]

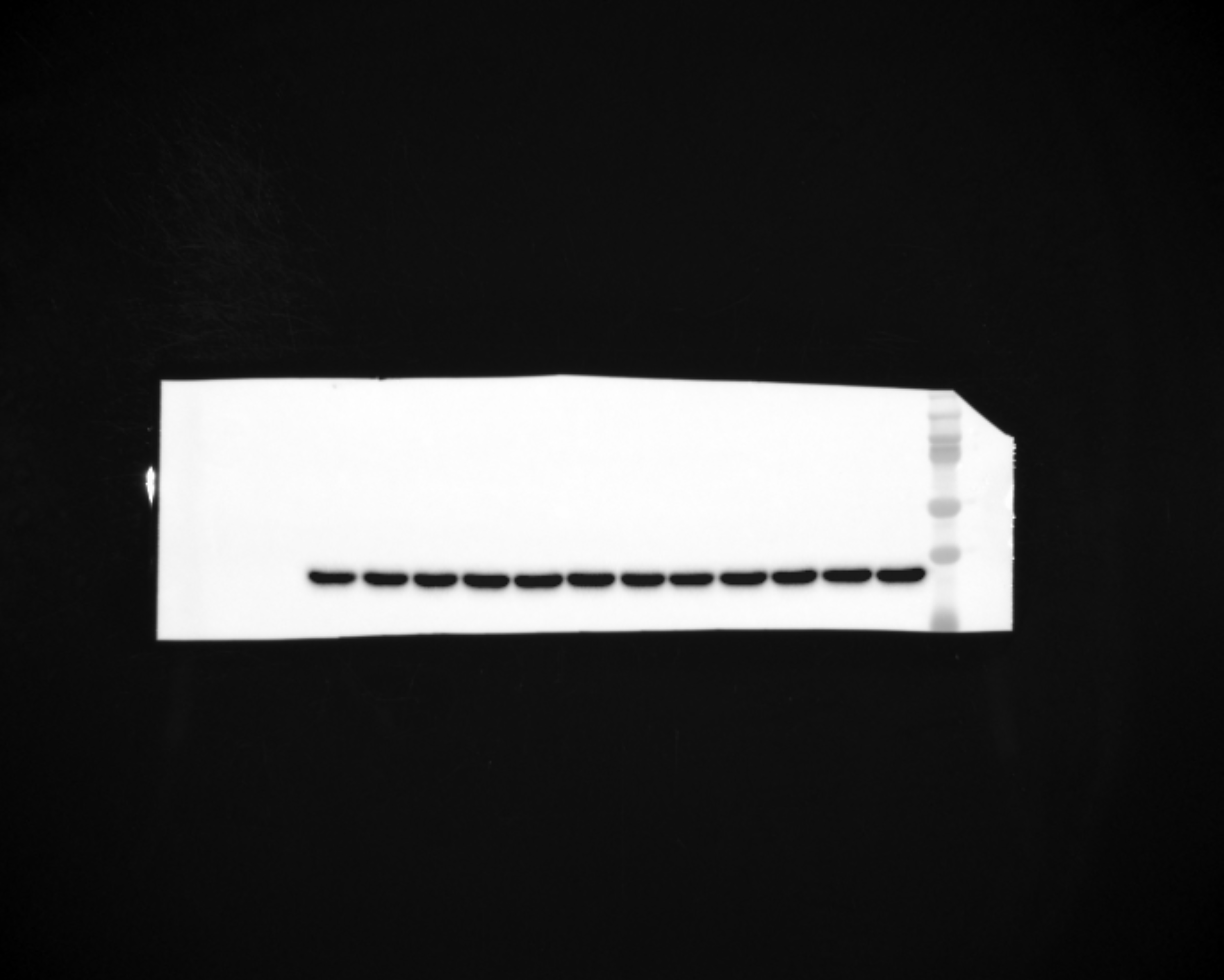

Supplement: Supplementary file 12 — Figure EV1 Source Data [file 44321_2025_337_MOESM12_ESM.zip › Figure EV1/Fig EV1G_Western blot/GAPDH.tif]

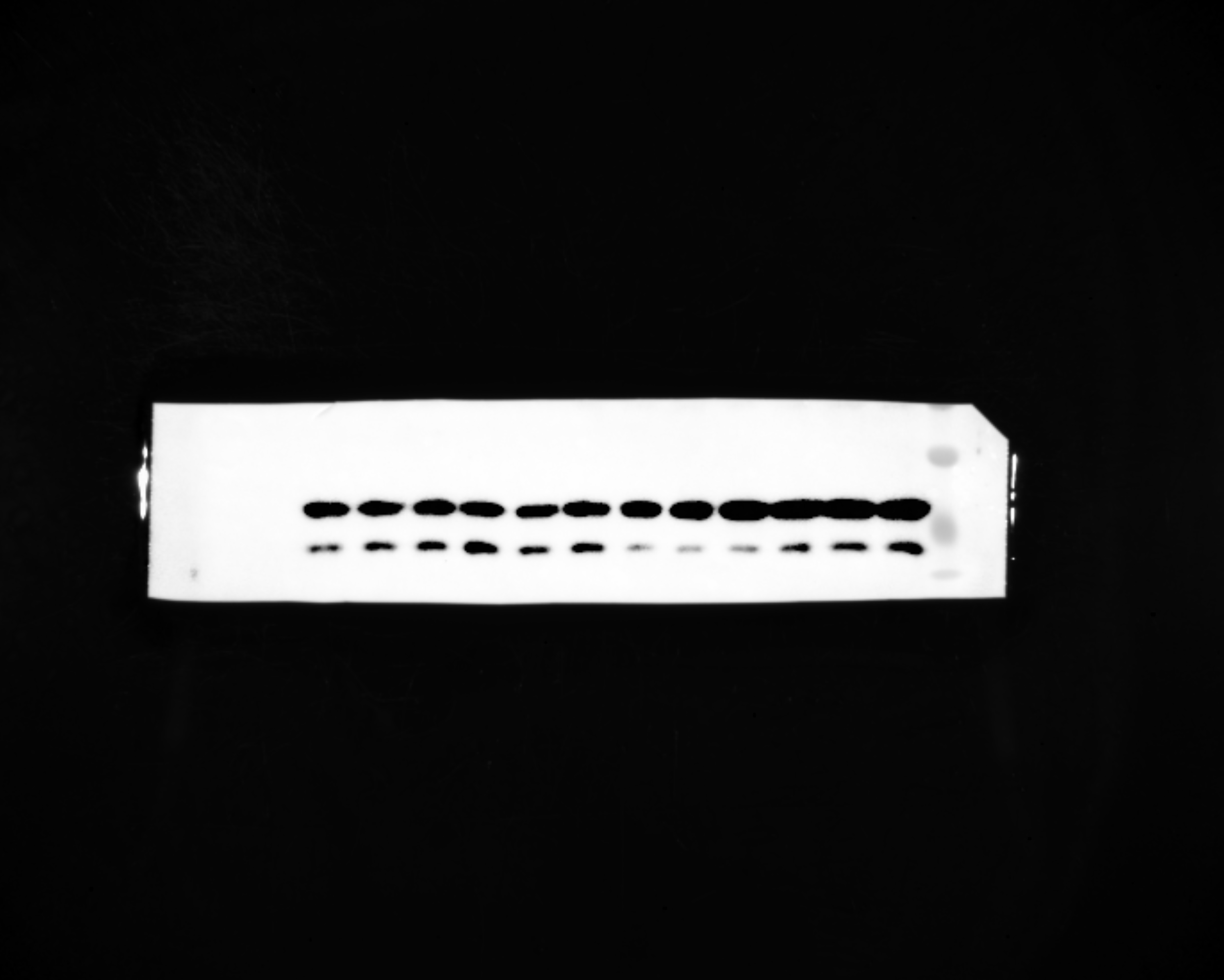

Supplement: Supplementary file 12 — Figure EV1 Source Data [file 44321_2025_337_MOESM12_ESM.zip › Figure EV1/Fig EV1G_Western blot/LC3B-I_II.tif]

## Slide 1
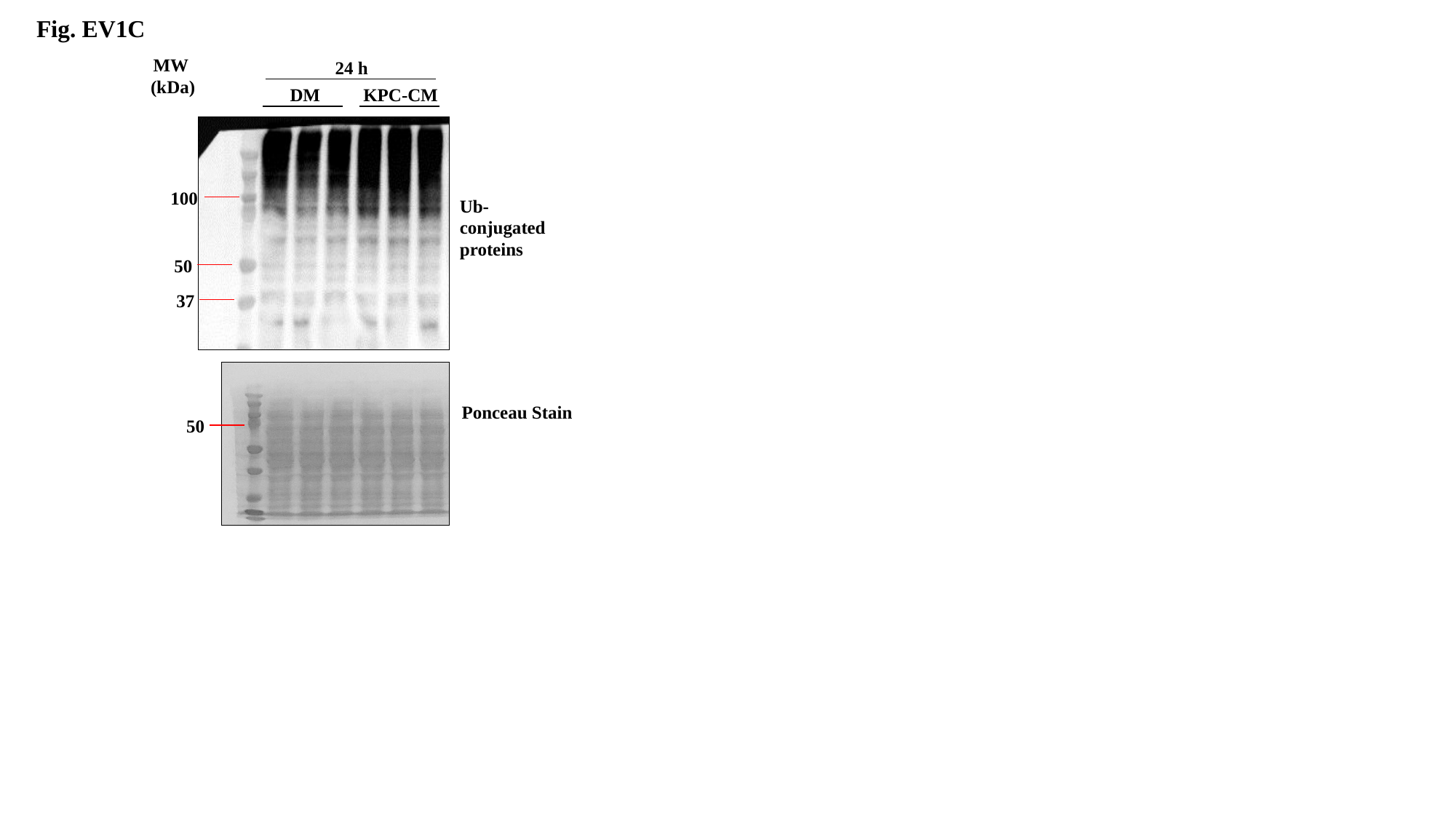

Fig. EV1C
MW
(kDa)
24 h
DM
KPC-CM
100
Ub-
conjugated proteins
50
37
Ponceau Stain
50

Supplement: Supplementary file 12 — Figure EV1 Source Data [file 44321_2025_337_MOESM12_ESM.zip › Figure EV1/Fig EV1I and K_Western blot/Fig EV1I-J Western blot and quantification/Fig EV1I_Western blot images.pptx]

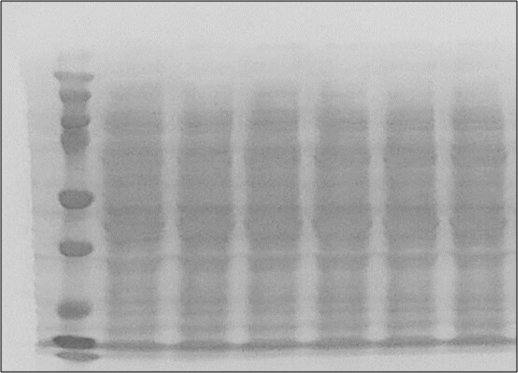

Supplement: Supplementary file 12 — Figure EV1 Source Data [file 44321_2025_337_MOESM12_ESM.zip › Figure EV1/Fig EV1I and K_Western blot/Fig EV1I-J Western blot and quantification/Western Ponceau stain.tif]

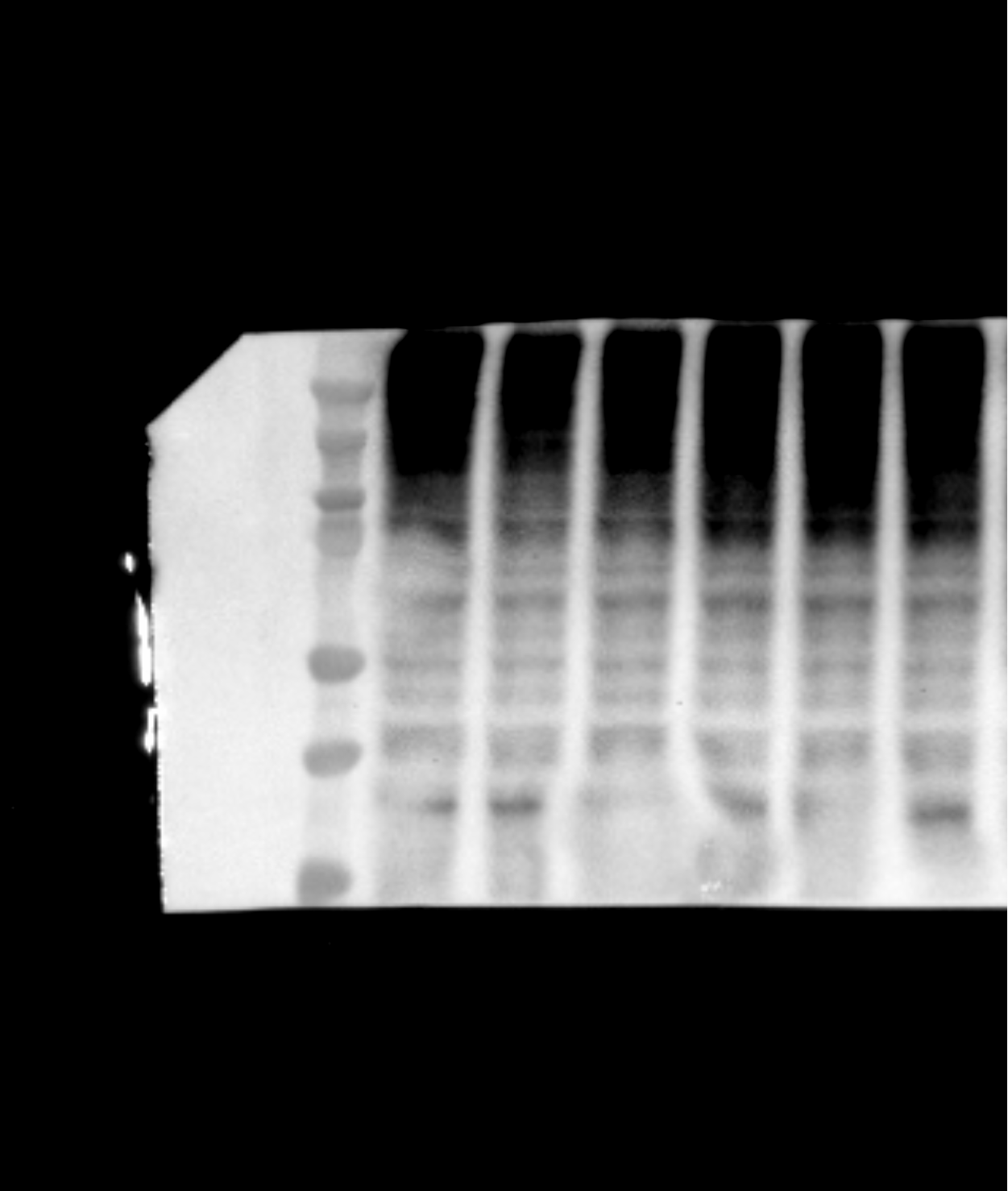

Supplement: Supplementary file 12 — Figure EV1 Source Data [file 44321_2025_337_MOESM12_ESM.zip › Figure EV1/Fig EV1I and K_Western blot/Fig EV1I-J Western blot and quantification/Western Ub-conjugated proteins.tif]

## Slide 1
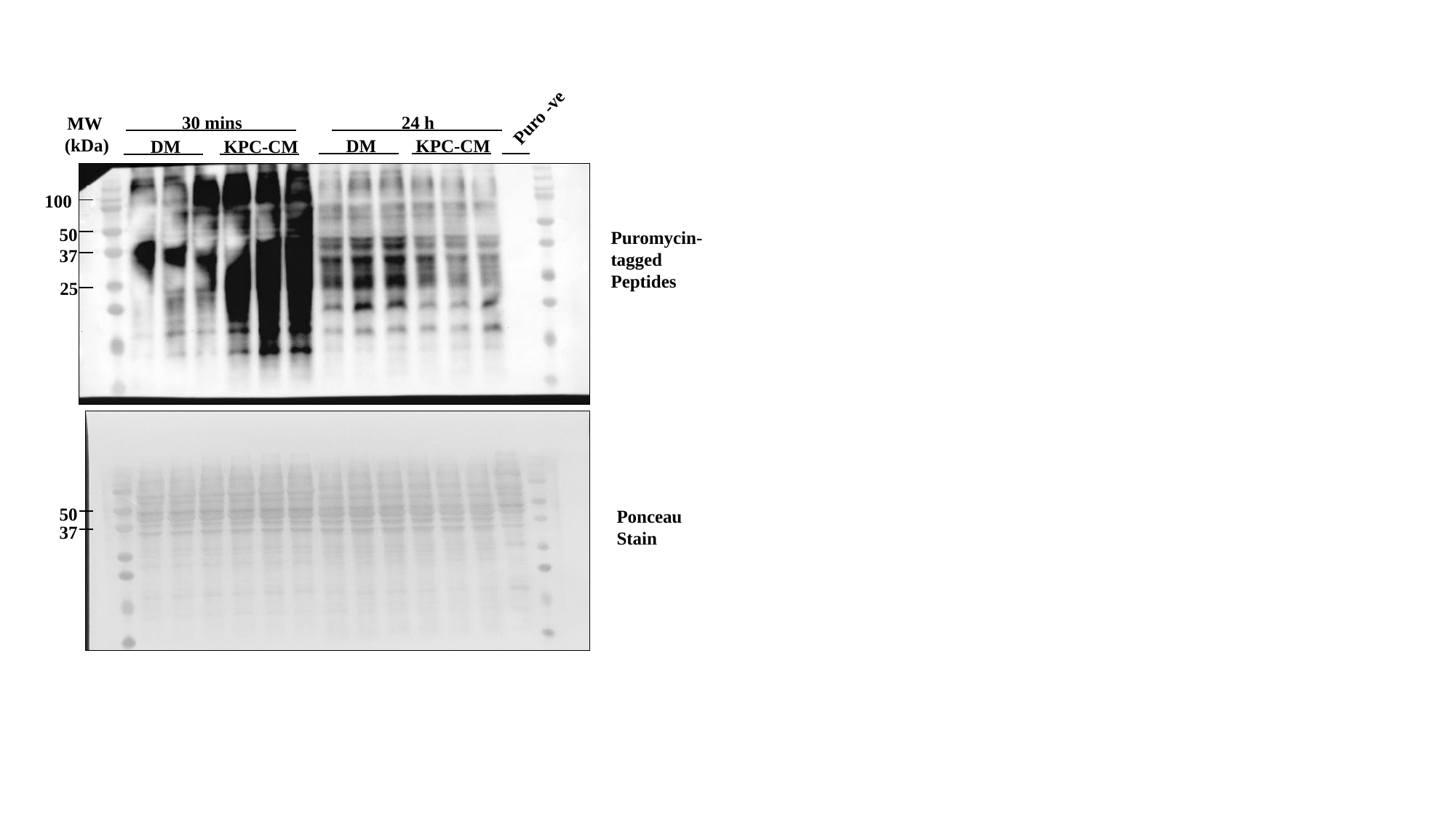

Puro -ve
30 mins
24 h
MW
(kDa)
DM
KPC-CM
DM
KPC-CM
100
50
Puromycin-tagged
Peptides
37
25
50
Ponceau
Stain
37

Supplement: Supplementary file 12 — Figure EV1 Source Data [file 44321_2025_337_MOESM12_ESM.zip › Figure EV1/Fig EV1I and K_Western blot/Fig EV1K-LWestern blot and quantification/Fig EV1K Western blot.pptx]

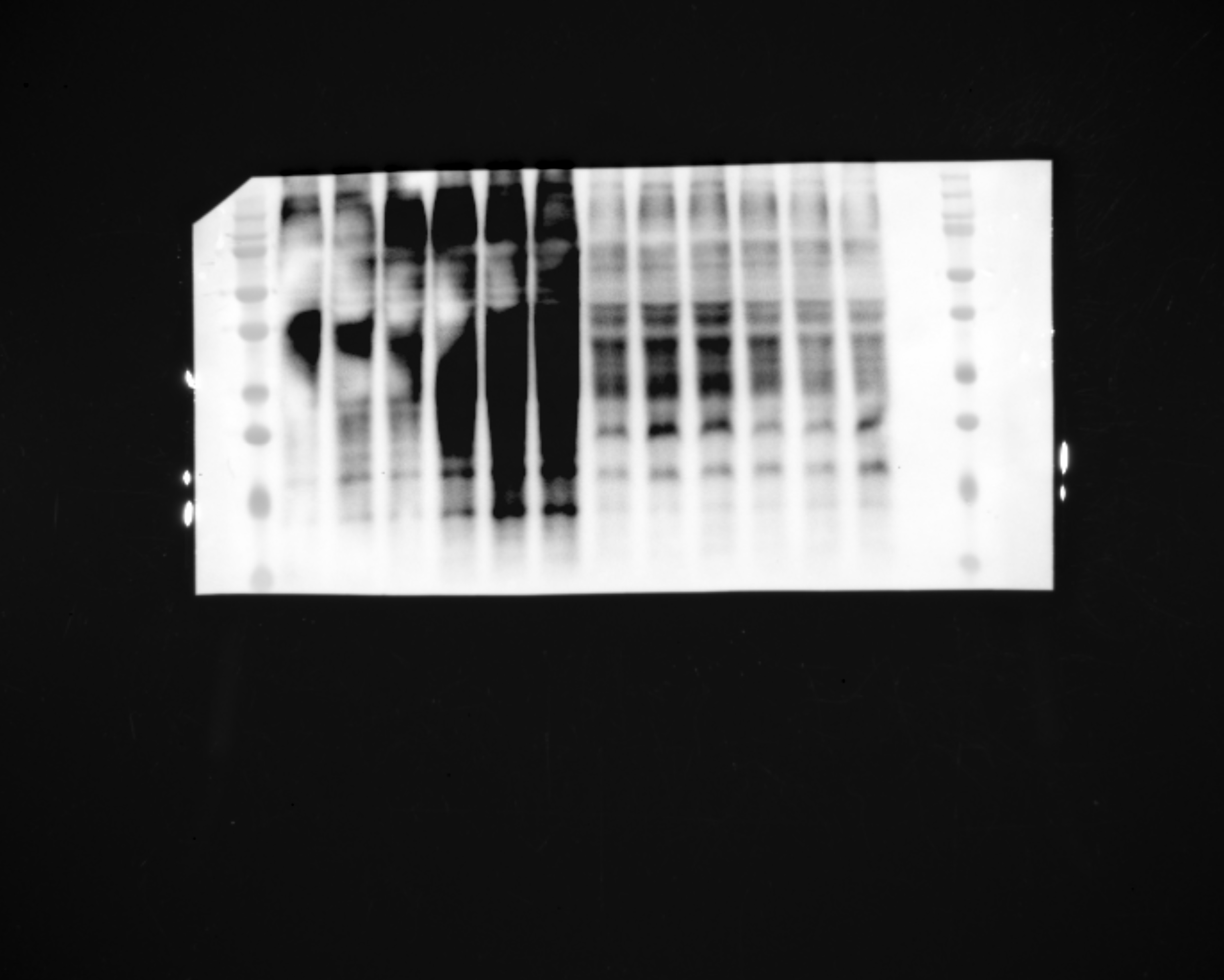

Supplement: Supplementary file 12 — Figure EV1 Source Data [file 44321_2025_337_MOESM12_ESM.zip › Figure EV1/Fig EV1I and K_Western blot/Fig EV1K-LWestern blot and quantification/Western Puromycin-tagged peptides.tif]

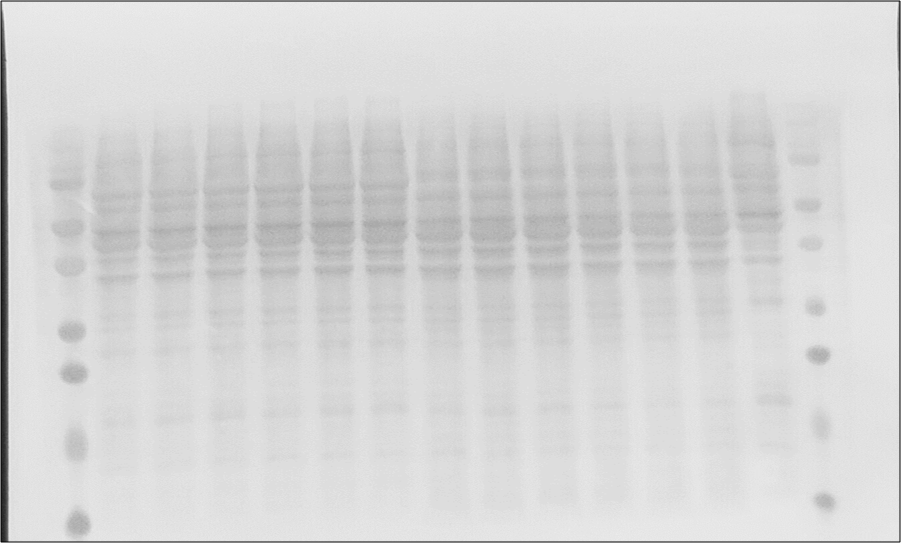

Supplement: Supplementary file 12 — Figure EV1 Source Data [file 44321_2025_337_MOESM12_ESM.zip › Figure EV1/Fig EV1I and K_Western blot/Fig EV1K-LWestern blot and quantification/Western Ponceau Stain.tif]

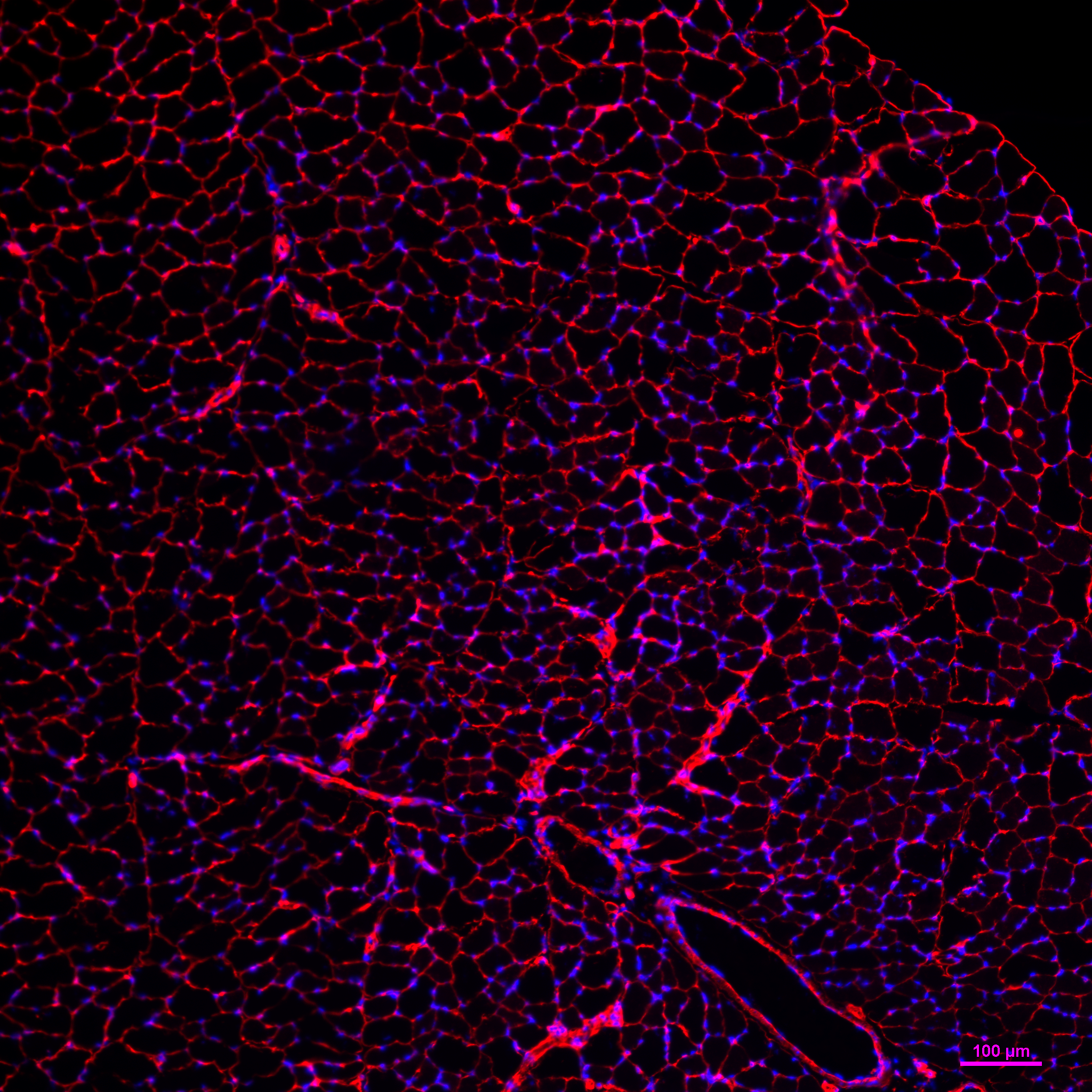

Supplement: Supplementary file 13 — Figure EV2 Source Data [file 44321_2025_337_MOESM13_ESM.zip › Figure EV2/Fig EV2C_TA muscle_Laminin DAPI whole section images/Xbp1-flfl-KPC-1.png]

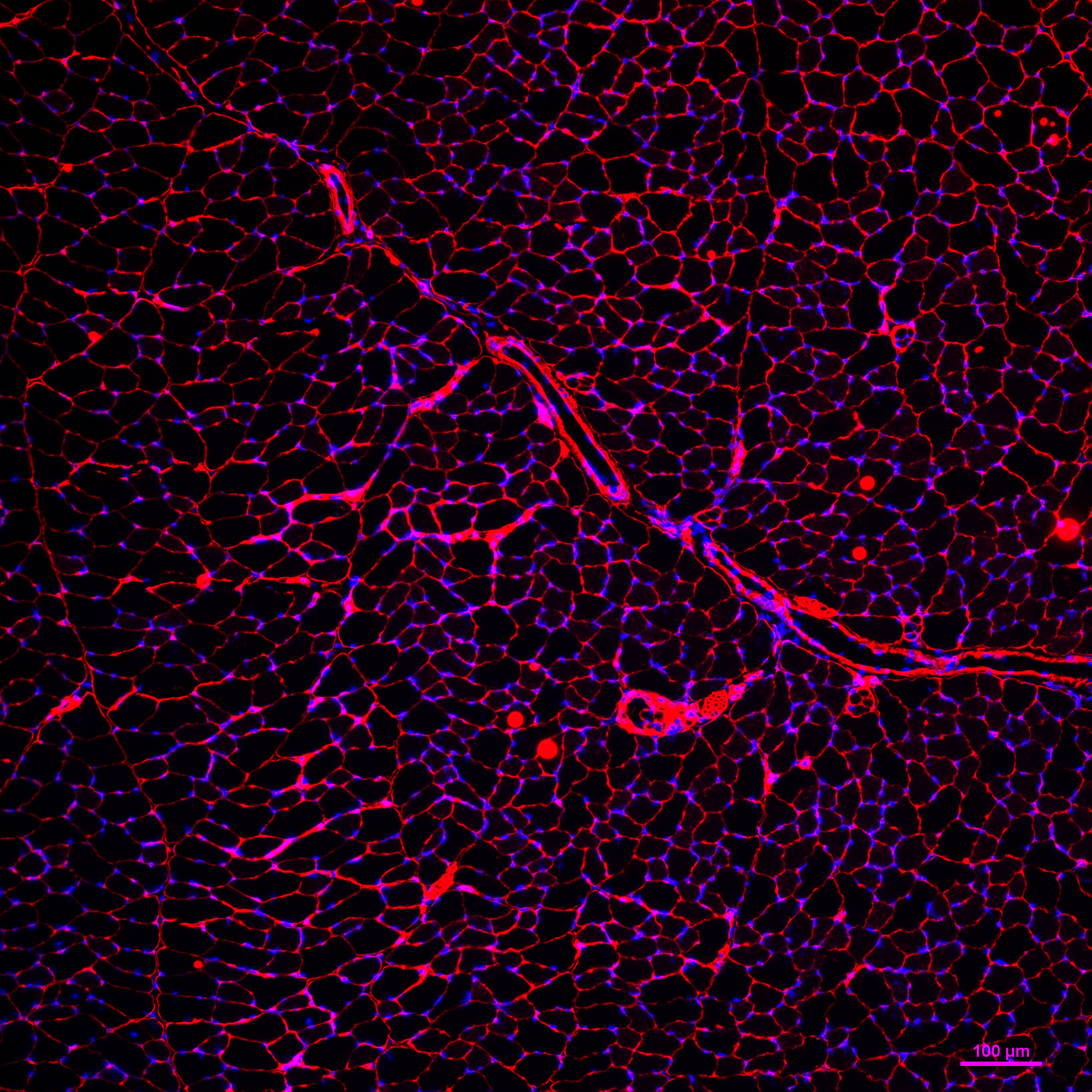

Supplement: Supplementary file 13 — Figure EV2 Source Data [file 44321_2025_337_MOESM13_ESM.zip › Figure EV2/Fig EV2C_TA muscle_Laminin DAPI whole section images/Xbp1-flfl-KPC-2.png]

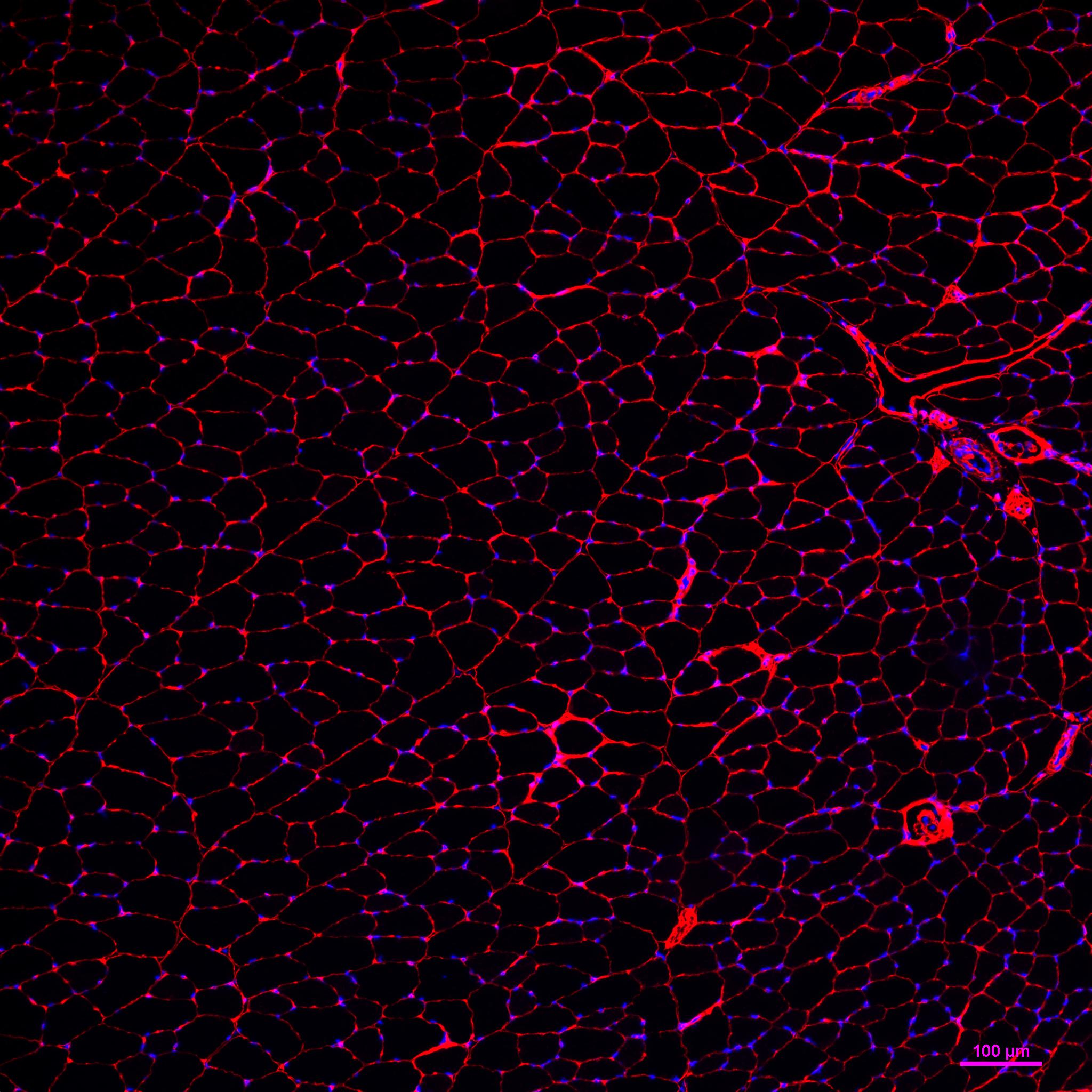

Supplement: Supplementary file 13 — Figure EV2 Source Data [file 44321_2025_337_MOESM13_ESM.zip › Figure EV2/Fig EV2C_TA muscle_Laminin DAPI whole section images/Xbp1-flfl-PBS-1.png]

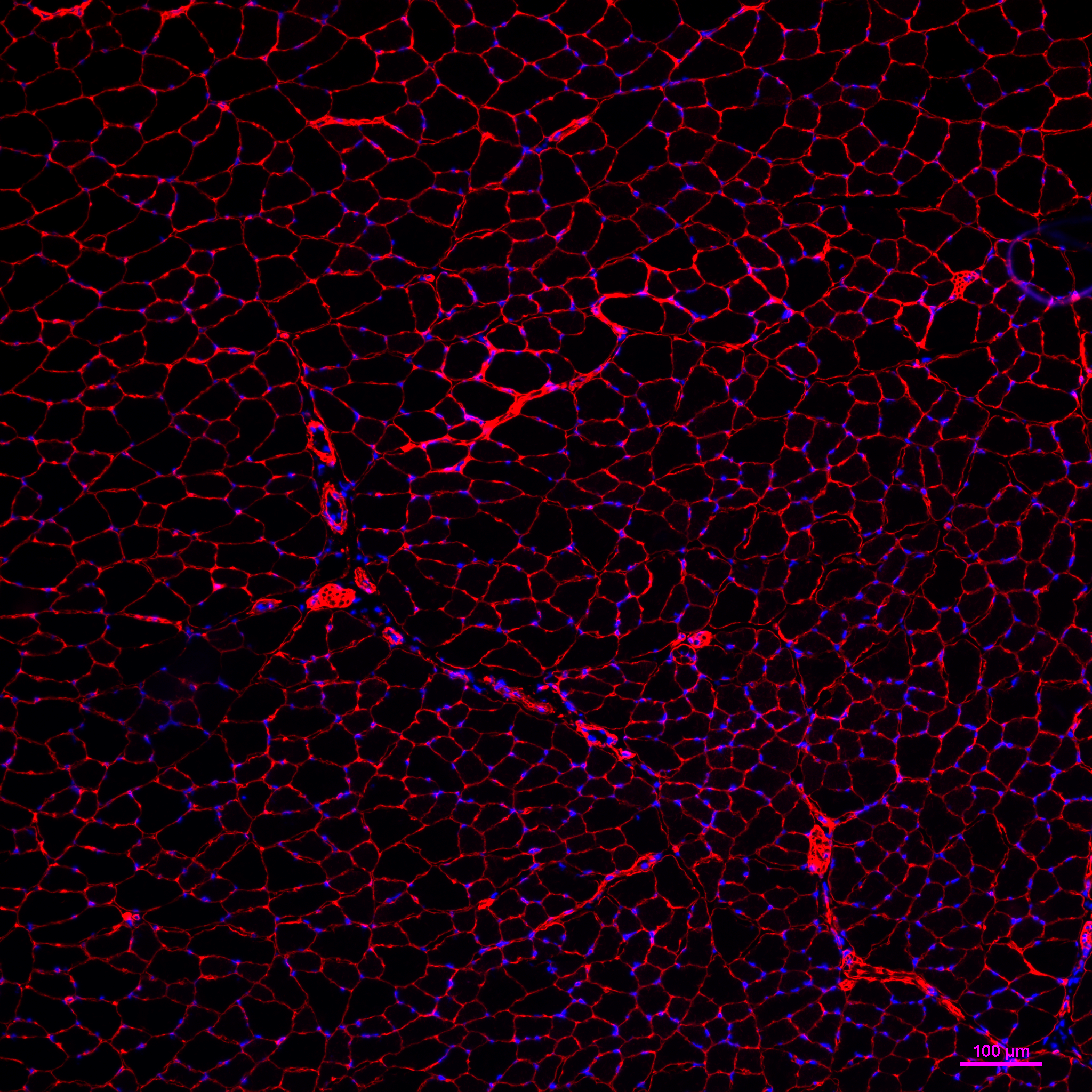

Supplement: Supplementary file 13 — Figure EV2 Source Data [file 44321_2025_337_MOESM13_ESM.zip › Figure EV2/Fig EV2C_TA muscle_Laminin DAPI whole section images/Xbp1-flfl-PBS-3.png]

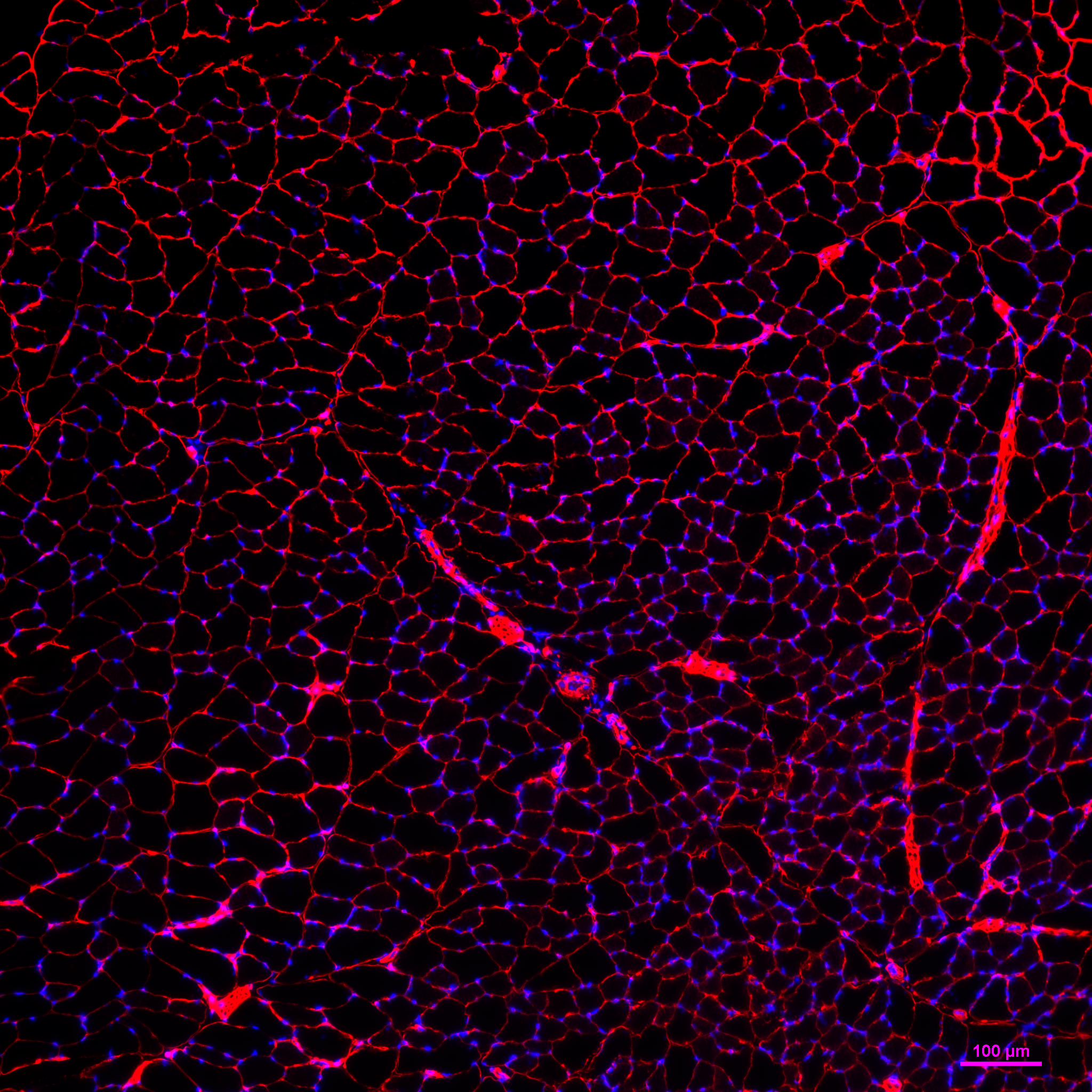

Supplement: Supplementary file 13 — Figure EV2 Source Data [file 44321_2025_337_MOESM13_ESM.zip › Figure EV2/Fig EV2C_TA muscle_Laminin DAPI whole section images/Xbp1-mKO-KPC-1.png]

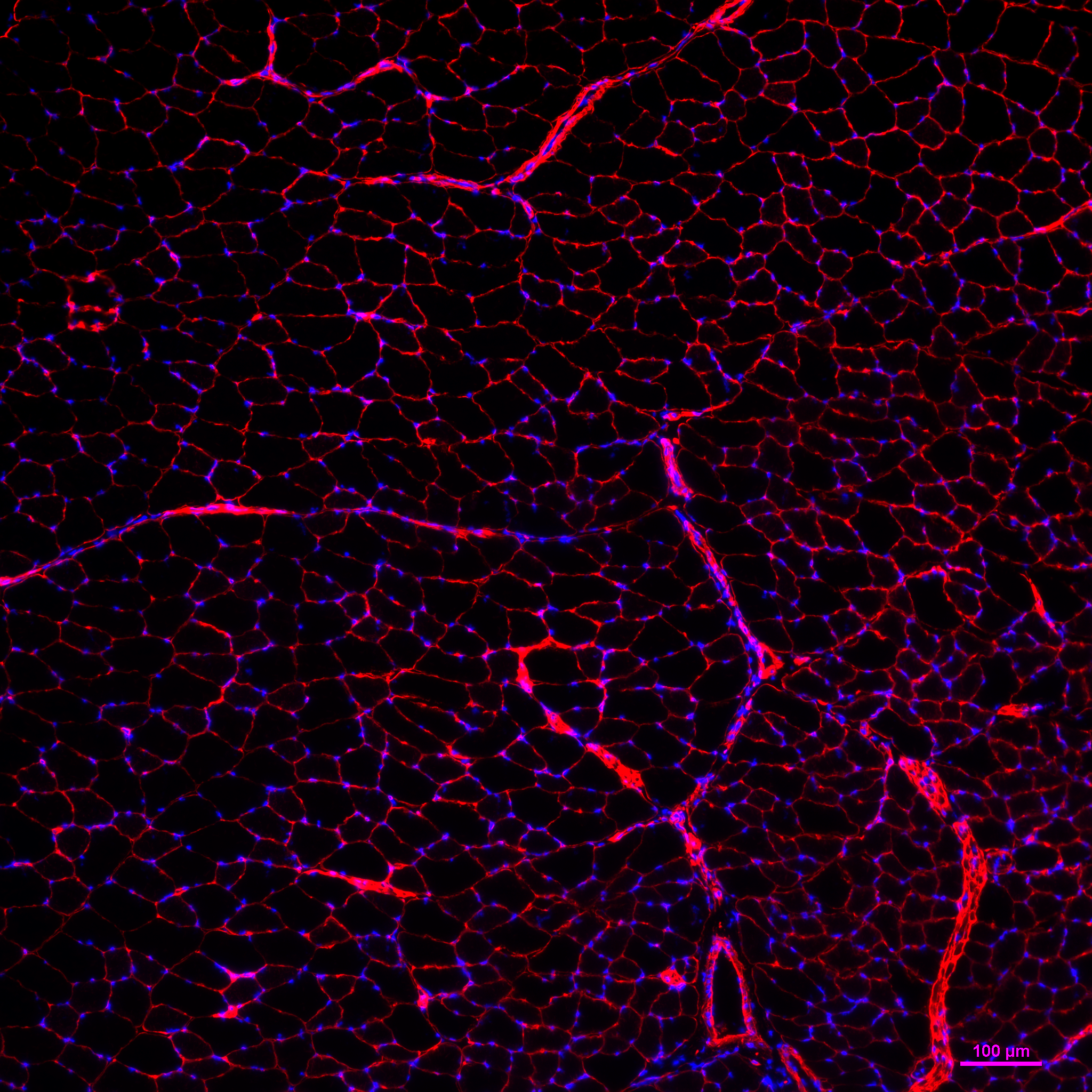

Supplement: Supplementary file 13 — Figure EV2 Source Data [file 44321_2025_337_MOESM13_ESM.zip › Figure EV2/Fig EV2C_TA muscle_Laminin DAPI whole section images/Xbp1-mKO-KPC-2.png]

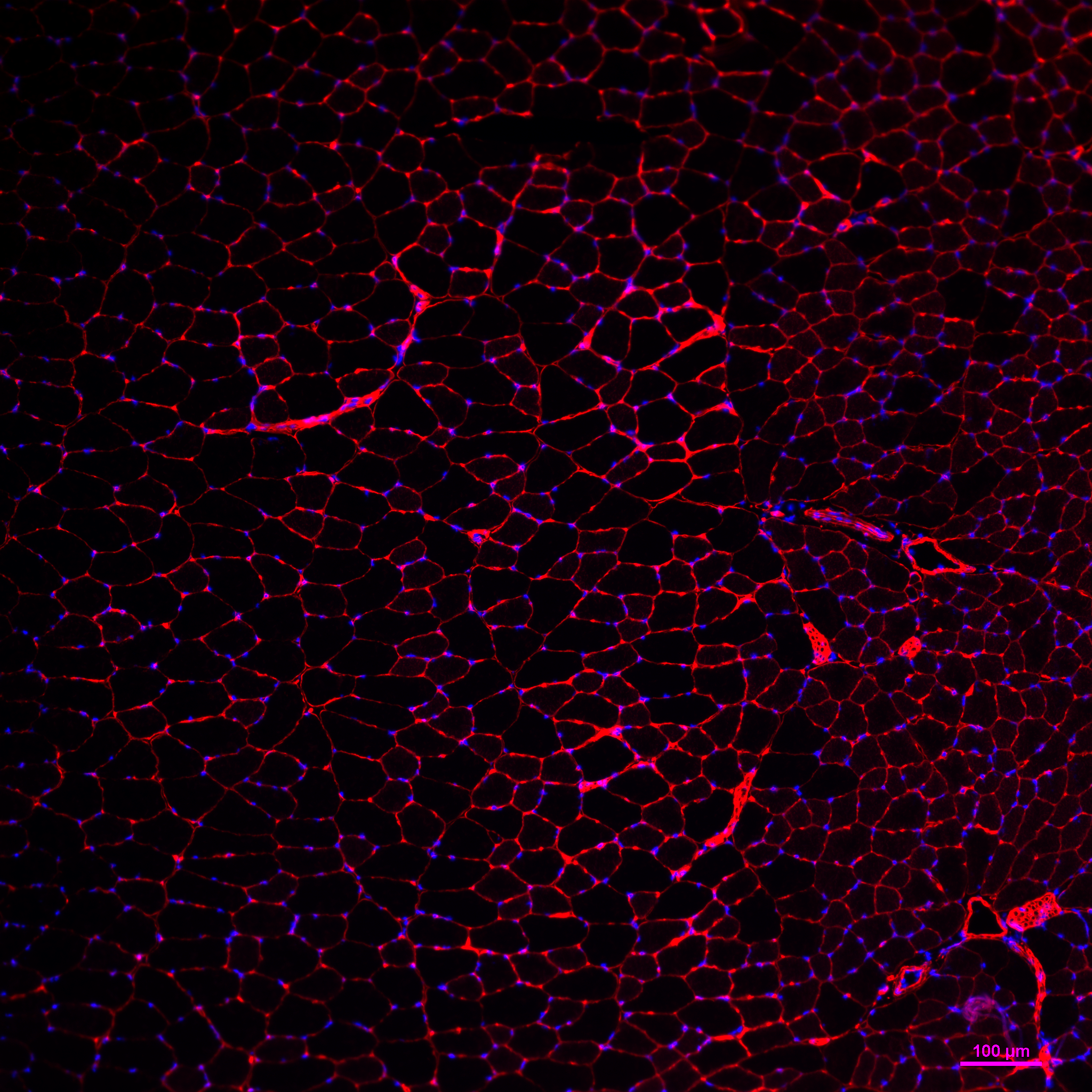

Supplement: Supplementary file 13 — Figure EV2 Source Data [file 44321_2025_337_MOESM13_ESM.zip › Figure EV2/Fig EV2C_TA muscle_Laminin DAPI whole section images/Xbp1-mKO-PBS-2.png]

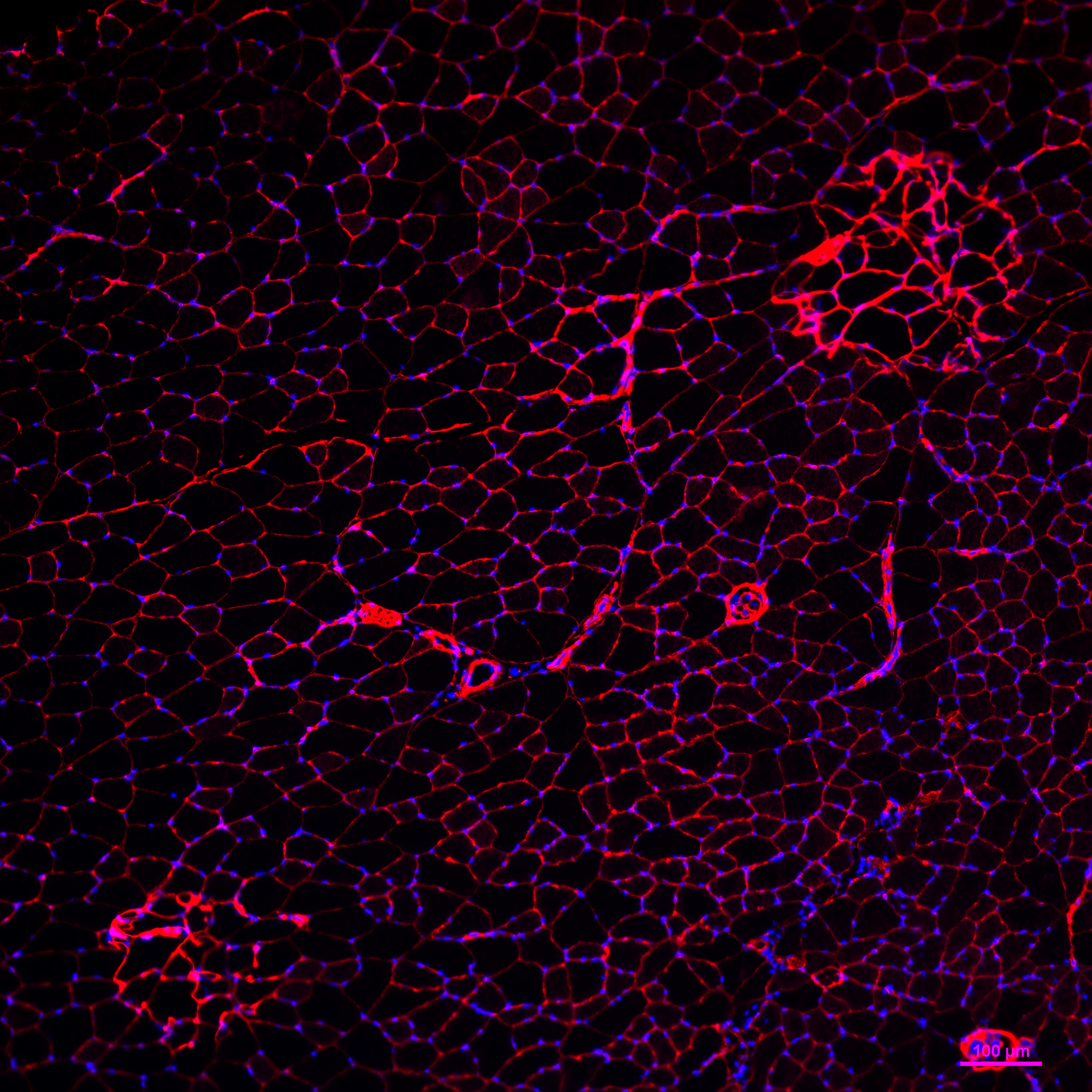

Supplement: Supplementary file 13 — Figure EV2 Source Data [file 44321_2025_337_MOESM13_ESM.zip › Figure EV2/Fig EV2C_TA muscle_Laminin DAPI whole section images/Xbp1-mKO-PBS-3.png]

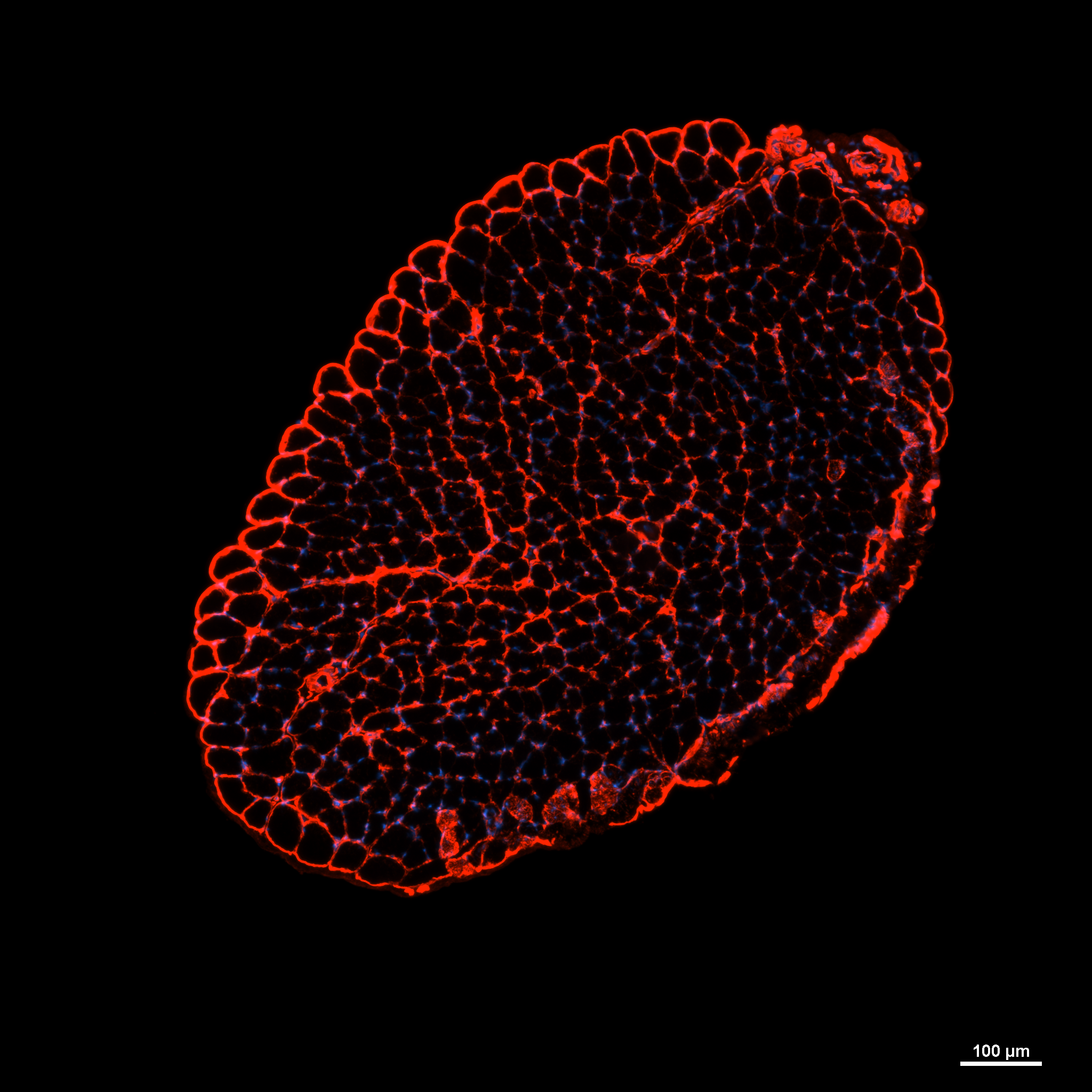

Supplement: Supplementary file 13 — Figure EV2 Source Data [file 44321_2025_337_MOESM13_ESM.zip › Figure EV2/Fig EV2D_Soleus muscle_Laminin DAPI whole section images/Xbp1-flfl-KPC-2.tif]

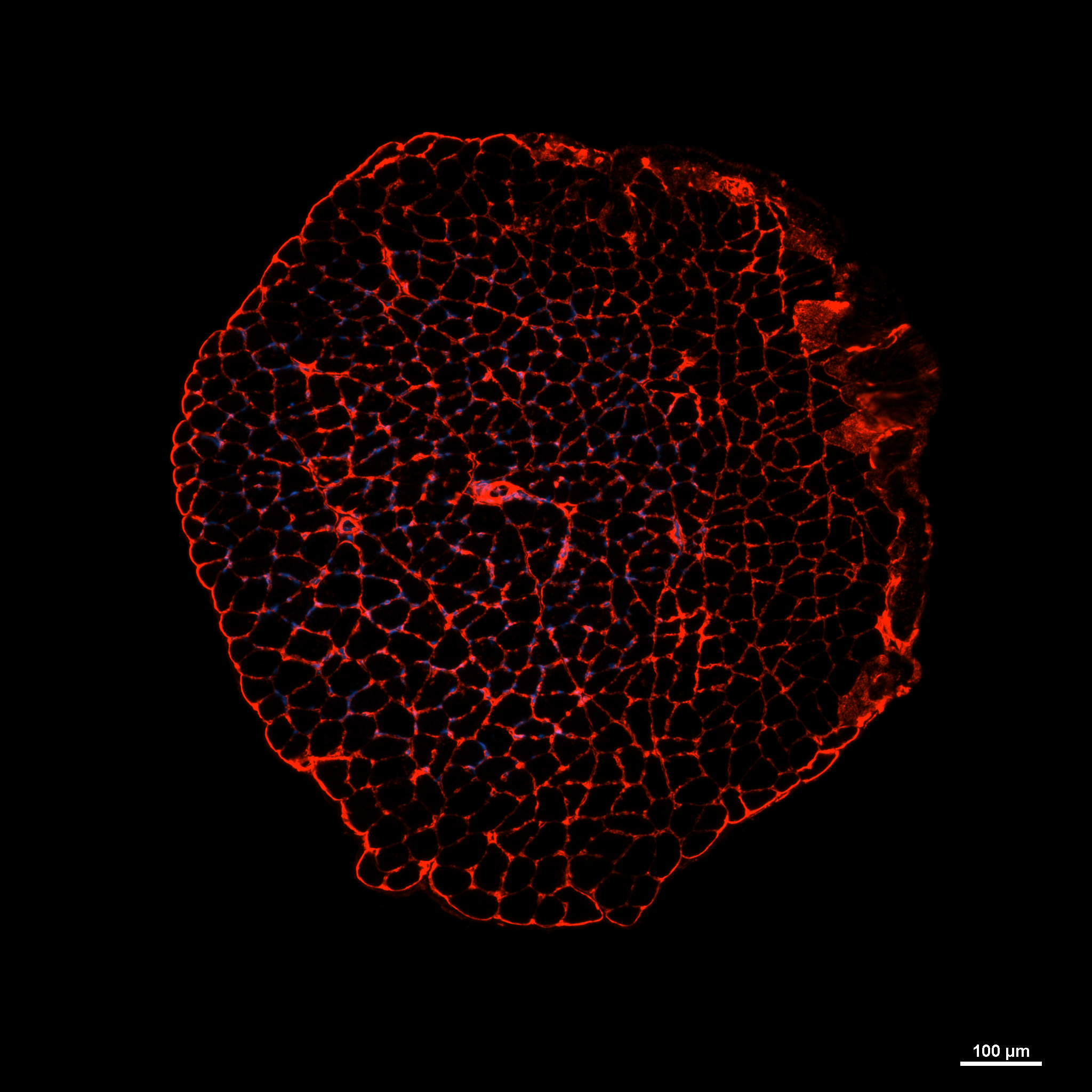

Supplement: Supplementary file 13 — Figure EV2 Source Data [file 44321_2025_337_MOESM13_ESM.zip › Figure EV2/Fig EV2D_Soleus muscle_Laminin DAPI whole section images/Xbp1-flfl-KPC-3.tif]

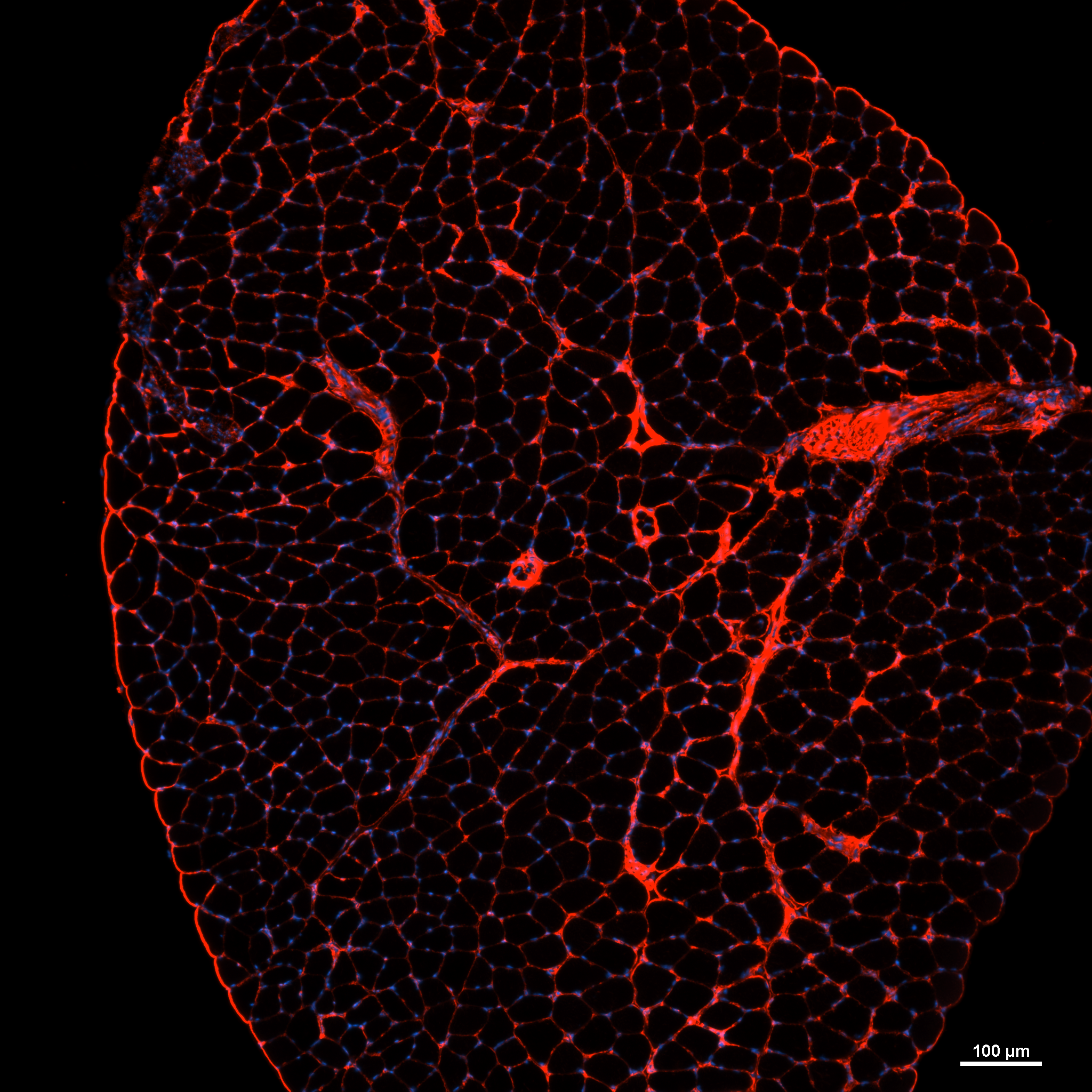

Supplement: Supplementary file 13 — Figure EV2 Source Data [file 44321_2025_337_MOESM13_ESM.zip › Figure EV2/Fig EV2D_Soleus muscle_Laminin DAPI whole section images/Xbp1-flfl-PBS-1.tif]

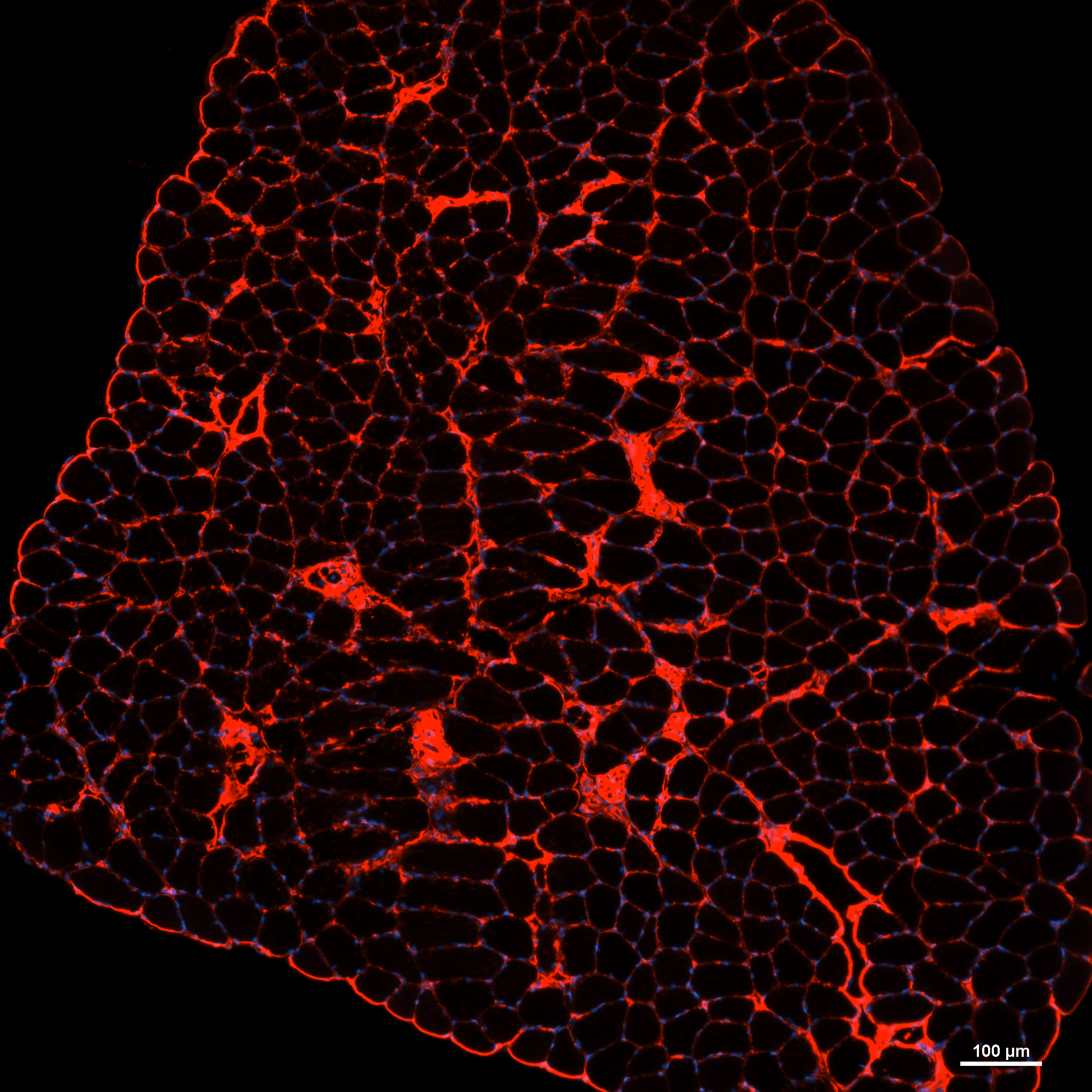

Supplement: Supplementary file 13 — Figure EV2 Source Data [file 44321_2025_337_MOESM13_ESM.zip › Figure EV2/Fig EV2D_Soleus muscle_Laminin DAPI whole section images/Xbp1-flfl-PBS-3.tif]

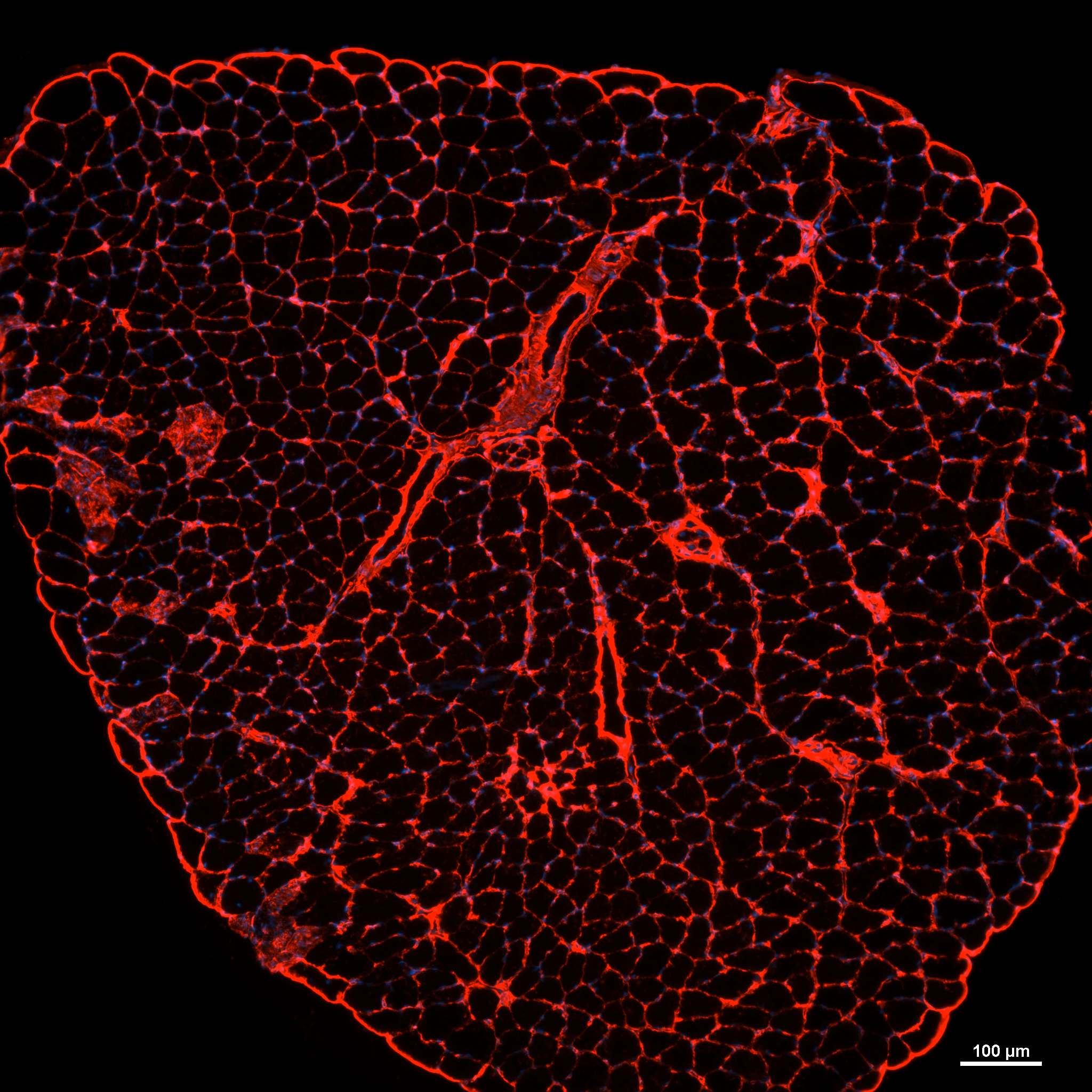

Supplement: Supplementary file 13 — Figure EV2 Source Data [file 44321_2025_337_MOESM13_ESM.zip › Figure EV2/Fig EV2D_Soleus muscle_Laminin DAPI whole section images/Xbp1-mKO-KPC-2.tif]

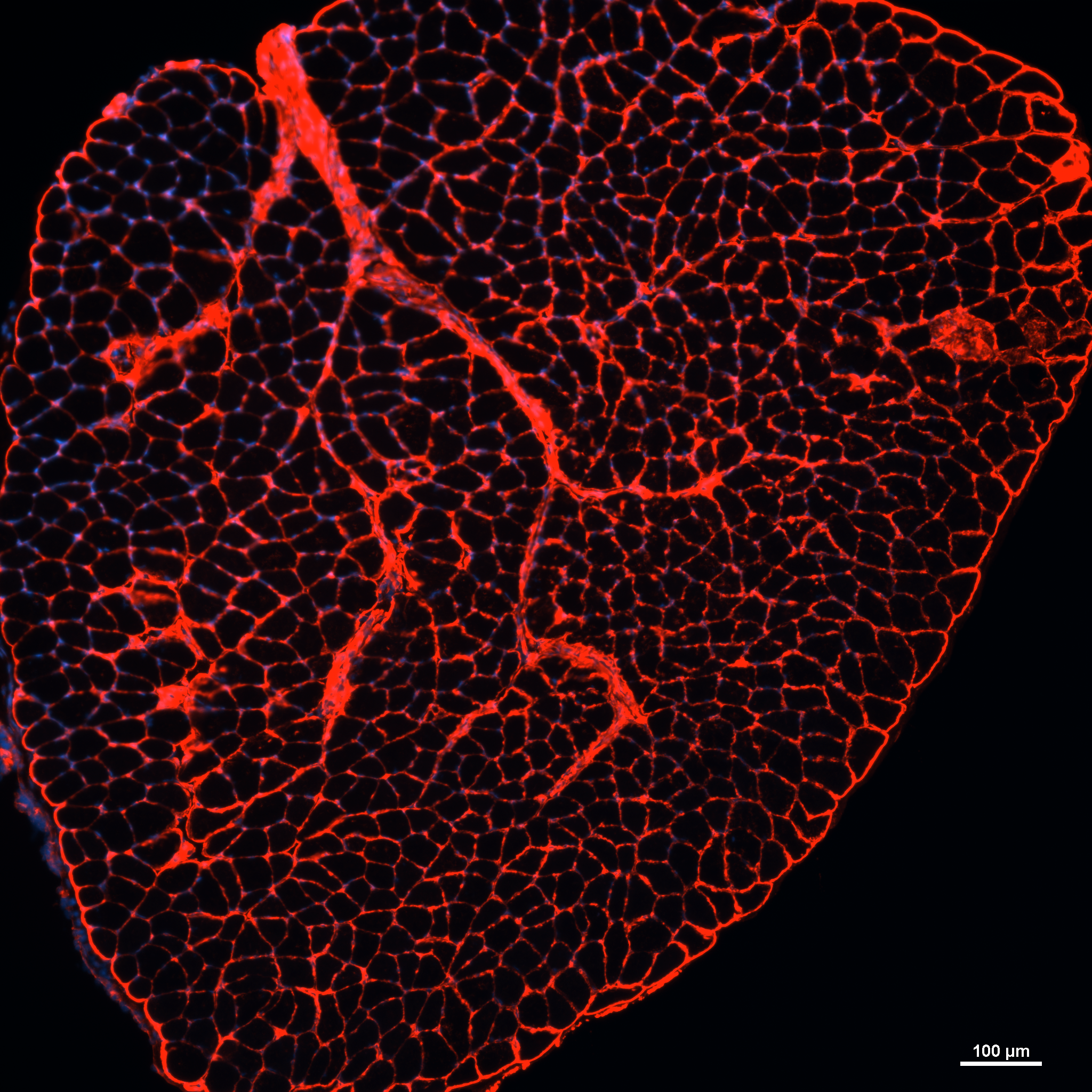

Supplement: Supplementary file 13 — Figure EV2 Source Data [file 44321_2025_337_MOESM13_ESM.zip › Figure EV2/Fig EV2D_Soleus muscle_Laminin DAPI whole section images/Xbp1-mKO-KPC-3.tif]

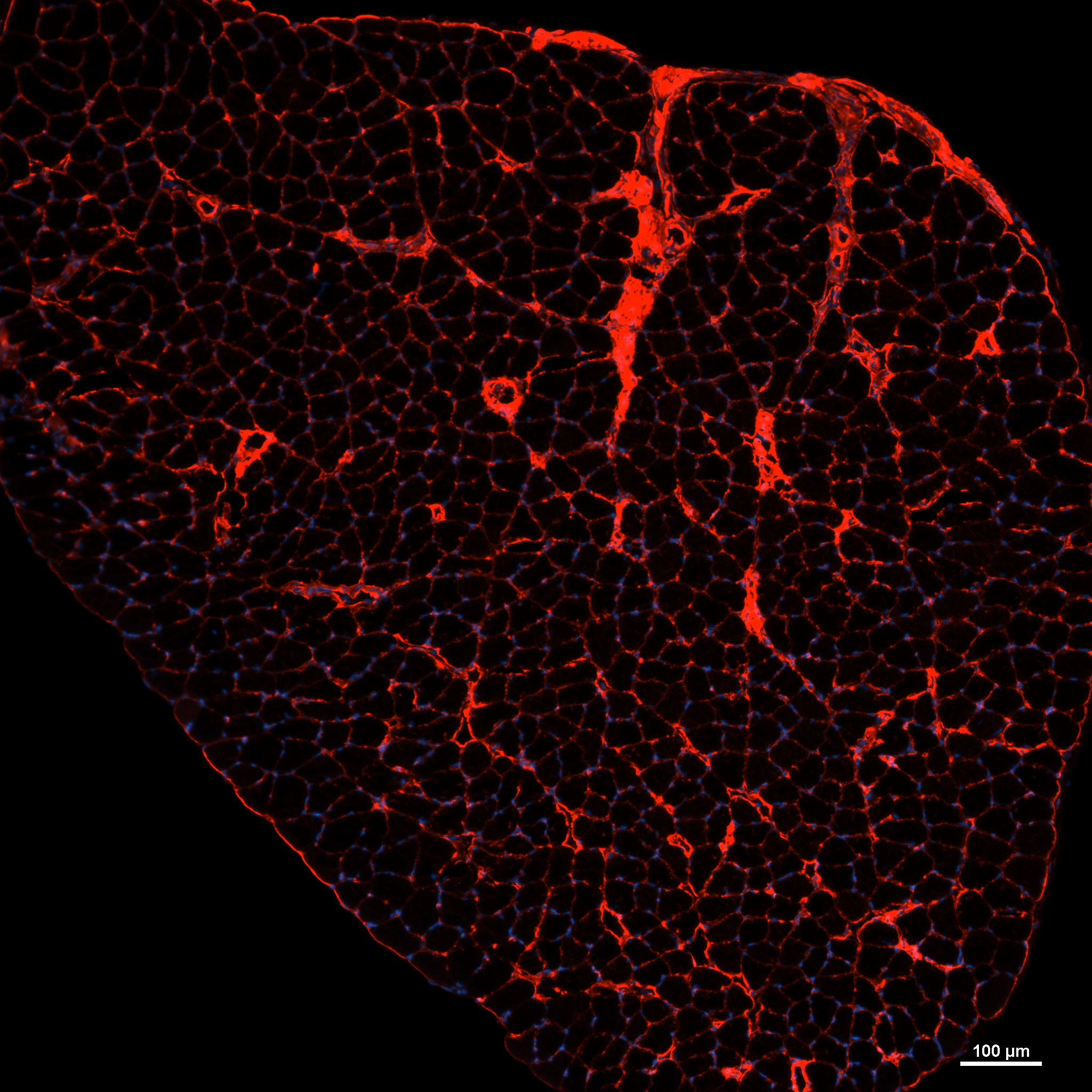

Supplement: Supplementary file 13 — Figure EV2 Source Data [file 44321_2025_337_MOESM13_ESM.zip › Figure EV2/Fig EV2D_Soleus muscle_Laminin DAPI whole section images/Xbp1-mKO-PBS-2.tif]

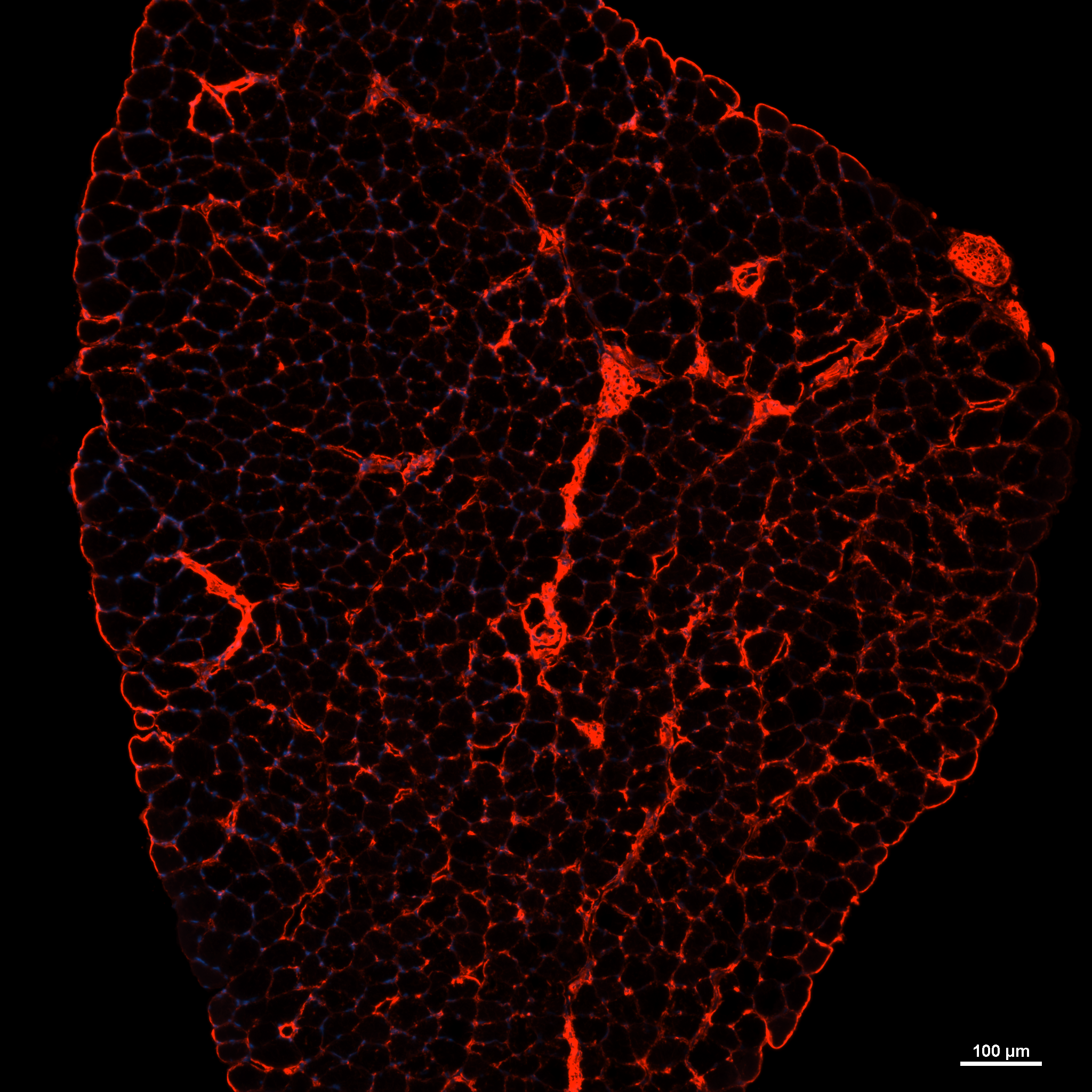

Supplement: Supplementary file 13 — Figure EV2 Source Data [file 44321_2025_337_MOESM13_ESM.zip › Figure EV2/Fig EV2D_Soleus muscle_Laminin DAPI whole section images/Xbp1-mKO-PBS-3.tif]

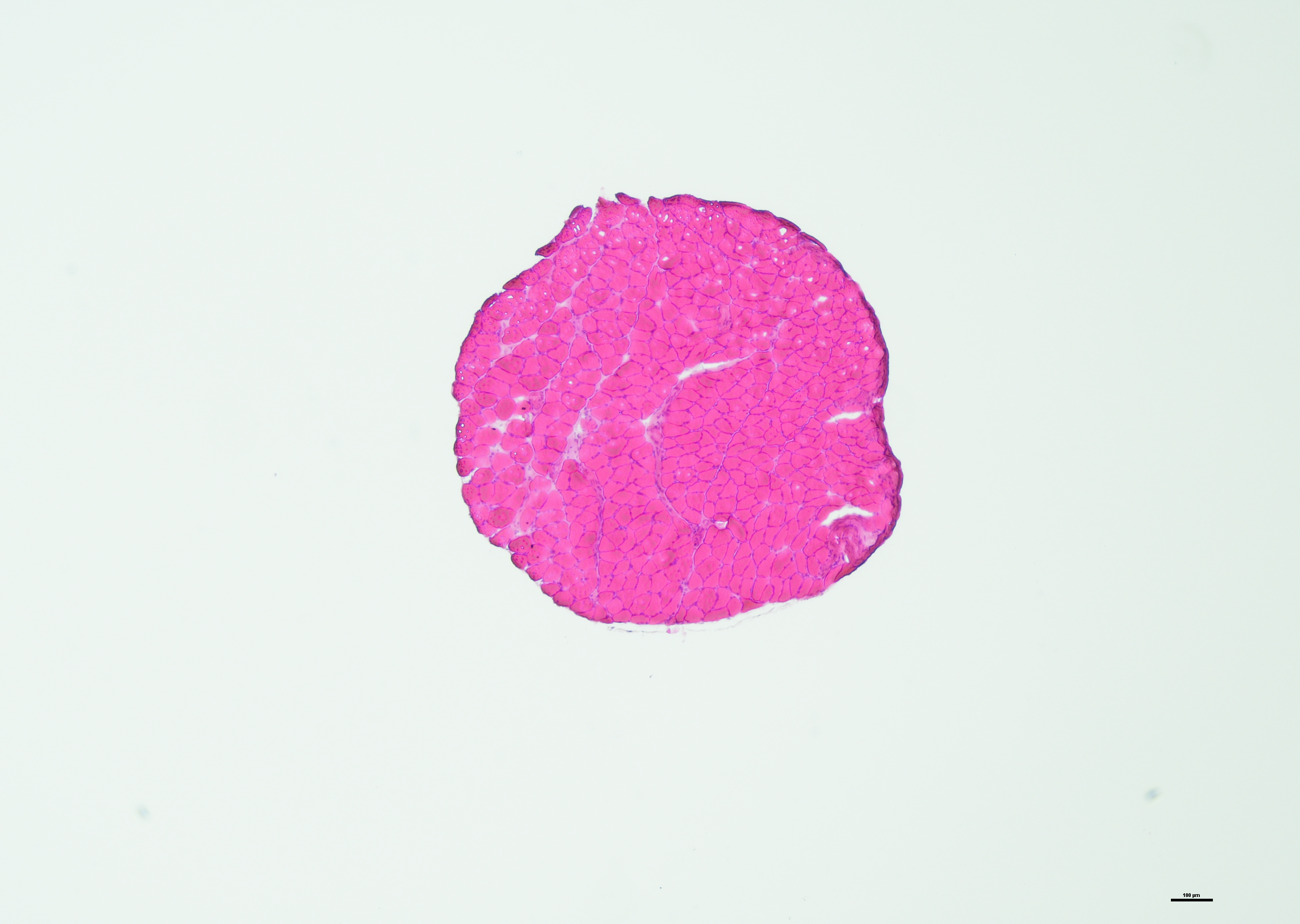

Supplement: Supplementary file 13 — Figure EV2 Source Data [file 44321_2025_337_MOESM13_ESM.zip › Figure EV2/Fig EV2E_TA and Soleus muscle_HE staining/Fig EV2E soleus muscle_HE staining_Representative images/Xbp1-flfl-KPC.tif]

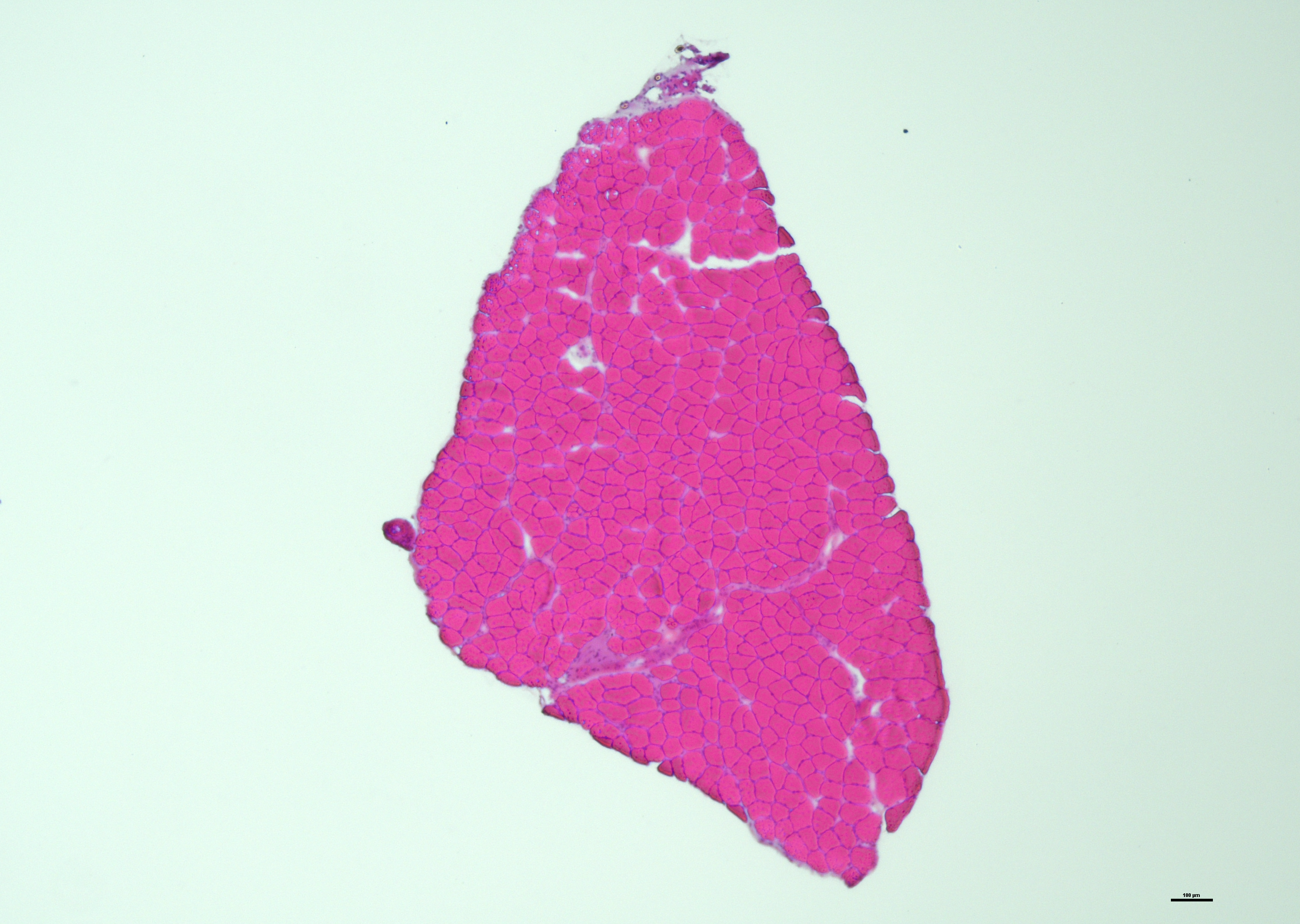

Supplement: Supplementary file 13 — Figure EV2 Source Data [file 44321_2025_337_MOESM13_ESM.zip › Figure EV2/Fig EV2E_TA and Soleus muscle_HE staining/Fig EV2E soleus muscle_HE staining_Representative images/Xbp1-flfl-PBS.tif]

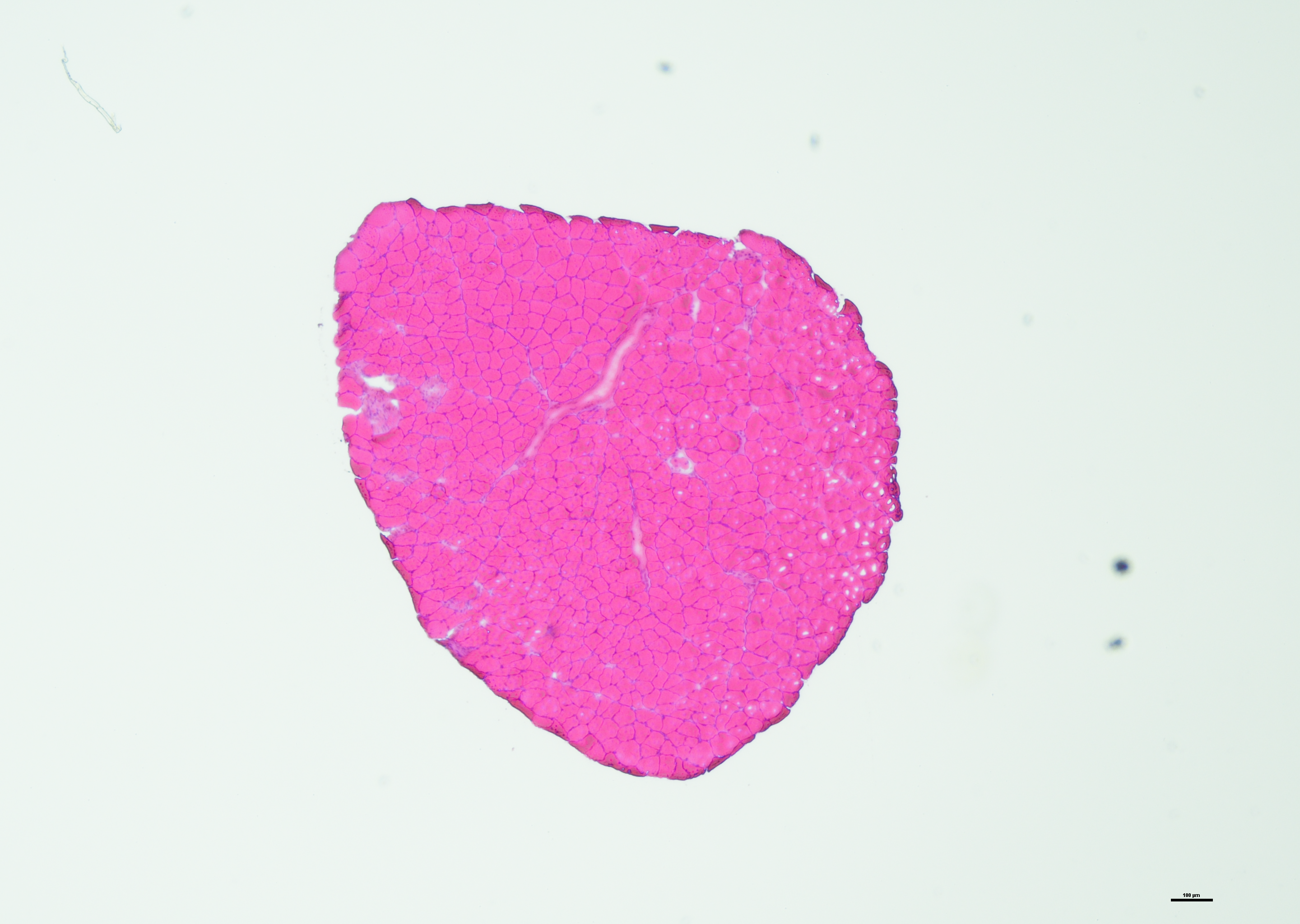

Supplement: Supplementary file 13 — Figure EV2 Source Data [file 44321_2025_337_MOESM13_ESM.zip › Figure EV2/Fig EV2E_TA and Soleus muscle_HE staining/Fig EV2E soleus muscle_HE staining_Representative images/Xbp1-mko-KPC.tif]

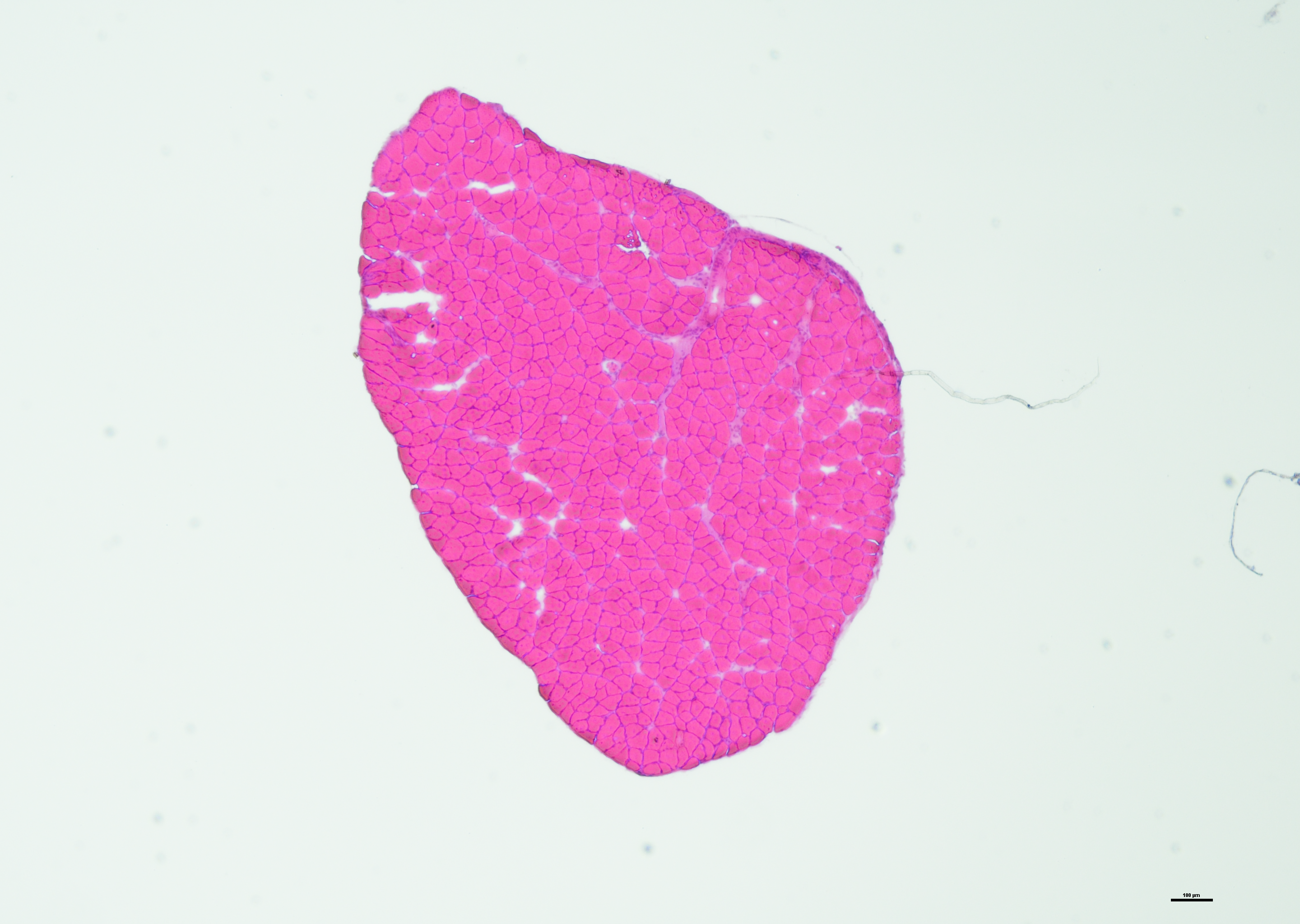

Supplement: Supplementary file 13 — Figure EV2 Source Data [file 44321_2025_337_MOESM13_ESM.zip › Figure EV2/Fig EV2E_TA and Soleus muscle_HE staining/Fig EV2E soleus muscle_HE staining_Representative images/Xbp1-mko-PBS.tif]

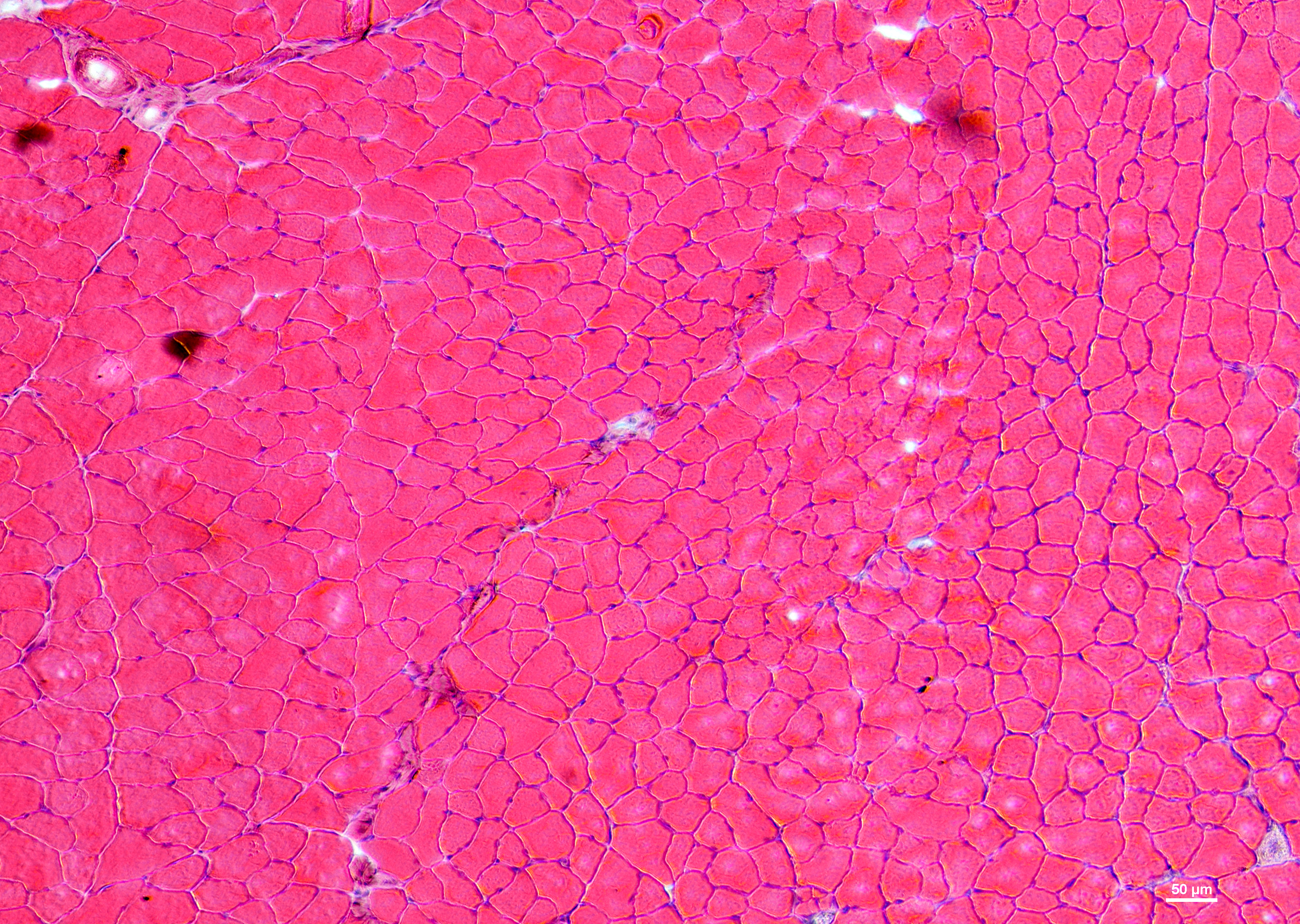

Supplement: Supplementary file 13 — Figure EV2 Source Data [file 44321_2025_337_MOESM13_ESM.zip › Figure EV2/Fig EV2E_TA and Soleus muscle_HE staining/Fig EV2E TA muscle_HE staining_Representative images/Xbp1-flfl-KPC.tif]

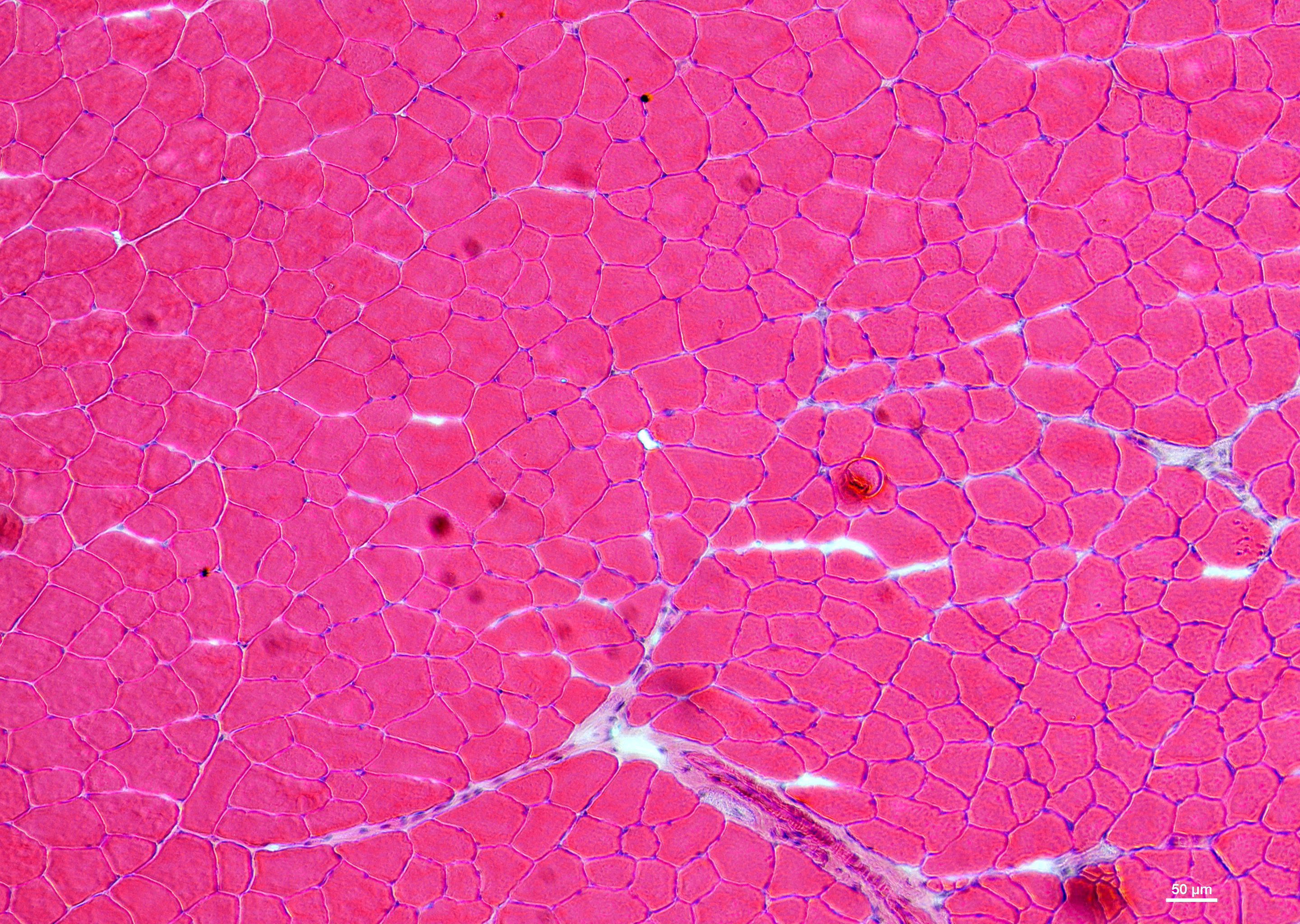

Supplement: Supplementary file 13 — Figure EV2 Source Data [file 44321_2025_337_MOESM13_ESM.zip › Figure EV2/Fig EV2E_TA and Soleus muscle_HE staining/Fig EV2E TA muscle_HE staining_Representative images/Xbp1-flfl-PBS.tif]

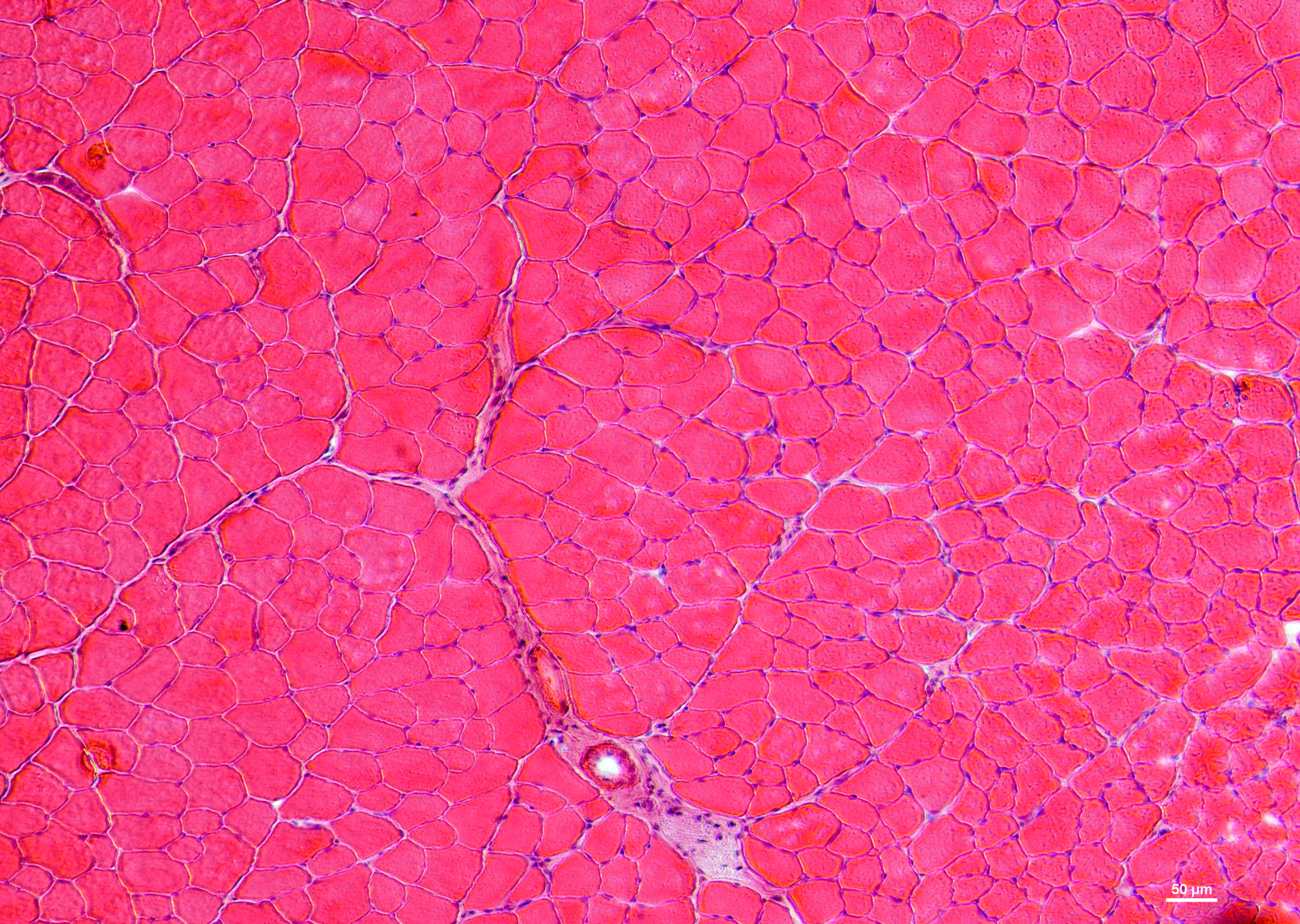

Supplement: Supplementary file 13 — Figure EV2 Source Data [file 44321_2025_337_MOESM13_ESM.zip › Figure EV2/Fig EV2E_TA and Soleus muscle_HE staining/Fig EV2E TA muscle_HE staining_Representative images/Xbp1-mKO-KPC.tif]

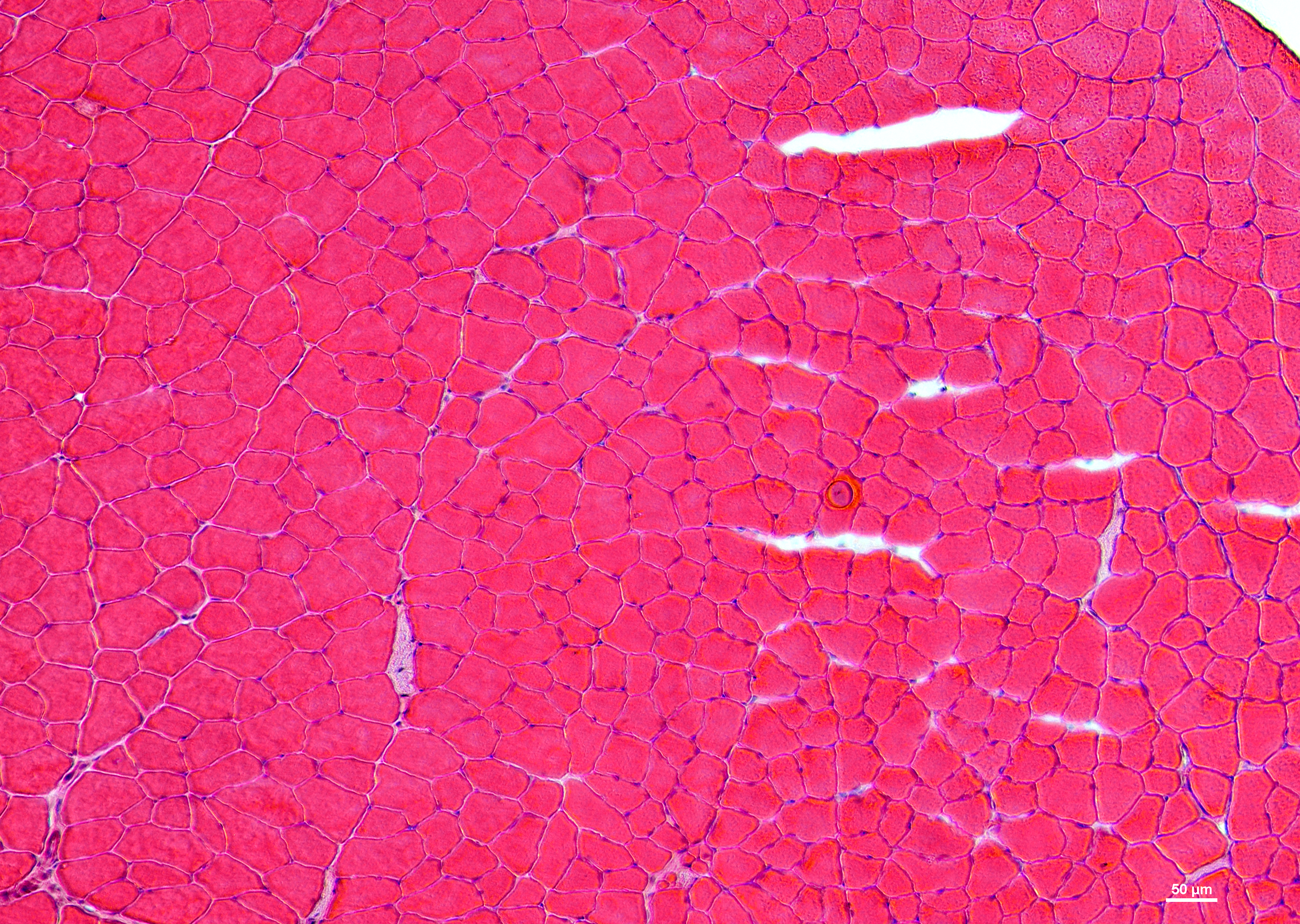

Supplement: Supplementary file 13 — Figure EV2 Source Data [file 44321_2025_337_MOESM13_ESM.zip › Figure EV2/Fig EV2E_TA and Soleus muscle_HE staining/Fig EV2E TA muscle_HE staining_Representative images/Xbp1-mKO-PBS.tif]

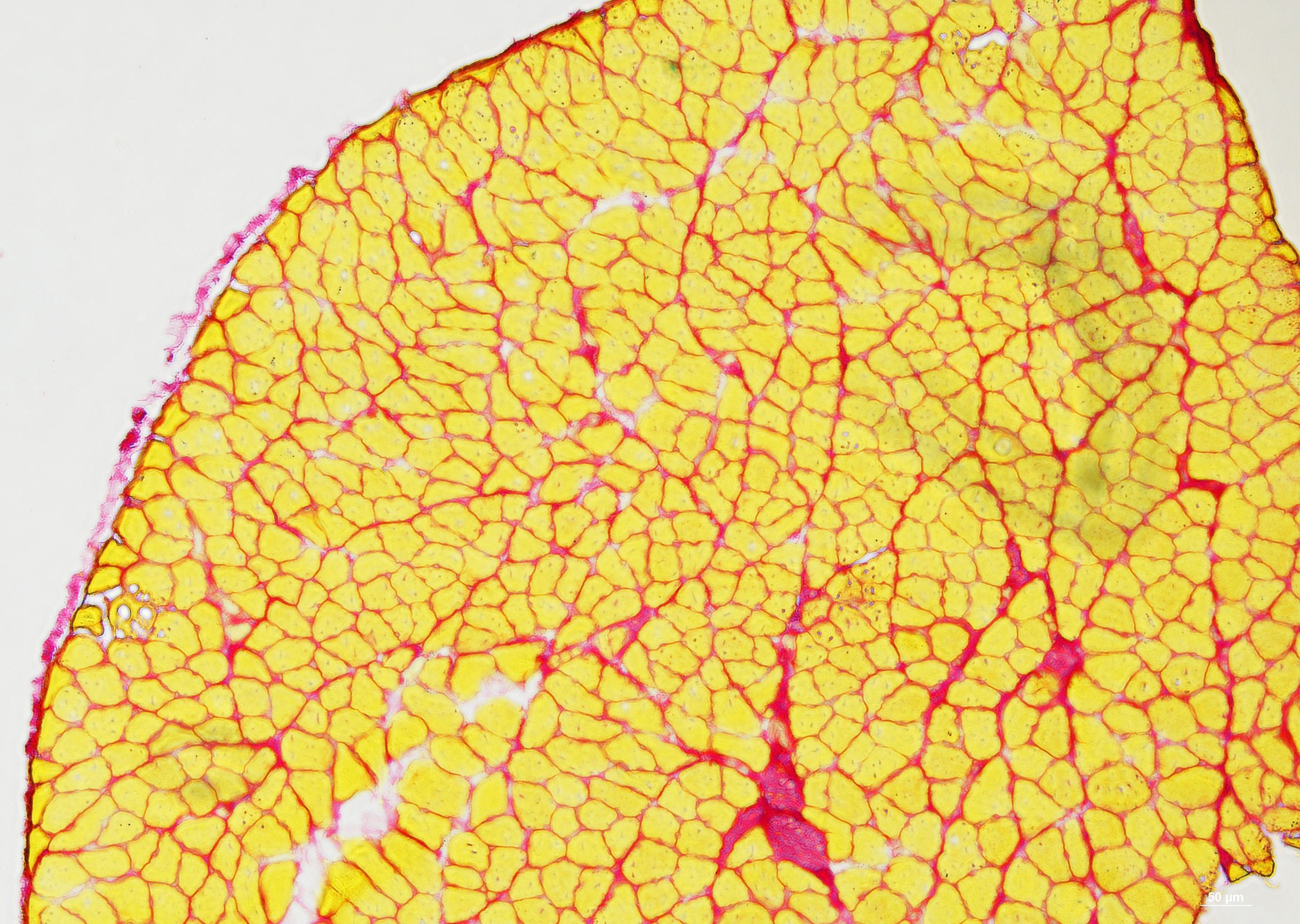

Supplement: Supplementary file 13 — Figure EV2 Source Data [file 44321_2025_337_MOESM13_ESM.zip › Figure EV2/Fig EV2F_Sirius Red staining_Representative images/Xbp1-flfl-KPC.tif]

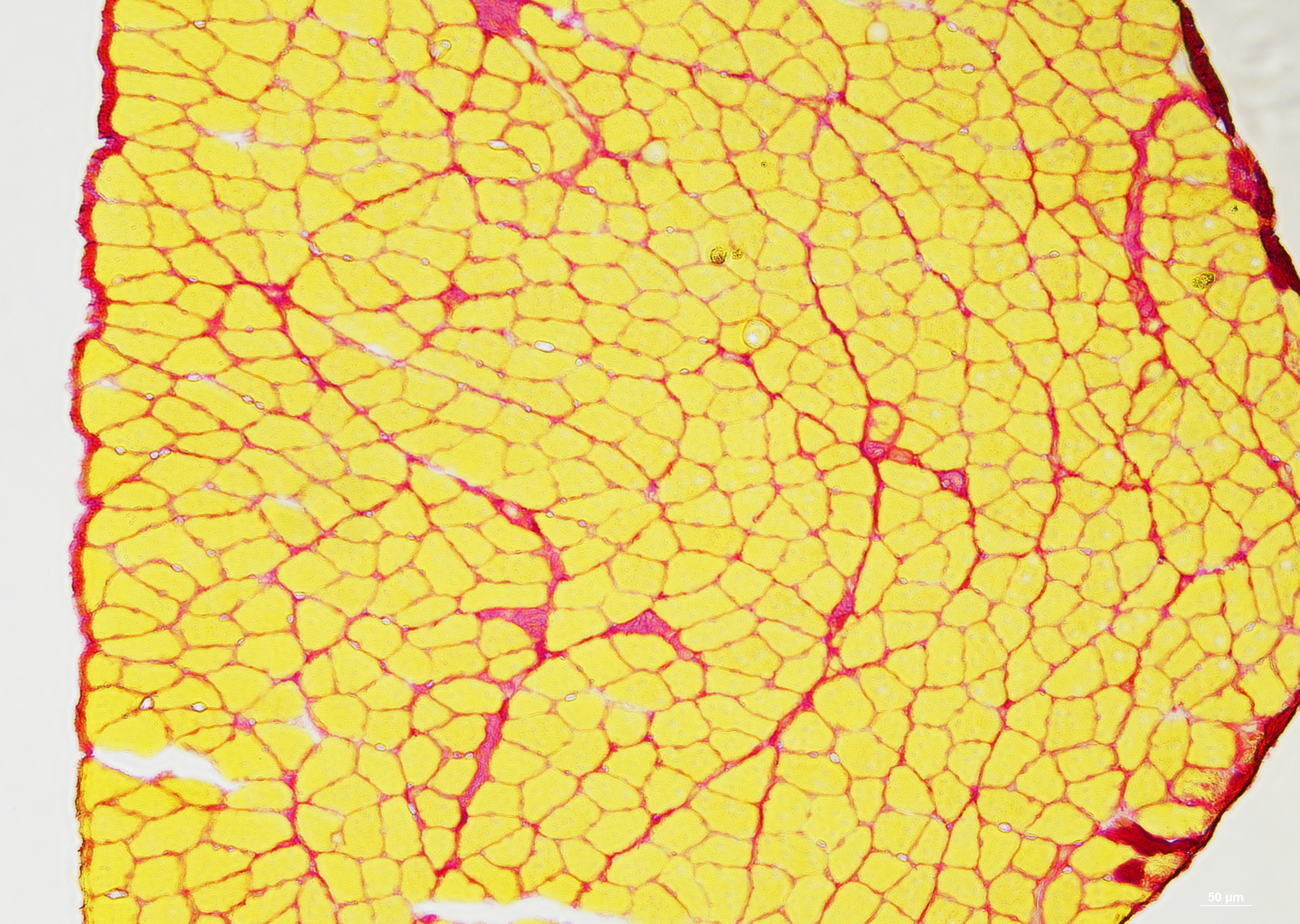

Supplement: Supplementary file 13 — Figure EV2 Source Data [file 44321_2025_337_MOESM13_ESM.zip › Figure EV2/Fig EV2F_Sirius Red staining_Representative images/Xbp1-flfl-PBS.tif]

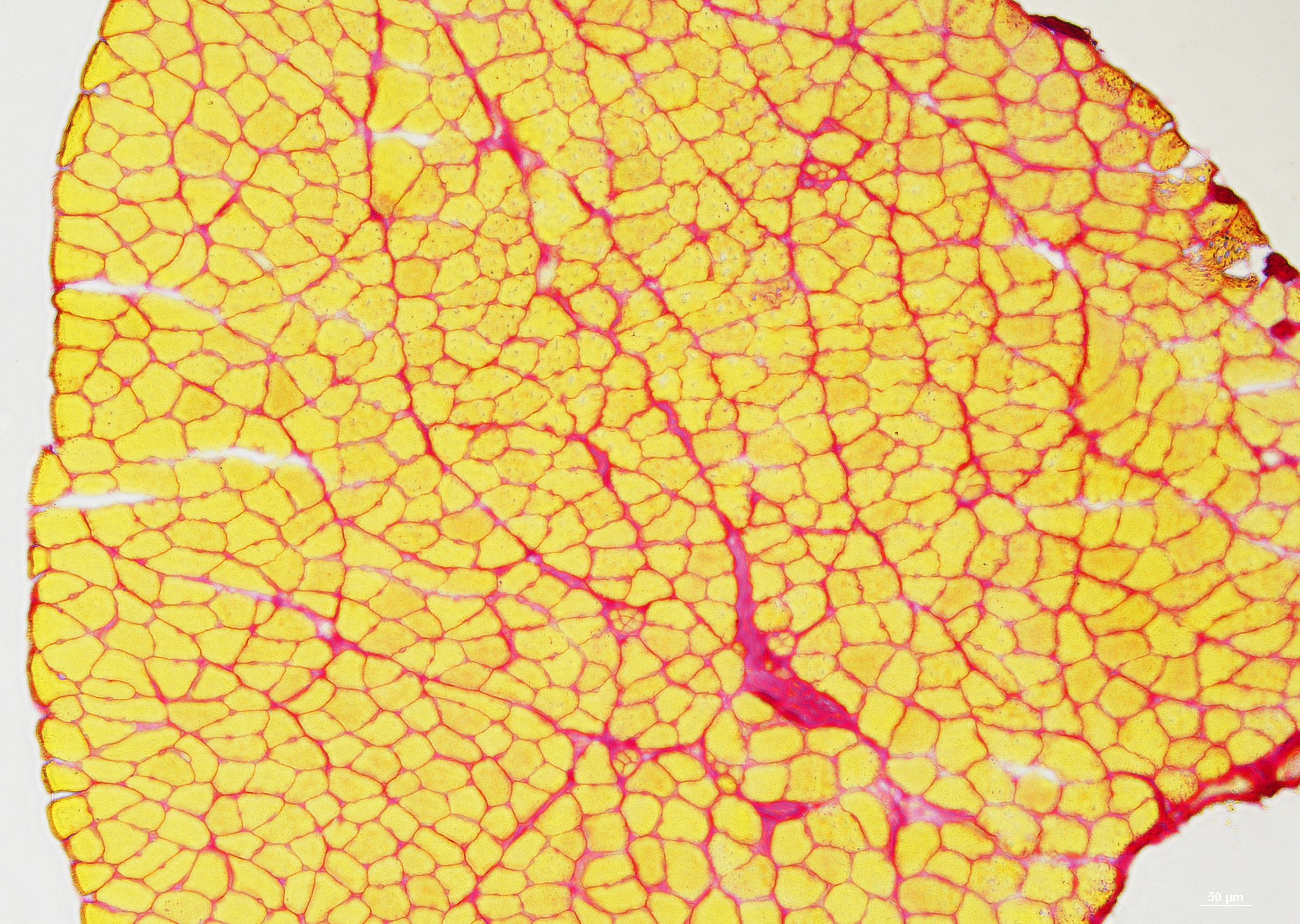

Supplement: Supplementary file 13 — Figure EV2 Source Data [file 44321_2025_337_MOESM13_ESM.zip › Figure EV2/Fig EV2F_Sirius Red staining_Representative images/Xbp1-mKO-KPC.tif]

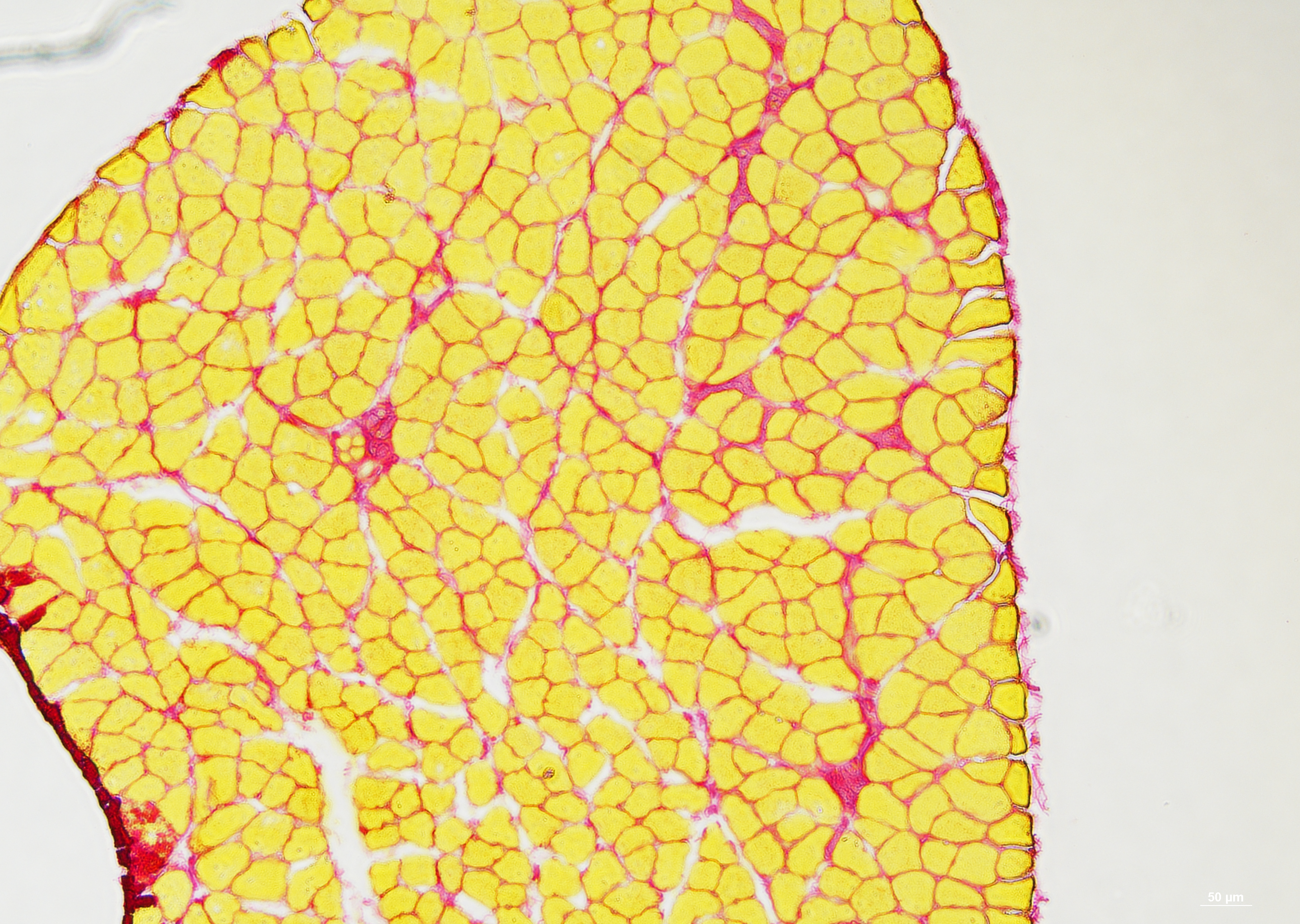

Supplement: Supplementary file 13 — Figure EV2 Source Data [file 44321_2025_337_MOESM13_ESM.zip › Figure EV2/Fig EV2F_Sirius Red staining_Representative images/Xbp1-mKO-PBS.tif]

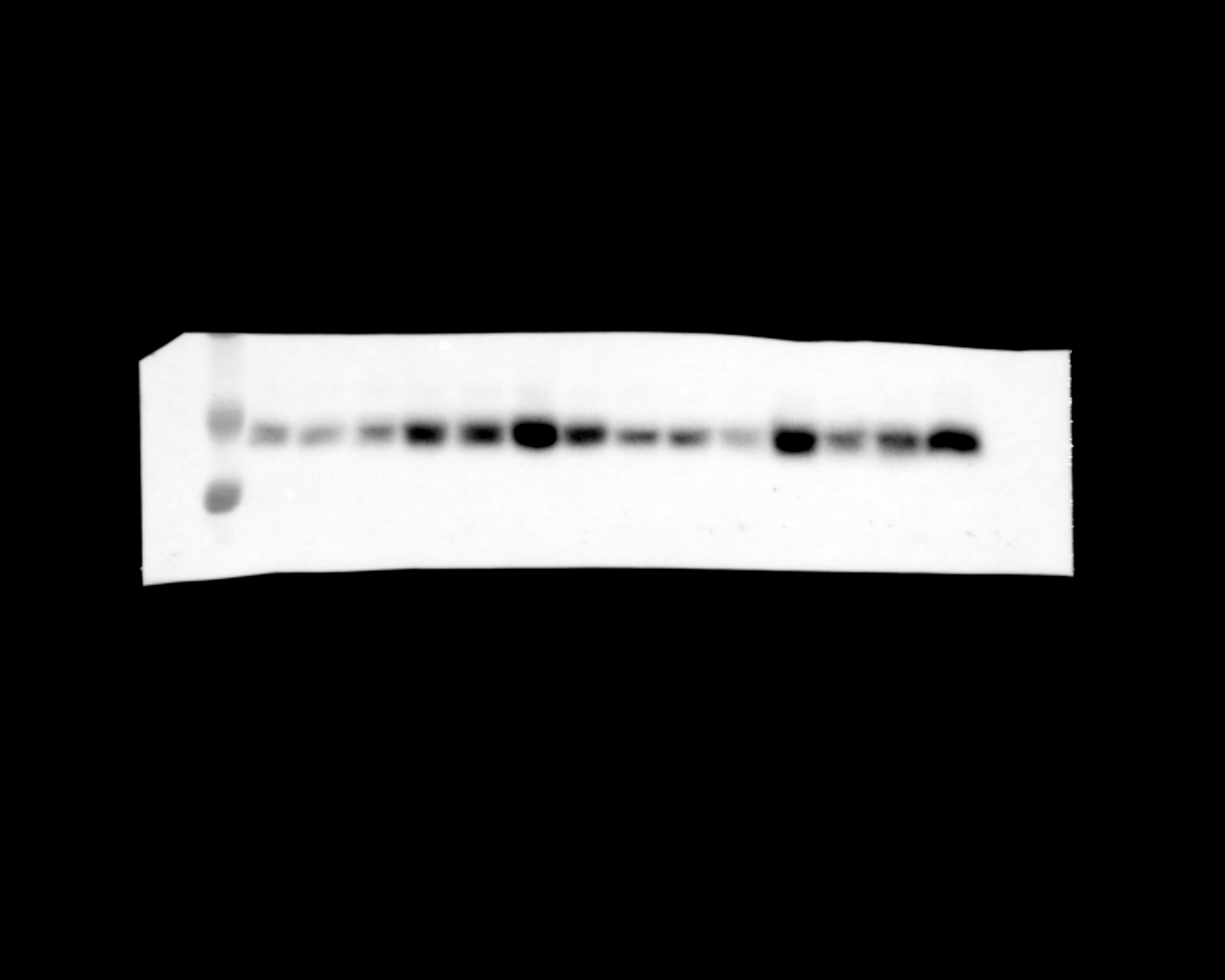

Supplement: Supplementary file 14 — Figure EV4 Source Data [file 44321_2025_337_MOESM14_ESM.zip › Figure EV4/Fig EV4A_Western blot images/CHOP.tif]

## Slide 1
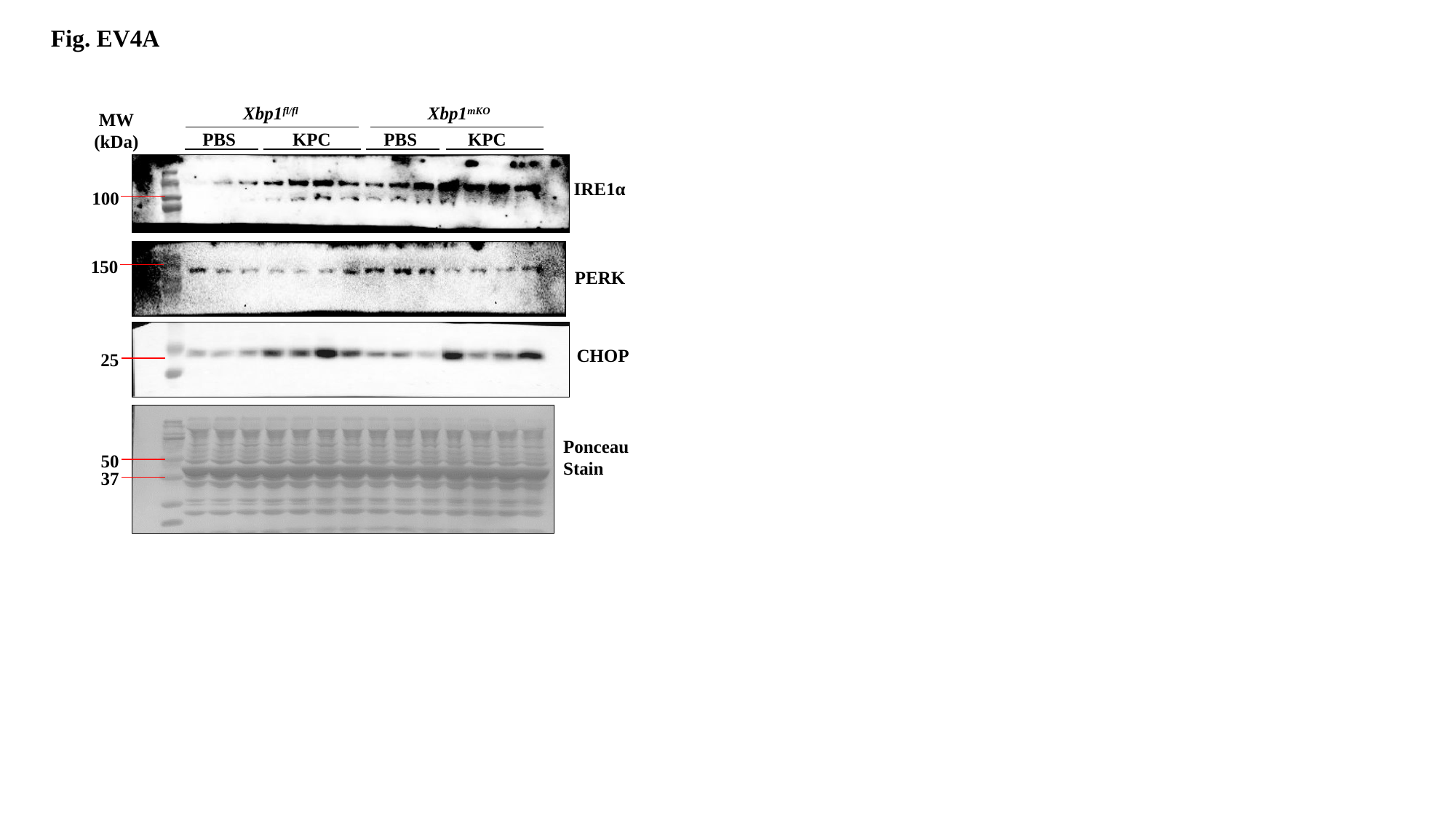

Fig. EV4A
Xbp1fl/fl
Xbp1mKO
MW (kDa)
PBS
KPC
PBS
KPC
IRE1α
100
150
PERK
CHOP
25
Ponceau Stain
50
37

Supplement: Supplementary file 14 — Figure EV4 Source Data [file 44321_2025_337_MOESM14_ESM.zip › Figure EV4/Fig EV4A_Western blot images/Fig EV4A_Western blot.pptx]

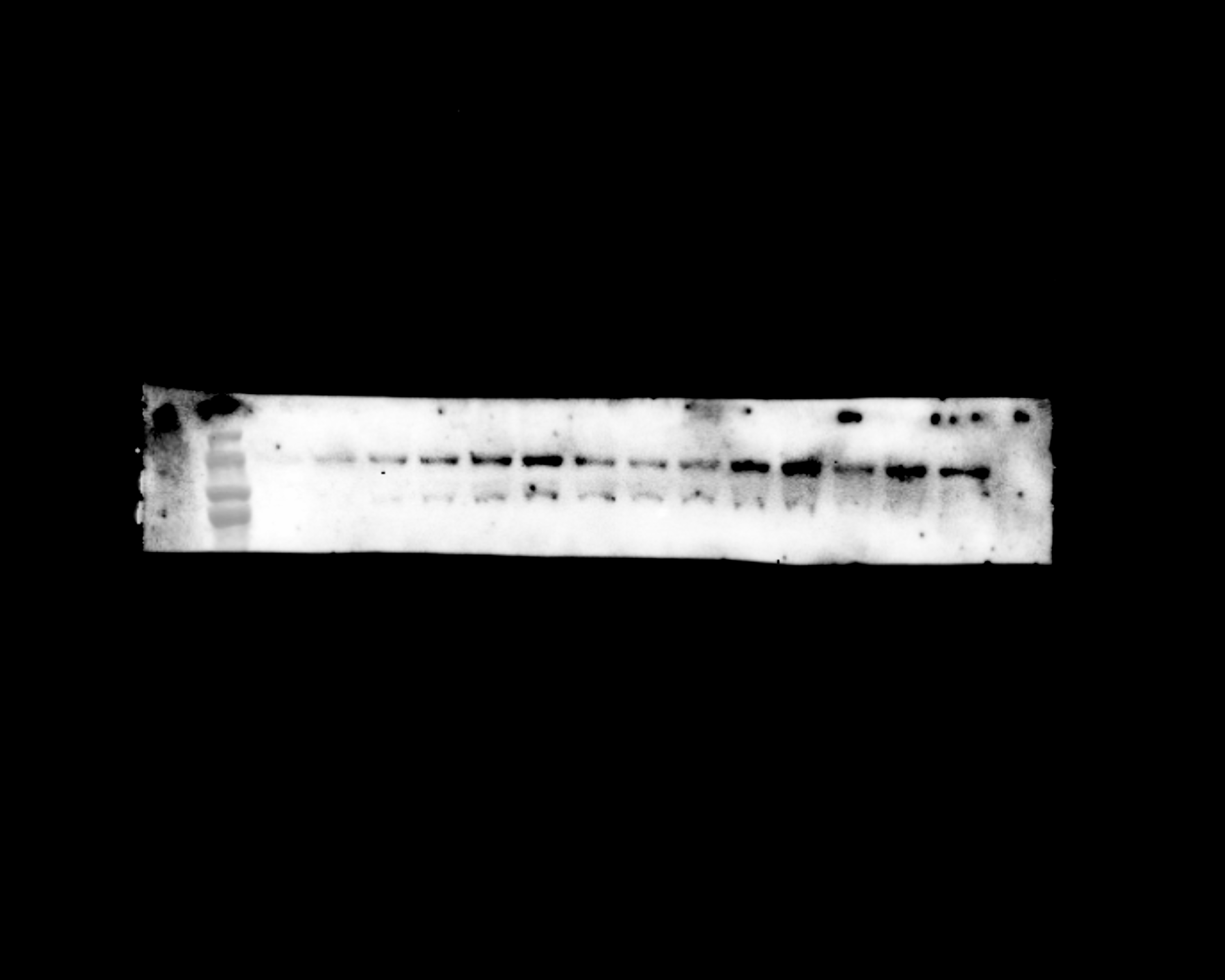

Supplement: Supplementary file 14 — Figure EV4 Source Data [file 44321_2025_337_MOESM14_ESM.zip › Figure EV4/Fig EV4A_Western blot images/IRE1a.tif]

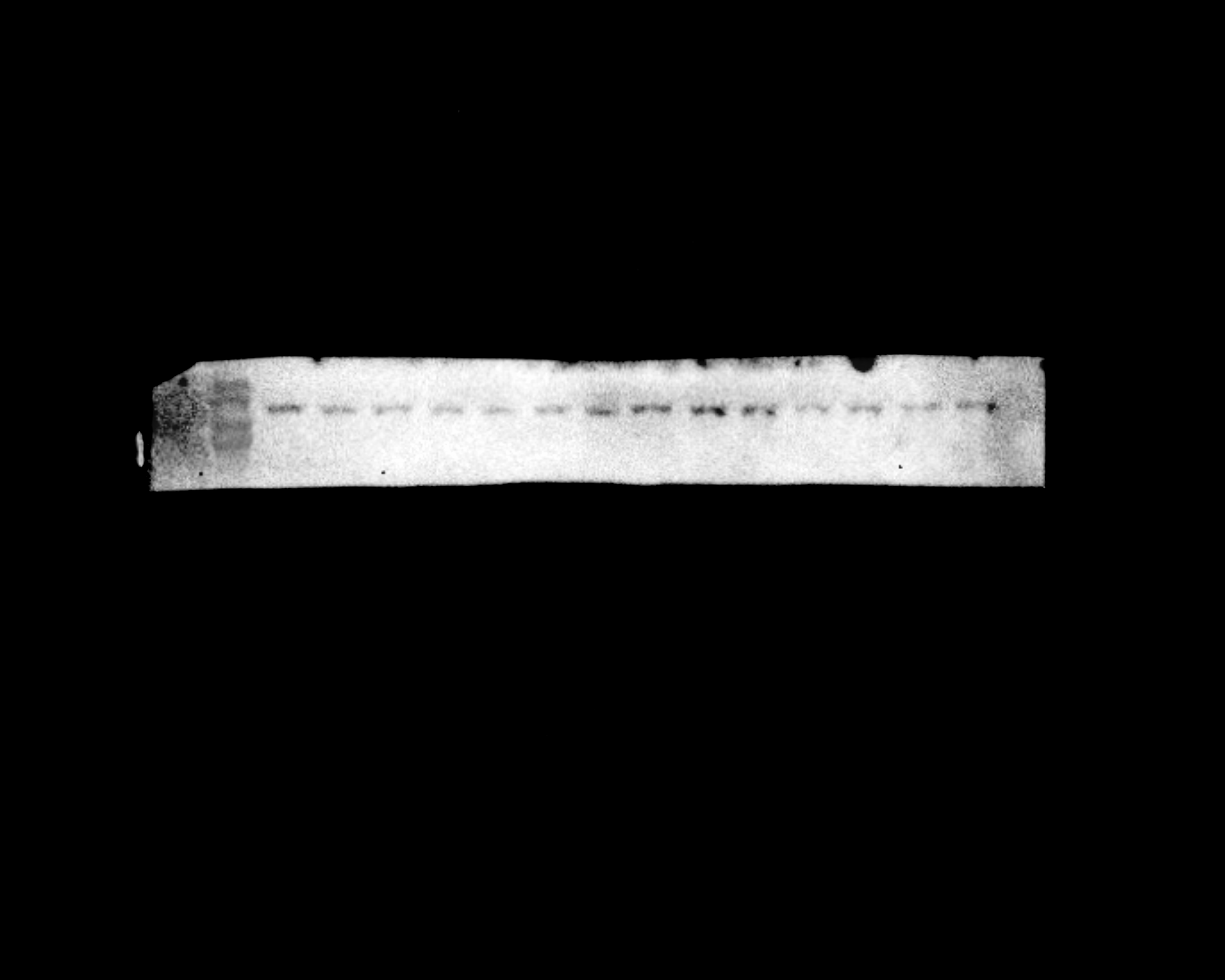

Supplement: Supplementary file 14 — Figure EV4 Source Data [file 44321_2025_337_MOESM14_ESM.zip › Figure EV4/Fig EV4A_Western blot images/PERK.tif]

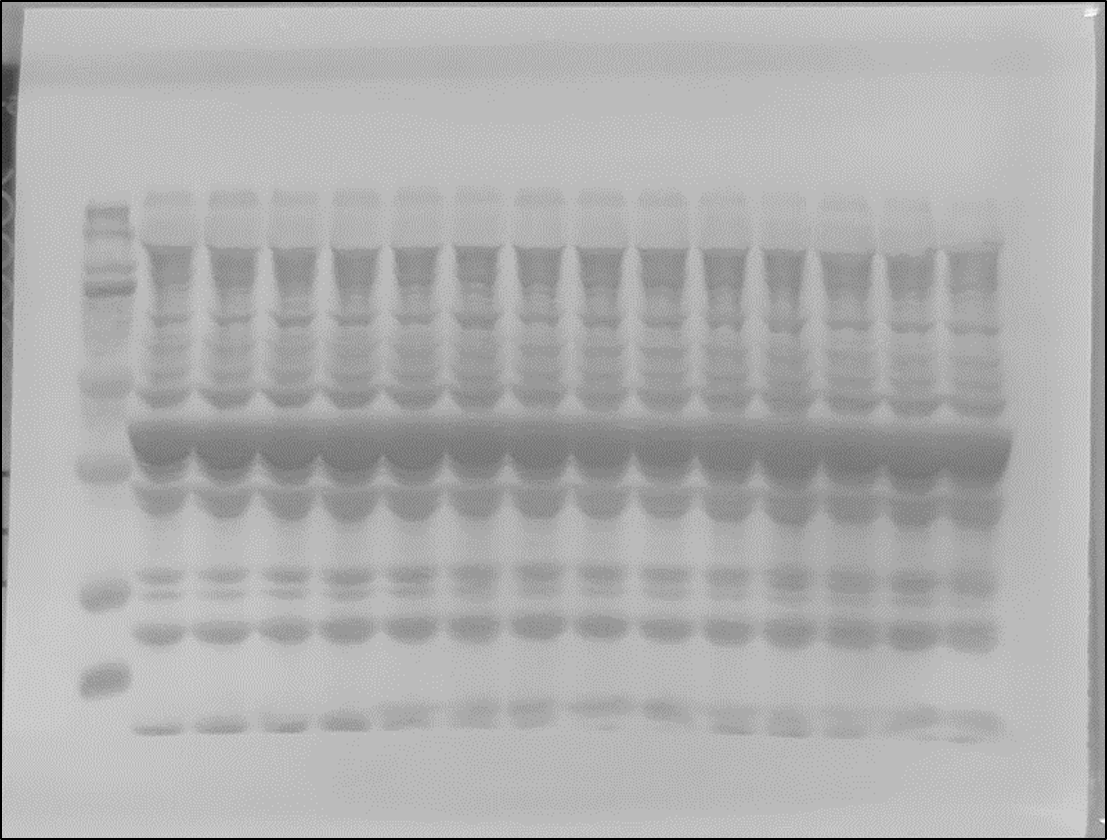

Supplement: Supplementary file 14 — Figure EV4 Source Data [file 44321_2025_337_MOESM14_ESM.zip › Figure EV4/Fig EV4A_Western blot images/Ponceau Stain.tif]

## Slide 1
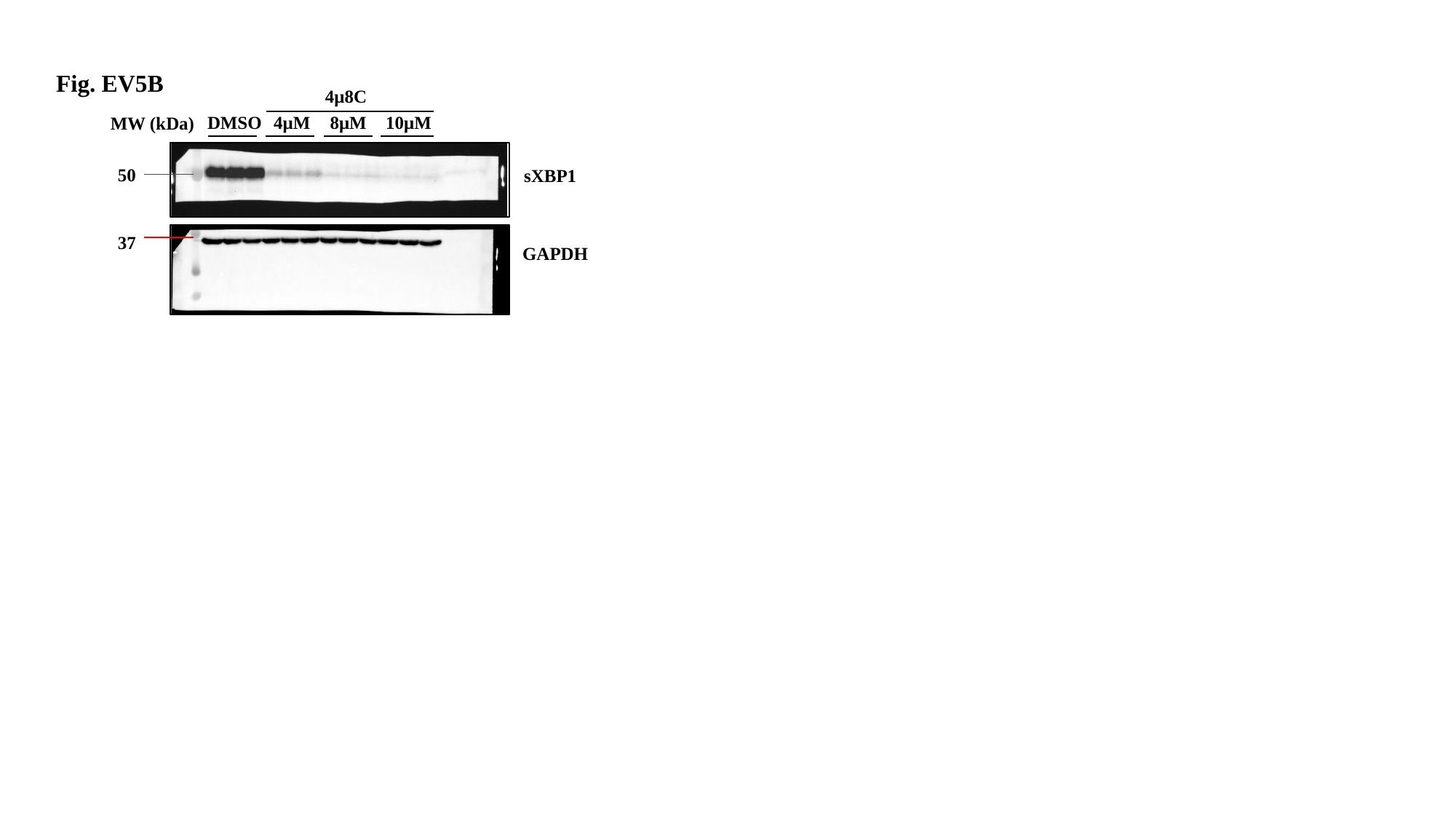

Fig. EV5B
4μ8C
DMSO
4μM
8μM
10μM
MW (kDa)
50
sXBP1
37
GAPDH

Supplement: Supplementary file 15 — Figure EV5 Source Data [file 44321_2025_337_MOESM15_ESM.zip › Figure EV5/Fig EV5B_Western blot/Fig EV5B Western blot.pptx]

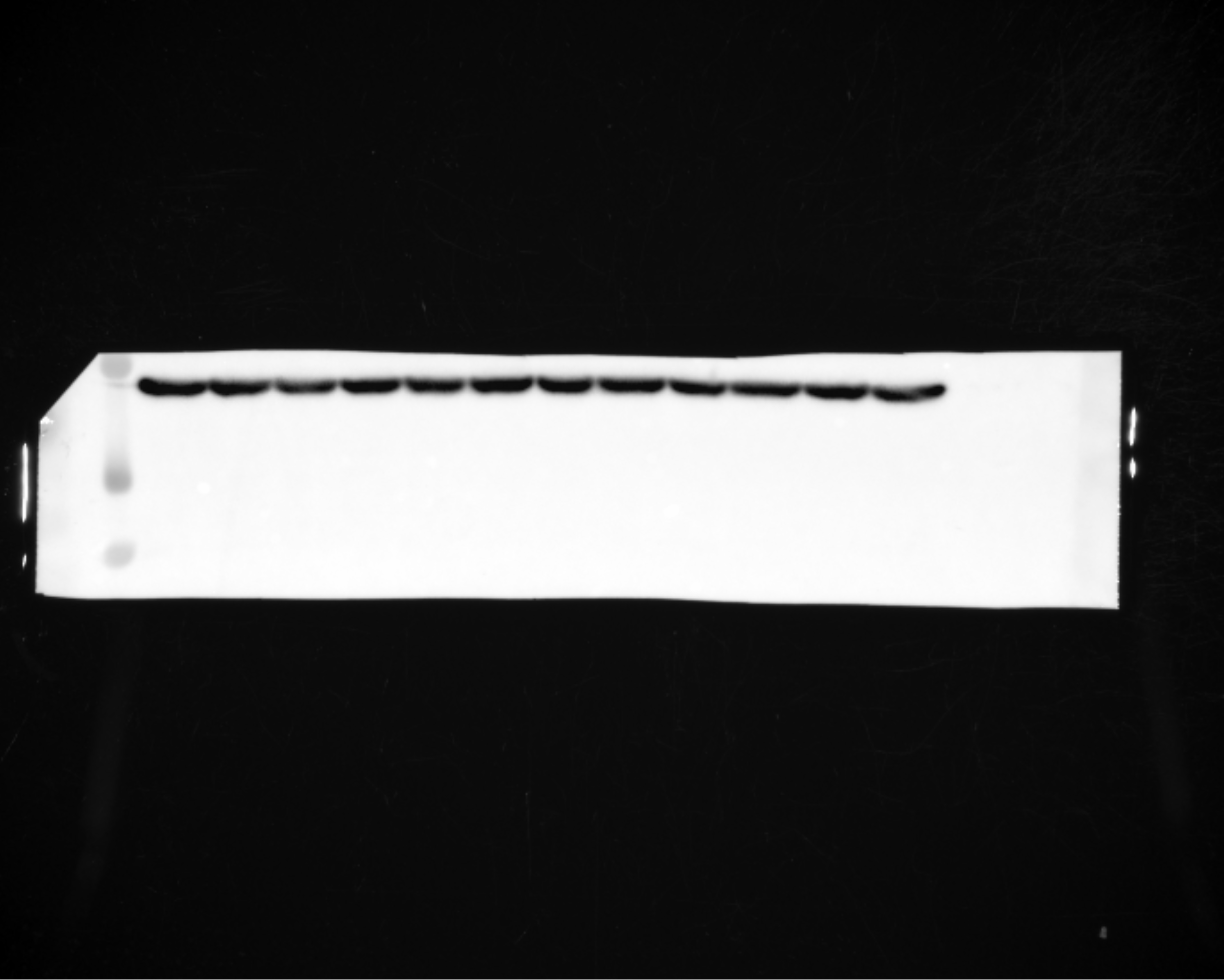

Supplement: Supplementary file 15 — Figure EV5 Source Data [file 44321_2025_337_MOESM15_ESM.zip › Figure EV5/Fig EV5B_Western blot/Western GAPDH.tif]

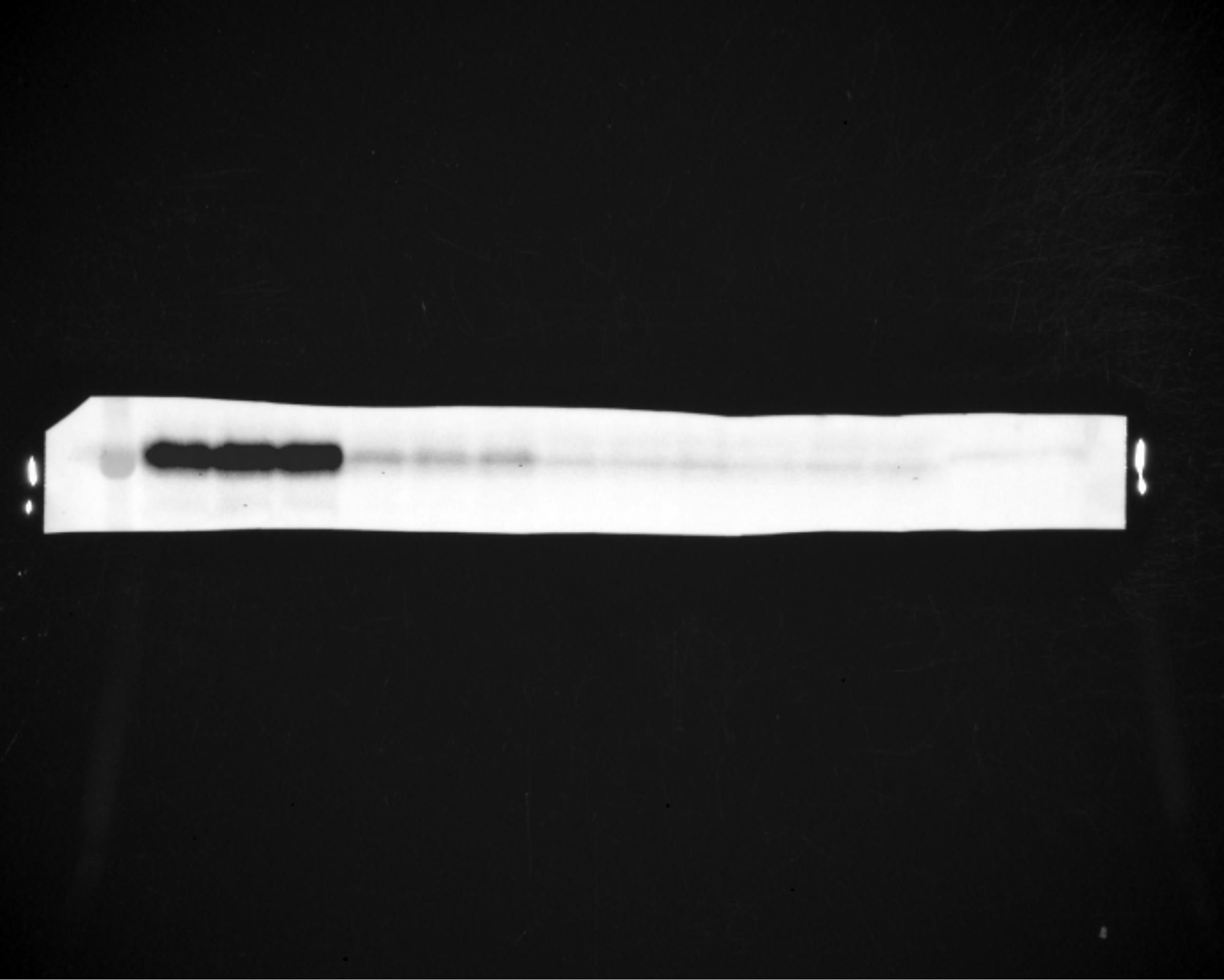

Supplement: Supplementary file 15 — Figure EV5 Source Data [file 44321_2025_337_MOESM15_ESM.zip › Figure EV5/Fig EV5B_Western blot/Western sXBP1.tif]

## Slide 1
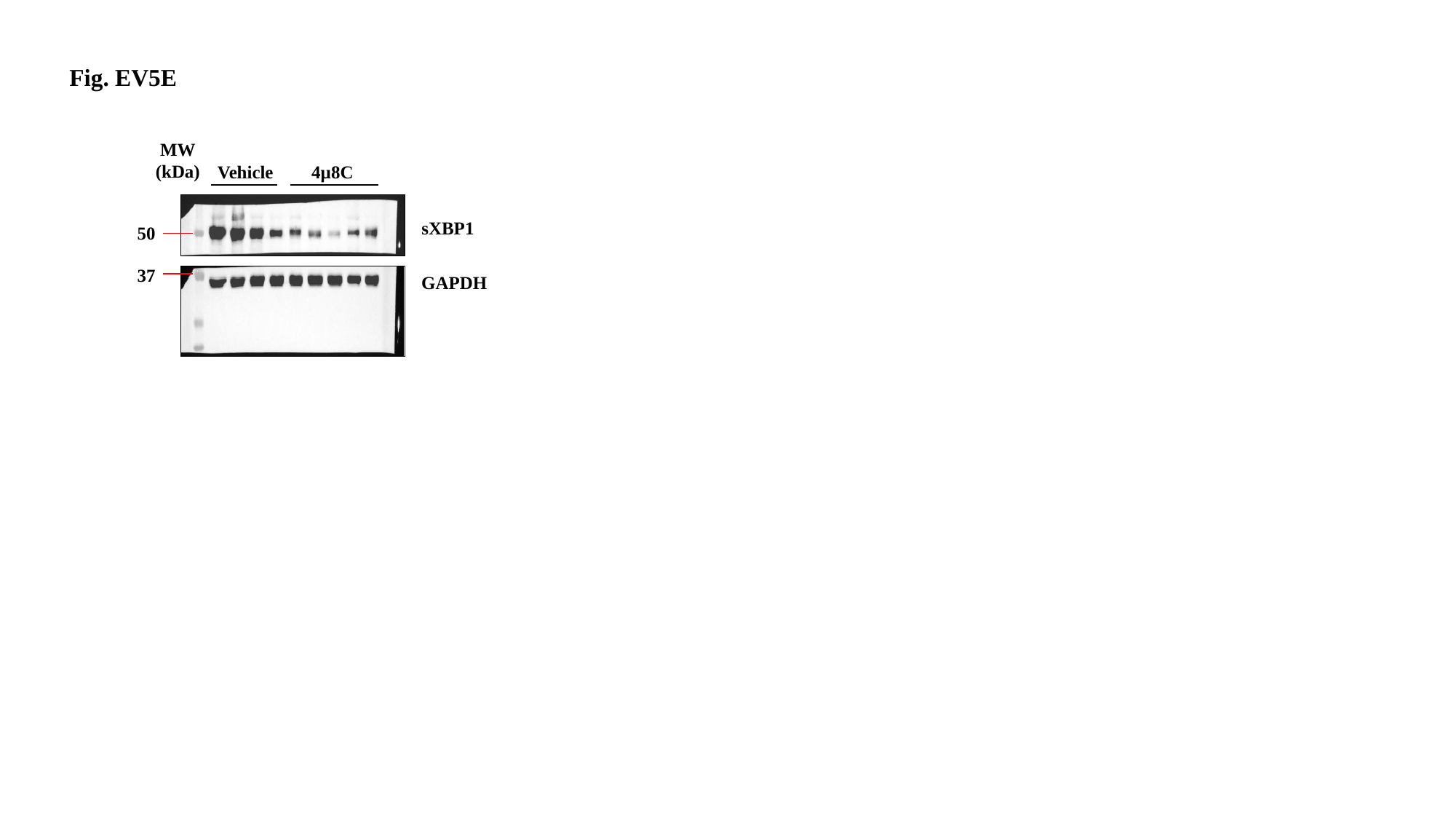

Fig. EV5E
MW (kDa)
Vehicle
4μ8C
sXBP1
50
37
GAPDH

Supplement: Supplementary file 15 — Figure EV5 Source Data [file 44321_2025_337_MOESM15_ESM.zip › Figure EV5/Fig EV5E-F_Western blot/Fig EV5E Western blot.pptx]

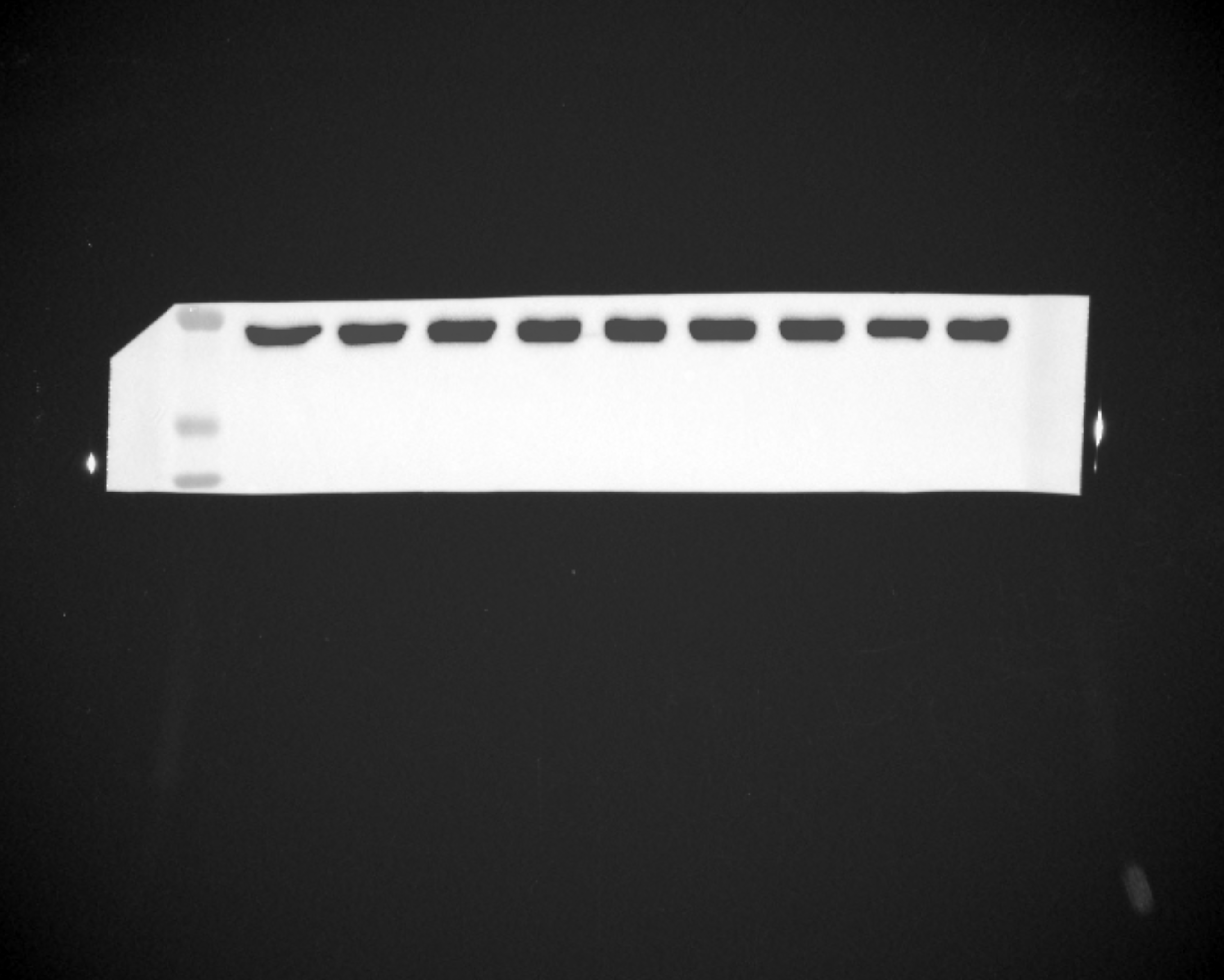

Supplement: Supplementary file 15 — Figure EV5 Source Data [file 44321_2025_337_MOESM15_ESM.zip › Figure EV5/Fig EV5E-F_Western blot/Western GAPDH.tif]

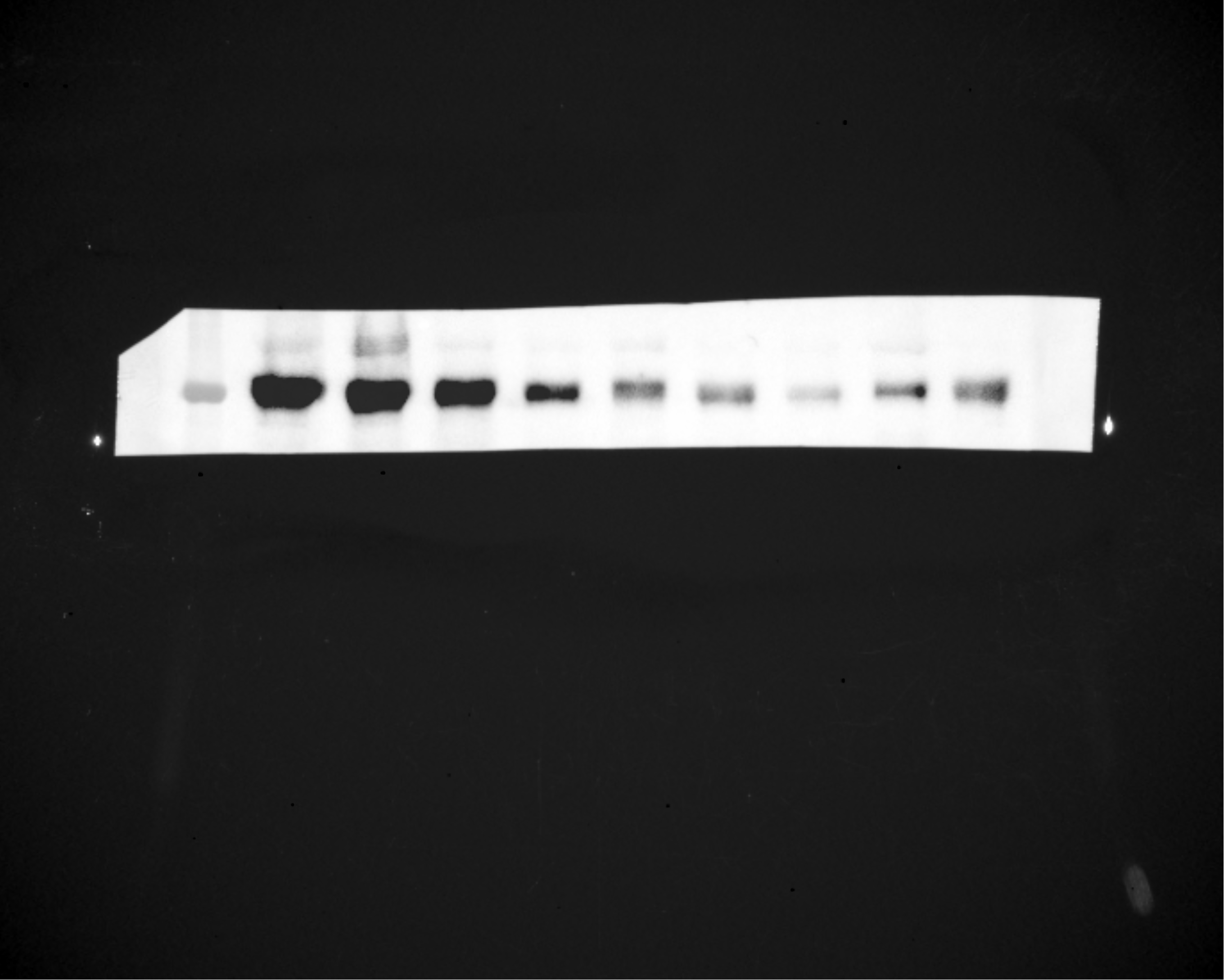

Supplement: Supplementary file 15 — Figure EV5 Source Data [file 44321_2025_337_MOESM15_ESM.zip › Figure EV5/Fig EV5E-F_Western blot/Western sXBP1.tif]

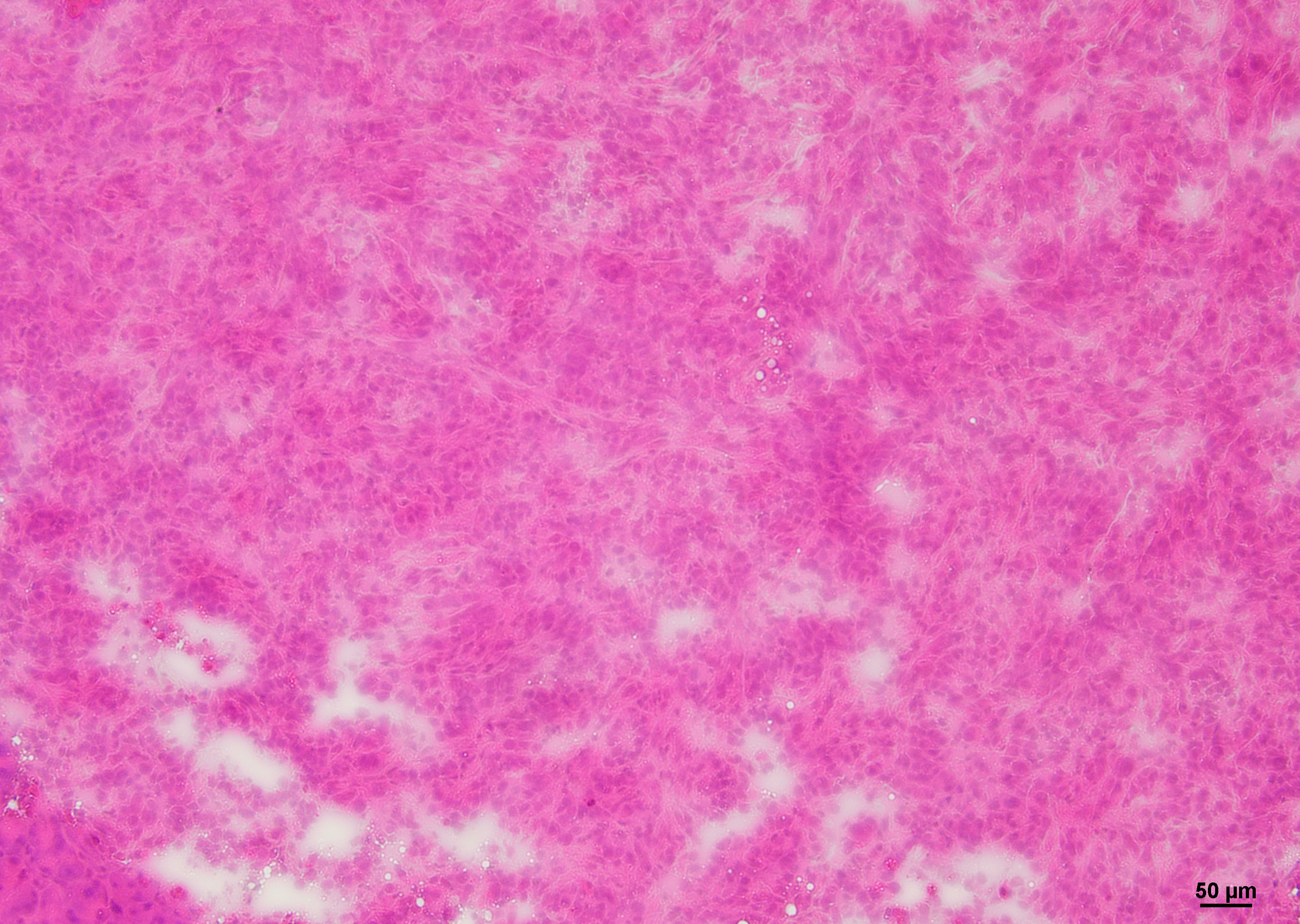

Supplement: Supplementary file 15 — Figure EV5 Source Data [file 44321_2025_337_MOESM15_ESM.zip › Figure EV5/Fig EV5G-I_Tumor histology/Fig EV5G H&E staining tumor sections_Representative images/H&E staining_4u8C KPC tumor.tif]

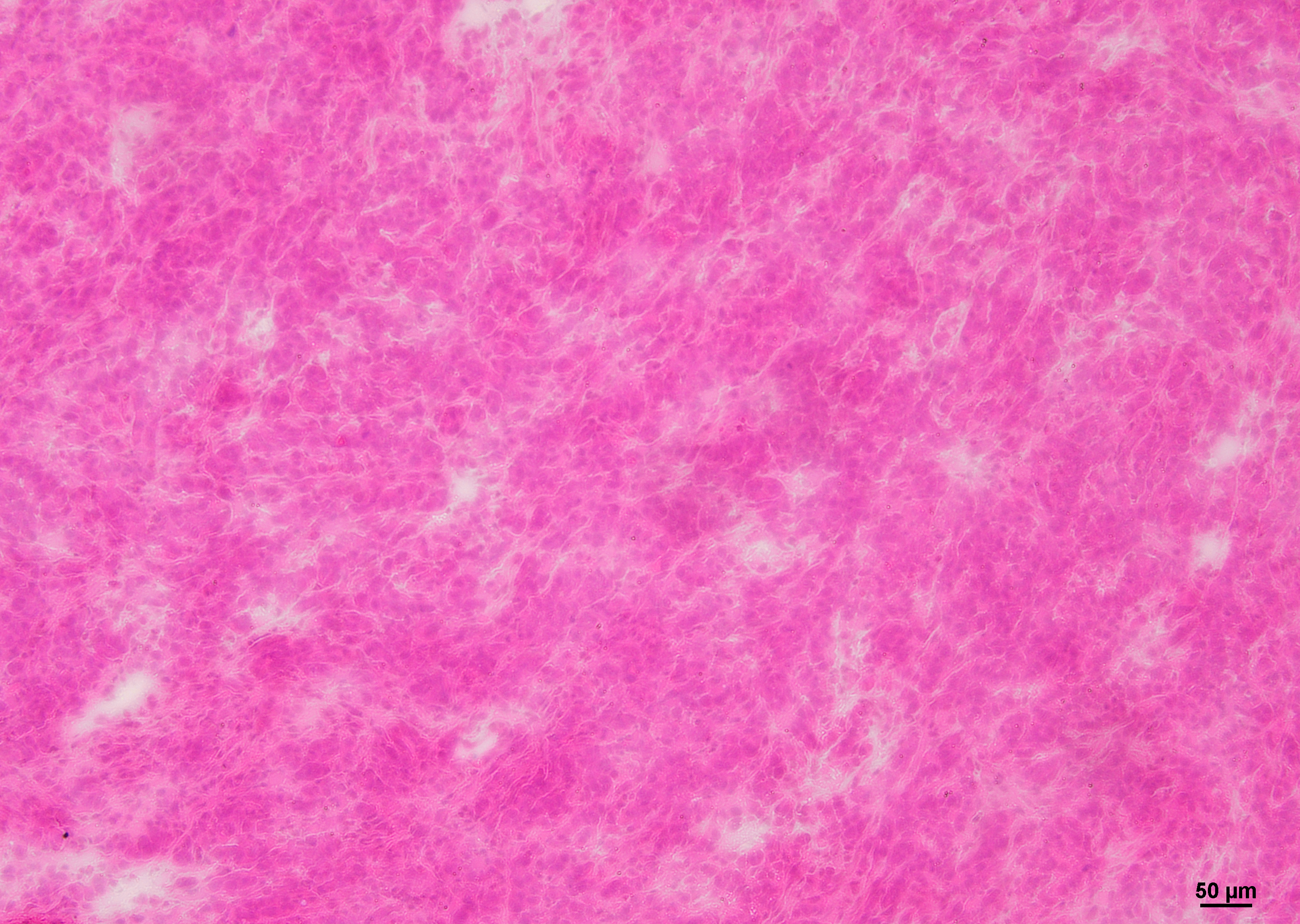

Supplement: Supplementary file 15 — Figure EV5 Source Data [file 44321_2025_337_MOESM15_ESM.zip › Figure EV5/Fig EV5G-I_Tumor histology/Fig EV5G H&E staining tumor sections_Representative images/H&E staining_Vehicle KPC tumor.tif]

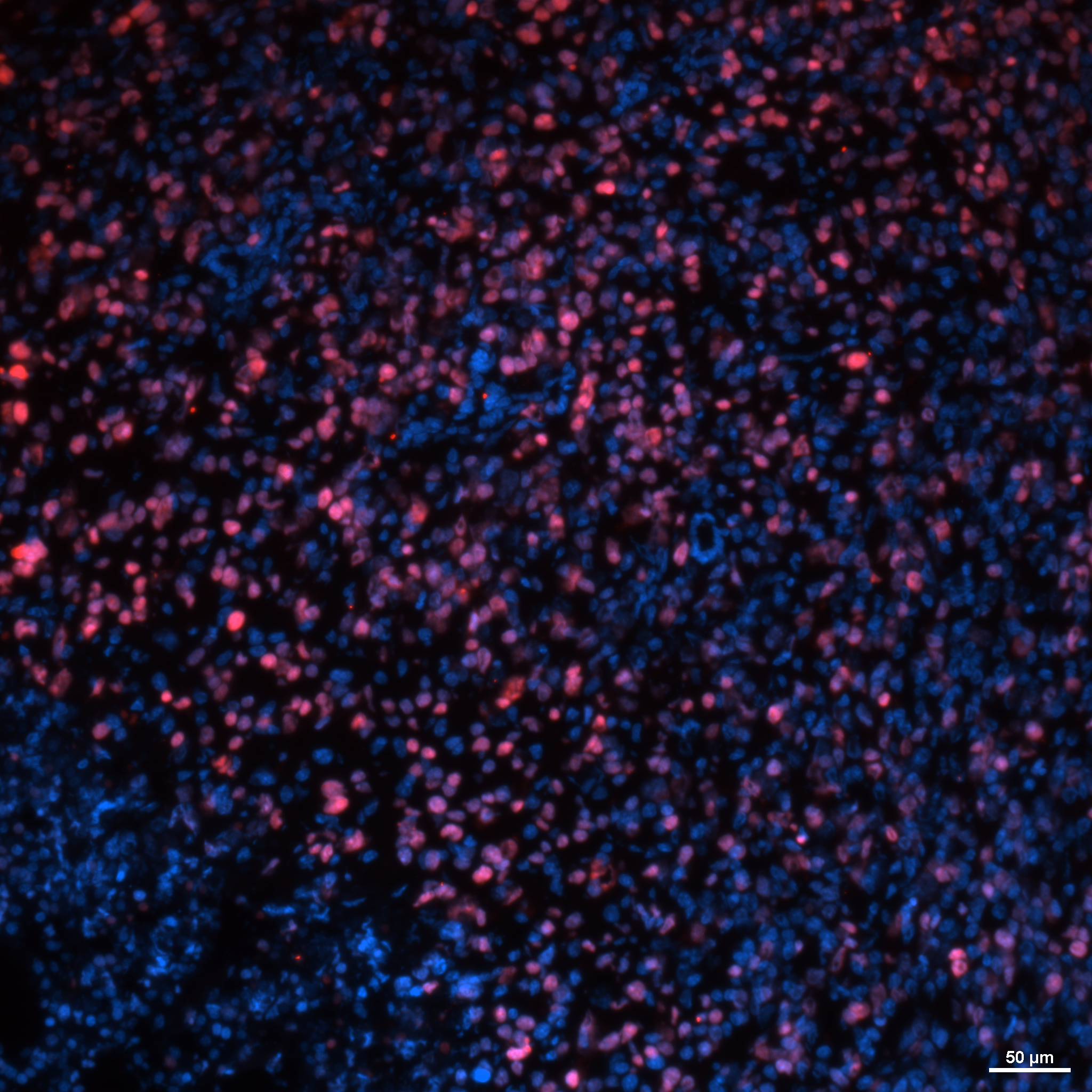

Supplement: Supplementary file 15 — Figure EV5 Source Data [file 44321_2025_337_MOESM15_ESM.zip › Figure EV5/Fig EV5G-I_Tumor histology/Fig EV5G Ki67 staining tumor sections_Representative images/Ki67 staining_4u8C KPC tumor.tif]

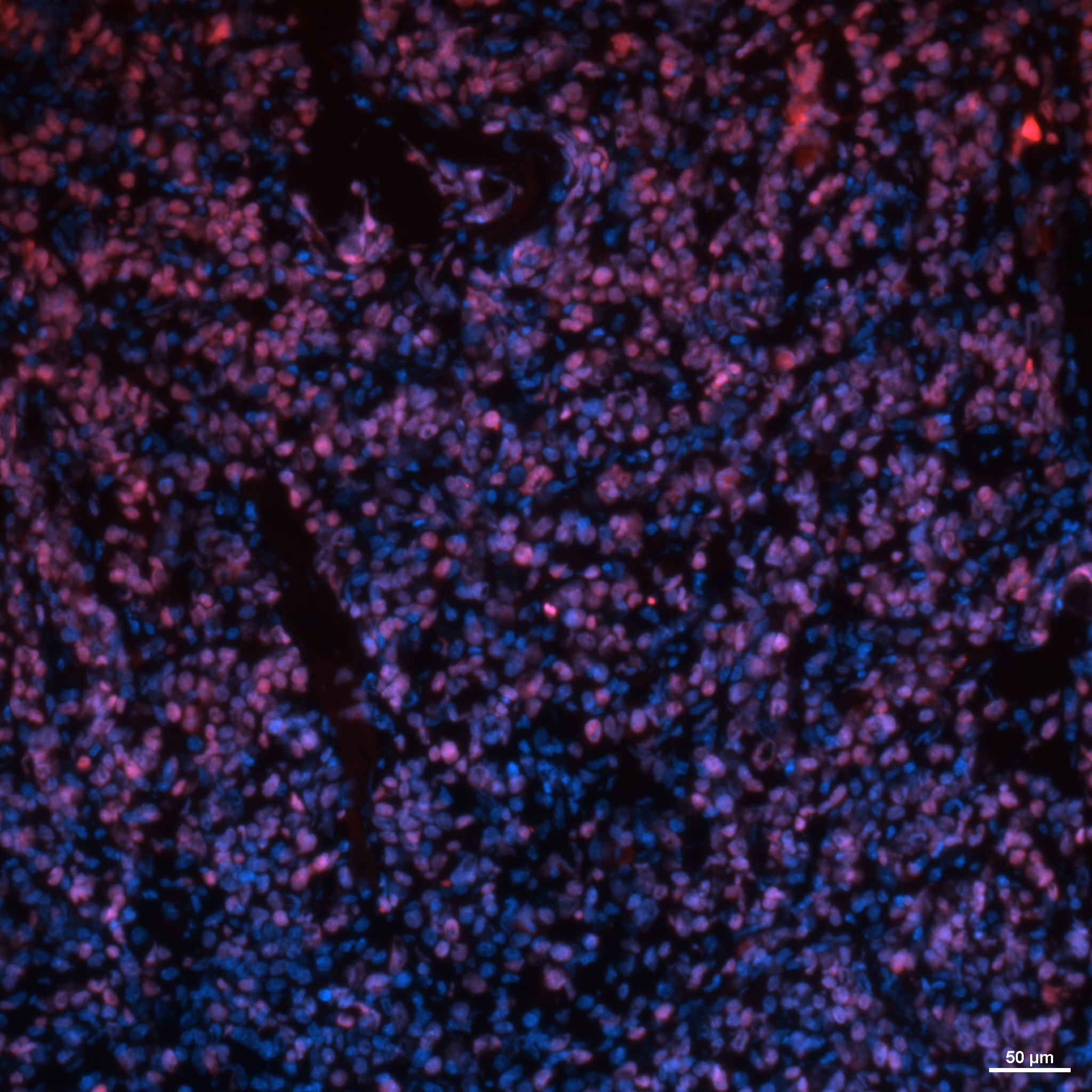

Supplement: Supplementary file 15 — Figure EV5 Source Data [file 44321_2025_337_MOESM15_ESM.zip › Figure EV5/Fig EV5G-I_Tumor histology/Fig EV5G Ki67 staining tumor sections_Representative images/Ki67 staining_Vehicle KPC tumor.tif]

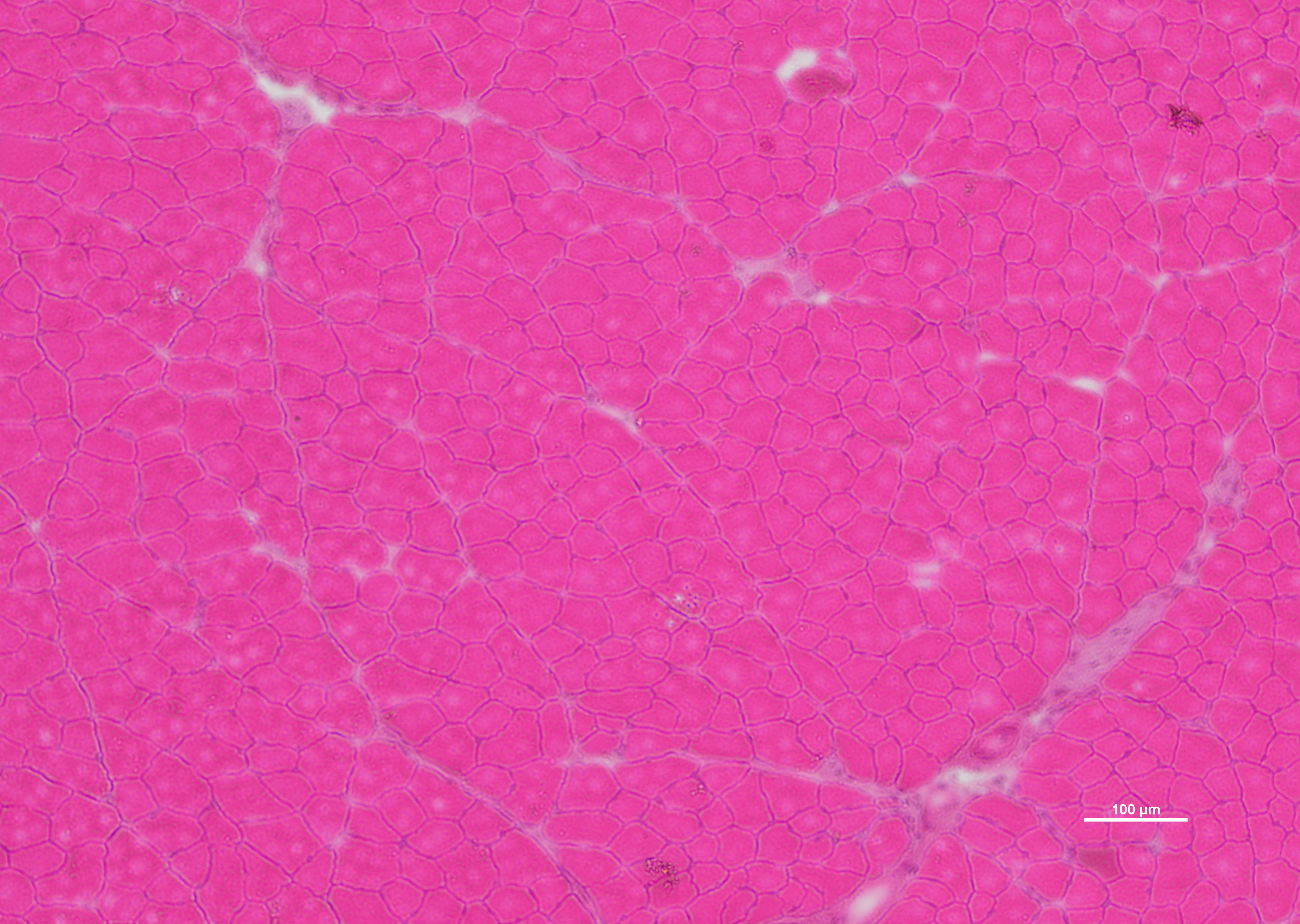

Supplement: Supplementary file 15 — Figure EV5 Source Data [file 44321_2025_337_MOESM15_ESM.zip › Figure EV5/Fig EV5J_TA muscle H&E staining/Fig EV5J H&E staining representative images/4u8C-KPC tumor-bearing.tif]

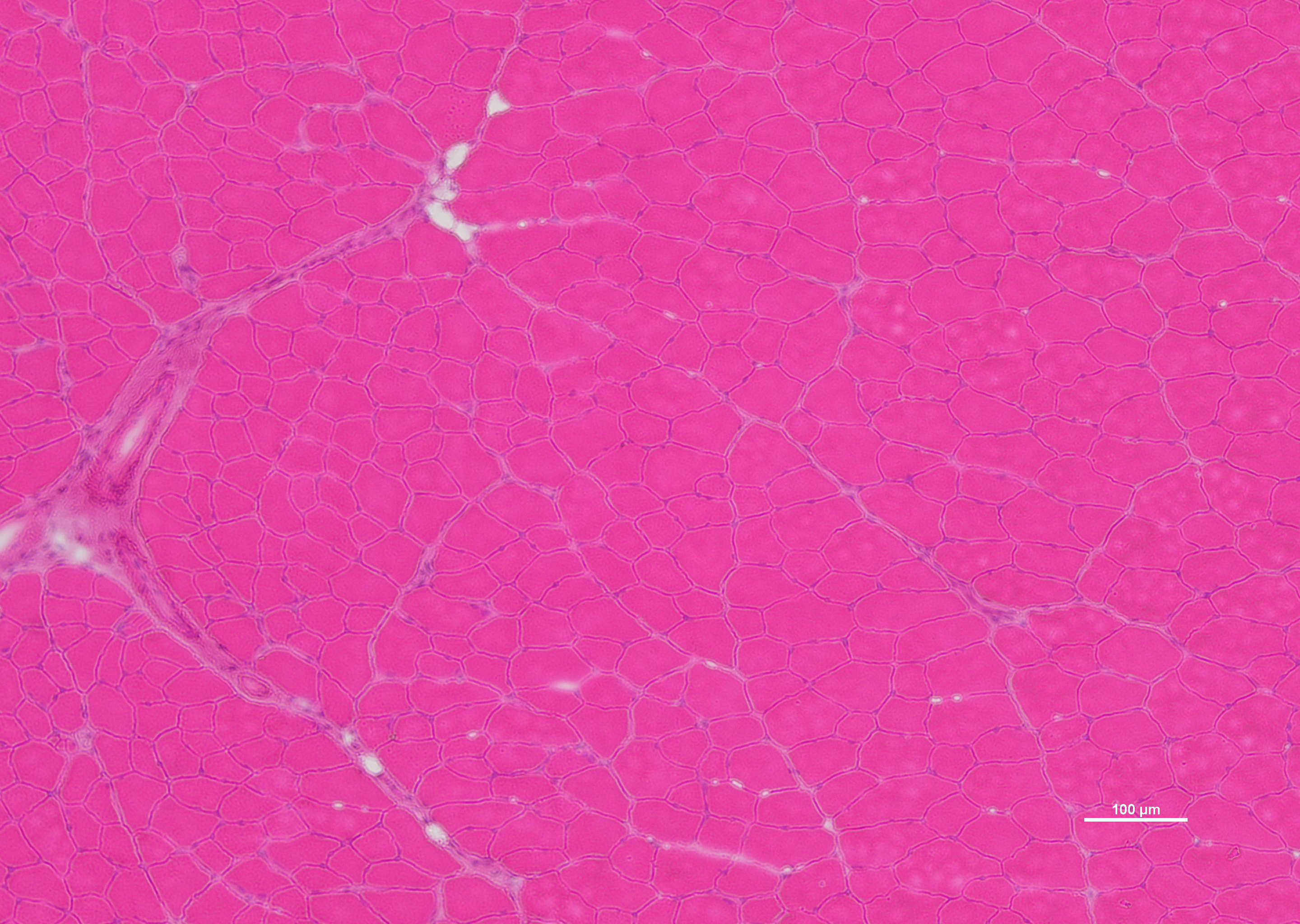

Supplement: Supplementary file 15 — Figure EV5 Source Data [file 44321_2025_337_MOESM15_ESM.zip › Figure EV5/Fig EV5J_TA muscle H&E staining/Fig EV5J H&E staining representative images/PBS-injected.tif]

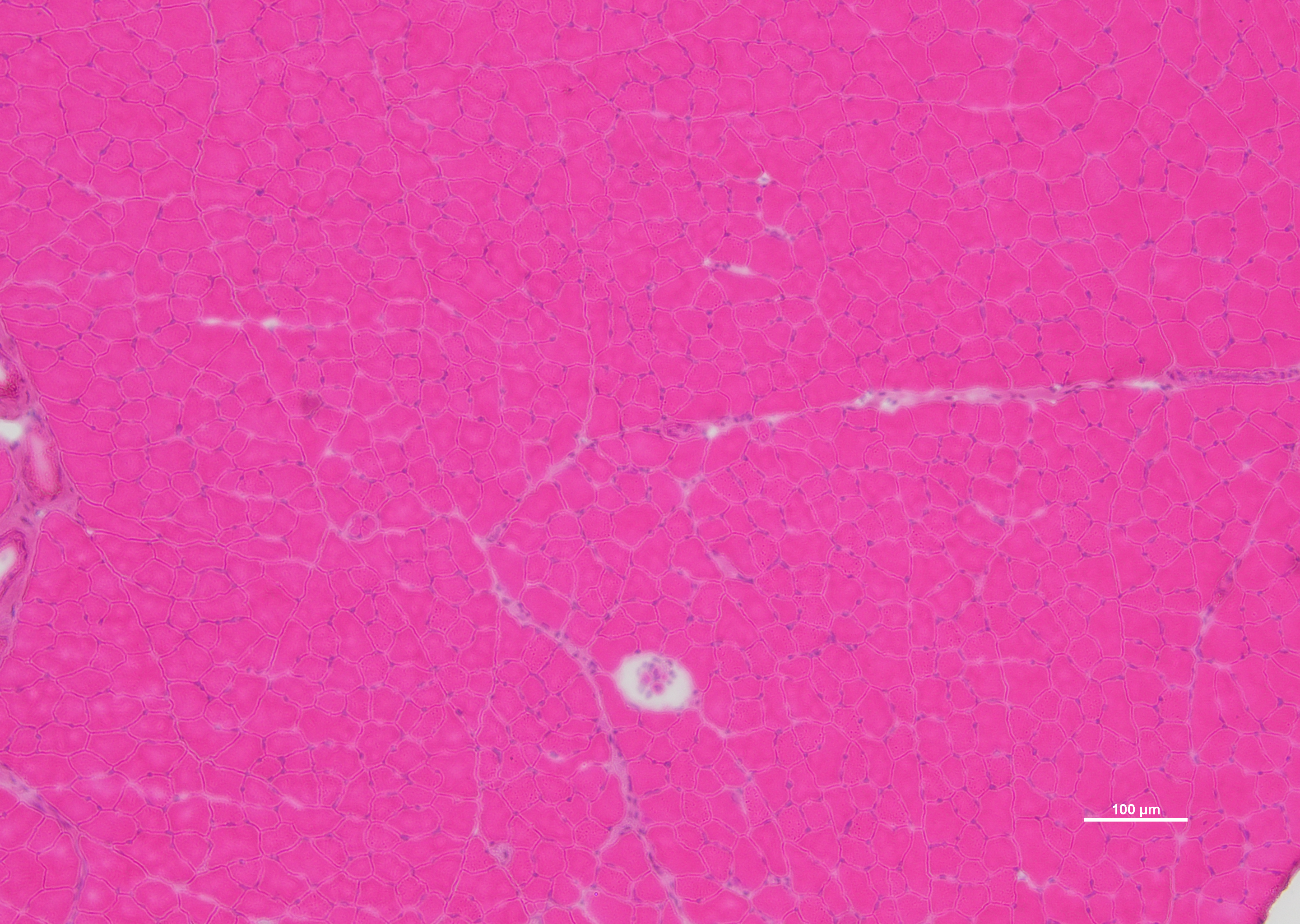

Supplement: Supplementary file 15 — Figure EV5 Source Data [file 44321_2025_337_MOESM15_ESM.zip › Figure EV5/Fig EV5J_TA muscle H&E staining/Fig EV5J H&E staining representative images/Vehicle-KPC tumor-bearing.tif]
