# Supplementary material for: The canonical ER stress IRE1α/XBP1 pathway mediates skeletal muscle wasting during pancreatic cancer cachexia
Source: EMBO Mol Med. 2025 Nov 17;17(12):3607–35. doi: 10.1038/s44321-025-00337-w (PMC12686462; doi:10.1038/s44321-025-00337-w)
Supplement: Supplementary file 16 — Expanded View Figures [file 44321_2025_337_MOESM16_ESM.pdf]

Expanded View Figures

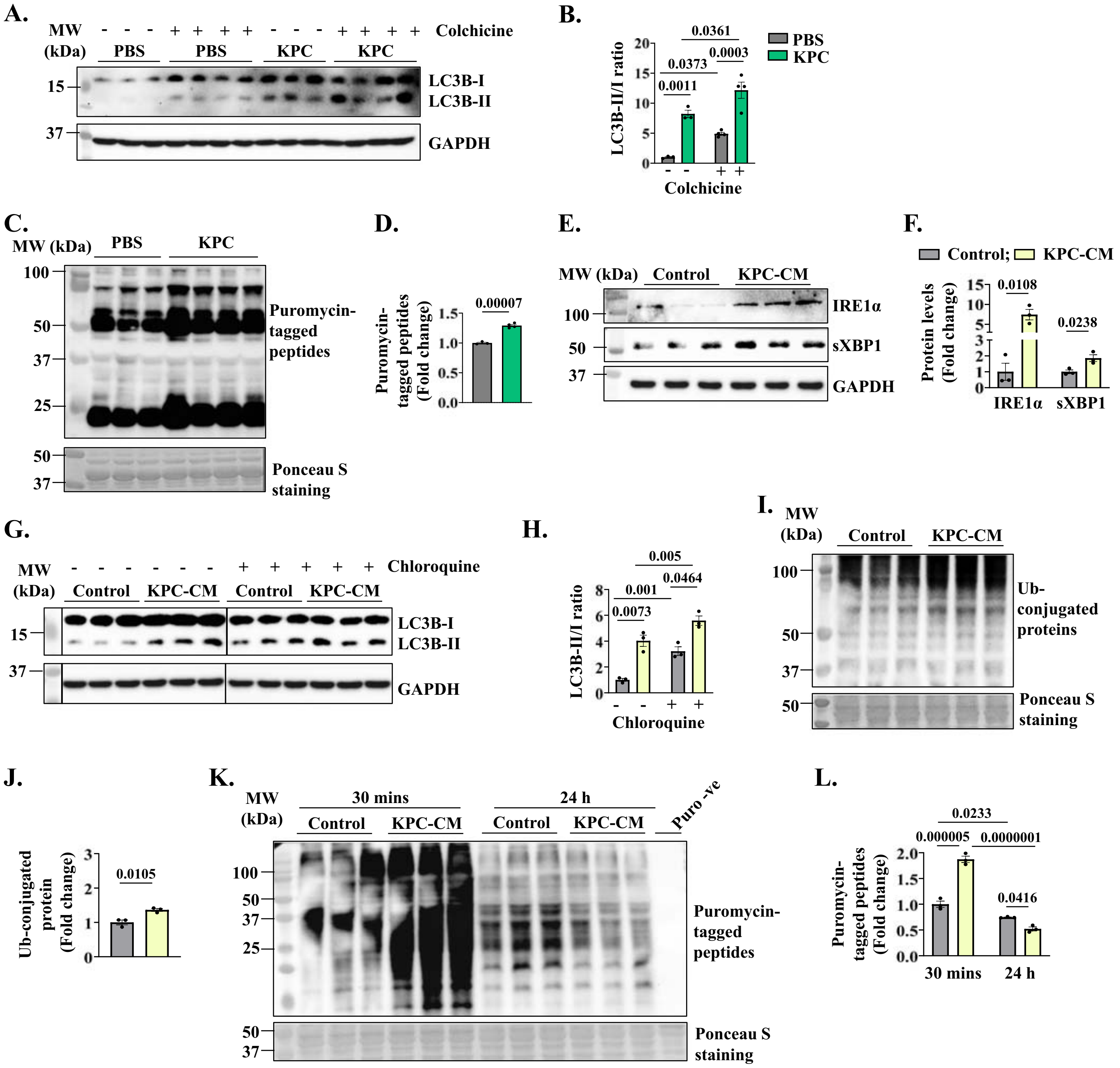

Figure EV1. KPC tumor-derived factors activate IRE1α/XBP1 signaling and regulate protein turnover in cultured myotubes.

(**A**) Immunoblots and (**B**) densitometry analysis of levels of LC3B protein in control (PBS) and KPC tumor-bearing mice with or without treatment with (0.4 mg/kg/day) colchicine. $n = 3$–4 mice in each group. Data information: Data are presented as mean ± SEM. Indicated *P* values were calculated using two-way ANOVA followed by Tukey's multiple comparison test. (**C**) Immunoblots and (**D**) densitometry analysis of amounts of puromycin-tagged peptides in control (PBS) and KPC tumor-bearing mice. $n = 3$–4 mice in each group. Data information: Data are presented as mean ± SEM. Indicated *P* values were calculated using unpaired Student *t* test. (**E**) Immunoblots and (**F**) densitometry analysis of levels of IRE1α and sXBP1 protein in control and KPC-CM treated myotube cultures. (**G**) Immunoblot and (**H**) quantification of ratio of LC3B-II/I protein in myotube cultures incubated in DM (control) or KPC-CM and treated with vehicle alone or (100 μM) chloroquine. $n = 3$ biological replicates per group. Data information: Data are presented as mean ± SEM. Indicated *P* values were calculated using two-way ANOVA followed by Tukey's multiple comparison test. (**I**) Immunoblots and (**J**) densitometry analysis of levels of ubiquitin (Ub)-conjugated proteins in control and KPC-CM treated cultures. $n = 3$ biological replicates per group. Data information: Data are presented as mean ± SEM. Indicated *P* values were calculated using unpaired Student *t* test. (**K**) Immunoblots and (**L**) densitometry analysis of amounts of puromycin-tagged peptides in myotube cultures after 30 mins or 24 h of incubation in DM (control) or KPC-CM. $n = 3$ biological replicates in each group. Data information: Data are presented as mean ± SEM. Indicated *P* values were calculated using two-way ANOVA followed by Tukey's multiple comparison test. Source data are available online for this figure.

© The Author(s)

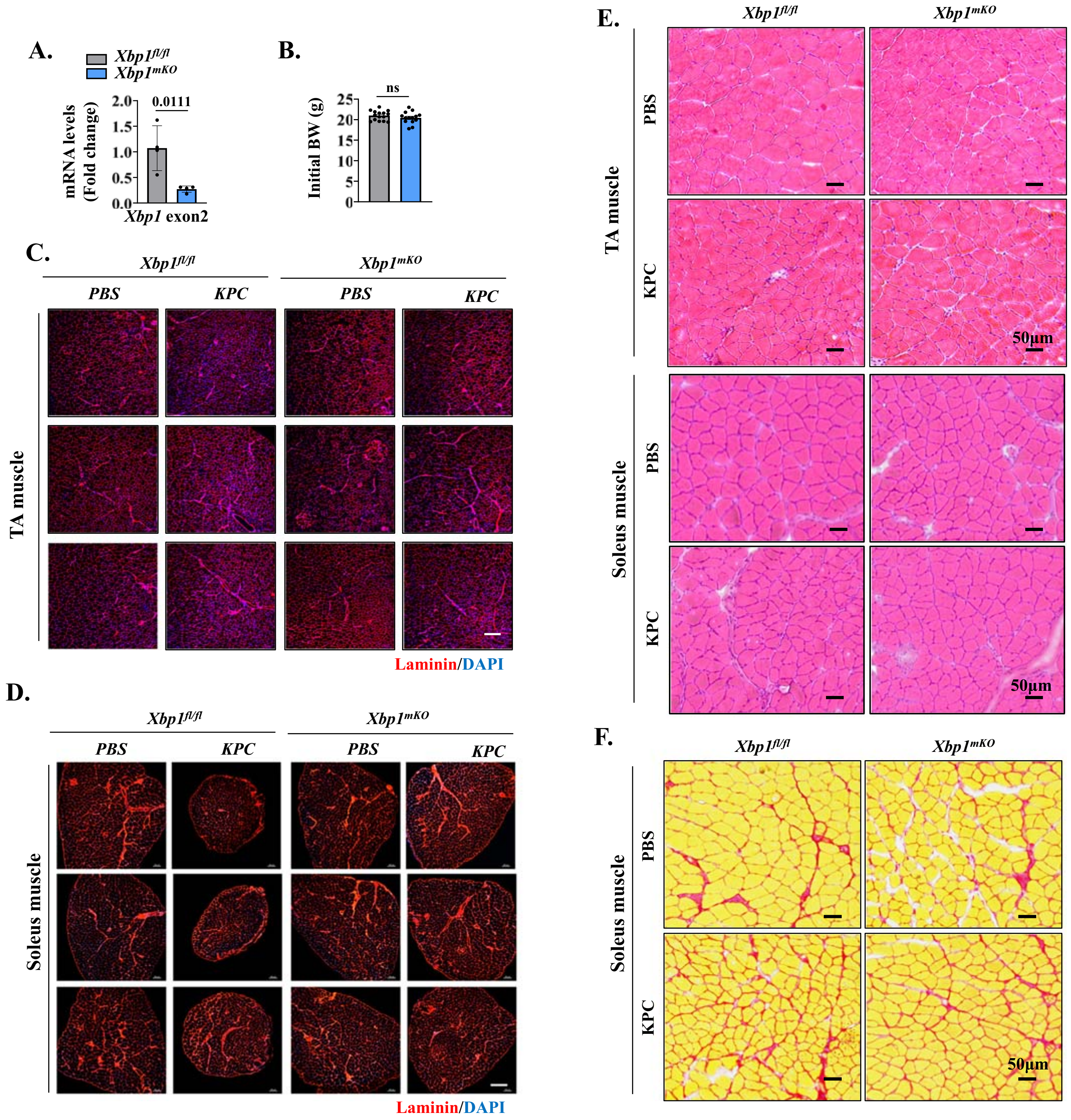

Figure EV2. Targeted deletion of XBP1 inhibits muscle atrophy in KPC tumor-bearing mice.

(**A**) Relative mRNA levels of XBP1 determined by qRT-PCR analysis using primer set specific for Xbp1 exon 2, a sequence flanked by the loxP sites, in gastrocnemius (GA) muscle of *Xbp1*$^{fl/fl}$ and *Xbp1*^{mKO} mice. $n = 4$ mice per group. Data information: Data are presented as mean ± SEM. Indicated *P* values were calculated using unpaired Student *t* test. (**B**) Quantification of initial body weight of *Xbp1*$^{fl/fl}$ and *Xbp1*^{mKO} mice. $n = 14$–15 mice per group. Data information: No significant difference was observed using unpaired Student *t* test. Transverse sections of TA and soleus muscle isolated from PBS- or KPC cells-injected *Xbp1*$^{fl/fl}$ and *Xbp1*^{mKO} mice were used for anti-laminin and DAPI staining or H&E staining. (**C, D**) Anti-laminin and DAPI stained sections of (**C**) TA and (**D**) soleus muscle from multiple mice. Scale bar, 200µm. (**E**) Representative photomicrographs of H&E-stained transverse sections of TA (upper panel) and soleus (lower panel) muscle of control and KPC tumor-bearing *Xbp1*$^{fl/fl}$ and *Xbp1*^{mKO} mice. Scale bar, 50µm. (**F**) Representative photomicrographs of Sirius red-stained soleus muscle sections of *Xbp1*$^{fl/fl}$ and *Xbp1*^{mKO} mice injected with PBS or KPC cells. Scale bar, 50 µm. Source data are available online for this figure.

© The Author(s)

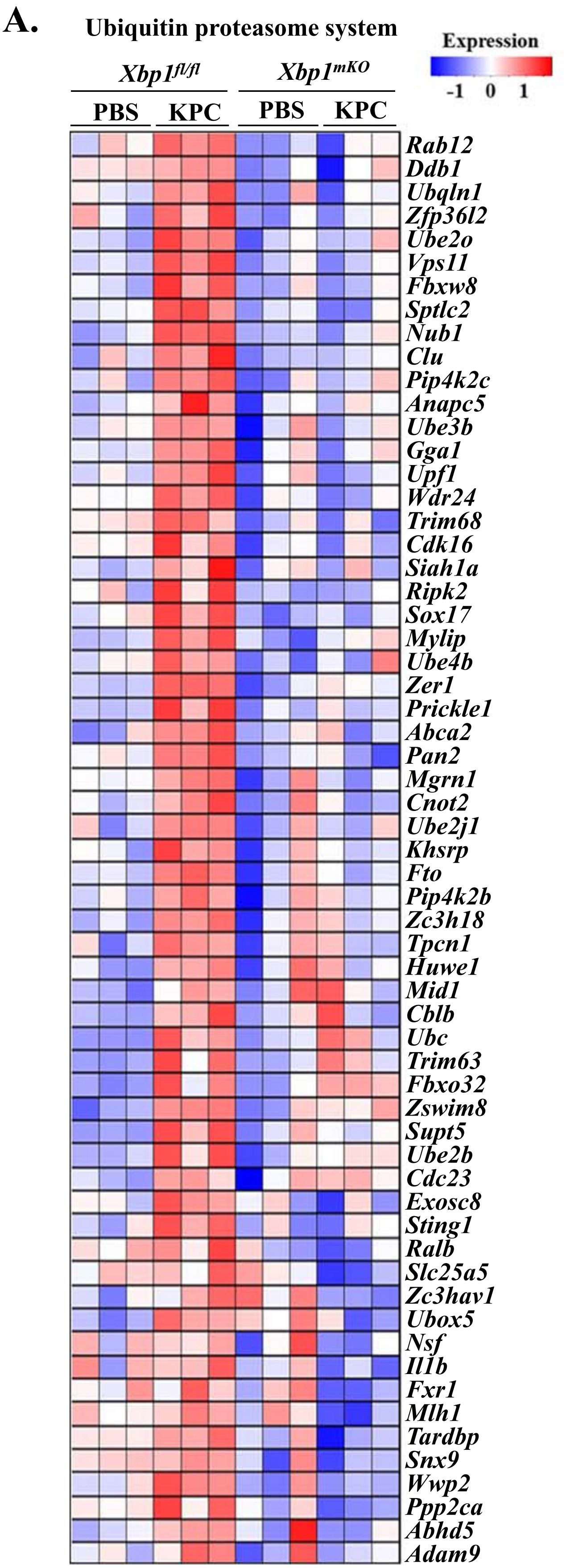
A.
Ubiquitin proteasome system
Expression
-1 0 1
Xbp1fl/fl
Xbp1mKO
PBS
KPC
PBS
KPC
Rab12
Ddb1
Ubqln1
Zfp36l2
Ube2o
Vps11
Fbxw8
Sptlc2
Nub1
Clu
Pip4k2c
Anapc5
Ube3b
Gga1
Upf1
Wdr24
Trim68
Cdk16
Siah1a
Ripk2
Sox17
Mylip
Ube4b
Zer1
Prickle1
Abca2
Pan2
Mgrn1
Cnot2
Ube2j1
Khsrp
Fto
Pip4k2b
Zc3h18
Tpcn1
Huwe1
Mid1
Cblb
Ubc
Trim63
Fbxo32
Zswim8
Supt5
Ube2b
Cdc23
Exosc8
Sting1
Ralb
Slc25a5
Zc3hav1
Ubox5
Nsf
Il1b
Fxr1
Mlh1
Tardbp
Snx9
Wwp2
Ppp2ca
Abhd5
Adam9

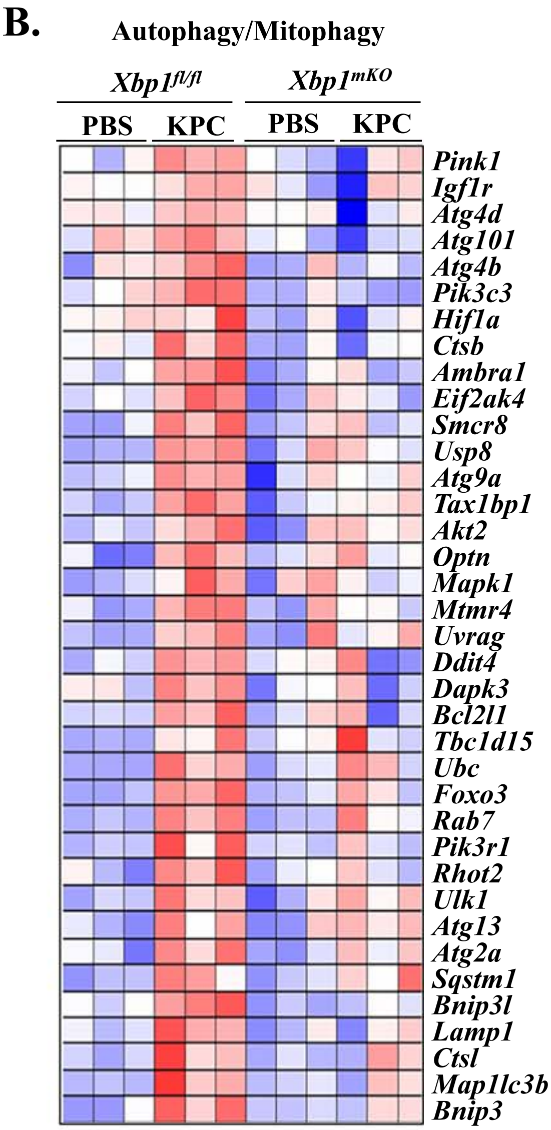
B.
Autophagy/Mitophagy
Xbp1fl/fl
Xbp1mKO
PBS
KPC
PBS
KPC
Pink1
Igf1r
Atg4d
Atg101
Atg4b
Pik3c3
Hif1a
Ctsb
Ambra1
Eif2ak4
Smcr8
Usp8
Atg9a
Tax1bp1
Akt2
Optn
Mapk1
Mtmr4
Uvrag
Ddit4
Dapk3
Bcl2l1
Tbc1d15
Ubc
Foxo3
Rab7
Pik3r1
Rhot2
Ulk1
Atg13
Atg2a
Sqstm1
Bnip3l
Lamp1
Ctsl
Map1lc3b
Bnip3

 © The Author(s)

Figure EV3. Targeted ablation of XBP1 inhibits the expression of multiple genes involved in proteolysis.

(**A**, **B**) Heatmap representation of RNA-Seq dataset analysis showing relative expression of genes involved in (**A**) Ubiquitin proteasome system (UPS), and (**B**) autophagy/mitophagy in gastrocnemius muscle of control and KPC tumor-bearing *Xbp1*$^{fl/fl}$ and *Xbp1*^{mKO} mice.

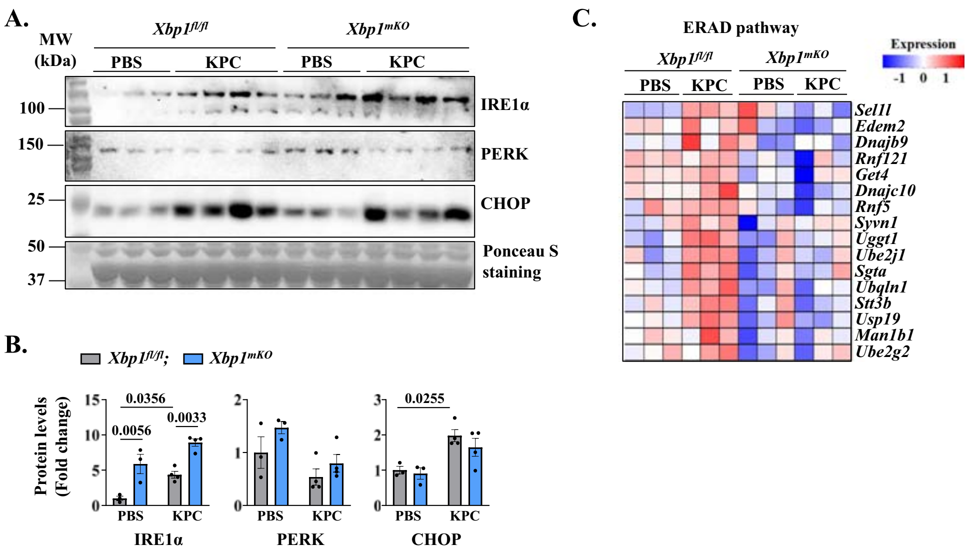

Figure EV4. Effect of targeted ablation of XBP1 on levels of UPR markers.

(**A**) Immunoblots and (**B**) densitometry analysis of protein levels of IRE1α, PERK, and CHOP in gastrocnemius (GA) muscle of *Xbp1*$^{fl/fl}$ and *Xbp1*^{mKO} mice injected with PBS or KPC cells. $n = 3$–4 mice per group. Data information: Data are presented as mean ± SEM. Indicated *P* values were calculated using two-way ANOVA followed by Tukey's multiple comparison test. (**C**) Heatmap representing relative gene expression of various molecules involved in ER associated degradation (ERAD) pathway in GA muscle of control and KPC tumor-bearing *Xbp1*$^{fl/fl}$ and *Xbp1*^{mKO} mice analyzed using bulk RNA-seq dataset. Source data are available online for this figure.

 © The Author(s)

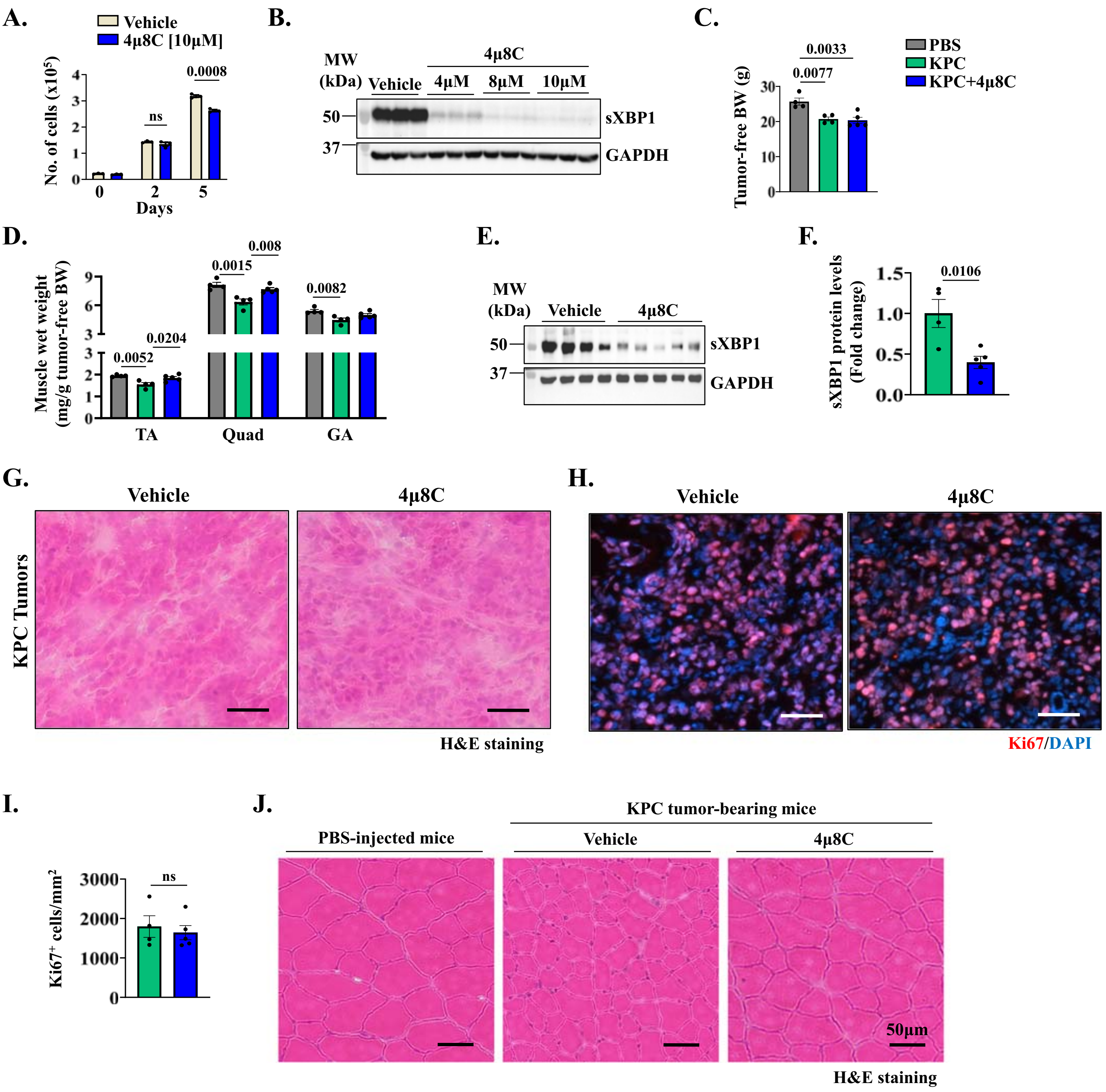

Figure EV5. Effect of pharmacological inhibition of IRE1α/XBP1 axis on cancer cachexia.

(A) Quantification of number of proliferating KPC cells on day 0, 2 and 5 after treatment with vehicle alone or 10 μM 4μ8C. $n = 3$ biological replicates per group. Data information: Data are presented as mean ± SEM. Indicated *P* values were calculated using unpaired Student *t* test. (B) Immunoblot showing levels of sXBP1 protein in KPC cells treated with vehicle alone or indicated concentrations of 4μ8C for 24 h. (C) Quantification of tumor-free body weight (BW) of control and KPC tumor-bearing mice treated with vehicle alone or 4μ8C after 18 days of KPC cells injection into the pancreas. (D) Quantification of wet weight of TA, Quad, and GA muscle normalized by tumor-free BW. $n = 4$–5 mice per group. Data information: Data are presented as mean ± SEM. Indicated *P* values were calculated using one-way ANOVA, followed by Tukey's multiple comparison test. (E) Immunoblot and (F) densitometry analysis showing levels of sXBP1 protein in KPC tumors of mice treated with vehicle alone or 4μ8C. Data information: Data are presented as mean ± SEM. Indicated *P* values were calculated using unpaired Student *t* test. (G, H) Representative photomicrographs of KPC tumors after (G) H&E staining, or (H) anti-Ki67 and DAPI staining. Scale bar, 50μm. (I) Quantification of number of Ki67$^+$ cells per unit area (mm^2) in KPC tumors of mice treated with vehicle alone of 4μ8C. Data information: No significant difference was observed using unpaired Student *t* test. (J) Transverse sections of TA muscle of control and vehicle or 4μ8C-treated KPC tumor-bearing mice were generated and used for H&E staining. Representative photomicrographs of H&E-stained sections are presented here. Scale bar, 50 μm. Source data are available online for this figure.

© The Author(s)
